# Decatungstate‐Mediated C(sp^3^)–H Heteroarylation via Radical‐Polar Crossover in Batch and Flow

**DOI:** 10.1002/anie.202104682

**Published:** 2021-07-09

**Authors:** Ting Wan, Luca Capaldo, Gabriele Laudadio, Alexander V. Nyuchev, Juan A. Rincón, Pablo García‐Losada, Carlos Mateos, Michael O. Frederick, Manuel Nuño, Timothy Noël

**Affiliations:** ^1^ Flow Chemistry Group Van't Hoff Institute for Molecular Sciences (HIMS) University of Amsterdam Science Park 904 1098 XH Amsterdam The Netherlands; ^2^ Department of Organic Chemistry Lobachevsky State University of Nizhny Novgorod Gagarina Avenue 23 603950 Nizhny Novgorod Russia; ^3^ Centro de Investigación Lilly S.A. Avda. de la Industria 30 28108 Alcobendas-Madrid Spain; ^4^ Small Molecule Design and Development Eli Lilly and Company Indianapolis IN 46285 USA; ^5^ Vapourtec Ltd. Park Farm Business Centre Fornham St Genevieve Bury St Edmunds Suffolk IP28 6TS UK

**Keywords:** flow chemistry, heteroarylation, hydrogen atom transfer, radical–polar crossover, TBADT

## Abstract

Photocatalytic hydrogen atom transfer is a very powerful strategy for the regioselective C(sp^3^)–H functionalization of organic molecules. Herein, we report on the unprecedented combination of decatungstate hydrogen atom transfer photocatalysis with the oxidative radical–polar crossover concept to access the direct net‐oxidative C(sp^3^)–H heteroarylation. The present methodology demonstrates a high functional group tolerance (40 examples) and is scalable when using continuous‐flow reactor technology. The developed protocol is also amenable to the late‐stage functionalization of biologically relevant molecules such as stanozolol, (−)‐ambroxide, podophyllotoxin, and dideoxyribose.

## Graphical abstract

The combination of decatungstate photocatalysis with oxidative radical–polar crossover is reported. This strategy enables the C(sp^3^)–H heteroarylation under mild conditions with excellent functional group tolerance. It was also adopted to enable late‐stage functionalization of valuable compounds. Microflow technology reduced reaction times and boosted productivity of the transformation.

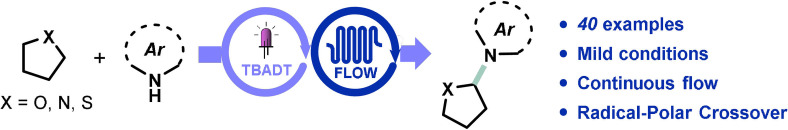

Photocatalytic hydrogen atom transfer (HAT) is witnessing an ever‐growing interest from the synthetic community as a versatile strategy for the late‐stage functionalization of C(sp^3^)−H bonds.[^1^, ^2^, ^3^] In this activation mode, the excited state of a photocatalyst can be conveniently exploited to cleave C(sp^3^)−H bonds to obtain carbon‐centered radicals. By exploiting inherent electronic and steric properties of the parent molecule and by tuning the reaction conditions, these nucleophilic radicals can be obtained with high regioselectivity, thus obviating the need to use any directing or activating groups (Scheme 1 a).

**Scheme 1:**
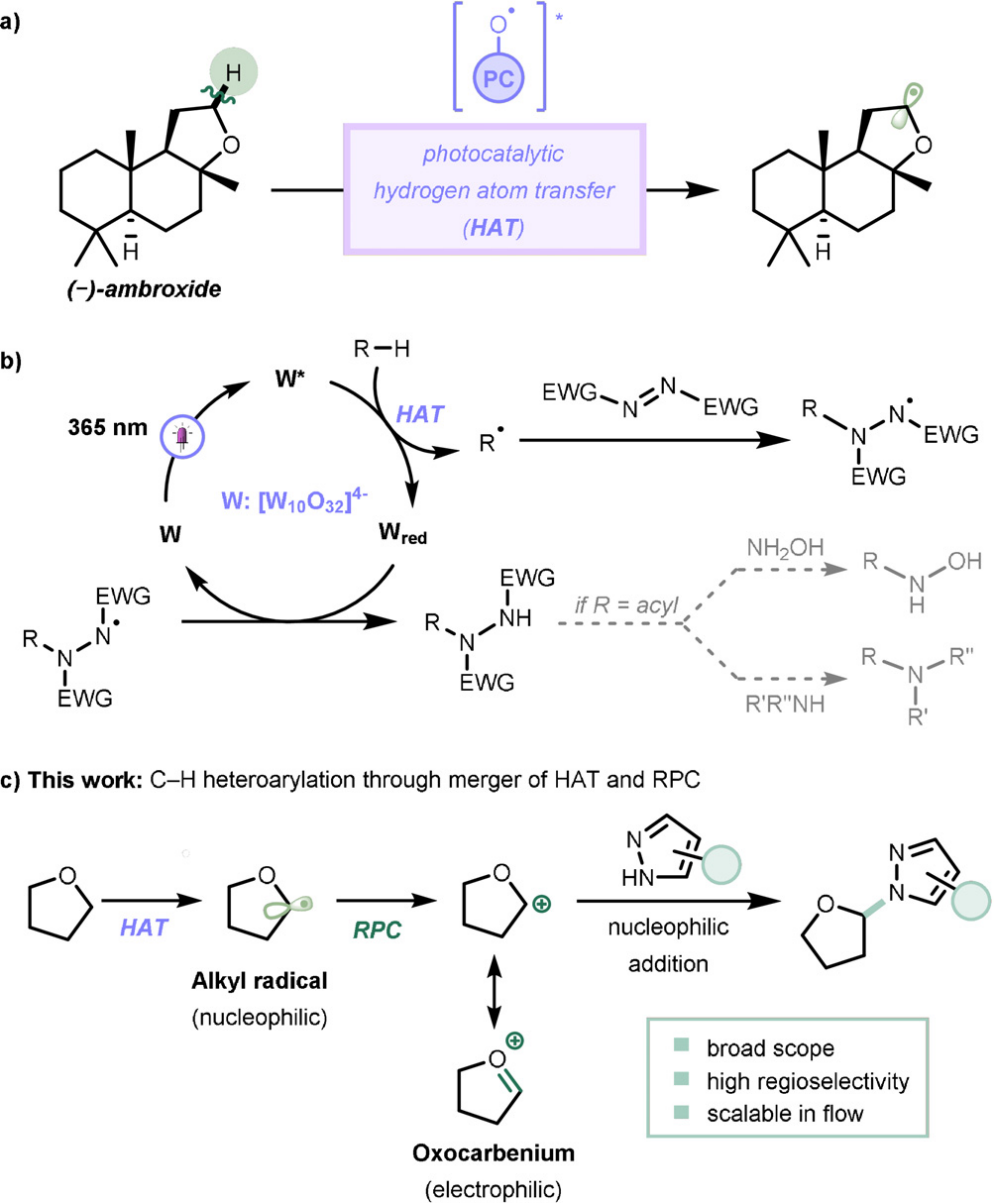
a) Photocatalytic hydrogen atom transfer (HAT) enables the conversion of C−H bonds in complex biologically active molecules. b) Established mechanism for the formation of C−N bonds via TBADT‐mediated HAT. c) Proposed approach to realize the regioselective C−H bond heteroarylation through combination of decatungstate‐enabled HAT and Radical‐Polar Crossover (RPC).

Amongst the different HAT photocatalysts, the decatungstate anion (W; [W_10_O_32_]^4−^) has proven to be an ideal candidate owing to its unique selectivity, robustness and ease of preparation.[^4^, ^5^] The excited state of W (W*) can be readily obtained upon exposure to UV‐A light (*λ*>365 nm) and has been used for the activation of C(sp^3^)−H bonds within a wide variety of hydrogen donors such as ethers, aldehydes, amides and even alkanes. In most cases, the fleeting radical intermediates were used to forge C−C,^[6]^ C−F,^[7]^ and C−O^[8]^ bonds. In contrast, only a handful of examples demonstrate the formation of C−N bonds.^[9]^ These examples mainly rely on the trapping of the radical with a suitable Michael acceptor, e.g., diisopropyl azodicarboxylate (DIAD), delivering the corresponding hydrazides. Despite its synthetic utility to access hydroxamic acids and amides,^[10]^ this approach remains fairly specific, atom‐inefficient and limited to the strongly electrophilic N=N double bond present in DIAD (Scheme 1 b).

To expand the scope of C(sp^3^)−N bond forming reactions using HAT photocatalysis, we envisioned that an unprecedented combination of a decatungstate‐induced HAT event with an oxidative radical–polar crossover (RPC) process might overcome this challenge.[^11^, ^12^, ^13^] More specifically, we surmised that subsequent oxidation of carbon‐centered radicals generated via HAT would lead to carbocations, which can be conveniently trapped with *N*‐heteroaryl‐based nucleophiles, thus establishing the targeted carbon–nitrogen bond. A crucial aspect in the development of such a methodology is the nature of the carbocation, which should not only be readily generated but also be sufficiently stable to enable interception with the selected nucleophile. We speculated that the generation of an oxocarbenium ion would provide the required stabilization of the carbocation (Scheme 1 c).

Herein, we report the development of an efficient TBADT‐mediated (TBADT: tetrabutylammonium decatungstate, (Bu_4_N)_4_W_10_O_32_) heteroarylation of C(sp^3^)−H bonds through the merger of HAT and RPC, thus demonstrating a new reactivity mode for this widely used HAT photocatalyst. Notably, as shown in this communication, the methodology is amenable to the late‐stage functionalization of complex organic molecules and is scalable when using continuous‐flow reactor technology.

Our initial investigations commenced with the coupling of tetrahydrofuran (**1 a**) and pyrazole (**2 a**) (Table 1). For this purpose, an acetonitrile solution of **1 a** and **2 a** in the presence of TBADT (2 mol %) was irradiated with UV‐A light (*λ*=365 nm, 36 W) for 16 hours. In order to trigger the RPC event, we also added TBHP (*tert*‐butylhydroperoxide) as a terminal oxidant. After extensive screening of potential reaction conditions (see the Supporting Information), we found that the target product **3** could be obtained in excellent yield using an excess of **1 a** and 3 equivalents of TBHP (Table 1, Entry 1). Reducing the amount of TBHP from 3 to 1 equivalents resulted in decreased yields (Table 1, Entries 2,3). Replacing TBHP with any other oxidant did not lead to improved results (Table 1, Entries 4–7). As expected, when no oxidant was added, the RPC event failed and the nucleophilic addition was precluded; under these circumstances, the desired product **3** was formed only in traces (Table 1, Entry 8). No product was observed in the absence of light and photocatalyst (see the Supporting Information). Finally, in order to reduce the required reaction times and to enable scalability of the reaction protocol,[^14^, ^15^] we optimized a flow process (Table 1, Entries 9–11) by using the commercially available photochemical reactor Vapourtec UV‐150 (PFA, inner diameter=1.3 mm, see Supporting Information). Thus, we were able to increase efficiency up to 81 % isolated yield (Table 1, Entry 11).

**Table 1:** Optimization of reaction conditions.^[a]^ 
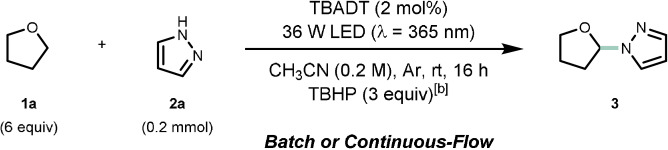

Entry	Variation from conditions	Yield^[c]^
**1**	**none**	**86**
2	TBHP (2 equiv)	80
3	TBHP (1 equiv)	70
4^[d]^	O_2_ atmosphere	4
5	H_2_O_2_ (3 equiv)	32
6	DTP (3 equiv)	20
7	BPO (3 equiv)	52
8	No sacrificial oxidant	traces
9^[e]^	flow, *t*_R_=1 h; TBADT (5 mol %)	64
10^[f]^	flow, *t*_R_=1 h; TBADT (5 mol %), 60 W LED	74
11	flow, *t*_R_=1 h; 60 W LED, TBADT (5 mol %); **1 a** (18 equiv)	86 (81)

[a] **1 a** (6 equiv), **2 a** (0.2 mmol), TBADT (2 mol %), TBHP (3 equiv) in CH_3_CN (1 mL); solution sparged with Ar prior to irradiation (*λ*=365 nm, 16 h). [b] TBHP (5.5 M in decane or nonane). [c] Yields determined by ^1^H NMR spectroscopy using pyrazine as external standard. [d] reaction mixture sparged with O_2_ prior to irradiation and kept under aerobic atmosphere (balloon filled with O_2_). [e] flow setup 1 (see SI): *V*_R_=6 mL, flow rate=0.1 mL min^−1^, *t*_R_=1 h, 36 W LED (*λ*=365 nm). [f] flow setup 2 (see SI): *V*_R_=10 mL, flow rate=0.167 mL min^−1^, *t*_R_=1 h, 60 W LED (*λ*=365 nm). In parenthesis is isolated yield.

With optimal reaction conditions in both batch and flow established, we examined the generality of our photocatalytic transformation (Scheme 2). We commenced by combining model substrate **1 a** with a set of structurally diverse nitrogen‐containing heteroarenes. Our benchmark reaction between tetrahydrofuran (**1 a**) and pyrazole (**2 a**) could be readily scaled up in flow using the standard procedure for prolonged operation times (10 mmol, 80 % isolated yield). Alkyl‐ and aryl‐bearing pyrazoles afforded the desired cross‐coupled products in fair to excellent yields (**4**–**8**, 44–94 % yield). Furthermore, pyrazoles decorated with electron‐withdrawing moieties (e.g. chloro **9**, bromo **10** and ethyl‐ester **11**) proved to be competent substrates as well (68–83 %). A pyrazole containing the boronic acid pinacol (Bpin) ester functionality was tolerated under the reaction conditions (**12**, 81 % yield); notably, this boron‐functionality can serve as a branching point for further diversification using Suzuki–Miyaura or Chan‐Evans‐Lam cross‐coupling chemistry.[^16^, ^17^] Next, we successfully extended this C(sp^3^)–H heteroarylation protocol to other *N*‐containing five‐membered rings such as imidazoles (**13**–**14,** 42–76 %), triazoles (**15**, 96 %) and tetrazoles (**16**, 46 %), as well as various benzo‐fused heteroaromatic structures (**17**–**19**, 42–55 %). Furthermore, several in nature‐occurring heterocycles, such as xanthines (**20** and **21**, 18–60 %) and purines (**22**–**25**, 24–63 %), served as adequate coupling partners. Most of the latter reactions were run in batch owing to the limited solubility of the heteroaromatic nucleophiles. Remarkably, we also managed to functionalize anabolic steroid stanozolol to get product **26** with an excellent mass balance (34 % yield, 87 % brsm).

**Scheme 2:**
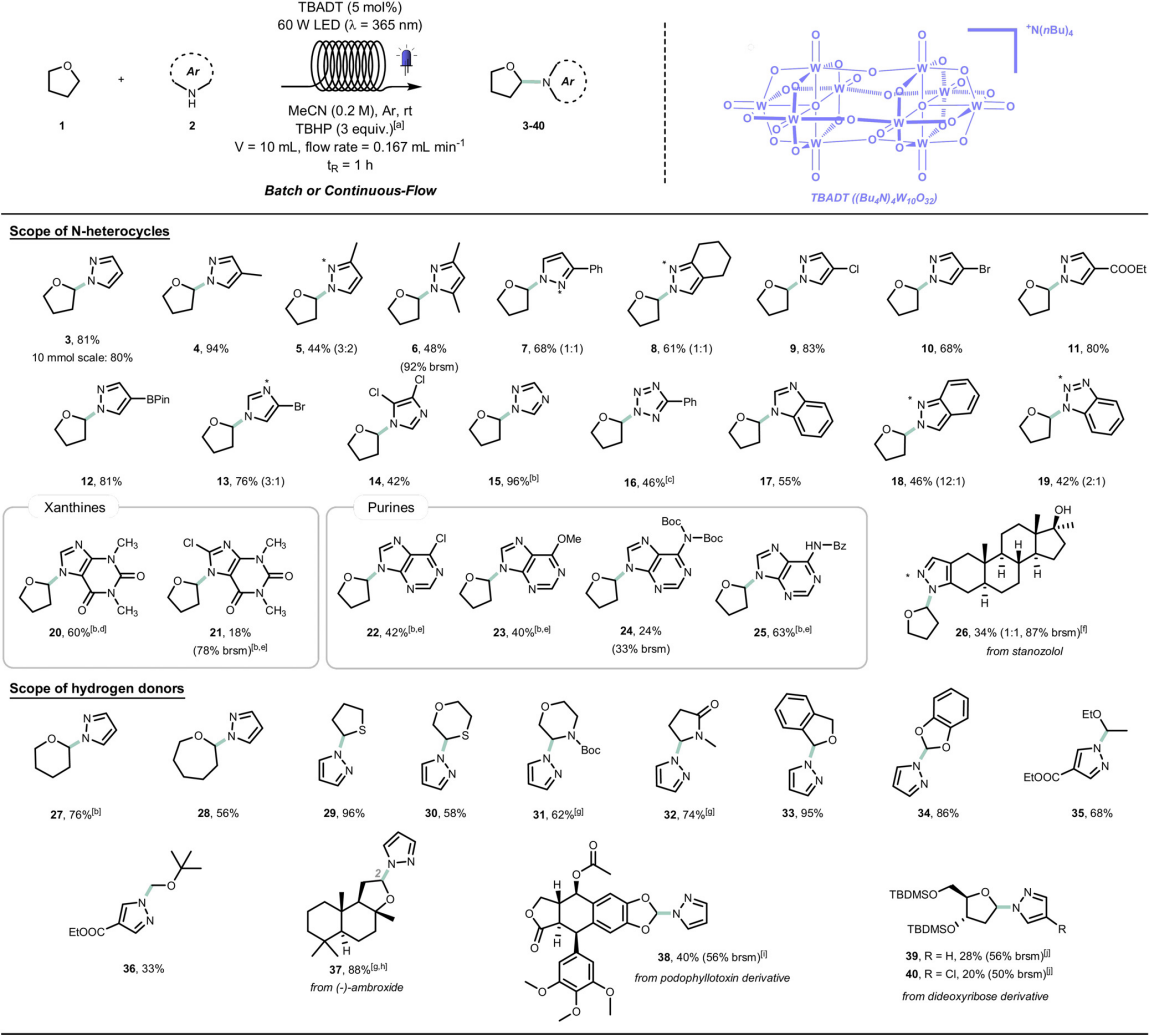
Substrate scope of the decatungstate‐mediated C(sp^3^)−H heteroarylation via radical‐polar crossover in batch and flow. **1 a** (18 equiv), **2 a** (1.0 mmol), TBADT (5 mol %), TBHP (3 equiv) in CH_3_CN (5 mL); solution sparged with Ar prior to irradiation (*λ*=365 nm 60 W, reactor volume: 10 mL, flow rate: 0.167 mL min^−1^, *t*_R_: 1 h). Isolated yields are given. [a] TBHP was used 5.5 M in decane or nonane. [b] Reaction carried out in batch (16 h); TBADT (5 mol %). [c] CH_3_CN (12 mL). [d] CH_3_CN/CHCl_3_ 1:1 (10 mL); **1 a** (18 equiv). [e] Solvent mixture: CH_3_CN/**1 a** 1:1 (10 mL). [f] Solvent mixture: CH_3_CN/**1 a** 2:5 (14 mL). [g] 6 equiv of the hydrogen donor were used. [h] CH_3_CN/CH_2_Cl_2_ 5:1 (6 mL). [i] 2 equiv of hydrogen donor were used, CH_3_CN (15 mL). [j] Reaction carried out as in Table 1, entry 1; 2 equiv of hydrogen donor were used. Brsm: based on remaining starting material.

Next, we investigated the scope of suitable H‐donors. Efficient α‐to‐O C−H functionalization was achieved for both tetrahydropyran and oxepane, providing the targeted compounds **27** and **28** in good yields (76 % and 56 %, respectively). Also α‐to‐S and α‐to‐N C−H bond functionalization was observed (**29**–**32**, 58–96 %) with great selectivity over competitive α‐to‐O C−H activation. In the case of very activated methylenes, such as in benzo[*b*]furan and 1,3‐benzodioxole, excellent yields of the corresponding products **33** (95 %) and **34** (86 %) were observed. Acyclic ethers such as diethyl ether and methyl *tert*‐butyl ether could be used as substrates as well, yielding products **35** and **36** in 68 % and 33 % yield, respectively. The ability of this method to enable the late‐stage functionalization of complex organic molecules or natural scaffolds was demonstrated in the case of (−)‐ambroxide and acetyl‐protected podophyllotoxin; these substrates were functionalized in 88 % (**37**) and 40 % (**38**), respectively. Finally, silyl‐protected dideoxyribose was subjected to our reaction conditions and we found that it could be functionalized in satisfactory yields (**39**–**40**, 20–28 % yield, 50–56 % brsm).

A plausible mechanistic rationale for the C(sp^3^)‐H heteroarylation is shown in Scheme 3 a. Upon absorption of UV‐A light, the excited state of TBADT (W*) is able to cleave the C−H bond in α‐position to the heteroatom (i.e., O, N or S) yielding a carbon‐centered nucleophilic α‐oxyalkyl radical (**I^.^**). This radical intermediate (**I^.^**) is readily oxidized by TBHP resulting in the formation of a stabilized electrophilic oxocarbenium ion (**I^+^**), which is trapped by the heteroarene establishing the targeted C−N bond.

**Scheme 3:**
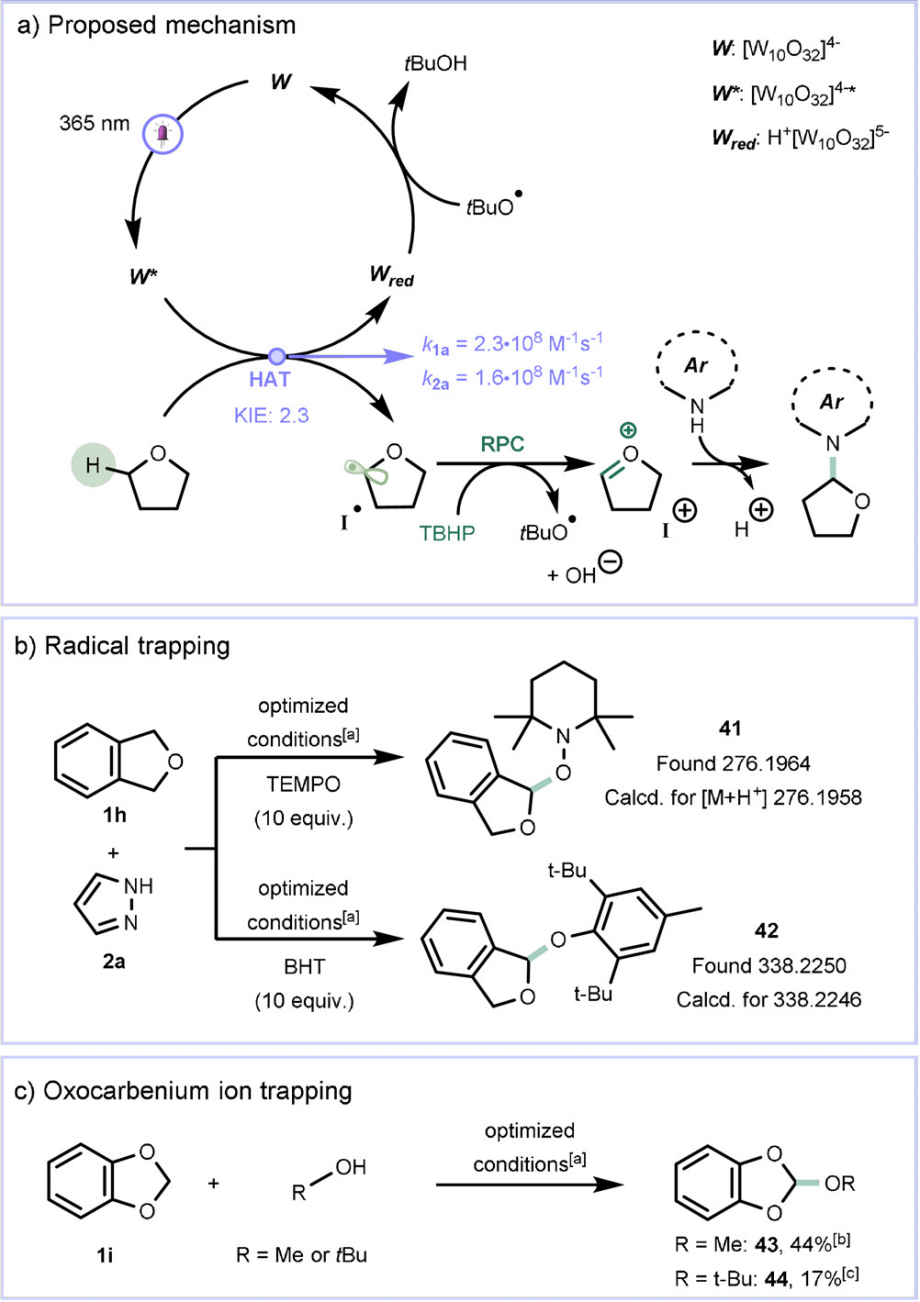
Mechanistic investigation: a) Proposed mechanism with KIE and quenching rates. b) Radical trapping experiments. c) Oxocarbenium trapping experiments. [a] **1 h**,**i** (18 equiv), **2 a** (1.0 mmol), TBADT (5 mol %), TBHP (3 equiv) in CH_3_CN (5 mL); solution sparged with Ar prior to irradiation (*λ*=365 nm 60 W, reactor volume: 10 mL, flow rate: 0.167 mL min^−1^, *t*_R_: 1 h). [b] Yield determined by ^1^H NMR spectroscopy using pyrazine as external standard. [c] Isolated yield.

Using laser flash photolysis, the quenching of the excited state of TBADT (W*) was studied in more detail (see Supporting Information, Section 3.4). From the derived Stern–Volmer plots, the quenching rates with both substrates **1 a** and **2 a** were calculated. Interestingly, both tetrahydrofuran and pyrazole are able to quench W* with comparable bimolecular rates (*k*=2.3 and 1.6×10^8^ M^−1^ s^−1^, respectively). However, given the excess of **1 a**, it is reasonable to state that tetrahydrofuran (**1 a**) is the actual quencher in the reported experiments, thus delivering **I^.^**. This assumption was further confirmed in radical trapping experiments by the detection of the corresponding adducts of the α‐oxyalkyl radical with radical scavengers (**41** and **42**, as evidenced by HRMS) (Scheme 3 b). Interestingly, a significant Kinetic Isotope Effect (KIE) of 2.3 was measured, which is in accordance with HAT being the rate‐determining step (see Supporting Information, Section 3.3). Quantum yield measurements (*Φ*=0.18) further ruled out a radical chain mechanism (See Supporting Information, Section 3.5). Next, we set out to trap the oxocarbenium ionic intermediate by adding an excess of CH_3_OH or *t*‐BuOH (18 equiv) (Scheme 3 c).^[18]^ In both cases, the corresponding orthoesters **43** and **44** could be detected via HRMS and ^1^H NMR spectroscopy. Despite significant efforts, this C(sp^3^)–H heteroarylation method could not be extended to unactivated aliphatic C(sp^3^)−H bonds (see the Supporting Information, Section 5 for limitations of the scope). We surmise that the oxidation of **I^.^** to **I^+^** is only favored when the latter species is strongly stabilized, e.g., as an oxocarbenium species.

It should be noted that our methodology compares favorably to thermal approaches relying on TBAI/TBHP^[19]^ (TBAI=tetrabutylammonium iodide) and Fe^III^/TBHP^[20]^ systems as it requires more controlled and milder conditions to generate the oxocarbenium ion, resulting in a broader functional group tolerance. Furthermore, in comparison to other photocatalytic^[21]^ and (photo)electrochemical methods,[^22^, ^23^] the developed approach is based on a direct and mild C(sp^3^)−H cleavage, presents a broader scope and does not require pre‐functionalization of the starting materials.

In conclusion, a convenient methodology to forge C(sp^3^)−N bonds by combining the radical–polar crossover concept with decatungstate HAT photocatalysis has been realized. Due to the mild reaction conditions, this protocol is amenable both to early and late‐stage functionalization of organic molecules. As such, this synthetic method is valuable for the synthesis of medicinal and agrochemical intermediates. While this is the first report to combine decatungstate‐induced HAT with oxidative RPC, we believe that the insights gained herein will inspire further advances in the use of this strategy to enable other challenging synthetic transformations.

## Conflict of interest

The authors declare no conflict of interest.

## Supporting information

As a service to our authors and readers, this journal provides supporting information supplied by the authors. Such materials are peer reviewed and may be re‐organized for online delivery, but are not copy‐edited or typeset. Technical support issues arising from supporting information (other than missing files) should be addressed to the authors.

Supporting Information
