# Supplementary material for: Decatungstate‐Mediated C(sp3)–H Heteroarylation via Radical‐Polar Crossover in Batch and Flow
Source: Angew Chem Int Ed Engl. 2021 Jul 9;60(33):17893–7. doi: 10.1002/anie.202104682 (PMC8457183; doi:10.1002/anie.202104682)
Supplement: Supplementary file 1 — Supporting Information [file ANIE-60-17893-s001.pdf]

Angewandte Chemie
Eine Zeitschrift der Gesellschaft Deutscher Chemiker

Supporting Information

Decatungstate-Mediated C(sp^3)–H Heteroarylation via Radical-Polar Crossover in Batch and Flow

*Ting Wan, Luca Capaldo, Gabriele Laudadio, Alexander V. Nyuchev, Juan A. Rincón, Pablo García-Losada, Carlos Mateos, Michael O. Frederick, Manuel Nuño, and Timothy Noël**

anie_202104682_sm_miscellaneous_information.pdf

Table of Contents

1. General information 2

Reactor Design 2

Homemade Setup 2

Vapourtec system 3

Synthesis of the photocatalyst (TBADT) 4

Synthesis of Starting Materials 5

Synthesis of **1m** 5

Synthesis of **1n** 5

Synthesis of **2v** 6

2. Chart of Starting Materials 7

3. Optimization of reaction conditions 8

4. Mechanistic Investigation 10

3.1 Radical Trapping Experiments 10

3.2 Oxocarbenium Ion Trapping Experiments 10

3.3 Intermolecular Kinetic Isotopic Effect (KIE) 13

3.4 Laser Flash Photolysis 14

3.5 Quantum yield measurement 16

5. Limitation of the scope 18

6. General procedure 19

General Procedure 1 (GP1), batch conditions: 19

General Procedure 2 (GP2), continuous-flow conditions: 19

7. Characterization data 20

8. Reference 34

9. NMR Spectra of starting materials **1m** and **1n** 35

10. NMR Spectra of compounds **3**-40 37

1. General information

All reagents and solvents were used as received without further purification. Reagents and solvents were bought from Sigma Aldrich, TCI and Flurochem. Technical solvents were bought form VWR International and Biosolve, and are used as received. The catalyst TBADT was prepared as illustrated below. According such procedure, the catalyst cost can be estimated around 0.7 €/g, employing 20 g tungstic acid (Sigma-Aldrich, BioUltra 72069) and 9.6 g of tetrabutylammonium bromide (Sigma-Aldrich, ReagentPlus, 193119), leading to 49 g of TBADT. For the preliminary experiment, LED strips (365 nm, 2.5 m, 300 SMD5050 LEDs, 36 W) were purchased form LedLightingHut. For scale-up, Vapourtec device was used, equipped with 60 W 365 nm LEDs. Disposable syringes were purchased from Laboratory Glass Specialist. Syringe pumps were purchased from Chemix Inc. model Fusion 200 Touch. Product isolation was performed manually, using silica (P60, SILICYCLE) or automatically by a Biotage® Isolation Four, with Biotage® SNAP KP-Sil 10 or 50 g flash chromatography cartridges. TLC analysis was performed using Silica on aluminum foils TLC plates (F254, SILICYCLE) with visualization under ultraviolet light (254 nm and 365 nm) or appropriate TLC staining (Cerium Ammonium Molybdate). ^{1}H (400 MHz) and ^{13}C (100 MHz) spectra were recorded at ambient temperature using a Bruker-Avance 400. ^{1}H NMR spectra are reported in parts per million (ppm) downfield relative to $CDCl_3$ (7.26 ppm) and all ^{13}C NMR spectra are reported in ppm relative to $CDCl_3$ (77.16 ppm) unless stated otherwise. The multiplicities of signals are designated by the following abbreviations: s (singlet), d (doublet), t (triplet), q (quartet), m (multiplet), dd (doublet of doublets), dt (doublet of triplets), td (triplet of doublets), ddd (doublet of doublet of doublets). Coupling constants (J) are reported in hertz (Hz). NMR data was processed using the MestReNova 14 software package. Known products were characterized by comparing to the corresponding ^{1}H NMR and ^{13}C NMR with those available in the literature. The melting points were measured using a Büchi Melting Point M-565 apparatus. High resolution mass spectra (HRMS) were collected on an AccuTOF LC, JMS-T100LP Mass spectrometer (JEOL, Japen).

Reactor Design

Homemade Setup

To assess the scope of the transformation reported, as well as for preliminary experiments, we adopted the homemade setup shown in Figure S1, left. It consists of a 3D-printed (PLA) reactor (inner diameter: 12.5 cm) that has been internally coated with LED strips (365 nm, 2.5 m, 300 SMD5050 LEDs, 36W). Cooling was applied via a strong compressed air flow to keep the temperature below 30 °C.

When the reaction was run in *batch-mode* (see entries **15, 20 – 23, 25, 27** in Scheme 2 or for preliminary experiments), the reactor was capped with a 3D-printed (PLA) lid with 8 holes serving as vials holder; in this way, up to 8 reactions could be run simultaneously (Figure S1, center).

When the reaction was run in *flow-mode* (referred to as "**flow setup 1**" in the main text) a different lid embedding a 3D-printed cylinder wrapped in PFA tubing was used (Figure S1, right). The total volume of the reactor was 6.0 mL and the cylinder was covered with reflective tape to increase the efficiency of the setup. Dimensions of the cylinder: length 9 cm, diameter 7.5 cm. Dimensions of the lid: diameter 14.5 cm In this case, the reaction mixture was pumped via a syringe pump and irradiated for the indicated time.

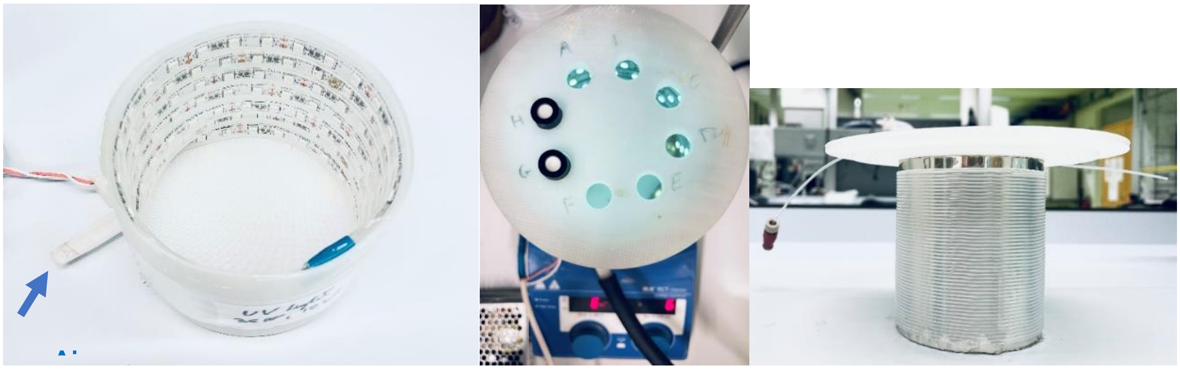

Figure S1: Left: 3D-printed homemade reactor used for preliminary experiments. **Center:** reactor lid for reactions run in *batch-mode*. **Right:** reactor lid for reactions run in *flow-mode*.

Vapourtec system

For the final optimization experiments and the evaluation of the scope, a Vapourtec device with a UV-150 photochemical reactor was used, equipped with 60 W 365 nm LED. This system is referred to as "**flow setup 2**" in the main text.

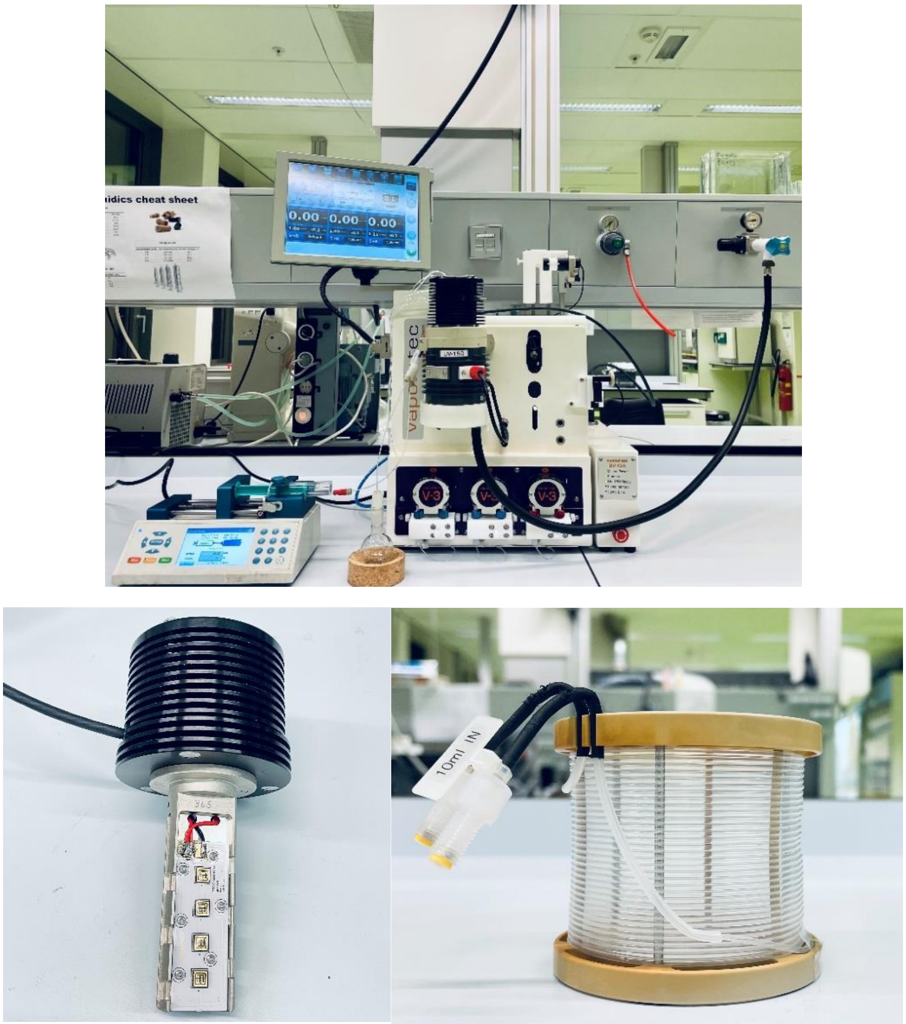

Figure S2: Overview and details of the Vapourtec system (flow setup 2): in particular, LEDs and the PFA coil (internal diameter 1.3 mm, external diameter 1.6 mm) are shown.

Synthesis of the photocatalyst (TBADT)

In a round bottom flask, tungstic acid sodium dehydrate (20 g, 0.061 mol, 1 eq, 15.5 eur) was dissolved in 5 L of demineralized water. The resultant solution was heated at 90 °C and stirred for 2 h. The solution was cooled to 0 °C and pH was adjusted with conc. hydrochloric acid to 2. In another flask, tetrabutylammonium bromide (9.6 g, 0.030 mol, 0.5 eq, 18.3 eur) was dissolved in 5 L of demineralized water. The resultant solution was heated at 90 °C and stirred for 2 h. The solution was cooled to 0 °C and pH was adjusted with conc. hydrochloric acid to 2. Finally, the solution of tetrabutylammonium bromide was poured into that of tungstic acid sodium dehydrate at room temperature. Total reaction mixture was heated again at 90 °Cand stirred for 2 h. The reaction mixture was cooled to 0 °C and filtered.

Obtained solid was diluted in CH_2Cl_2 (400 mL) and stirred at room temperature for 5 h, resulting in a white turbid mixture that was filtered to afford 16 g of crude TBADT in the residue and 5 g crude from the filtrate. The residue was treated with CH_2Cl_2 (200 mL) stirred at room temperature for 3 h, resulting again in a white turbid mixture that was filtered to afford 13 g of crude TBADT in the residue and 1.4 g crude from the filtrate. The above was repeated additional four additional 4 times to get 66.7 g of solid, which was treated with $(CH_3)_2CO$:CH_3CN 1:1 mixture (530 mL) and stirred at room temperature for 3 h and filtered. The filtrate was evaporated to get a white solid, which was triturated with *n*-pentane (300 mL) to afford 40 g of TBADT as a white solid. Parallelly, the residue was diluted with $(CH_3)_2CO$:CH_3CN 1:1 mixture (250 mL) and stirred at room temperature for 3 h. The solvent was evaporated and residue triturated with *n*-pentane (150 mL) to afford 9 g of TBADT as a white solid.

Purity of both fractions was evaluated via UV-Vis spectroscopy and matched with that of authentic samples prepared via a previously reported procedure on a smaller scale.[1]

Total yield (filtrate+residue): 49 g, 49% yield based on W.

Synthesis of Starting Materials

*Synthesis of **1m***

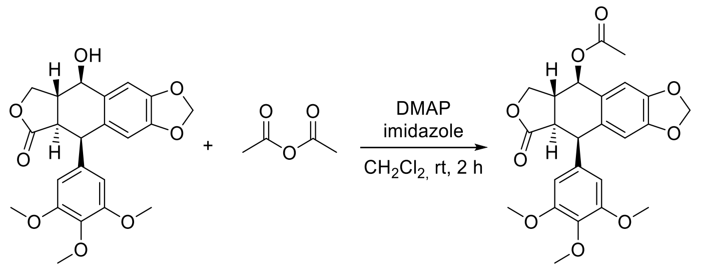

I **1m**

Product **1m** was synthesized by adapting a procedure reported in the literature.[2]
A suspension of podofyllotoxin **I** (0.62 g, 1.5 mmol) in CH_2C1_2 (15 mL) was treated with DMAP (73 mg, 0.6 mmol, 0.4 equiv), imidazole (122 mg, 1.8 mmol, 1.2 equiv) and acetic anhydride (1.5 mL). After 2 h the clear solution was washed once with saturated $NaHCO_3$ solution and twice with water. The volume was reduced over a steam bath, and MeOH added; crystals of **1m** formed upon standing (0.502 g, 73%). Spectroscopic data are in accordance with those reported in the literature (solvent: pyridine-d_5).[2]
^{1}H NMR (400 MHz, $CDCl_3$) δ 6.75 (s, 1H), 6.50 (s, 1H), 6.36 (s, 2H), 5.98 – 5.93 (m, 2H), 5.85 (d, *J* = 9 Hz, 1H), 4.56 (d, *J* = 4 Hz, 1H), 4.35 (dd, J_1 = 9 Hz, J_2 = 7 Hz, 1H), 4.16 (dd, J_1 = 10 Hz, J_2 = 9 Hz, 1H), 3.77 (s, 3H), 3.73 (s, 6H), 2.93 – 2.74 (m, 2H), 2.16 (s, 3H); ^{13}C NMR (100 MHz, $CDCl_3$) δ 173.7, 171.4, 152.7, 148.2, 147.6, 137.2, 134.9, 132.4, 128.4, 109.7, 108.1, 107.1, 101.7, 73.7, 71.4, 60.8, 56.2, 45.6, 43.8, 38.7, 21.1.

*Synthesis of **1n***

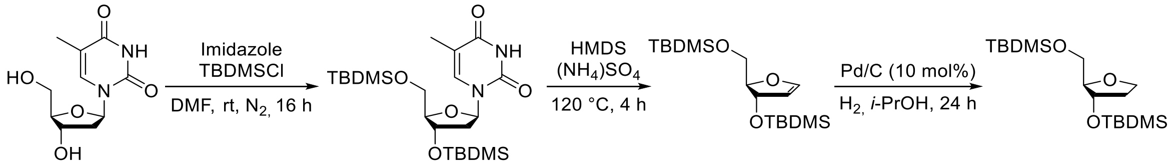

I **II** **III** **1n**

Product **1n** was synthesized by adapting a procedure reported in the literature.[3–5] A mixture of 2′-deoxythymidine (10 g, 41.3 mmol) and imidazole (11.8 g, 173 mmol, 4.2 equiv) in anhydrous DMF (60 mL) was stirred at room temperature for 5 min. Then *tert*-butyldimethylsilyl chloride (TBDMSCl; 13.1 g, 86.9 mmol, 2.1 equiv) was added, and the mixture was stirred for additional 12 h. After adding water (100 mL), the reaction mixture was extracted with hexane, dried with Na_2SO_4 and concentrated under vacuum to give **II** (15 g, 77%) as a white solid.[3]
II (2.1 g, 4.4 mmol) and $(NH_4)_2SO_4$ (1.2 g, 9.2 mmol, 2.1 equiv) were added into a oven-dried flask under N_2. HMDS (20 mL) was added, and the solution was refluxed for 4 h. The HMDS was evaporated under reduced pressure and the residue was partitioned between water and cyclohexane. The organic layer was washed with saturated $NaHCO_3$ solution and then distilled water. It was dried over Na_2SO_4 and evaporated under reduced pressure to give a yellow oil that was purified by flash chromatography (neutral Al_2O_3; cyclohexane:diethyl ether 2:1) (0.82 g, 54%).[4]

Product **III** (0.81 g, 2.35 mmol) and 10% Pd/C (15 wt%, 184 mg) in *i*-PrOH (10 mL) was vigorously stirred at room temperature under ambient pressure of H_2 for 24 h (balloon). Afterwards, the reaction mixture was filtered through a celite pad, and the filtrate was evaporated under reduced pressure. The residue was purified by column chromatography on silica gel (cyclohexane:Ethyl acetate 7:1) to give **1n** as a light yellow oil (0.74 g, 91%). Spectroscopic data are in accordance with the literature.[5]

^{1}H NMR (400 MHz, $CDCl_3$) δ 4.29 (dt, J_1 = 6 Hz, J_2 = 3 Hz, 1H), 3.95 – 3.89 (m, 2H), 3.77 – 3.73 (m, 1H), 3.62 (dd, J_1 = 11 Hz, J_2 = 4 Hz, 1H), 3.49 (dd, J_1 = 11 Hz, J_2 = 6 Hz, 1H), 2.03 – 1.93 (m, 1H), 1.83 – 1.75 (m, 1H), 0.91 – 0.87 (m, 18H), 0.09 – 0.04 (m, 12H); ^{13}C NMR (100 MHz, $CDCl_3$) δ 87.1, 73.8, 67.4, 64.0, 35.5, 26.1, 26.0, 18.5, 18.2, -4.5, -4.6, -5.2, -5.3.

Synthesis of ***2v***

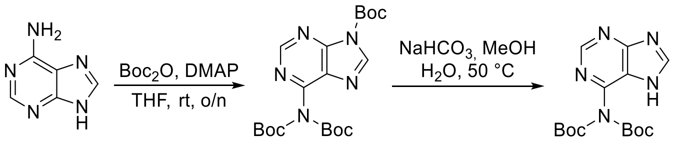

I **2v**

Product **2v** was synthesized according to a procedure reported in the literature.[6] To a 100 mL N_2-flushed round-bottomed flask equipped with a magnetic stir bar adenine (1.35 g, 10.0 mmol) and DMAP (0.122 g, 1.0 mmol) were added 50 mL of dry THF via gas-tight syringe. To the stirring suspension 8.7 g (39.8 mmol) of Boc_2O were added under N_2 atmosphere. The reaction mixture was stirred for 12 h at room temperature, after which solvent was removed by rotary evaporation to give **I** as a yellow oil. **I** (10 mmol) was taken in ethyl acetate (400 mL) and washed once with HCl 1 N (30 mL) followed by brine (3 × 100 mL). The ethyl acetate layer was dried over Na_2SO_4 and evaporated to give a colorless oil, which was used without any further purification.

This oil was dissolved in MeOH (100 mL), to which 45 mL of saturated $NaHCO_3$ solution was added. The so-obtained turbid mixture was stirred at 50 ^{o}C for 1 h to get product **2v**. The reaction can be conveniently monitored via TLC (SiO_2, cyclohexane:ethyl acetate 7:3). After evaporation of MeOH, water (100 mL) was added to the crude and the aqueous layer was extracted with $CHCl_3$ (2 × 300 mL). The organic layers were gathered and dried over Na_2SO_4, filtered, and evaporated to give a white solid. The crude material was dissolved in ethyl acetate and filtered through silica gel and washed with ethyl acetate to give 2.1 g (63% yield) of pure **2v**. Spectroscopic data are in accordance with the literature.[6]

2. Chart of Starting Materials

H-donors：

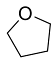

1a

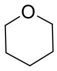

1b

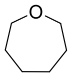

1c

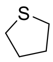

1d

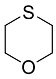

1e

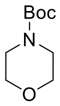

1f

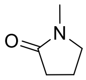

1g

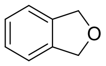

1h

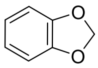

1i

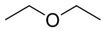

1j

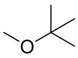

1k

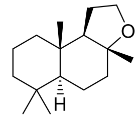

1l

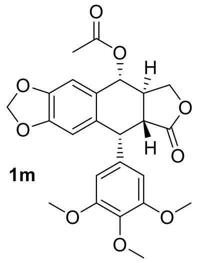

1m

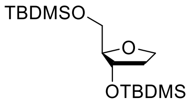

1n

N-heteroaromatics：

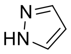

2a

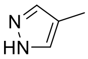

2b

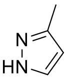

2c

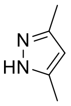

2d

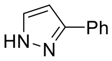

2e

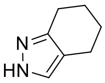

2f

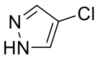

2g

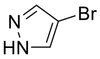

2h

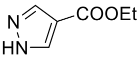

2i

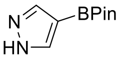

2j

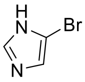

2k

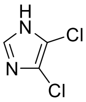

2l

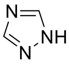

2m

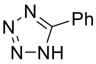

2n

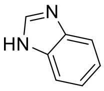

2o

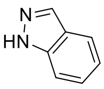

2p

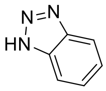

2q

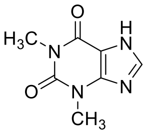

2r

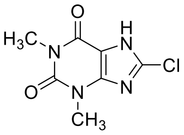

2s

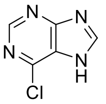

2t

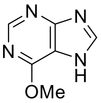

2u

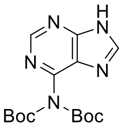

2v

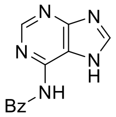

2w

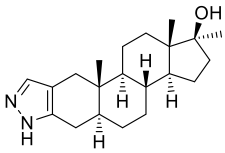

2x

3. Optimization of reaction conditions

The optimization of reaction conditions in batch was carried out by studying the cross-coupling between tetrahydrofuran (**1a**) and parent pyrazole (**2a**) in CH_3CN (1 mL) on a 0.2 mmol scale (Tables S1).
Reaction conditions: **2a** (0.2 M), **1a** (6 equiv, 1.2 M), TBADT (*n* mol%) in 1 mL of CH_3CN were mixed in a 7 mL tube and irradiated with UV-A LEDs (λ = 365 nm, 36 W) for the required time (see Figure S1). After irradiation, CD_3CN (200 µl) was added to the crude along with pyrazine as external standard and the mixture was analyzed via ^{1}H-NMR to evaluate consumption and yield.

Table S1. Optimization of reaction conditions in batch

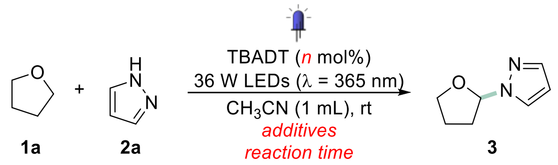

Entry	TBADT (mol%)	Additive	Time (h)	Yield (%)[a]
1[b]	2	/	16	traces
2	2	Air (balloon)	16	30
3	2	O_2 (balloon)	16	4
4	2	DTBP (2 equiv)	16	20
5	2	H_2O_2 (2 equiv)	16	32
6	2	BPO (2 equiv)	16	52
7	2	TBHP (2 equiv)	16	80
8	2	TBHP (1 equiv)	16	70
**9**	**2**	**TBHP (3 equiv)**	**16**	**86**
11	/	/	16	ND
12	/	TBHP (3 equiv)	16	ND
13[c]	2	/	16	ND
14[c]	2	TBHP (3 equiv)	16	ND

[a] Yields determined by ^{1}H NMR spectroscopy, pyrazine as external standard. [b] reaction performed under N_2.
[c] Dark. ND = not detected.

The optimization of reaction conditions in flow was carried out by studying the cross-coupling between tetrahydrofuran (**1a**) and parent pyrazole (**2a**) in CH_3CN (1 mL) on a 0.2 mmol scale (Tables S2).
Reaction conditions: **2a** (0.2 M), **1a** (6 equiv, 1.2 M), TBADT (*n* mol%) in 1 mL of CH_3CN were mixed in a 7 mL tube, the solution was withdrawn with a syringe and infused in flow setups described in Section S1 (see Figure S1 and S2). After irradiation, CD_3CN (200 µl) was added to the crude along with pyrazine as external standard and the mixture was analyzed via ^{1}H-NMR to evaluate consumption and yield.

Table S2. Optimization of reaction conditions in flow

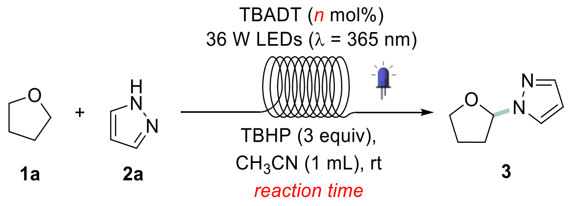

	Entry	**1a** (equiv)	TBADT (mol%)	Flow rate (mL min^{-1})	Residence time (h)	Lamp (W)	Yield (%)[a]
Flow setup 1	1	6	2	0.050	2	36	50
	2	6	5	0.050	2	36	82
	3	6	5	0.100	1	36	64
Flow setup 2	4	6	5	0.167	1	60	74
	**5**	**18**	**5**	**0.167**	**1**	**60**	**86**

[a] Yields determined by ^{1}H NMR spectroscopy, pyrazine as standard.

4. Mechanistic Investigation

3.1 Radical Trapping Experiments

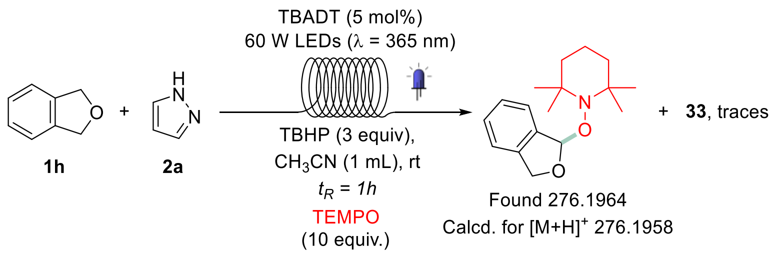

A 7 mL flame-dried tube was charged with TBADT (5 mol%), **2a** (1 mmol), TEMPO (10 equiv), CH_3CN (5 mL), TBHP (3 equiv), 1,3-dihydroisobenzofuran **1h** (18 equiv). The mixture was swirled until homogenous, placed in a 10 mL disposable syringe and mounted on a syringe pump. The flow rate was set to 0.167 mL min^{-1} to a residence time of 1 h. When the syringe was fully empty, again acetonitrile was loaded into a syringe and injected to collect all product at the end of the reactor in a flask. Pyrazine (0.5 mmol) were added to the mixture and the yield was calculated by ^{1}H-NMR. HRMS (ESI) *m/z* calcd for $C_{17}H_{26}NO_2^+$: 276.1958 $[M+H]^+$; found: 276.1964.

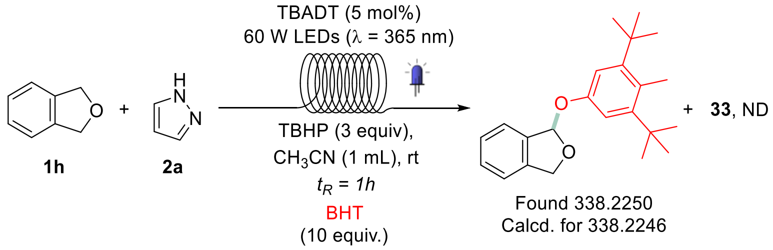

To a 7 mL flame-dried tube was added TBADT (5 mol%), **2a** (1 mmol), BHT (10 equiv), CH_3CN (5 mL), TBHP (3 equiv), 1,3-dihydroisobenzofuran **1h** (18 equiv). The mixture was swirled until homogenous, placed in a 10 mL disposable syringe and mounted on a syringe pump. The flow rate was set to 0.167 mL min^{-1} to a residence time of 1 h. When the syringe was fully empty, again acetonitrile was loaded into a syringe and injected to collect all product at the end of the reactor in a flask. Pyrazine (0.5 mmol) were added to the mixture and calculated the yield by ^{1}H-NMR. HRMS (FI) *m/z* calcd for $C_{23}H_{30}O_2$: 338.2246; found: 338.2250.

3.2 Oxocarbenium Ion Trapping Experiments

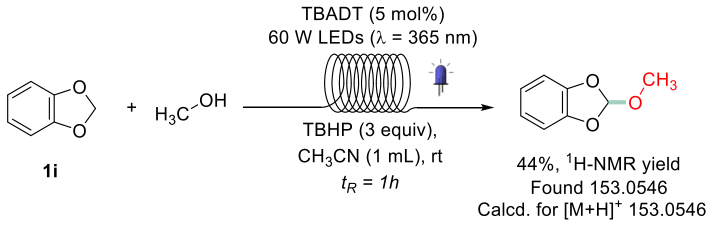

To a 7 mL flame-dried tube was added TBADT (5 mol%), **1i** (1 mmol), CH_3CN (5 mL), TBHP (3 equiv), methanol (18 equiv). The mixture was swirled until homogenous, placed in a 10 mL disposable syringe and mounted on a syringe pump. The flow rate was set to 0.167 mL min^{-1} to a residence time of 1 h. When the syringe was fully empty, again acetonitrile was loaded into a syringe and injected to collect all product at the end of the reactor in a flask. Pyrazine (0.5 mmol) were added to the mixture and calculated the yield by ^{1}H-NMR. HRMS (ESI) *m/z* calcd for $C_8H_9O_3^+$: 153.0546 $[M+H]^+$; found: 153.0546.
All attempts to separate compound **43** from starting material failed.

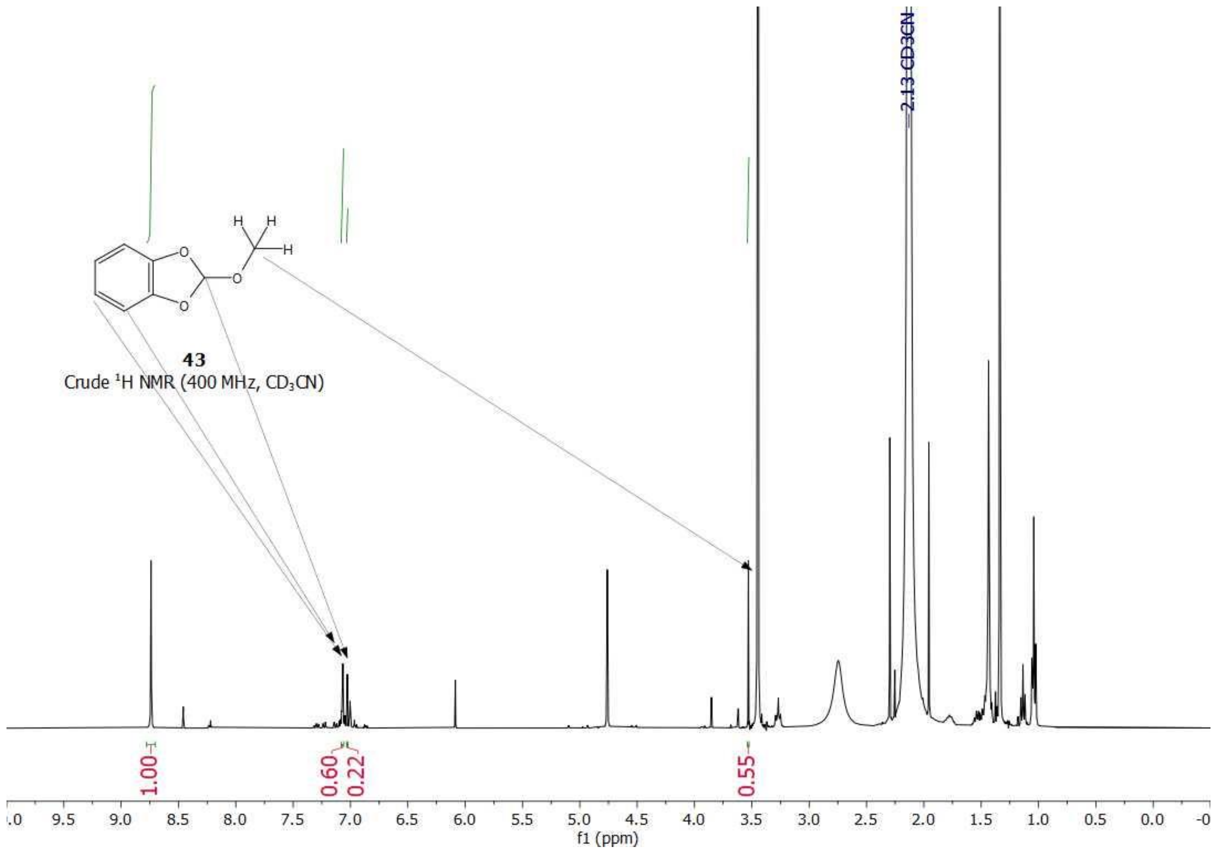

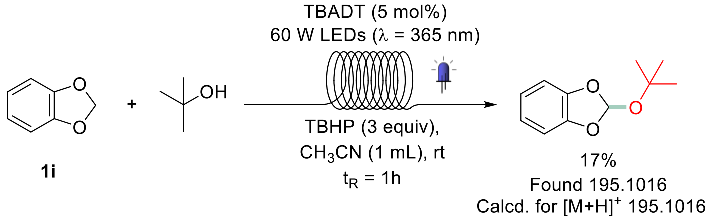

To a 7 mL flame-dried tube was added TBADT (5 mol%), **1i** (1 mmol), CH_3CN (5 mL), TBHP (3 equiv), *tert*-butanol (18 equiv). The mixture was swirled until homogenous, placed in a 10 mL disposable syringe and mounted on a syringe pump. The flow rate was set to 0.167 mL min^{-1} to a residence time of 1 h. When the syringe was fully empty, again acetonitrile was loaded into a syringe and injected to collect all product at the end of the reactor in a flask. The mixture was dried under reduced pressure and purified by column chromatography on silica gel to provide the product. The final product was weighed and characterized by HRMS, ^{1}H NMR. ^{1}H NMR (400 MHz, $CDCl_3$) δ 6.97 (s, 1H), 6.90 – 6.81 (m, 4H), 1.41 (s, 9H); ^{13}C NMR (100 MHz, $CDCl_3$) δ 145.8, 121.5, 116.8, 108.7, 28.9; HRMS (ESI) *m/z* calcd for $C_{11}H_{15}O_3^+$: 195.1016

[M+H]$^+$; found: 195.1016.

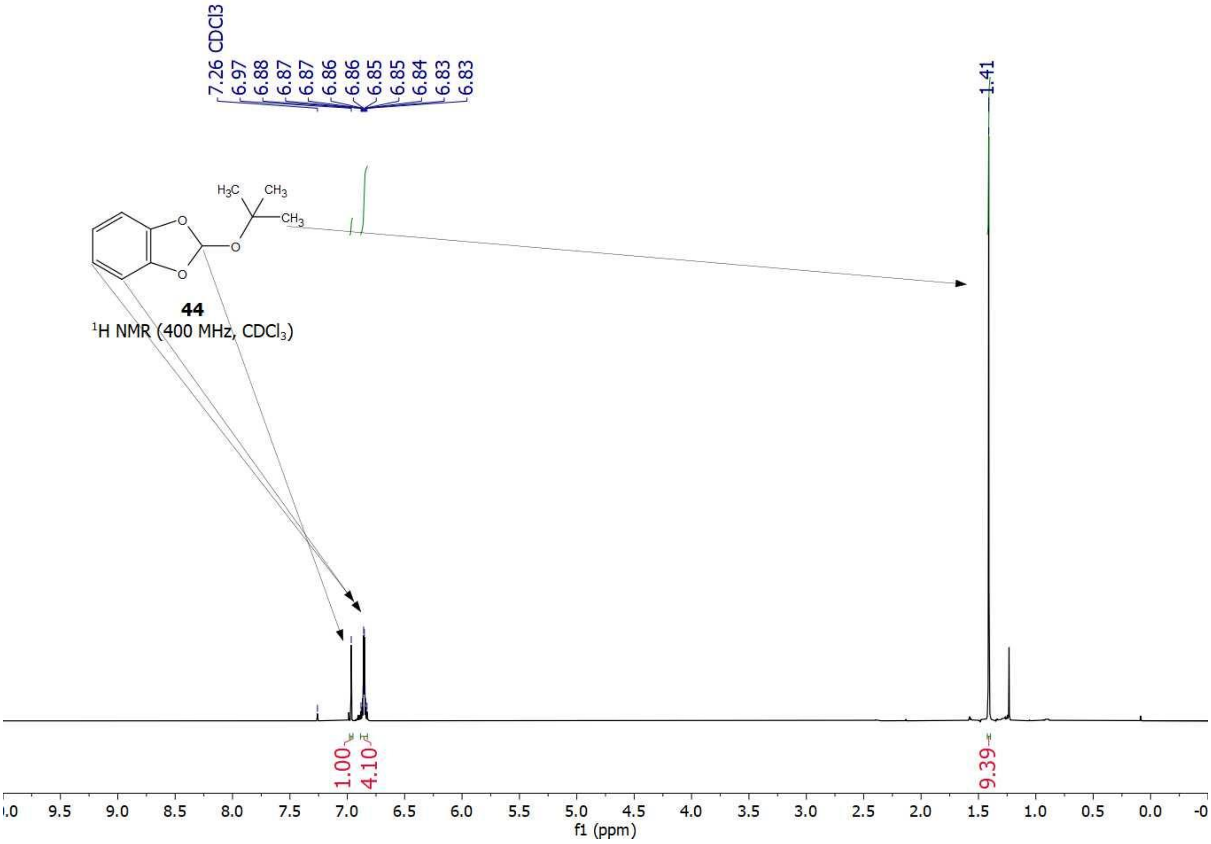

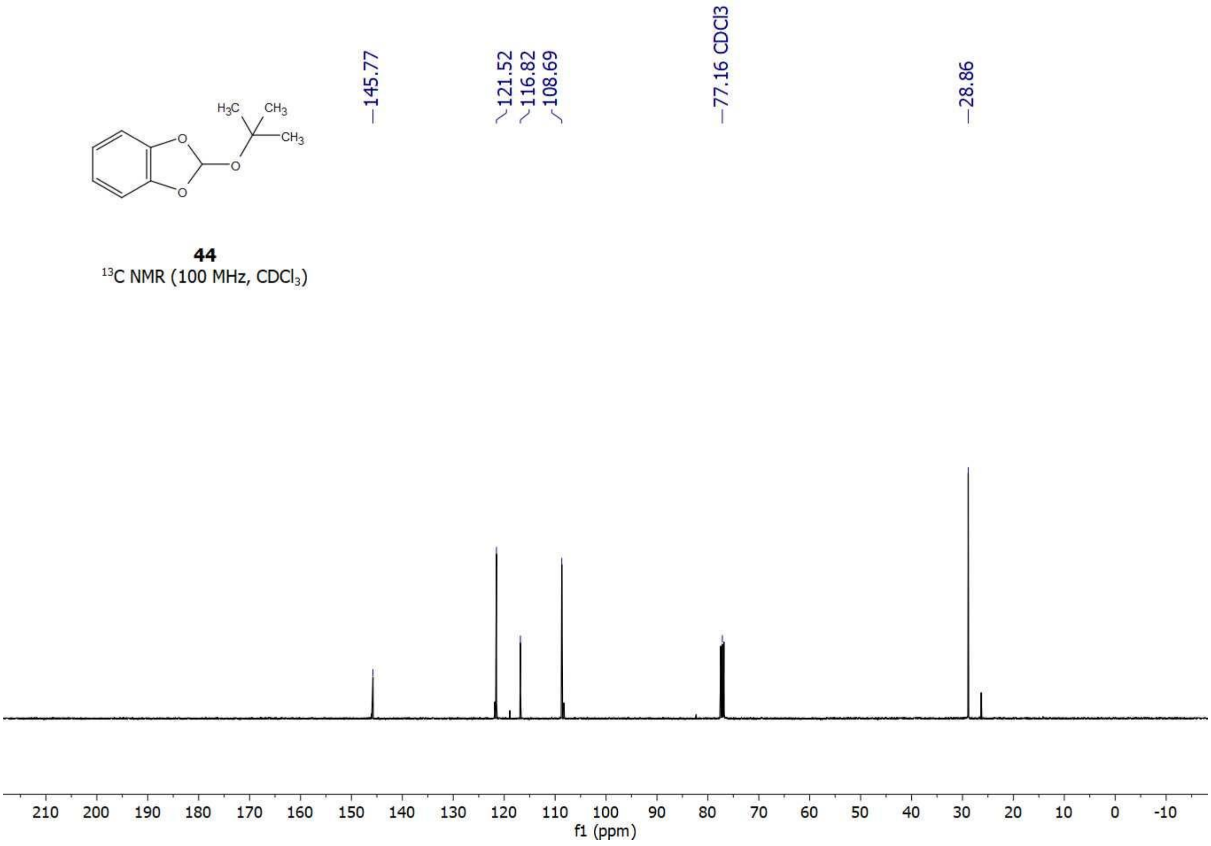

3.3 Intermolecular Kinetic Isotopic Effect (KIE)

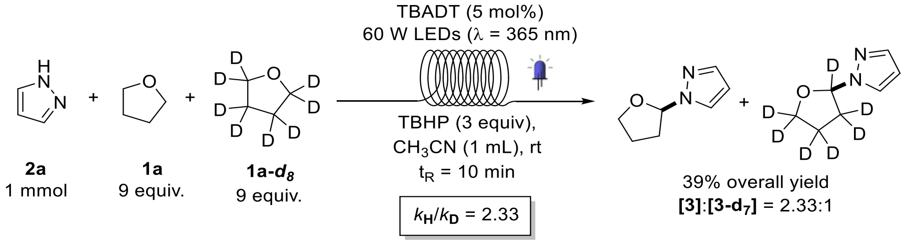

To a 7 mL flame-dried tube was added TBADT (5 mol%), **2a** (1 mmol), CH_3CN (5 mL), TBHP (3 equiv), **1a** (9 equiv), **1a-*d₈*** (9 equiv). The mixture was swirled until homogenous, placed in a 10 mL disposable syringe and mounted on a syringe pump. The flow rate was set to 0.167 mL min^{-1} to a residence time of 1 h. When the syringe was fully empty, again acetonitrile was loaded into a syringe and injected to collect all product at the end of the reactor in a flask. The mixture was dried under reduced pressure and purified by column chromatography on silica gel to provide the product. The final product was weighed and characterized by HRMS, ^{1}H NMR.

^{1}H NMR (400 MHz, $CDCl_3$) δ 7.55 (d, J = 3 Hz, 2H), 6.26 (t, J = 2 Hz, 1H), 5.99 (dd, J_1 = 7 Hz, J_2 = 3 Hz, 0.7H), 4.11 (td, J_1 = 8 Hz, J_2= 6 Hz, 0.7H), 3.98 (q, J = 7 Hz, 0.7H), 2.66 – 2.54 (m, 0.8H), 2.38 – 2.27 (m, 0.7H), 2.24 – 2.12 (m, 0.8H), 2.08 – 1.97 (m, 0.8H). HRMS (ESI) m/z calcd for $C_7H_4D_7O^+$: 146.1305 [M+H]$^+$; found: 146.1305. HRMS (ESI) m/z calcd for $C_7H_{11}N_2O^+$: 139.0866 [M+H]$^+$; found: 139.0871.

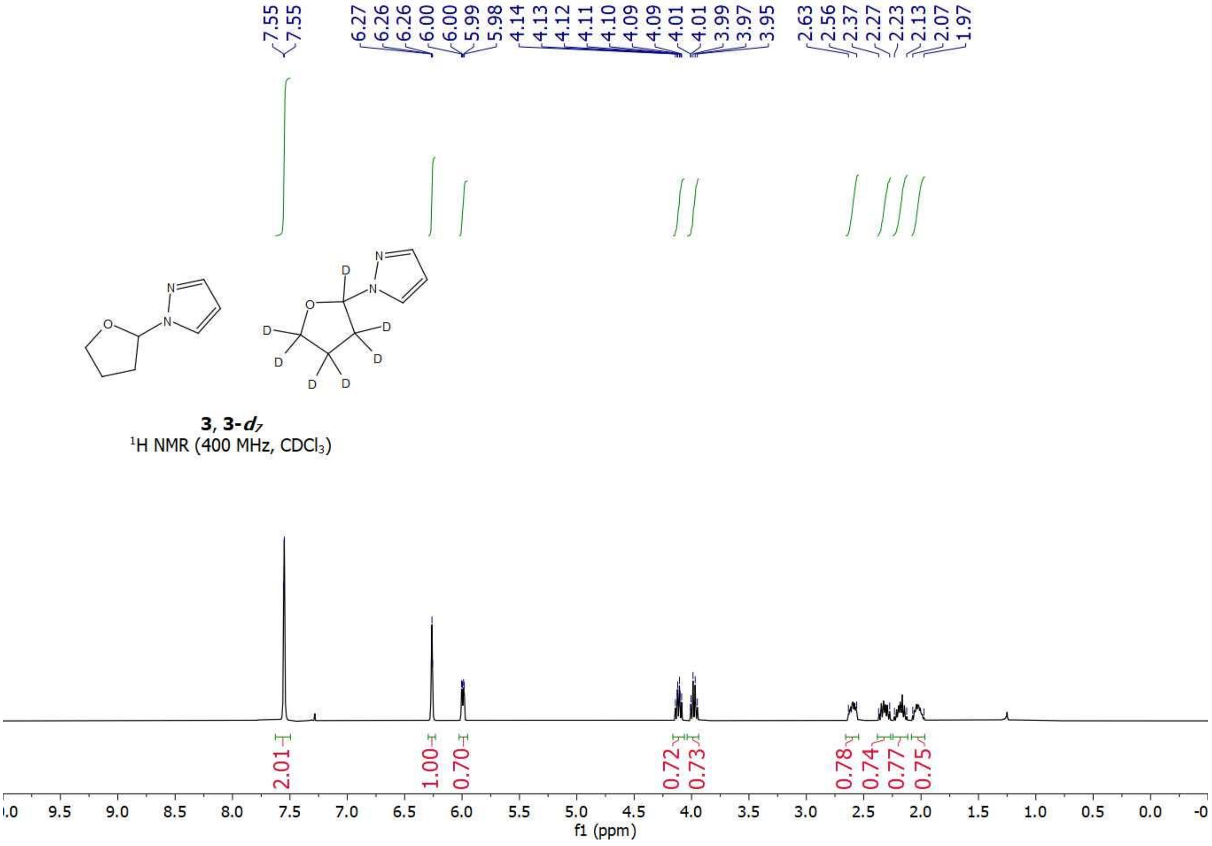

3.4 Laser Flash Photolysis

Laser flash photolysis (LFP) experiments were performed to study the decay of the reactive excited state of decatungstate (tagged W*) in the presence of increasing concentrations of quenchers **1a** and **2a**, which can be monitored at 780 nm. Thus, we measured the quenching constants for **1a** and **2a** through relevant Stern-Volmer plots and we found two comparable reaction rates. In detail, a bimolecular rate constant $k_Q = 2.3\times10^8$ $M^{-1}\cdot s^{-1}$ was measured for **1a**, while $k_Q = 1.6\times10^8$ $M^{-1}\cdot s^{-1}$ was determined for **2a**.

Experimental.

Nanosecond transient absorptions were recorded with an in-house assembled setup. An excitation wavelength of 324 nm was used. The excitation wavelength of 324 nm was generated using a tunable Nd:YAG-laser system (NT342B, Ekspla) comprising the pump laser (NL300) with harmonics generators (SHG, THG) producing 355 nm to pump an optical parametric oscillator (OPO) with SHG connected in a single device. The laser system was operated at a repetition rate of 5 Hz with a pulse length of 5ns. The probe light running at 10 Hz was generated by a high-stability short arc xenon flash lamp (FX-1160, Excelitas Technologies) using a modified PS302 controller (EG&G). Using a 50/50 beam splitter, the probe light was split equally into a signal beam and a reference beam and focused on the entrance slit of a spectrograph (SpectraPro-150, Princeton Instruments) with a grating of 150ln/mm blaze at 500nm. The probe beam (A = 1 mm2) was passed through the sample cell and orthogonally overlapped with the excitation beam on a 1 mm × 1 cm area. The excitation energy was recorded by measuring the excitation power at the back of an empty sample holder. In order to correct for fluctuations in the flash lamp spectral intensity, the reference was used to normalize the signal. Both beams were recorded simultaneously using a gated intensified CCD camera (PI-MAX3, Princeton Instruments) which has an adjustable gate of minimal 2.9 ns. Two delay generators (DG535 and DG645, Stanford Research Systems, Inc.) were used to time the excitation pulse, and to change the delay of the flash lamp and gate of the camera during the experiment. The setup was controlled by an in-house written Labview program.

1.0×10^{-4} M starting solutions of TBADT $(n\mathrm{Bu_4N})_4[W_{10}O_{32}]$ in CH_3CN were employed, with an optical density of 1.51 at 324 nm. The sample solution was placed in a 1×1 cm quartz cell and excited with single pulses (1.3 mJ, 5 Hz) delivered from the laser and analyzed with a pulsed Xe arc lamp. Lifetimes of the reactive transient W* were obtained by fitting the first order decay profiles recorded at 780 nm by using the following equation:

$$y = y_0 + A \cdot e^{-\frac{x}{\tau}}$$

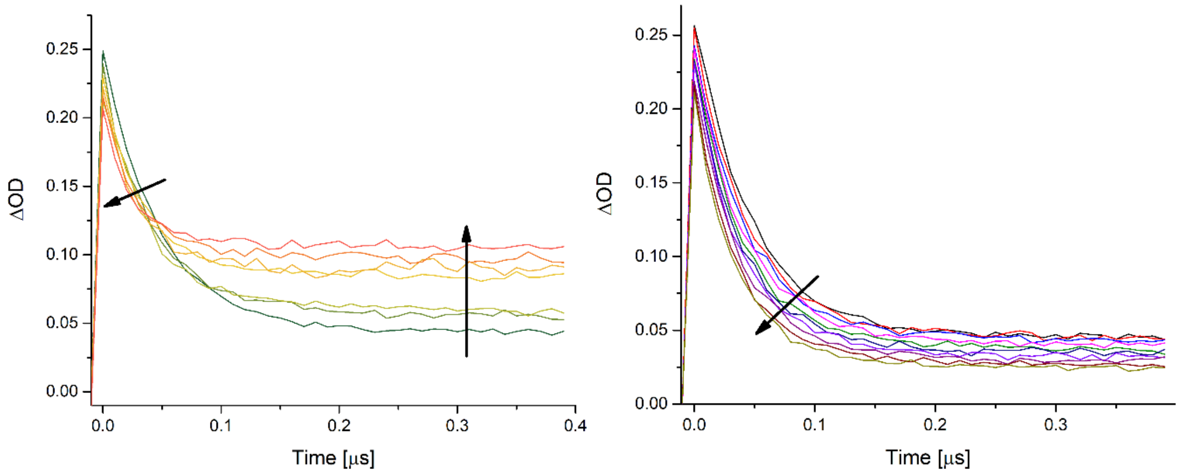

Figure S3. Decay profiles of a CH_3CN solution of TBADT (10^{-4} M) upon addition of increasing amounts of **1a** (left) and **2a** (right).

From decays reported in Figure S3, it is possible to derive the Stern-Volmer plots shown in Figure S4 following the equation:

$$\frac{\tau_0}{\tau} = 1 + \overbrace{k_Q \cdot \tau_0}^{b} \cdot [Q]$$

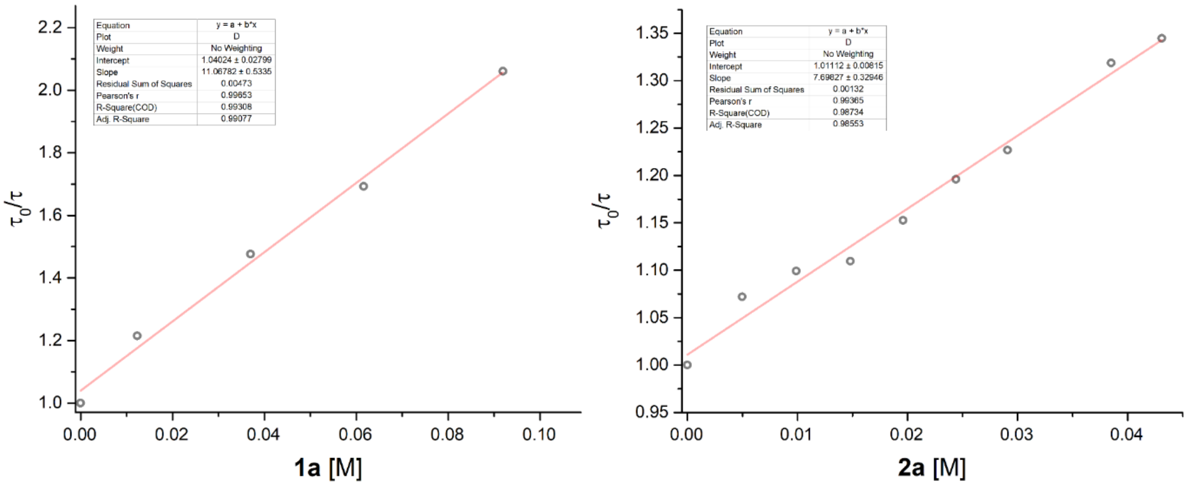

Figure S4. Stern-Volmer plots derived from Figure S3 **1a** (left) and **2a** (right).

b = $k_Q \times \tau_0$ $\tau_0 = 47.5 \times 10^{-9}$ s

k_Q(**1a**) = 2.3×10^8 $M^{-1} \cdot s^{-1}$
k_Q(**2a**) = 1.6×10^8 $M^{-1} \cdot s^{-1}$

3.5 Quantum yield measurement

The Quantum yield (QY) was determined via ferrioxalate actinometry according to a procedure reported in the literature.[7]

Synthesis of the actinometer. Potassium ferrioxalate was prepared according to a procedure reported in the literature.[8] In particular, 3.2 g of $FeCl_3$ was dissolved in 8 mL of distilled H_2O and was added to a hot solution of 12 g of $K_2C_2O_4$ in 20 mL of distilled H_2O. After a couple of minutes at 100 °C, the mixture was cooled down at room temperature and crystallization was triggered with a glass stick. After crystallization was complete, the mother liquor was removed via a Pasteur pipette and the green crystals ($K_3[Fe(C_2O_4)_3]$) were dissolved again in 20 mL of distilled H_2O. Potassium ferrioxalate was recrystallized two more times, washed with MeOH and dried at 45°C for 1 hour, light green crystals (5 g, 55%) were obtained.

Actinometric measurement. As for the actinometry, two solutions were prepared (It should be noted that the following steps were carried out in the dark, with a deep red lamp):

a) Ferrioxalate solution: 52 mg in 10 mL of H_2SO_4 0.05 M (0.012 M). For this concentration, the fraction of absorbed photons is >99% and decomposition QY was reported to be ~1.2.[9]

b) *o*-phenantroline solution: 10 mg of o-phenanthroline (0.0055 M), 2.25 g of sodium acetate trihydrate (0.016 M) in 10 mL of H_2SO_4 0.5 M.

So, 1 mL of solution a) was transferred into a vial. This operation was repeated three times to get 3 identical solutions: one of them was kept in the dark (Blank), while the other two (Test 1 and Test 2) were irradiated with the same setup adopted for batch experiments (Figure S1) for 60 seconds. After irradiation, 20 μL of each of the three solutions were withdrawn and diluted with 2 mL (1:100 dilution) of solution b) each. The obtained solutions were further diluted 1:1.33 with distilled water. The total dilution factor was 1:133.

Then, a single-beam spectrophotometer was used to read the difference of absorbance (ΔA) between Test 1 and 2 and the Blank.

for Test 1: ΔA = 0.15796

for Test 2: ΔA = 0.14166

We calculated the quantum yield after 2.5 hours of reaction, when 4.1×10^{-5} moles (20% yield) of product **3** were formed (Figure S5). QY was calculated from the ratio between the moles of product produced after 2.5 hours and the moles of photon reaching the reaction vial in 2.5 hours.

	**[Fe^{II}]**	**mol Fe^{II}**	**E/s**	**E/h**	**E in 2.5 h**	**QY**
**Test 1**	1.89×10^{-3} M	1.89×10^{-6}	2.63×10^{-8}	9.46×10^{-5}	2.37×10^{-4}	18%
**Test 2**	1.69×10^{-3} M	1.69×10^{-6}	2.36×10^{-8}	8.49×10^{-5}	2.12×10^{-4}	19%

[Fe^{II}]: concentration of iron(II) produced in the irradiated solutions.

mol Fe^{II}: moles of iron(II) produced in the irradiated solutions.

E/s: Einstein per second calculated as mol Fe^{II} divided by QY and irradiation time in seconds.

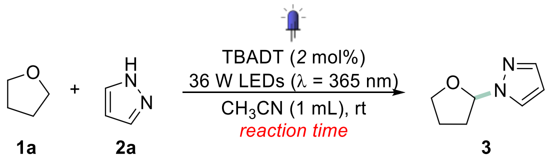

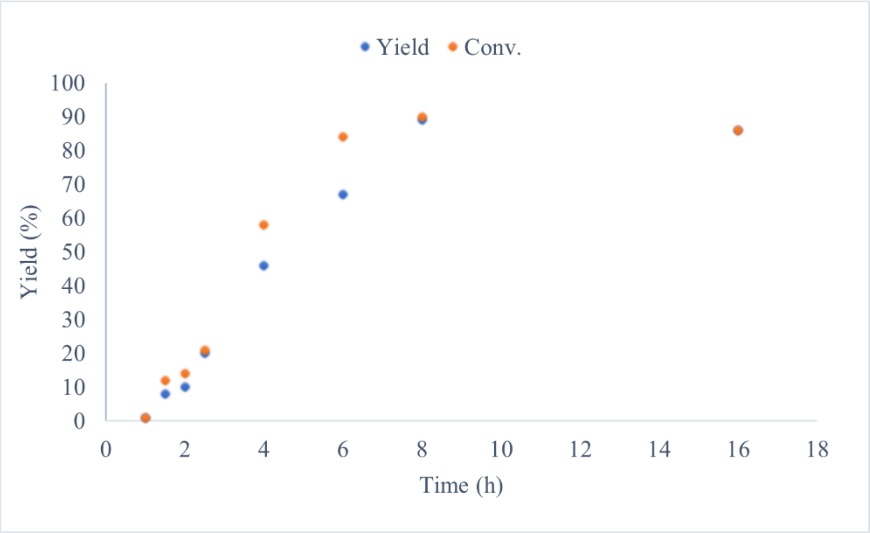

Figure S5. Kinetic profile of reaction.

QY was determined to be 18% (as average of two experiments), indicating that the process is most likely not a radical-chain reaction.

5. Limitation of the scope

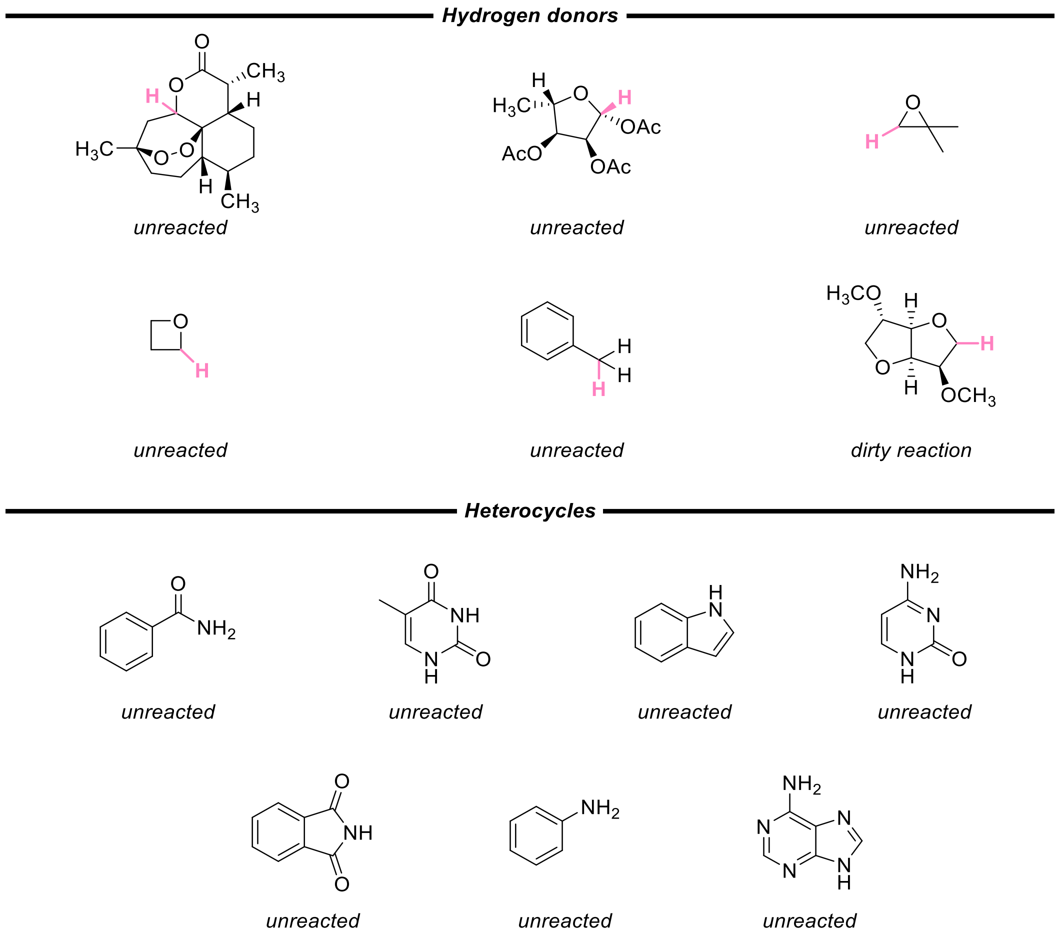

6. General procedure

General Procedure 1 (GP1), batch conditions:

A stock solution containing TBADT (5 mol%, 166 mg), azole (1 mmol), dry acetonitrile (5 mL), TBHP (545 µL, 5.5 M in decane or nonane, 3 equiv) and ether was prepared in a vial equipped with cap with septum and stirring bar (see each case for details). The solution was then split into 5 vials (1 mL each) and individual solutions were sparged with nitrogen. The reaction was stirred and irradiated with 36 W UV-A LEDs for 16 h. The solutions were collected, solvent was removed under reduced pressure and the crude was purified via column chromatography on silica gel to provide the product.

General Procedure 2 (GP2), continuous-flow conditions:

To an oven-dried tube was added TBADT (5 mol%, 166 mg), azole (1 mmol), dry acetonitrile (5 mL), TBHP (5.5 M in decane, 3 equiv, 545 µL), ether (18 equiv). The mixture was swirled until homogenous, taken with a 10 mL disposable syringe and mounted on a syringe pump. The flow rate was set to 0.167 mL min^{-1}, corresponding to a residence time of 1 h; *flow setup 2* was used. When the syringe was fully empty, again acetonitrile was loaded into a syringe and injected to collect all product at the end of the reactor in a flask. The solvent was removed under reduced pressure and purified via column chromatography on silica gel to provide the product.

7. Characterization data

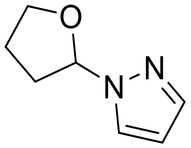

1-(Tetrahydrofuran-2-yl)-1H-pyrazole (3). Prepared according to GP2. R_f=0.3 (Cyclohexane:Ethyl acetate 6:1); purified by flash column chromatography on silica gel (Cyclohexane:Ethyl Acetate 98:2) to afford the product as light yellow oil (112 mg, 81%). Reaction performed on 10 mmol scale: 80%. ^{1}H NMR (400 MHz, $CDCl_3$) δ 7.56 – 7.52 (m, 2H), 6.25 (t, J = 2 Hz, 1H), 5.98 (dd, J_1 = 7 Hz, J_2 = 3 Hz, 1H), 4.10 (td, J_1 = 8 Hz, J_2 = 6 Hz, 1H), 4.00 – 3.93 (m, 1H), 2.63 – 2.55 (m, 1H), 2.36 – 2.26 (m, 1H), 2.23 – 2.11 (m, 1H), 2.07 – 1.96 (m, 1H); ^{13}C NMR (100 MHz, $CDCl_3$) δ 140.0, 128.1, 105.7, 90.1, 69.3, 31.8, 24.5; HRMS (ESI) *m/z* calcd for $C_7H_{11}N_2O^+$: 139.0866 $[M+H]^+$; found: 139.0871. Spectroscopic data are in accordance with the literature.[10]

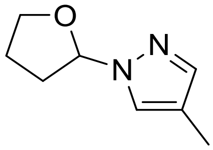

4-Methyl-1-(tetrahydrofuran-2-yl)-1H-pyrazole (4). Prepared according to GP2. R_f=0.5 (Cyclohexane:Ethyl acetate 3:1); purified by flash column chromatography on silica gel (Cyclohexane:Ethyl Acetate 96:4) to afford the product as colorless oil (144 mg, 94%). ^{1}H NMR (400 MHz, $CDCl_3$) δ 7.32 (s, 1H), 7.29 (s, 1H), 5.88 (dd, J_1 = 7 Hz, J_2 = 3 Hz, 1H), 4.09 – 4.02 (m, 1H), 3.96 – 3.89 (m, 1H), 2.59 – 2.50 (m, 1H), 2.32 – 2.11 (m, 2H), 2.04 (s, 3H), 2.02 – 1.94 (m, 1H); ^{13}C NMR (100 MHz, $CDCl_3$) δ 140.5, 127.0, 116.4, 89.9, 69.1, 31.5, 24.7, 9.0; HRMS (ESI) *m/z* calcd for $C_8H_{13}N_2O^+$: 153.1022 $[M+H]^+$; found: 153.1028. Spectroscopic data are in accordance with the literature.[11]

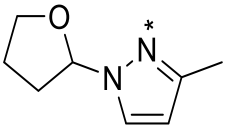

3-Methyl-1-(tetrahydrofuran-2-yl)-1H-pyrazole (5) 5-methyl-1-(tetrahydrofuran-2-yl)-1H-pyrazole (5′). Prepared according to GP2. Purified by flash column chromatography on silica gel (Cyclohexane: Ethyl Acetate 98:2) to afford the product **5** (39.3 mg, 26%) and **5′** (27.4 mg, 18%) as two colorless oils. Ratio **5**:**5′** = 3:2.

Characterization of **5**

Major: R_f=0.5 (Cyclohexane:Ethyl acetate 5:1). ^{1}H NMR (400 MHz, $CDCl_3$) δ 7.42 (d, J = 2 Hz, 1H), 6.03 (d, J = 2 Hz, 1H), 5.91 (dd, J_1 = 7 Hz, J_2 = 3 Hz, 1H), 4.14 – 4.06 (m, 1H), 4.99 – 3.91 (m, 1H), 2.59 – 2.50 (m, 1H), 2.35 – 2.23 (m, 4H), 2.21 – 2.10 (m, 1H), 2.04 – 1.95 (m, 1H); ^{13}C NMR (100 MHz, $CDCl_3$) δ 149.4, 128.8, 105.5, 90.0, 69.1, 31.8, 24.7, 13.8; HRMS (ESI) *m/z* calcd for $C_8H_{13}N_2O^+$: 153.1022 $[M+H]^+$; found: 153.1028. Spectroscopic data are in accordance with the literature.[10]

Characterization of **5′**

Minor: R_f=0.4 (Cyclohexane:Ethyl acetate 5:1). ^{1}H NMR (400 MHz, $CDCl_3$) δ 7.41 (d, *J* = 2 Hz, 1H), 6.04 – 5.99 (m, 1H), 5.96 (dd, J_1 = 7 Hz, J_2 = 3 Hz, 1H), 4.03 (q, *J* = 7 Hz, 1H), 3.92 (td, J_1 = 8 Hz, J_2 = 6 Hz, 1H), 2.87 – 2.78 (m, 1H), 2.43 – 2.22 (m, 5H), 2.09 – 1.97 (m, 1H); ^{13}C NMR (100 MHz, $CDCl_3$) δ 139.3, 138.9, 106.2, 86.2, 68.8, 30.3, 25.3, 11.2; HRMS (ESI) *m/z* calcd for $C_8H_{13}N_2O^+$: 153.1022 [M+H]$^+$; found: 153.1028.

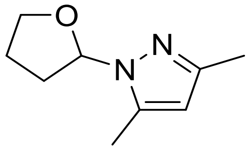

3,5-Dimethyl-1-(tetrahydrofuran-2-yl)-1H-pyrazole (6). Prepared according to GP2. R_f=0.3 (Cyclohexane:Ethyl Acetate 10:1); purified by flash column chromatography on silica gel (Cyclohexane:Ethyl Acetate 98:2) to afford the product as colorless oil (80 mg, 48%), as well as 45 mg (48%) of the starting azole. ^{1}H NMR (400 MHz, $CDCl_3$) δ 5.87 (dd, J_1 = 7 Hz, J_2 = 4 Hz, 1H), 5.81 (s, 1H), 4.05 (q, *J* = 7 Hz, 1H), 3.89 (td, J_1 = 8 Hz, J_2 = 6 Hz, 1H), 2.84-2.74 (m, 1H), 2.41-2.28 (m, 4H), 2.27-2.19 (m, 4H), 2.05-1.95 (m, 1H); ^{13}C NMR (100 MHz, $CDCl_3$) δ 148.2, 140.1, 106.3, 86.0, 68.7, 30.3, 25.6, 13.9, 11.1; HRMS (FI) *m/z* calcd for $C_9H_{14}N_2O$: 166.1106; found: 166.1124. Spectroscopic data are in accordance with the literature.[11]

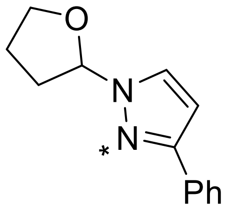

3-Phenyl-1-(tetrahydrofuran-2-yl)-1H-pyrazole (7) and 5-phenyl-1-(tetrahydrofuran-2-yl)-1H-pyrazole (7′). Prepared according to GP2. Purified by flash column chromatography on silica gel (Cyclohexane:Ethyl Acetate 98:2) to afford the product **7** (75.9 mg, 35%) and **7′** (70.6 mg, 33%) as two colorless oils. Ratio **7**:**7′** = 1:1.

Characterization of **7**

R_f=0.8 (Cyclohexane:Ethyl acetate 3:1); ^{1}H NMR (400 MHz, $CDCl_3$) δ 7.87 – 7.79 (m, 2H), 7.58 (d, *J* = 2 Hz, 1H), 7.43 – 7.36 (m, 2H), 7.34 – 7.27 (m, 1H), 6.57 (d, *J* = 2 Hz, 1H), 6.03 (dd, J_1 = 7 Hz, J_2 = 3 Hz, 1H), 4.22 – 4.15 (m, 1H), 4.01 (q, *J* = 7 Hz, 1H), 2.72 – 2.63 (m, 1H), 2.40 – 2.28 (m, 1H), 2.29 – 2.16 (m, 1H), 2.11 – 1.99 (m, 1H); ^{13}C NMR (100 MHz, $CDCl_3$) δ 152.0, 133.7, 129.3, 128.7, 127.7, 125.8, 103.0, 90.4, 69.4, 32.0, 24.5; HRMS (ESI) *m/z* calcd for $C_{13}H_{15}N_2O^+$: 215.1179 [M+H]$^+$; found: 215.1184. Spectroscopic data are in accordance with the literature.[10]

Characterization of **7′**

R_f=0.9 (Cyclohexane:Ethyl acetate 3:1). ^{1}H NMR (400 MHz, $CDCl_3$) δ 7.61 – 7.50 (m, 3H), 7.50 – 7.36 (m, 3H), 6.32 (d, *J* = 2 Hz, 1H), 5.97 (dd, J_1 = 7 Hz, J_2 = 3 Hz, 1H), 4.21 (q, *J* = 8 Hz, 1H), 4.01 – 3.92 (m, 1H), 2.83 – 2.71 (m, 1H), 2.56 – 2.41 (m, 1H), 2.28 – 2.17 (m, 1H), 2.10 – 1.96 (m, 1H); ^{13}C NMR (100 MHz, $CDCl_3$) δ 144.5, 139.4, 130.6, 129.4, 128.7, 128.6, 106.6, 86.4, 69.1, 30.9, 25.6; HRMS (ESI) *m/z* calcd for $C_{13}H_{15}N_2O^+$: 215.1179 [M+H]$^+$; found: 215.1184.

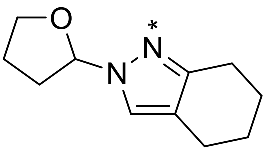

2-(Tetrahydrofuran-2-yl)-4,5,6,7-tetrahydro-2H-indazole (8) and 1-(tetrahydrofuran-2-yl)-4,5,6,7-tetrahydro-1H-indazole (8′). Prepared according to GP2. Purified by flash column chromatography on silica gel (Cyclohexane:Ethyl Acetate 98:2) to afford the product **8** (59.0 mg, 31%) and **8′** (58.2 mg, 30%) as two colorless oils. Ratio **8**:**8′** = 1:1.

Characterization of **8**

R_f=0.4 (Cyclohexane:Ethyl acetate 3:1). ^{1}H NMR (400 MHz, $CDCl_3$) δ 7.23 (s, 1H), 5.89 (dd, J_1 = 7 Hz, J_2 = 3 Hz, 1H), 4.12 – 4.05 (m, 1H), 3.94 (td, J_1 = 8 Hz, J_2 = 6 Hz, 1H), 2.67 (t, J = 6 Hz, 2H), 2.63 – 2.48 (m, 3H), 2.36 – 2.11 (m, 2H), 2.07 – 1.93 (m, 1H), 1.83 – 1.76 (m, 2H), 1.75 – 1.68 (m, 2H); ^{13}C NMR (100 MHz, $CDCl_3$) δ 150.0, 125.4, 116.3, 90.0, 69.0, 31.5, 24.9, 23.7, 23.7, 23.6, 20.8; HRMS (ESI) *m/z* calcd for $C_{11}H_{17}N_2O^+$: 193.1335 [M+H]$^+$; found: 193.1330.

Characterization of **8′**

R_f=0.5 (Cyclohexane:Ethyl acetate 3:1). ^{1}H NMR (400 MHz, $CDCl_3$) δ 7.29 (s, 1H), 5.88 (dd, J_1 = 7 Hz, J_2 = 3 Hz, 1H), 4.07 – 3.99 (m, 1H), 3.91 (td, J_1 = 8 Hz, J_2 = 5 Hz, 1H), 2.84 – 2.69 (m, 2H), 2.67 – 2.58 (m, 1H), 2.51 – 2.45 (m, 2H), 2.40 – 2.21 (m, 2H), 2.07 – 1.96 (m, 1H), 1.86 – 1.78 (m, 2H), 1.75 – 1.68 (m, 2H); ^{13}C NMR (100 MHz, $CDCl_3$) δ 138.9, 137.7, 116.8, 86.1, 68.7, 30.1, 25.4, 23.1, 22.8, 21.4, 20.7; HRMS (ESI) *m/z* calcd for $C_{11}H_{17}N_2O^+$: 193.1335 [M+H]$^+$; found: 193.1336.

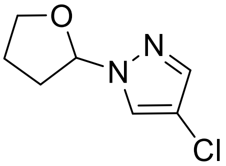

4-Chloro-1-(tetrahydrofuran-2-yl)-1H-pyrazole (9). Prepared according to GP2. R_f=0.5 (Cyclohexane:Ethyl acetate 5:1); purified by flash column chromatography on silica gel (Cyclohexane:Ethyl Acetate 98:2) to afford the product as colorless oil (142 mg, 83%). ^{1}H NMR (400 MHz, $CDCl_3$) δ 7.52 (s, 1H), 7.43 (s, 1H), 5.88 (dd, J_1 = 7 Hz, J_2 = 3 Hz, 1H), 4.10 – 4.03 (m, 1H), 3.99 – 3.91 (m, 1H), 2.58 – 2.49 (m, 1H), 2.33 – 2.22 (m, 1H), 2.15 – 1.94 (m, 2H); ^{13}C NMR (100 MHz, $CDCl_3$) δ 138.2, 126.0, 110.3, 90.7, 69.4, 31.6, 24.2; HRMS (FI) *m/z* calcd for $C_7H_9ClN_2O$: 172.0403; found: 172.0405. Spectroscopic data are in accordance with the literature.[12]

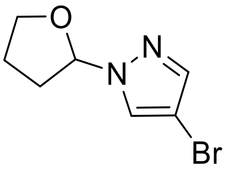

4-Bromo-1-(tetrahydrofuran-2-yl)-1H-pyrazole (10). Prepared according to GP2. R_f=0.5 (Cyclohexane:Ethyl acetate 5:1); purified by flash column chromatography on silica gel (Cyclohexane:Ethyl Acetate 98:2) to afford the product as colorless oil (147 mg, 68%). ^{1}H NMR (400 MHz, $CDCl_3$) δ 7.57 (d, J = 1 Hz, 1H), 7.47 (s, 1H), 5.92 (dd, J_1 = 7 Hz, J_2 = 3 Hz, 1H), 4.12 – 4.05 (m, 1H), 4.01 – 3.93 (m, 1H), 2.60 – 2.51 (m, 1H), 2.35 – 2.24 (m, 1H), 2.17 – 1.96 (m, 2H); ^{13}C NMR (101 MHz, $CDCl_3$) δ 140.3, 128.2, 93.5,

90.7, 69.5, 31.8, 24.2; HRMS (FI) *m/z* calcd for $C_7H_9BrN_2O$: 215.9898; found: 215.9895. Spectroscopic data are in accordance with the literature.[11]

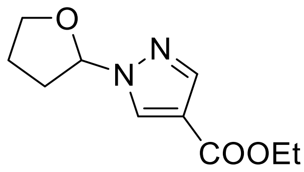

Ethyl 1-(tetrahydrofuran-2-yl)-1H-pyrazole-4-carboxylate (11). Prepared according to GP2. R_f=0.3 (Cyclohexane:Ethyl acetate 5:1); purified by flash column chromatography on silica gel (Cyclohexane : Ethyl Acetate 98:2) to afford the product as colorless oil (168 mg, 80%). ^{1}H NMR (400 MHz, $CDCl_3$) δ 8.02 (s, 1H), 7.89 (s, 1H), 5.94 (dd, J_1 = 7 Hz, J_2 = 2 Hz, 1H), 4.25 (q, J = 7 Hz, 2H), 4.13 (td, J_1 = 8 Hz, J_2 = 5 Hz, 1H), 3.97 (q, J = 8 Hz, 1H), 2.56 – 2.47 (m, 1H), 2.36 – 2.24 (m, 1H), 2.11 – 1.97 (m, 2H), 1.30 (t, J = 7 Hz, 3H); ^{13}C NMR (100 MHz, $CDCl_3$) δ 163.1, 141.4, 131.2, 115.1, 90.7, 69.7, 60.2, 32.2, 24.0, 14.4; HRMS (FI) *m/z* calcd for $C_{10}H_{14}N_2O_3$: 210.1004; found: 210.0993. Spectroscopic data are in accordance with the literature.[11]

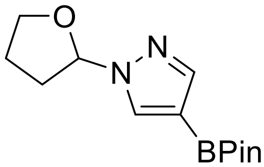

1-(Tetrahydrofuran-2-yl)-4-(4,4,5,5-tetramethyl-1,3,2-dioxaborolan-2-yl)-1H-pyrazole (12). Prepared according to GP2. R_f=0.5 (Cyclohexane:Ethyl acetate 3:1); purified by flash column chromatography on silica gel (Cyclohexane:Ethyl Acetate 93:7) to afford the product as colorless oil (214 mg, 81%); ^{1}H NMR (400 MHz, $CDCl_3$) δ 7.79 (s, 1H), 7.73 (s, 1H), 5.92 (dd, J_1 = 7 Hz, J_2 = 3 Hz, 1H), 4.05 (td, J_1 = 8 Hz, J_2 = 6 Hz, 1H), 3.89 (q, J = 7 Hz, 1H), 2.50 – 2.41 (m, 1H), 2.29 – 2.18 (m, 1H), 2.12 – 2.01 (m, 1H), 1.98 – 1.87 (m, 1H), 1.23 (s, 12H); ^{13}C NMR (100 MHz, $CDCl_3$) δ 145.9, 134.9, 90.1, 83.3, 69.3, 32.0, 24.8, 24.8, 24.2. Note that the carbon atom bearing the Bpin group cannot be seen in the ^{13}C-NMR; ^{11}B NMR (128 MHz, $CDCl_3$) δ 29.85; HRMS (ESI) *m/z* calcd for $C_{13}H_{22}BN_2O_3^+$: 265.1718 [M+H]$^+$; found: 265.1726. Spectroscopic data are in accordance with the literature.[11]

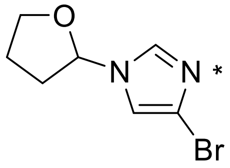

4-Bromo-1-(tetrahydrofuran-2-yl)-1H-imidazole and 5-bromo-1-(tetrahydrofuran-2-yl)-1H-imidazole (13+13′). Prepared according to GP2. R_f=0.2 (Cyclohexane:Ethyl acetate 3:1); purified by flash column chromatography on silica gel (Cyclohexane:Ethyl Acetate 85:25) to afford a mixture of two isomers as colorless oil (165 mg, 76%). Ratio = 3:1. ^{1}H NMR (400 MHz, $CDCl_3$) δ 7.61 (s, $1H_{minor}$), 7.43 (d, J = 1 Hz, $1H_{major}$), 6.96 – 6.95 (m, $1H_{minor}$), 6.91 (d, J = 1 Hz, $1H_{major}$), 5.89 (dd, J_1 = 7 Hz, J_2 = 3 Hz, $1H_{minor}$), 5.79 (dd, J_1 = 7 Hz, J_2 = 3 Hz, $1H_{major}$), 4.18 – 4.11 (m, $1H_{minor}$), 4.04 (td, J_1 = 8 Hz, J_2 = 6 Hz, $1H_{major}$), 4.00 – 3.87 (m, 2H), 2.41 – 2.27 (m, 2H), 2.22 – 2.11 (m, 2H), 2.10 – 1.93 (m, 4H); ^{13}C NMR (100 MHz, $CDCl_3$) δ 135.4, 134.9, 130.0 (2C), 115.8, 115.6, 87.1 (2C), 69.5, 69.5, 33.1, 33.03, 23.95, 23.79; HRMS (FI) *m/z*

calcd for $C_7H_9BrN_2O$: 215.9898; found: 215.9886.

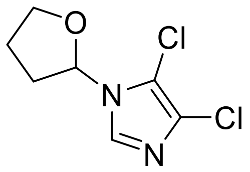

4,5-Dichloro-1-(tetrahydrofuran-2-yl)-1H-imidazole (14). Prepared according to GP2. R_f=0.3 (Cyclohexane:Ethyl acetate 3:1); purified by flash column chromatography on silica gel (Cyclohexane:Ethyl Acetate 92:8) to afford the product as colorless oil (87 mg, 42%). ^{1}H NMR (400 MHz, $CDCl_3$) δ 7.46 (s, 1H), 5.87 (dd, J_1 = 6 Hz, J_2 = 3 Hz, 1H), 4.21 – 4.14 (m, 1H), 4.03 –3.95 (m, 1H), 2.44 – 2.33 (m, 1H), 2.27 – 2.18 (m, 1H), 2.13 – 1.97 (m, 2H); ^{13}C NMR (100 MHz, $CDCl_3$) δ 131.9, 126.9, 111.4, 87.3, 69.9, 32.9, 23.7; HRMS (FI) *m/z* calcd for $C_7H_8Cl_2N_2O$: 206.0014; found: 206.0007.

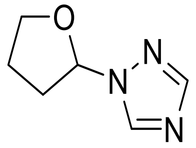

1-(Tetrahydrofuran-2-l)-1H-1,2,4-triazole (15). Prepared according to GP1. R_f=0.2 (Cyclohexane:Ethyl acetate 1:1); purified by flash column chromatography on silica gel (Cyclohexane:Ethyl Acetate 87:13) to afford the product as colorless oil (133 mg, 96%). ^{1}H NMR (400 MHz, $CDCl_3$) δ 8.17 (s, 1H), 7.88 (s, 1H), 6.00 (dd, J_1 = 7 Hz, J_2 = 2 Hz, 1H), 4.13 – 4.06 (m, 1H), 3.96 (q, J = 7 Hz, 1H), 2.52 – 2.44 (m, 1H), 2.34 – 2.25 (m, 1H), 2.12 – 1.95 (m, 2H); ^{13}C NMR (100 MHz, $CDCl_3$) δ 152.1, 142.2, 88.8, 69.7, 32.1, 23.9; HRMS (FI) *m/z* calcd for $C_6H_9N_3O$: 139.0746 ; found: 139.0744. Spectroscopic data are in accordance with the literature.[11]

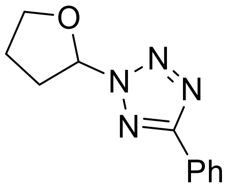

5-Phenyl-2-(tetrahydrofuran-2-yl)-2H-tetrazole (16). Prepared according to GP2 (CH_3CN 12 mL). R_f=0.5 (Cyclohexane:Ethyl acetate 5:1); purified by flash column chromatography on silica gel (Cyclohexane:Ethyl Acetate 98:2) to afford the product as colorless oil (100 mg, 46%). ^{1}H NMR (400 MHz, $CDCl_3$) δ 8.18 – 8.14 (m, 2H), 7.52 – 7.44 (m, 3H), 6.58 (dd, J_1 = 6 Hz, J_2 = 2 Hz, 1H), 4.31 – 4.24 (m, 1H), 4.17 – 4.10 (m, 1H), 2.75 – 2.65 (m, 1H), 2.57 – 2.44 (m, 2H), 2.22 – 2.12 (m, 1H); ^{13}C NMR (100 MHz, $CDCl_3$) δ 165.3, 130.5, 129.0 (2C), 127.5, 127.1 (2C), 91.8, 70.4, 32.0, 24.2; HRMS (FI) *m/z* calcd for $C_{11}H_{12}N_4O$: 216.1011; found: 216.0998. Spectroscopic data are in accordance with the literature.[11]

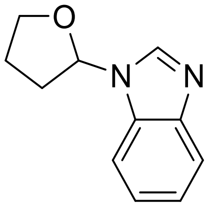

1-(Tetrahydrofuran-2-yl)-1H-benzo[d]imidazole (17). Prepared according to GP2. R_f=0.2 (CH_2Cl_2:CH_3OH 20:1); purified by flash column chromatography on silica gel (CH_2Cl_2:CH_3OH 95:5) to afford the product as colorless oil (103 mg, 55%). ^{1}H NMR (400 MHz, $CDCl_3$) δ 7.93 (s, 1H), 7.77 – 7.67 (m, 1H), 7.42 – 7.33 (m, 1H), 7.26 – 7.16 (m, 2H), 6.08 (dd, J_1 = 6 Hz, J_2 = 4 Hz, 1H), 4.12 – 4.05 (m, 1H), 4.01 – 3.89 (m, 1H), 2.39 – 2.29 (m, 2H), 2.09 – 1.99 (m, 2H); ^{13}C NMR (100 MHz, $CDCl_3$) δ 144.3, 140.3, 132.6, 123.1, 122.5, 120.4, 110.5, 86.1, 69.0, 31.9, 24.3; HRMS (FI) *m/z* calcd for $C_{11}H_{12}N_2O$: 188.0950; found: 188.0957. Spectroscopic data are in accordance with the literature.[13]

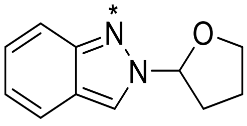

2-(tetrahydrofuran-2-yl)-2H-indazole (18) and 1-(Tetrahydrofuran-2-yl)-1H-indazole (18′). Prepared according to GP2. Purified by flash column chromatography on silica gel (Cyclohexane:Ethyl Acetate 98:2) to afford the product **18** (80.3 mg, 43%) and **18′** (6.7 mg, 3%) as two colorless oils. Ratio **18**:**18′** = 12:1.

Characterization of **18**

Major: R_f=0.5 (Cyclohexane:Ethyl acetate 3:1); ^{1}H NMR (400 MHz, $CDCl_3$) δ 8.01 (s, 1H), 7.66 – 7.60 (m, 1H), 7.60 – 7.54 (m, 1H), 7.19 (ddd, J_1 = 8 Hz, J_2 = 7 Hz, J_3 = 1 Hz, 1H), 7.02 – 6.45 (m, 1H), 6.18 (dd, J_1 = 7 Hz, J_2 = 2 Hz, 1H), 4.17 (td, J_1 = 8 Hz, J_2 = 5 Hz, 1H), 3.98 (q, J = 8 Hz, 1H), 2.67 – 2.58 (m, 1H), 2.35 (dtd, J_1 = 13 Hz, J_2 = 9 Hz, J_3 = 7 Hz, 1H), 2.06 – 1.90 (m, 2H); ^{13}C NMR (100 MHz, $CDCl_3$) δ 149.1, 126.2, 121.7, 121.4, 121.1, 120.6, 117.8, 91.7, 70.0, 33.0, 24.0; HRMS (FI) *m/z* calcd for $C_{11}H_{12}N_2O$: 188.0950; found: 188.0951.

Characterization of **18′**

Minor: R_f=0.3 (Cyclohexane:Ethyl acetate 3:1); ^{1}H NMR (400 MHz, $CDCl_3$) δ 8.02 (s, 1H), 7.72 (dt, J_1 = 8 Hz, J_2 = 1 Hz, 1H), 7.63 – 7.58 (m, 1H), 7.43 – 7.37 (m, 1H), 7.20 – 7.14 (m, 1H), 6.40 (dd, J_1 = 7 Hz, J_2 = 3 Hz, 1H), 4.10 – 3.95 (m, 2H), 3.00 – 2.89 (m, 1H), 2.49 – 2.34 (m, 2H), 2.18 – 2.05 (m, 1H); ^{13}C NMR (100 MHz, $CDCl_3$) δ 140.0, 134.1, 126.7, 124.8, 121.3, 121.1, 109.8, 87.0, 68.9, 30.4, 25.2; HRMS (FI) *m/z* calcd for $C_{11}H_{12}N_2O$: 188.0950; found: 188.0958. Spectroscopic data are in accordance with the literature.[14]

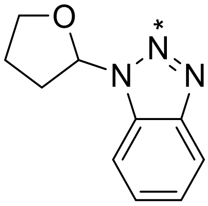

1-(Tetrahydrofuran-2-yl)-1H-benzo[d][1,2,3]triazole (19) and 2-(tetrahydrofuran-2-yl)-2H-benzo[d][1,2,3]triazole (19′). Prepared according to GP2. Purified by flash column chromatography on

silica gel (Cyclohexane:Ethyl Acetate 96:4) to afford the product **19** (52.9 mg, 28%) and **19′** (26.5 mg, 14%) as two colorless oils. Ratio **19**:**19′** = 2:1.

Characterization of **19**

Major: R_f=0.4 (Cyclohexane:Ethyl acetate 3:1). ^{1}H NMR (400 MHz, $CDCl_3$) δ 8.05 (d, *J* = 8 Hz, 1H), 7.70 (d, *J* = 8 Hz, 1H), 7.52 – 7.43 (m, 1H), 7.40 – 7.33 (m, 1H), 6.50 (dd, J_1 = 7 Hz, J_2 = 2 Hz, 1H), 4.14 – 3.97 (m, 2H), 3.19 – 3.09 (m, 1H), 2.57 – 2.30 (m, 2H), 2.23– 2.11 (m, 1H); ^{13}C NMR (100 MHz, $CDCl_3$) δ 146.6, 133.0, 127.6, 124.2, 120.0, 110.5, 88.0, 69.4, 30.9, 24.5; HRMS (FI) *m/z* calcd for $C_{10}H_{11}N_3O$: 189.0902; found: 189.0894. Spectroscopic data are in accordance with the literature.[10]

Characterization of **19′**

Minor: R_f=0.5 (Cyclohexane:Ethyl acetate 3:1); ^{1}H NMR (400 MHz, $CDCl_3$) δ 7.92 – 7.83 (m, 2H), 7.42 – 7.33 (m, 2H), 6.59 (dd, J_1 = 6 Hz, J_2 = 2 Hz, 1H), 4.37 – 4.30 (m, 1H), 4.19 – 4.09 (m, 1H), 2.80 – 2.69 (m, 1H), 2.58 – 2.45 (m, 2H), 2.21 – 2.09 (m, 1H); ^{13}C NMR (100 MHz, $CDCl_3$) δ 144.4, 126.7, 118.6, 94.3, 70.4, 32.5, 24.4; HRMS (FI) *m/z* calcd for $C_{10}H_{11}N_3O$: 189.0902; found: 189.0905. Spectroscopic data are in accordance with the literature.[10]

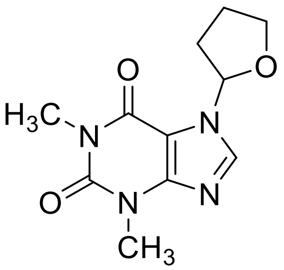

1,3-Dimethyl-7-(tetrahydrofuran-2-yl)-3,7-dihydro-1H-purine-2,6-dione (20). Prepared according to GP1 (**1a**, 18 equiv, $CH_3CN/CHCl_3$ 1:1, 10 mL). R_f=0.2 (Cyclohexane:Ethyl acetate 1:2); m.p. 111-112 °C; purified by flash column chromatography on silica gel (Cyclohexane:Ethyl Acetate 40:60) to afford the product as yellow solid (150 mg, 60%). ^{1}H NMR (400 MHz, $CDCl_3$) δ 7.65 (s, 1H), 6.34 (dd, J_1 = 6 Hz, J_2 = 2 Hz, 1H), 4.22 (td, J_1 = 8 Hz, J_2 = 4 Hz, 1H), 3.98 (td, J_1 = 8 Hz, J_2 = 7 Hz, 1H), 3.50 (s, 3H), 3.31 (s, 3H), 2.49 – 2.37 (m, 1H), 2.26 – 2.17 (m, 1H), 2.06 – 1.96 (m, 1H), 1.94 – 1.82 (m, 1H); ^{13}C NMR (100 MHz, $CDCl_3$) δ 155.0, 151.6, 149.6, 138.3, 105.9, 88.4, 70.3, 34.4, 29.8, 28.0, 23.3; HRMS (ESI) *m/z* calcd for $C_{11}H_{15}N_4O_3^+$: 251.1139 [M+H]$^+$; found: 251.1144.

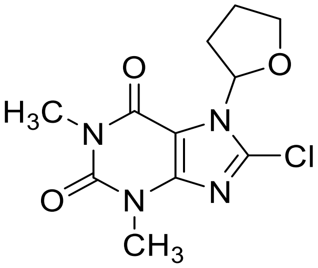

8-Chloro-1,3-dimethyl-7-(tetrahydrofuran-2-yl)-3,7-dihydro-1H-purine-2,6-dione (21). Prepared according to GP1 (**1a**/CH_3CN 1:1, 10 mL). R_f=0.6 (Cyclohexane:Ethyl acetate 1:4); m.p. 245.0 ~ 246.4 °C; purified by flash column chromatography on silica gel (Cyclohexane:Ethyl Acetate 75:25) to afford the product as yellow solid (51 mg, 18%) and starting material **2s** (165 mg, 77%). Yield based on remaining starting material: 78%. ^{1}H NMR (400 MHz, $CDCl_3$) δ 6.54 (t, *J* = 7 Hz, 1H), 4.46 (td, J_1 = 8 Hz, J_2 = 7 Hz, 1H), 4.00 (td, J_1 = 8 Hz, J_2 = 4 Hz, 1H), 3.54 (s, 3H), 3.39 (s, 3H), 2.62 – 2.51 (m, 1H), 2.48 – 2.29 (m, 2H),

2.17 – 2.06 (m, 1H); ^{13}C NMR (100 MHz, $CDCl_3$) δ 154.1, 151.3, 148.0, 138.3, 107.7, 88.3, 70.0, 32.3, 30.1, 28.5, 25.8; HRMS (ESI) *m/z* calcd for $C_{11}H_{14}ClN_4O_3^+$: 285.0749 [M+H]$^+$; found: 285.0754.

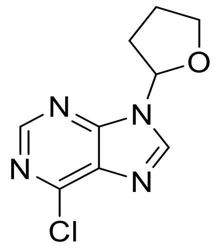

6-Chloro-9-(tetrahydrofuran-2-yl)-9H-purine (22). Prepared according to GP1 (**1a**/CH_3CN 1:1, 10 mL). R_f=0.3 (Cyclohexane:Ethyl acetate 1:2); m.p. 82-83 °C; purified by flash column chromatography on silica gel (Cyclohexane:Ethyl Acetate 35:65) to afford the product as yellow solid (94 mg, 42%). ^{1}H NMR (400 MHz, $CDCl_3$) δ 8.66 (s, 1H), 8.20 (s, 1H), 6.29 (dd, J_1 = 6 Hz, J_2 = 3 Hz, 1H), 4.24 (dt, J_1 = 8 Hz, J_2 = 7 Hz, 1H), 4.03 (q, J = 8 Hz, 1H), 2.60-2.43 (m, 2H), 2.16-2.07 (m, 2H); ^{13}C NMR (100 MHz, $CDCl_3$) δ 151.8, 150.9(2C), 143.4, 132.4, 86.6, 70.0, 32.5, 24.3; HRMS (ESI) *m/z* calcd for $C_9H_{10}ClN_4O^+$: 225.0538 [M+H]$^+$; found: 225.0543. Spectroscopic data are in accordance with the literature.[15]

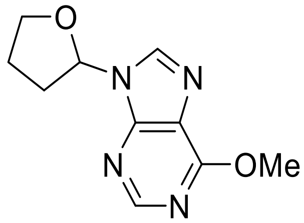

6-Methoxy-9-(tetrahydrofuran-2-yl)-9H-purine (23). Prepared according to GP1 (**1a**/CH_3CN 1:1, 10 mL). R_f=0.2 (Cyclohexane:Ethyl acetate 1:3); purified by flash column chromatography on silica gel (Cyclohexane:Ethyl Acetate 40:60) to afford the product as colorless oil (88 mg, 40%). ^{1}H NMR (400 MHz, $CDCl_3$) δ 8.51 (s, 1H), 8.02 (s, 1H), 6.31 (dd, J_1 = 6 Hz, J_2 = 3 Hz, 1H), 4.26 (ddd, J_1 = 8 Hz, J_2 = 7 Hz, J_3 = 6 Hz, 1H), 4.16 (s, 3H), 4.05 (dt, J_1 = 8 Hz, J_2 = 7 Hz, 1H), 2.60 – 2.43 (m, 2H), 2.19 – 2.09 (m, 2H); ^{13}C NMR (100 MHz, $CDCl_3$) δ 161.1, 152.1, 151.3, 140.3, 122.3, 86.1, 69.8, 54.3, 32.6, 24.4; HRMS (ESI) *m/z* calcd for $C_{10}H_{13}N_4O_2^+$: 221.1033 [M+H]$^+$; found: 221.1030. Spectroscopic data are in accordance with the literature. [16]

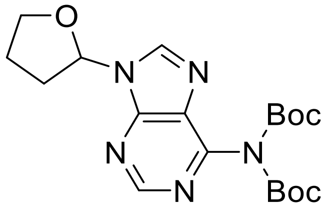

Tert-butyl (tert-butoxycarbonyl)(9-(tetrahydrofuran-2-yl)-9H-purin-6-yl)carbamate (24). Prepared according to GP2. R_f=0.6 (Cyclohexane:Ethyl acetate 1:4); purified by flash column chromatography on silica gel (Cyclohexane:Ethyl Acetate 65:35) to afford the product as colorless oil (97 mg, 24%), and starting material **2v** (93.8 mg, 28%). Yield based on remaining starting material: 33%; ^{1}H NMR (400 MHz, $CDCl_3$) δ 8.79 (s, 1H), 8.15 (s, 1H), 6.30 (dd, J_1 = 6 Hz, J_2 = 3 Hz, 1H), 4.27 – 4.19 (m, 1H), 4.07 – 3.98 (m, 1H), 2.60 – 2.42 (m, 2H), 2.17 – 2.07 (m, 2H), 1.39 (s, 18H); ^{13}C NMR (100 MHz, $CDCl_3$) δ 152.5, 151.9, 150.5,

150.2, 142.9, 129.5, 86.2, 83.7, 69.8, 32.3, 27.8, 24.3; HRMS (ESI) *m/z* calcd for $C_{19}H_{28}N_5O_5^+$: 406.2085 $[M+H]^+$; found: 406.2090. Spectroscopic data are in accordance with the literature. [15]

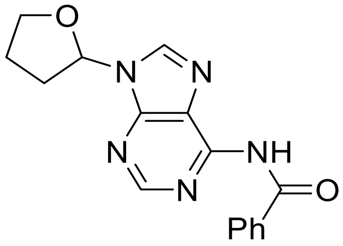

***N*-(9-(tetrahydrofuran-2-yl)-9H-purin-6-yl)benzamide (25).** Prepared according to GP1 (**1a**/CH_3CN 1:1, 10 mL)**.** R_f=0.2 (CH_2Cl_2:MeOH 20:1); purified by flash column chromatography on silica gel (CH_2Cl_2:MeOH 98:2) to afford the product as yellow oil (195 mg, 63%); ^1H NMR (400 MHz, $CDCl_3$) δ 8.74 (s, 1H), 8.08 (s, 1H), 8.04 – 7.97 (m, 2H), 7.56 (td, J_1 = 7 Hz, J_2 = 1 Hz, 1H), 7.51 – 7.43 (m, 2H), 6.32 (dd, J_1 = 6 Hz, J_2 = 3 Hz, 1H), 4.31 – 4.23 (m, 1H), 4.06 (q, J = 7 Hz, 1H), 2.63 – 2.44 (m, 2H), 2.18 – 2.09 (m, 2H); ^{13}C NMR (100 MHz, $CDCl_3$) δ 165.0, 152.5, 151.3, 149.6, 141.2, 133.8, 132.8, 128.9, 128.0, 123.8, 86.3, 69.9, 32.6, 24.3; HRMS (ESI) *m/z* calcd for $C_{16}H_{16}N_5O_2^+$: 310.1299 $[M+H]^+$; found: 310.1304. Spectroscopic data are in accordance with the literature.[17]

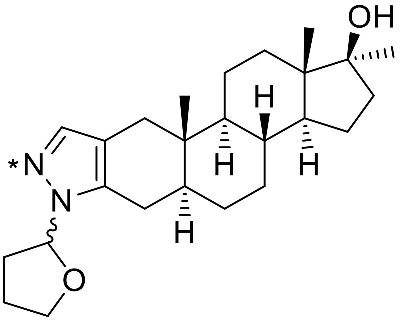

(1S,3aS,3bR,5aS,10aS,10bS,12aS)-1,10a,12a-trimethyl-7-(tetrahydrofuran-2-yl)-1,2,3,3a,3b,4,5,5a,6,7,10,10a,10b,11,12,12a-hexadecahydrocyclopenta[5,6]naphtho[1,2-f]indazol-1-ol (26) and (1S,3aS,3bR,5aS,10aS,10bS,12aS)-1,10a,12a-trimethyl-8-(tetrahydrofuran-2-yl)-1,2,3,3a,3b,4,5,5a,6,8,10,10a,10b,11,12,12a-hexadecahydrocyclopenta[5,6]naphtho[1,2-f]indazol-1-ol (26′). Prepared according to GP2 (CH_3CN/**1a** 2:5,14 mL). Purified by flash column chromatography on silica gel (Cyclohexane:Ethyl Acetate 78:22) to afford the product **26** (62.9 mg, 16%, dr: 1:1) and **26′** (73.6 mg, 18%, dr: 1:1) as two deliquescent solid. Ratio **26**:**26′** = 1:1, and starting material **2x** (199 mg, 61%). Yield based on remaining starting material: 87%.

Characterization of **26**

R_f=0.7 (Cyclohexane:Ethyl acetate 1:1); ^1H NMR (400 MHz, $CDCl_3$) δ 7.27 (d, J = 1 Hz, 1H), 5.87 (dd, J_1 = 7 Hz, J_2 = 4 Hz, 1H), 4.08 – 3.97 (m, 1H), 3.91 (td, J_1 = 8 Hz, J_2 = 5Hz, 1H), 2.84 – 2.75 (m, 1H), 2.73 – 2.50 (m, 2H), 2.42 – 2.19 (m, 3H), 2.13 – 1.96 (m, 2H), 1.87 – 1.69 (m, 3H), 1.67 – 1.50 (m, 5H), 1.46 – 1.14 (m, 10H), 0.96 – 0.77 (m, 5H), 0.74 (d, J = 5 Hz, 3H); ^{13}C NMR (100 MHz, $CDCl_3$) δ 138.2, 138.1, 137.9, 137.8, 116.2, 116.1, 86.4, 86.3, 81.8, 68.8, 68.8, 53.9, 53.9, 50.7, 50.7, 45.5, 42.4, 42.2, 39.1, 39.1, 36.7, 36.6, 36.5, 35.1, 31.8, 31.6, 31.6, 30.2, 30.1, 29.3, 26.2, 26.0, 25.9, 25.5, 25.3, 23.4, 20.9, 14.0, 11.7. HRMS (ESI) *m/z* calcd for $C_{20}H_{39}N_2O_2^+$: 399.3006 $[M+H]^+$; found: 399.3012.

Characterization of **26′**

R_f=0.6 (Cyclohexane:Ethyl acetate 1:1); ^1H NMR (400 MHz, $CDCl_3$) δ 7.21 (d, J = 3 Hz, 1H), 5.88 (d, J =

12 Hz, 1H), 4.12 – 4.05 (m, 1H), 3.97 – 3.90 (m, 1H), 2.65 – 2.49 (m, 3H), 2.34 – 2.22 (m, 2H), 2.22 – 2.13 (m, 1H), 2.11 – 2.05 (m, 1H), 2.03 (s, 1H), 1.85 – 1.68 (m, 3H), 1.65 – 1.49 (m, 5H), 1.44 – 1.19 (m, 9H), 0.94 – 0.74 (m, 8H); ^{13}C NMR (100 MHz, $CDCl_3$) δ 148.9, 126.1, 125.8, 115.9, 115.8, 90.0, 89.9, 81.8, 69.0, 68.9, 54.0, 54.0, 50.7, 45.5, 42.8, 42.7, 39.1, 36.8, 36.4, 35.0, 31.8, 31.6, 31.5, 31.4, 29.5, 27.9, 25.9, 24.9, 23.4, 20.9, 14.0, 11.7, 11.7. HRMS (ESI) *m/z* calcd for $C_{20}H_{39}N_2O_2^+$: 399.3006 [M+H]$^+$; found: 399.3012.

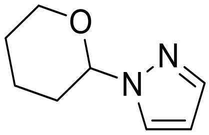

1-(Tetrahydro-2H-pyran-2-yl)-1H-pyrazole (27). Prepared according to GP1**.** (**1b**, 18 equiv, CH_3CN 5 mL). R_f=0.5 (Cyclohexane:Ethyl acetate 10:1); purified by flash column chromatography on silica gel (Cyclohexane:Ethyl Acetate 85:15) to afford the product as colorless oil (115.5 mg, 76%). ^{1}H NMR (400 MHz, $CDCl_3$) δ 7.60 (d, J = 2 Hz, 1H), 7.55 (d, J = 1 Hz, 1H), 6.30 (t, J = 2 Hz, 1H), 5.40 (dd, J_1 = 10 Hz, J_2 = 3 Hz, 1H), 4.09 – 4.02 (m, 1H), 3.74 – 3.65 (m, 1H), 2.19 – 2.00 (m, 3H), 1.76 – 1.57 (m, 3H); ^{13}C NMR (100 MHz, $CDCl_3$) δ 139.7, 127.7, 106.1, 87.7, 67.9, 30.7, 25.1, 22.6; HRMS (FI) *m/z* calcd for $C_8H_{12}N_2O$: 152.0950; found: 152.0956. Spectroscopic data are in accordance with the literature.[18]

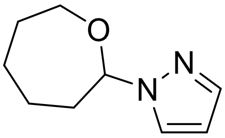

1-(Oxepan-2-yl)-1H-pyrazole (28). Prepared according to GP2. R_f=0.5 (Cyclohexane:Ethyl acetate 10:1); purified by flash column chromatography on silica gel (Cyclohexane:Ethyl Acetate 99:1) to afford the product as colorless oil (94 mg, 56%). ^{1}H NMR (400 MHz, $CDCl_3$) δ 7.59 (d, J = 2 Hz, 1H), 7.55 (d, J = 1 Hz, 1H), 6.29 (t, J = 2 Hz, 1H), 5.66 (dd, J_1 = 10 Hz, J_2 = 5 Hz, 1H), 4.03 – 3.95 (m, 1H), 3.72 (dt, J_1 = 12 Hz, J_2 = 4 Hz, 1H), 2.54 – 2.43 (m, 1H), 2.34 – 2.25 (m, 1H), 1.92 – 1.50 (m, 6H); ^{13}C NMR (100 MHz, $CDCl_3$) δ 139.5, 128.0, 105.8, 89.3, 65.8, 33.8, 31.0, 28.4, 24.1; HRMS (FI) *m/z* calcd for $C_9H_{14}N_2O$: 166.1106; found: 166.1119.

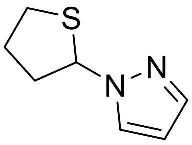

1-(Tetrahydrothiophen-2-yl)-1H-pyrazole (29). Prepared according to GP2. R_f=0.5 (Cyclohexane:Ethyl acetate 10:1); purified by flash column chromatography on silica gel (Cyclohexane:Ethyl Acetate 98:2) to afford the product as colorless oil (148 mg, 96%); ^{1}H NMR (400 MHz, $CDCl_3$) δ 7.70 (d, J = 2 Hz, 1H), 7.53 (d, J = 1 Hz, 1H), 6.23 (t, J = 2 Hz, 1H), 5.99 (dd, J_1 = 6 Hz, J_2 = 3 Hz, 1H), 3.26 – 3.18 (m, 1H), 3.00 – 2.90 (m, 1H), 2.57 – 2.48 (m, 1H), 2.33 – 2.04 (m, 3H); ^{13}C NMR (100 MHz, $CDCl_3$) δ 139.9, 128.2, 105.4, 68.9, 38.8, 33.3, 28.8; HRMS (FI) *m/z* calcd for $C_7H_{10}N_2S$: 154.0565; found: 154.0572. Spectroscopic data are in accordance with the literature.[19]

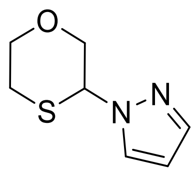

1-(1,4-Oxathian-3-yl)-1H-pyrazole (30). Prepared according to GP2. R_f=0.5 (Cyclohexane:Ethyl acetate 3:1); purified by flash column chromatography on silica gel (Cyclohexane:Ethyl Acetate 95:5) to afford the product as colorless oil (99 mg, 58%); ^{1}H NMR (400 MHz, $CDCl_3$) δ 7.90 (d, *J* = 2 Hz, 1H), 7.56 (d, *J* = 1 Hz, 1H), 6.29 (t, *J* = 2 Hz, 1H), 5.18 (t, *J* = 3 Hz, 1H), 4.47 (dd, J_1 = 12 Hz, J_2 = 4 Hz, 1H), 4.22 (dd, J_1 = 12 Hz, J_2 = 3 Hz, 1H), 4.15 – 4.08 (m, 1H), 3.98 – 3.90 (m, 1H), 2.99 (ddd J_1 = 13 Hz, J_2 = 9 Hz, J_3 = 3 Hz, 1H), 2.53 (ddd, J_1 = 13 Hz, J_2 = 5 Hz, J_3 = 3 Hz, 1H); ^{13}C NMR (100 MHz, $CDCl_3$) δ 139.9, 129.2, 106.1, 71.6, 68.2, 56.3, 25.2; HRMS (ESI) *m/z* calcd for $C_7H_{11}N_2OS^+$: 171.0587 [M+H]$^+$; found: 171.0592. Spectroscopic data are in accordance with the literature.[14]

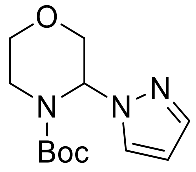

Tert-butyl 3-(1H-pyrazol-1-yl)morpholine-4-carboxylate (31). Prepared according to GP2. (**1f**, 6 equiv). R_f=0.3 (Cyclohexane:Ethyl acetate 6:1); m.p. 86°C; purified by flash column chromatography on silica gel (Cyclohexane:Ethyl Acetate 98:2) to afford the product as white solid (157 mg, 62%). ^{1}H NMR (400 MHz, $CDCl_3$) δ 7.53 (d, *J* = 2 Hz, 1H), 7.48 (d, *J* = 1 Hz, 1H), 6.19 (t, *J* = 2 Hz, 1H), 6.00 (s, 1H), 4.46 (d, *J* = 12 Hz, 1H), 3.91 (dd, J_1 = 11 Hz, J_2 = 3 Hz, 1H), 3.79 (dd, J_1 = 13 Hz, J_2 = 3 Hz, 1H), 3.71 (d, *J* = 13 Hz, 1H), 3.54 (td, J_1 = 12 Hz, J_2 = 3 Hz, 1H), 3.17 (td, J_1 = 13 Hz, J_2 = 4 Hz, 1H), 1.39 (s, 9H); ^{13}C NMR (100 MHz, $CDCl_3$) δ 154.0, 139.3, 128.3, 105.4, 81.2, 67.7, 66.2, 65.0, 39.4, 28.0; HRMS (ESI) *m/z* calcd for $C_{12}H_{20}N_3O_3^+$: 254.1499 [M+H]$^+$; found: 254.1505. Spectroscopic data are in accordance with the literature.[20]

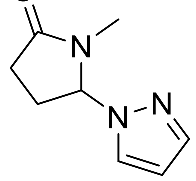

1-Methyl-5-(1H-pyrazol-1-yl)pyrrolidin-2-one (32). Prepared according to GP2 (**1g**, 6 equiv). R_f=0.2 (Cyclohexane:Ethyl acetate 1:3); purified by flash column chromatography on silica gel (Cyclohexane:Ethyl Acetate 50:50) to afford the product as colorless oil (122 mg, 74%). ^{1}H NMR (400 MHz, $CDCl_3$) δ 7.56 (d, *J* = 2 Hz, 1H), 7.46 (d, *J* = 2 Hz, 1H), 6.31(t, *J* = 2 Hz, 1H), 5.72 (dd, J_1 = 8 Hz, J_2 = 3 Hz, 1H), 2.83 – 2.72 (m, 1H), 2.65 (s, 3H), 2.61 – 2.52 (m 1H), 2.50 – 2.42 (m, 1H), 2.35 – 2.27 (m, 1H); ^{13}C NMR (100 MHz, $CDCl_3$) δ 174.8, 140.7, 127.5, 106.7, 76.4, 29.1, 27.4, 25.9; HRMS (FI) *m/z* calcd for $C_8H_{11}N_3O^+$: 165.0902 [M+H]$^+$; found: 165.0898. Spectroscopic data are in accordance with the literature.[21]

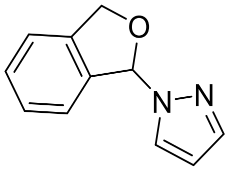

1-(1,3-Dihydroisobenzofuran-1-yl)-1H-pyrazole (33). Prepared according to GP2. R_f=0.5 (Cyclohexane:Ethyl acetate 3:1); purified by flash column chromatography on silica gel (Cyclohexane:Ethyl Acetate 98:2) to afford the product as colorless oil (176 mg, 95%). ^{1}H NMR (400 MHz, $CDCl_3$) δ 7.60 (d, *J* = 2 Hz, 1H), 7.48 – 7.42 (m, 1H), 7.39 – 7.33 (m, 2H), 7.33 – 7.29 (m, 1H), 7.28 (d, *J* = 2 Hz, 1H), 7.20 (d, *J* = 3 Hz, 1H), 6.28 (t, *J* = 2 Hz, 1H), 5.34 (dd, J_1 = 13 Hz, J_2 = 3 Hz, 1H), 5.18 (d, *J* = 13 Hz, 1H); ^{13}C NMR (100 MHz, $CDCl_3$) δ 140.5, 139.8, 136.0, 129.7, 128.2, 127.8, 123.1, 121.3, 106.5, 93.5, 73.1; HRMS (ESI) *m/z* calcd for $C_{11}H_{11}N_2O^+$: 187.0866 [M+H]$^+$; found: 187.0871.

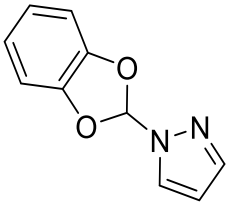

1-(Benzo[d][1,3]dioxol-2-yl)-1H-pyrazole (34). Prepared according to GP2. R_f=0.5 (Cyclohexane:Ethyl acetate 3:1); purified by flash column chromatography on silica gel (Cyclohexane:Ethyl Acetate 97:3) to afford the product as colorless oil (162 mg, 86%). ^{1}H NMR (400 MHz, $CDCl_3$) δ 7.75 (s, 1H), 7.66 (d, *J* = 2 Hz, 1H), 7.60 (d, *J* = 3 Hz, 1H), 6.99 – 6.93 (m, 4H), 6.38 – 6.34 (m, 1H); ^{13}C NMR (100 MHz, $CDCl_3$) δ 145.5, 141.5, 126.7, 122.7, 109.8, 109.1, 107.9; HRMS (FI) *m/z* calcd for $C_{10}H_8N_2O_2$: 188.0586; found: 188.0582.

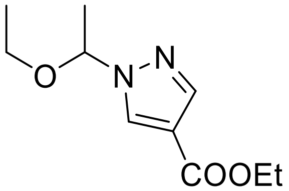

Ethyl 1-(1-ethoxyethyl)-1H-pyrazole-4-carboxylate (35). Prepared according to GP2. R_f=0.6 (Cyclohexane:Ethyl acetate 3:1); purified by flash column chromatography on silica gel (Cyclohexane:Ethyl Acetate 95:5) to afford the product as colorless oil (145 mg, 68%). ^{1}H NMR (400 MHz, $CDCl_3$) δ 8.07 (s, 1H), 7.89 (s, 1H), 5.50 (q, *J* = 6 Hz, 1H), 4.28 (q, *J* = 7 Hz, 2H), 3.46 (dq, J_1 = 9 Hz, J_2 = 7 Hz, 1H), 3.34 (dq, J_1 = 9 Hz, J_2 = 7 Hz, 1H), 1.65 (d, *J* = 6 Hz, 3H), 1.33 (t, *J* = 7 Hz, 3H), 1.14 (t, *J* = 7 Hz, 3H); ^{13}C NMR (100 MHz, $CDCl_3$) δ 163.1, 140.5, 129.7, 116.0, 88.2, 64.6, 60.4, 22.3, 14.9, 14.5; HRMS (ESI) *m/z* calcd for $C_{10}H_{17}N_2O_3^+$: 213.1234 [M+H]$^+$; found: 213.1239.

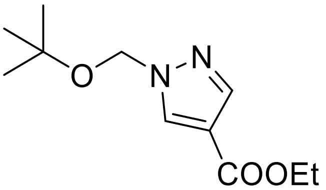

Ethyl 1-(tert-butoxymethyl)-1H-pyrazole-4-carboxylate (36). Prepared according to GP2. R_f=0.6 (Cyclohexane:Ethyl acetate 3:1); purified by flash column chromatography on silica gel (Cyclohexane:Ethyl Acetate 95:5) to afford the product as colorless oil (75 mg, 33%); ^{1}H NMR (400 MHz, $CDCl_3$) δ 8.05 (s, 1H), 7.86 (d, *J* = 1 Hz, 1H), 5.45 (s, 2H), 4.25 (q, *J* = 7 Hz, 2H), 1.30 (t, *J* = 7 Hz, 3H), 1.17 (s, 9H); ^{13}C NMR (100 MHz, $CDCl_3$) δ 163.0, 140.8, 132.5, 116.2, 76.0, 75.4, 60.3, 27.9, 14.4; HRMS (ESI) *m/z* calcd for $C_{11}H_{19}N_2O_3^+$: 227.1390 [M+H]$^+$; found: 227.1396.

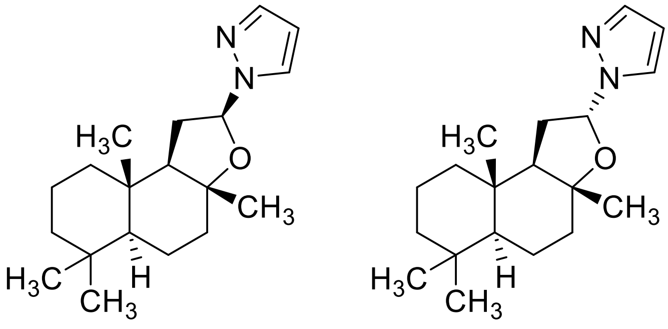

37 **37'**

1-((2S,3aR,5aS,9aS,9bR)-3a,6,6,9a-tetramethyldodecahydronaphtho[2,1-b]furan-2-yl)-1H-pyrazole (37) and 1-((2R,3aR,5aS,9aS,9bR)-3a,6,6,9a-tetramethyldodecahydronaphtho[2,1-b]furan-2-yl)-1H-pyrazole (37′). Prepared according to GP2. (**1l**, 6 equiv, CH_3CN/CH_2Cl_2 5:1, 6 mL). Purified by flash column chromatography on silica gel (Cyclohexane:Diethyl ether 75:25) to afford the product **37** (164 mg, 54%) and **37′** (102.7 mg, 34%) as two colorless oils. Ratio **37**:**37′** = 1.6:1.

Characterization for **37**

Major: R_f=0.5 (Cyclohexane: Diethyl ether 3:1); ^{1}H NMR (400 MHz, $CDCl_3$) δ 7.63 (d, *J* = 2 Hz, 1H), 7.55 (d, *J* = 2 Hz, 1H), 6.27 (t, *J* = 2 Hz, 1H), 5.90 (dd, J_1 = 8 Hz, J_2 = 7 Hz, 1H), 2.72 – 2.61 (m, 1H), 2.28 (dt, J_1 = 12 Hz, J_2 = 6 Hz, 1H), 1.98 (dt, J_1 = 12 Hz, J_2 = 3 Hz, 1H), 1.83 – 1.61 (m, 3H), 1.55 – 1.39 (m, 4H), 1.39 – 1.27 (m, 1H), 1.26 – 1.15 (m, 4H), 1.09 (td, J_1 = 13 Hz, J_2 = 3 Hz, 1H), 1.01 (dd, J_1 = 12 Hz, J_2 = 3 Hz, 1H), 0.92 (s, 3H), 0.88 (s, 3H), 0.83 (s, 3H); ^{13}C NMR (100 MHz, $CDCl_3$) δ 139.6, 129.0, 105.8, 89.4, 83.3, 60.6, 57.2, 42.5, 40.2, 40.0, 36.5, 33.6, 33.2, 29.1, 23.8, 21.2, 20.8, 18.5, 15.6; HRMS (ESI) *m/z* calcd for $C_{19}H_{31}N_2O^+$: 303.2431 [M+H]$^+$; found: 303.2436.

Characterization for **37′**

Minor: R_f=0.4 (Cyclohexane:Ether 3:1); ^{1}H NMR (400 MHz, $CDCl_3$) δ 7.57 (d, *J* = 2 Hz, 1H), 7.55 (d, *J* = 2 Hz, 1H), 6.24 (t, *J* = 2 Hz, 1H), 6.02 (dd, J_1 = 8 Hz, J_2 = 1 Hz, 1H), 2.32 – 2.21 (m, 1H), 2.17 –2.08 (m, 1H), 2.03 (dt, J_1 = 12 Hz, J_2 = 3 Hz, 1H), 1.89 (dd, J_1 = 14 Hz, J_2 = 7 Hz, 1H), 1.86 – 1.76 (m, 1H), 1.72 – 1.55 (m, 2H), 1.46 – 1.36 (m, 3H), 1.35 – 1.14 (m, 5H), 1.13 – 1.04 (m, 2H), 0.88 (s, 3H), 0.87 (s, 3H), 0.83 (s, 3H); ^{13}C NMR (100 MHz, $CDCl_3$) δ 140.0, 127.7, 105.5, 88.4, 83.8, 58.1, 57.1, 42.4, 39.9, 39.9, 36.3, 33.6, 33.2, 30.7, 22.4, 21.2, 20.8, 18.4, 15.3; HRMS (ESI) *m/z* calcd for $C_{19}H_{31}N_2O^+$: 303.2431 [M+H]$^+$; found: 303.2436.

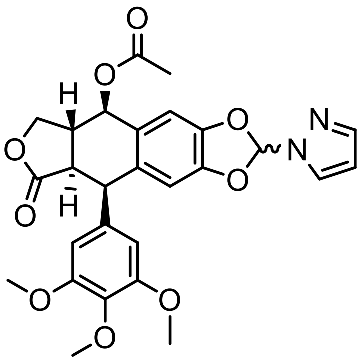

(5R,5aR,8aR,9R)-8-oxo-2-(1H-pyrazol-1-yl)-9-(3,4,5-trimethoxyphenyl)-5,5a,6,8,8a,9-hexahydrofuro[3',4':6,7]naphtho[2,3-d][1,3]dioxol-5-yl acetate (38), Prepared according to GP2 (**1m**, 2 equiv, CH_3CN 15 mL)**.** R_f=0.35 (Cyclohexane/Ethyl acetate 1:1); m.p. 111-113 °C; purified by flash column chromatography on silica gel (Cyclohexane:Diethyl ether 65:35) to afford the product as a mixture of two diastereomers as white solid (211 mg, 40%, dr = 2:1). and starting material **1m** (127.7 mg, 28%). Yield based on remaining starting material: 56%. ^{1}H NMR (400 MHz, $CDCl_3$) δ 7.74 (s, 0.3H_{minor}), 7.74 (s, 0.6H_{major}), 7.64 (d, J = 2 Hz, 0.3H_{minor}), 7.63 (d, J = 2 Hz, 0.6H_{major}), 7.60 (d, J = 3 Hz, 0.3H_{minor}), 7.58 (d, J = 3 Hz, 0.7H_{minor}), 6.92 (s, 1H), 6.68 (s, 1H), 6.39 – 6.33 (m, 3H), 5.93 – 5.87 (m, 1H), 4.66 – 4.62 (m, 1H), 4.40 – 4.34 (m, 1H), 4.23 – 4.16 (m, 1H), 3.79 (d, J = 1 Hz, 3H), 3.74 (d, J = 4 Hz, 6H), 3.00 – 2.78 (m, 2H), 2.18 (d, J = 1 Hz, 3H); ^{13}C NMR (100 MHz, $CDCl_3$) δ 173.6, 171.4, 171.4, 152.8, 152.7, 146.2, 145.7, 142.0, 141.9, 137.3, 137.3, 134.8, 134.7, 133.4, 133.4, 129.5, 129.5, 127.0, 126.9, 110.2, 110.2, 110.1, 108.2, 108.2, 107.9, 107.6, 73.6, 71.3, 60.8, 56.2, 56.2, 45.6, 45.5, 43.9, 43.8, 38.7, 38.7, 21.2; HRMS (ESI): m/z calcd for $C_{27}H_{27}N_2O_9^+$: 523.1711 [M+H]$^+$; found: 523.1717.

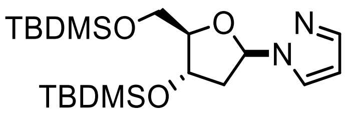

1-((2R,4S,5R)-4-((tert-butyldimethylsilyl)oxy)-5-(((tert-butyldimethylsilyl)oxy)methyl)tetrahydrofuran-2-yl)-1H-pyrazole (39). Prepared according to GP2 (**2a**, 0.2 mmol, **1n** 2 equiv, CH_3CN 1 mL). R_f=0.4 (Cyclohexane:Ethyl acetate 10:1); purified by flash column chromatography on silica gel (Cyclohexane: Ethyl Acetate 95:5) to afford the product as colorless oil (23.1 mg, 28%). and starting material **1n** (6.8 mg, 50%). Yield based on remaining starting material: 56%. ^{1}H NMR (400 MHz, $CDCl_3$) δ 7.87 (d, J = 2 Hz, 1H), 7.51 (d, J = 2 Hz, 1H), 6.26 (t, J = 2 Hz, 1H), 6.14 (dd, J_1 = 7 Hz, J_2 = 3 Hz, 1H), 4.48 (dt, J_1 = 6 Hz, J_2 = 3 Hz, 1H), 4.21 – 4.16 (m, 1H), 3.73 – 3.62 (m, 2H), 2.72 – 2.63 (m, 1H), 2.38 (dt J_1 = 14 Hz, J_2 = 3 Hz, 1H), 0.90 (s, 9H), 0.87 (s, 9H), 0.08 (s, 3H), 0.07 (s, 6H), 0.05 (s, 3H); ^{13}C NMR (100 MHz, $CDCl_3$) δ 139.4, 127.9, 106.0, 91.0, 88.6, 72.6, 63.2, 41.5, 26.0, 25.9, 18. 5, 18.1, -4.7, -4.7, -5.2, -5.4; HRMS (FD): m/z calcd for $C_{20}H_{40}N_2O_3Si_2$: 412.2577; found: 412.2577.

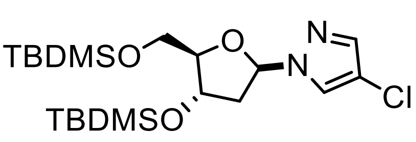

1-((2R,4S,5R)-4-((tert-butyldimethylsilyl)oxy)-5-(((tert-butyldimethylsilyl)oxy)methyl)tetrahydrofuran-2-yl)-1H-pyrazole (40). Prepared according to GP2 (**2a**, 0.2 mmol, **1n** 2 equiv, CH_3CN 1 mL). R_f=0.6 (Cyclohexane:Ethyl acetate 10:1); purified by flash column

chromatography on silica gel (Cyclohexane: Ethyl Acetate 98:2) to afford the product as colorless oil (17.8 mg, 20%). and starting material **1n** (12.1 mg, 60%). Yield based on remaining starting material: 50%. ^{1}H NMR (400 MHz, $CDCl_3$) δ 7.90 (s, 1H), 7.41 (s, 1H), 6.05 (dd, *J* = 7.5, 2.2 Hz, 1H), 4.47 (dt, J_1 = 6.4 Hz, J_2= 2.1 Hz, 1H), 4.21 (dt, J_1 = 5.2 Hz, J_2 = 2.8 Hz, 1H), 3.73 – 3.57 (m, 2H), 2.68 – 2.59 (m, 1H), 2.32 (dt, J_1 = 14.2 Hz, J_2 = 2.2 Hz, 1H), 0.91 (s, 9H), 0.88 (s, 9H), 0.10 – 0.07 (m, 9H), 0.06 (s, 3H). ^{13}C NMR (100 MHz, $CDCl_3$) δ 137.7, 126.2, 110.5, 92.1, 89.5, 72.9, 63.4, 41.6, 26.1, 25.8, 18.5, 18.0, -4.7, -5.2, -5.4; HRMS (FD): *m/z* calcd for $C_{20}H_{40}ClN_2O_3Si^+$: 447.2261 [*M*+H]$^+$; found: 447.2266.

8. Reference

[1] S. Protti, D. Ravelli, M. Fagnoni, A. Albini, *Chem. Commun.* **2009**, 7351–7353.

[2] S. P. Forsey, D. Rajapaksa, N. J. Taylor, R. Rodrigo, *Articles Comprehensive Synthetic Route to Eight Diastereomeric Podophyllum Lignans*, **1989**.

[3] N. Gaß, H. A. Wagenknecht, *Eur. J. Org. Chem.* **2015**, *30*, 6661–6668.

[4] M. A. Cameron, S. B. Cush, R. P. Hammer, *Facile Preparation of Protected Furanoid Glycals from Thymidine*, Walker, **1997**.

[5] Y. Nagaya, Y. Kitamura, R. Nakashima, A. Shibata, M. Ikeda, Y. Kitade, *Nucleosides, Nucleotides and Nucleic Acids* **2016**, *35*, 64–75.

[6] J. Chem ; Eriksson, M. Nielsen, P. E. Uhlmann, E. Peyman, A. Breipohl, G. Will, D. W. Coull, J. M. Hodge, *J. Org. Chem.* **2000**, *65*, 7697–7699.

[7] M. A. Cismesia, T. P. Yoon, *Chem. Sci.* **2015**, *6*, 5426–5434.

[8] R. C. Johnson, *J. Chem. Educ.* **1970**, *47*, 702.

[9] C. G. Hatchard, C. A. Parker, *Proc. R. Soc. London. Ser. A. Math. Phys. Sci.* **1956**, *235*, 518–536.

[10] H. Aruri, U. Singh, S. Sharma, S. Gudup, M. Bhogal, S. Kumar, D. Singh, V. K. Gupta, R. Kant, R. A. Vishwakarma, P. P. Singh, *J. Org. Chem.* **2015**, *80*, 1929–1936.

[11] T. Sheng, H.-J. Zhang, M. Shang, C. He, J. C. Vantourout, P. S. Baran, *Org. Lett.* **2020**, *22*, 7594–7598.

[12] L. Zhang, H. Yi, J. Wang, A. Lei, *J. Org. Chem.* **2017**, *82*, 10704–10709.

[13] X. Shao, Y. Zheng, L. Tian, I. Martín-Torres, A. M. Echavarren, Y. Wang, *Org. Lett.* **2019**, *21*, 9262–9267.

[14] J. Wu, Y. Zhou, Y. Zhou, C.-W. Chiang, A. Lei, *ACS Catal.* **2017**, *7*, 8320–8323.

[15] H. M. Guo, C. Xia, H. Y. Niu, X. T. Zhang, S. N. Kong, D. C. Wang, G. R. Qu, *Adv. Synth. Catal.* **2011**, *353*, 53–56.

[16] Z. Luo, Z. Jiang, W. Jiang, D. Lin, *J. Org. Chem.* **2018**, *83*, 3710–3718.

[17] D. Stewart, J. Trauth, J. Windholtz, K. M. Church, *Synth. Commun.* **1996**, *26*, 4279–4288.

[18] Y. Zhan, X. Ding, H. Wang, H. Yu, F. Ren, *Tetrahedron Lett.* **2018**, *59*, 2150–2153.

[19] C. G. Kruse, E. K. Poels, A. Van der Gen, *J. Org. Chem.* **1979**, *44*, 2911–2915.

[20] I. B. Perry, T. F. Brewer, P. J. Sarver, D. M. Schultz, D. A. DiRocco, D. W. C. MacMillan, *Nature* **2018**, *560*, 70–75.

[21] Z. Wan, D. Wang, Z. Yang, H. Zhang, S. Wang, A. Lei, *Green Chem.* **2020**, *22*, 3742–3747.

9. NMR Spectra of starting materials 1m and 1n

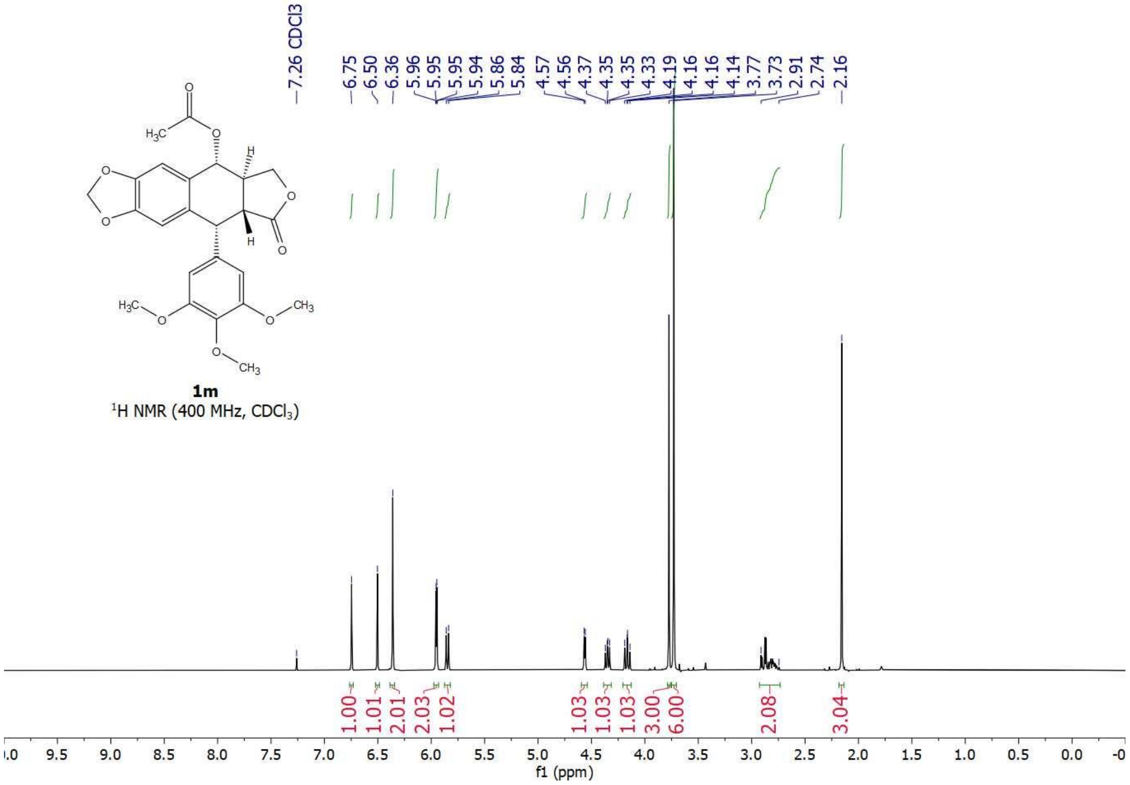

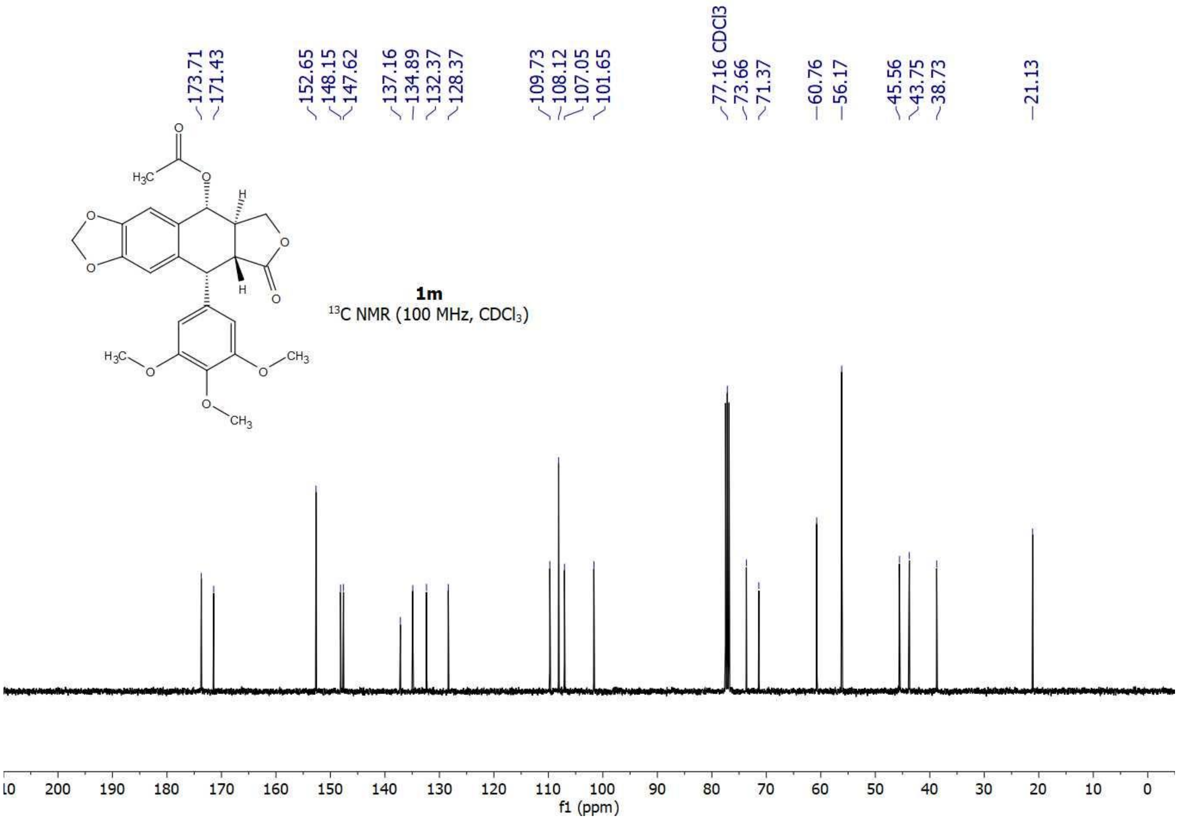

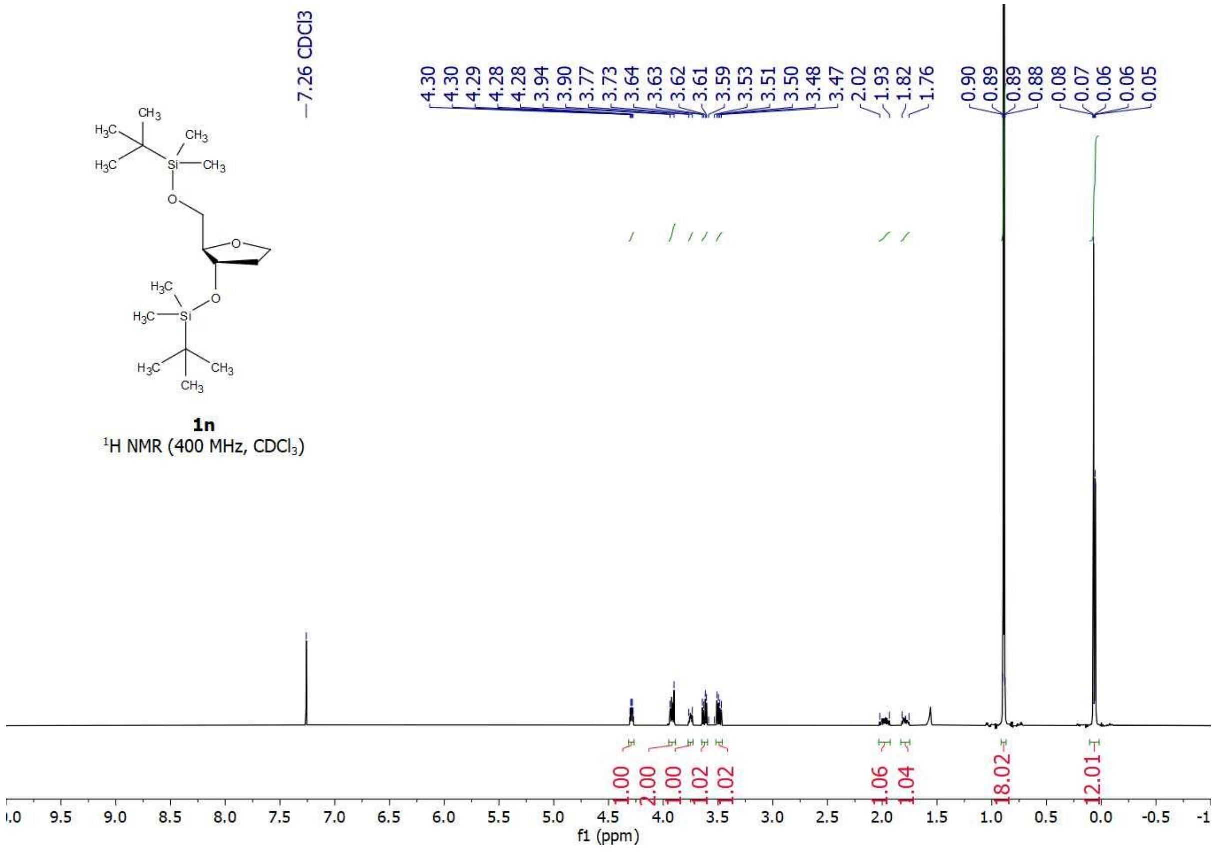

1n
^{1}H NMR (400 MHz, $CDCl_3$)

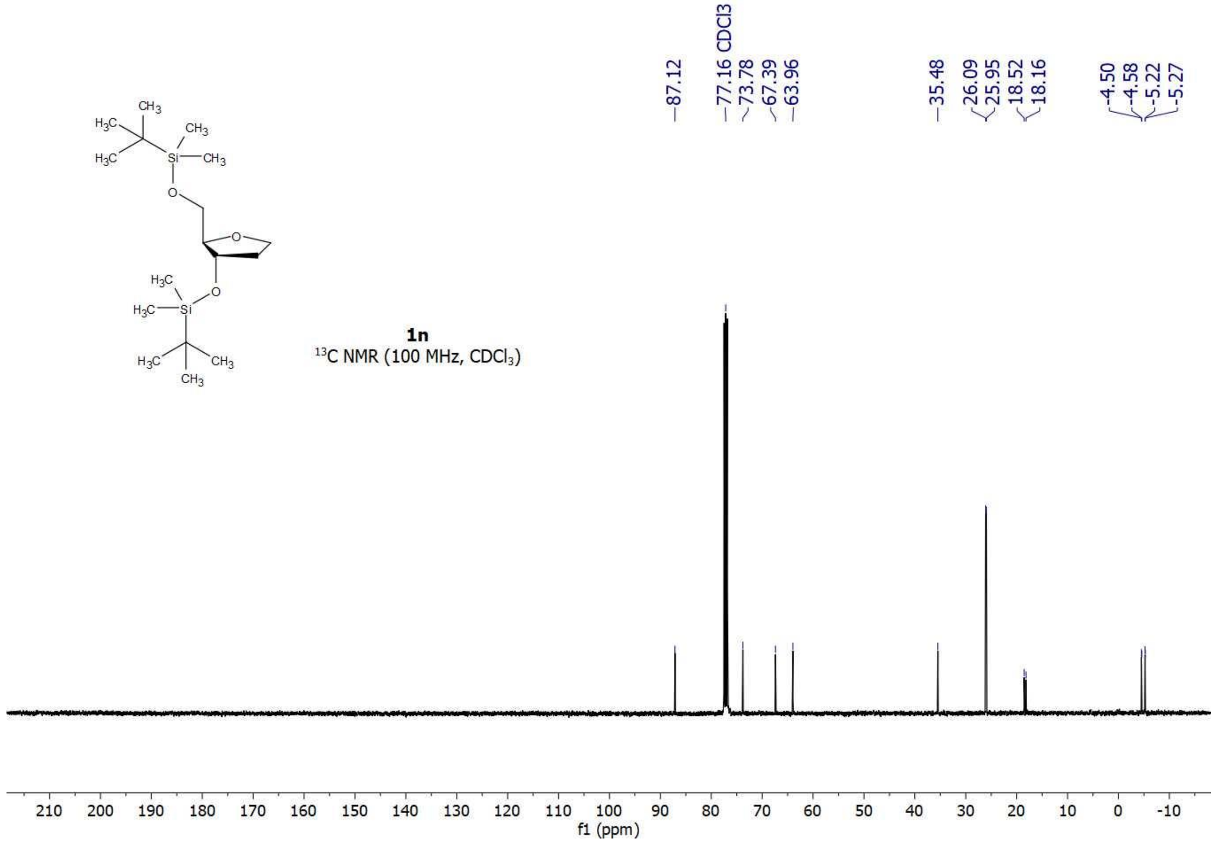

1n
^{13}C NMR (100 MHz, $CDCl_3$)

10. NMR Spectra of compounds 3-40

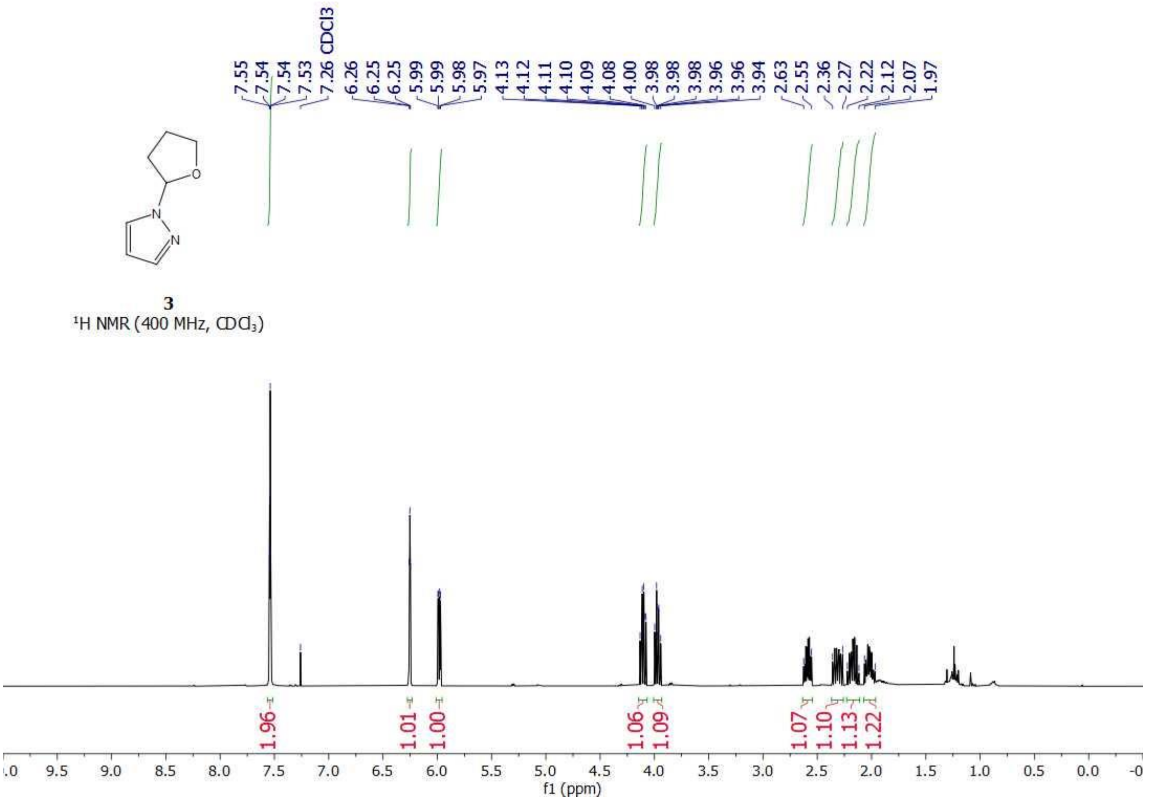

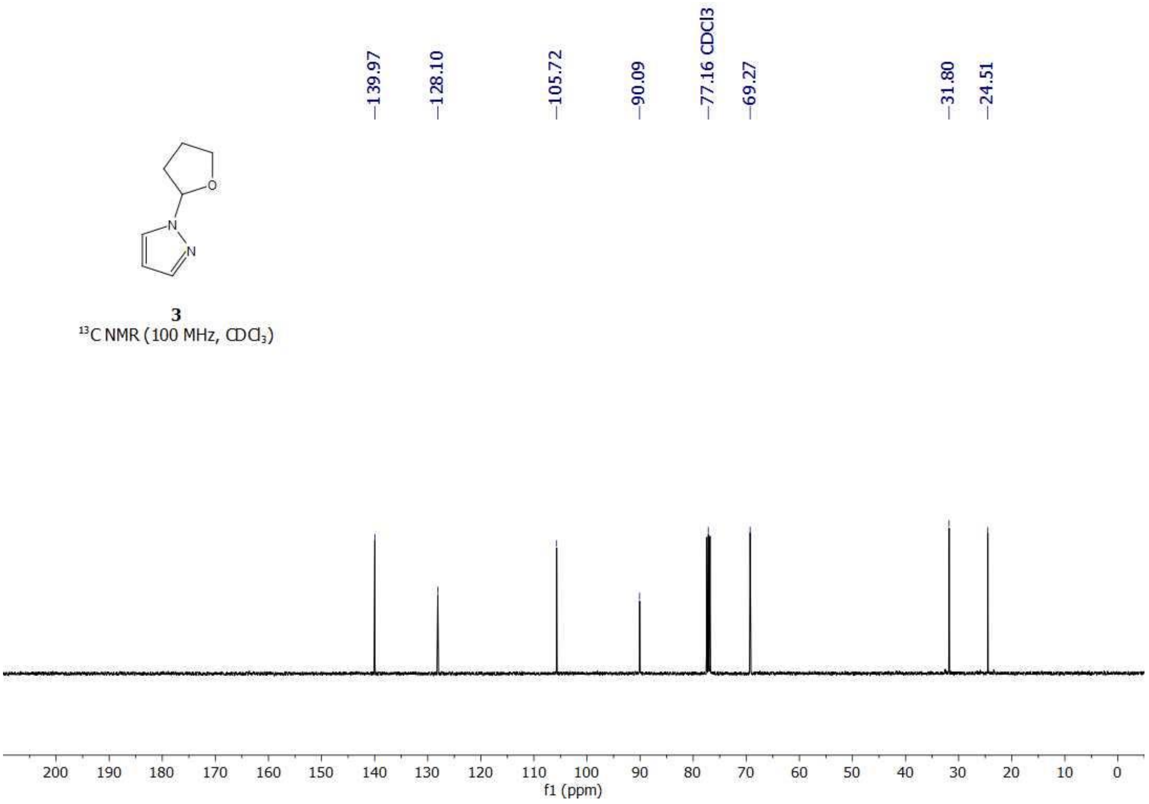

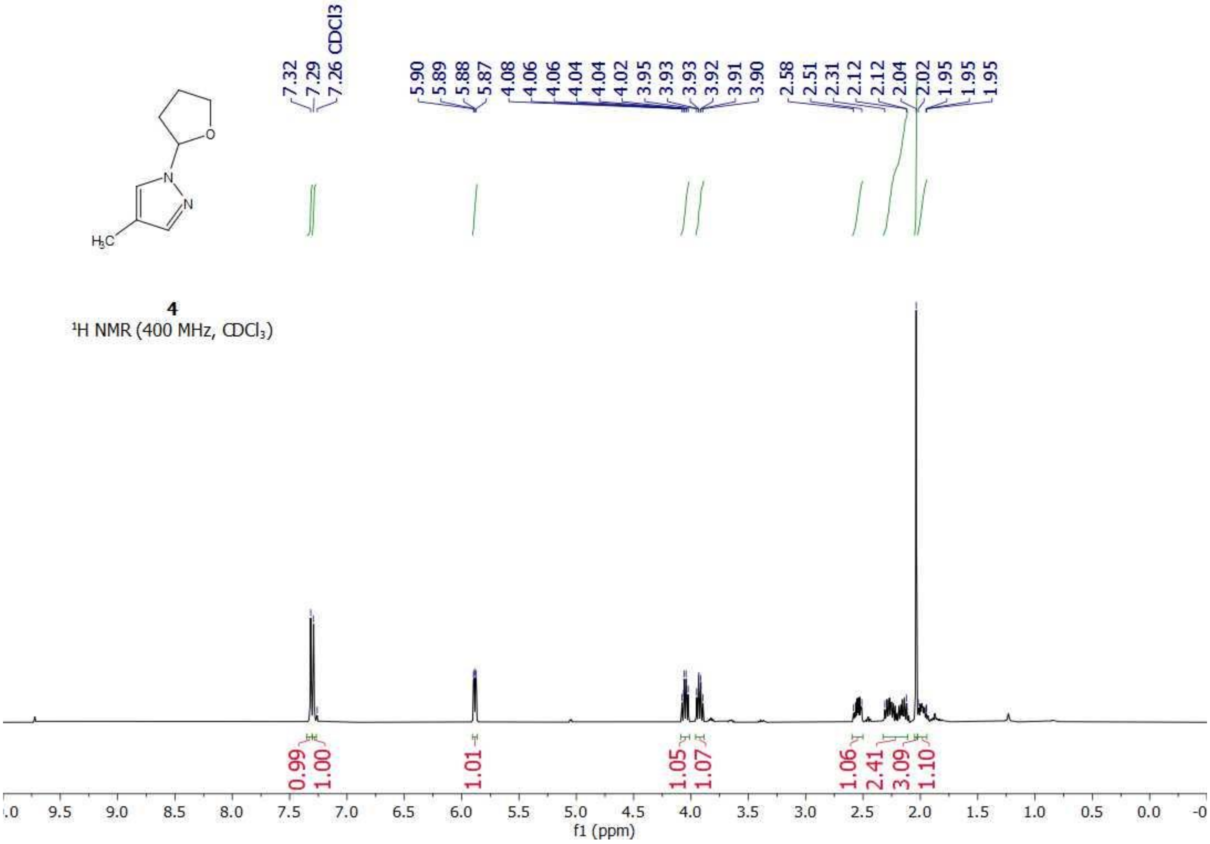

4
^{1}H NMR (400 MHz, $CDCl_3$)

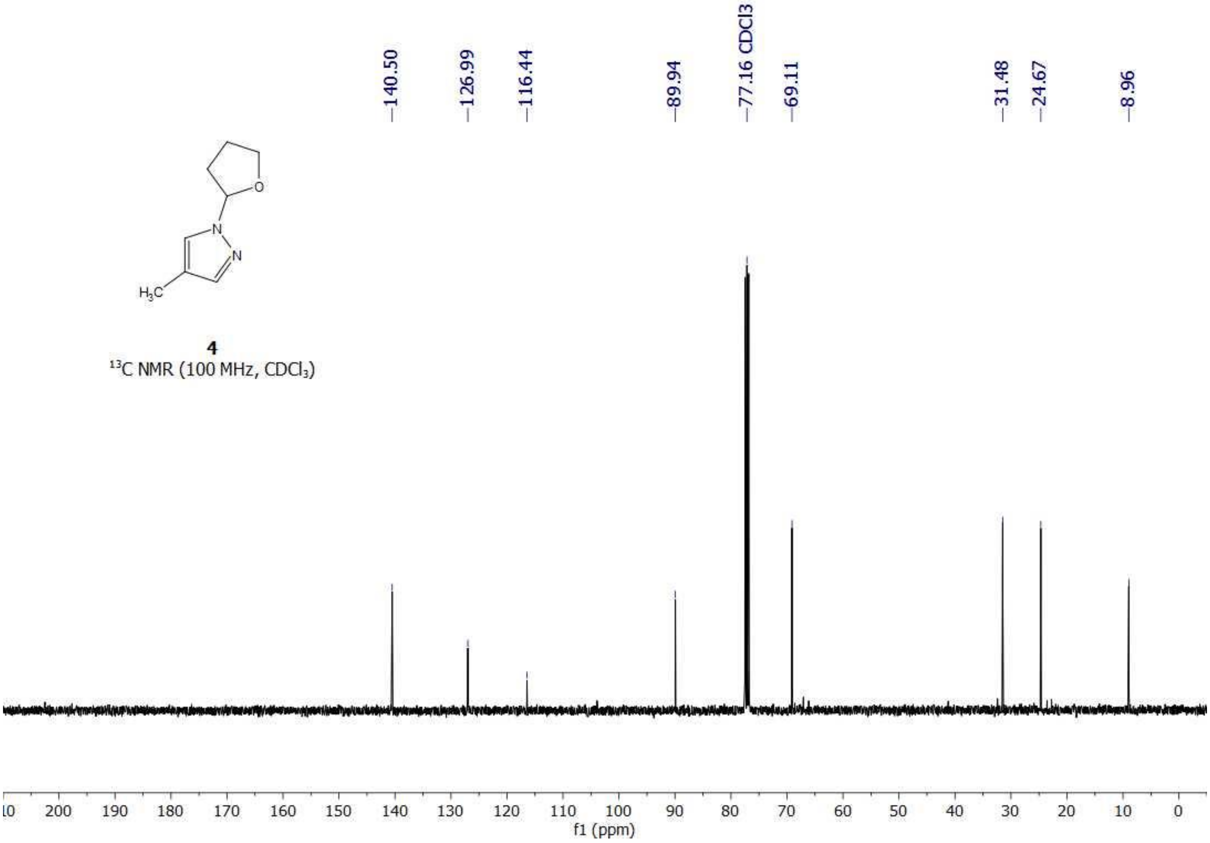

4
^{13}C NMR (100 MHz, $CDCl_3$)

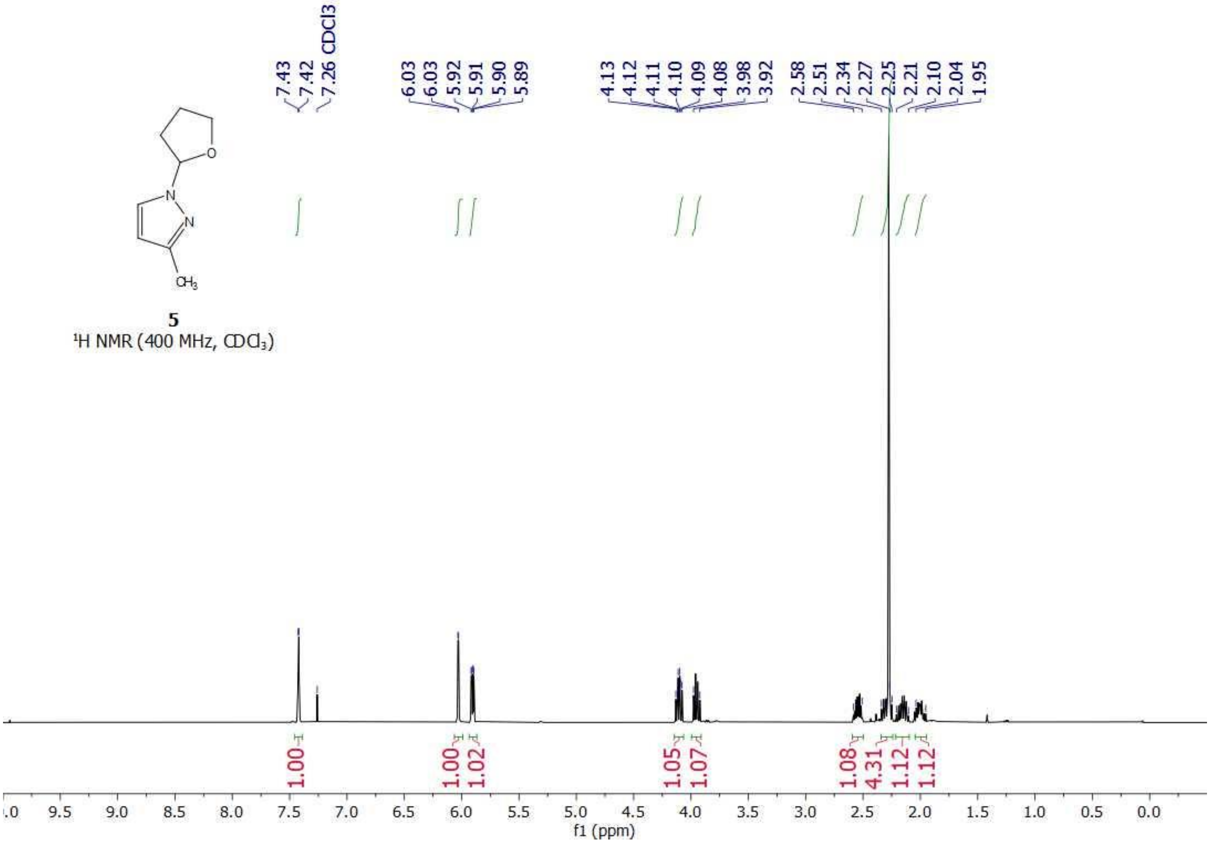

5
^1H NMR (400 MHz, $CDCl_3$)

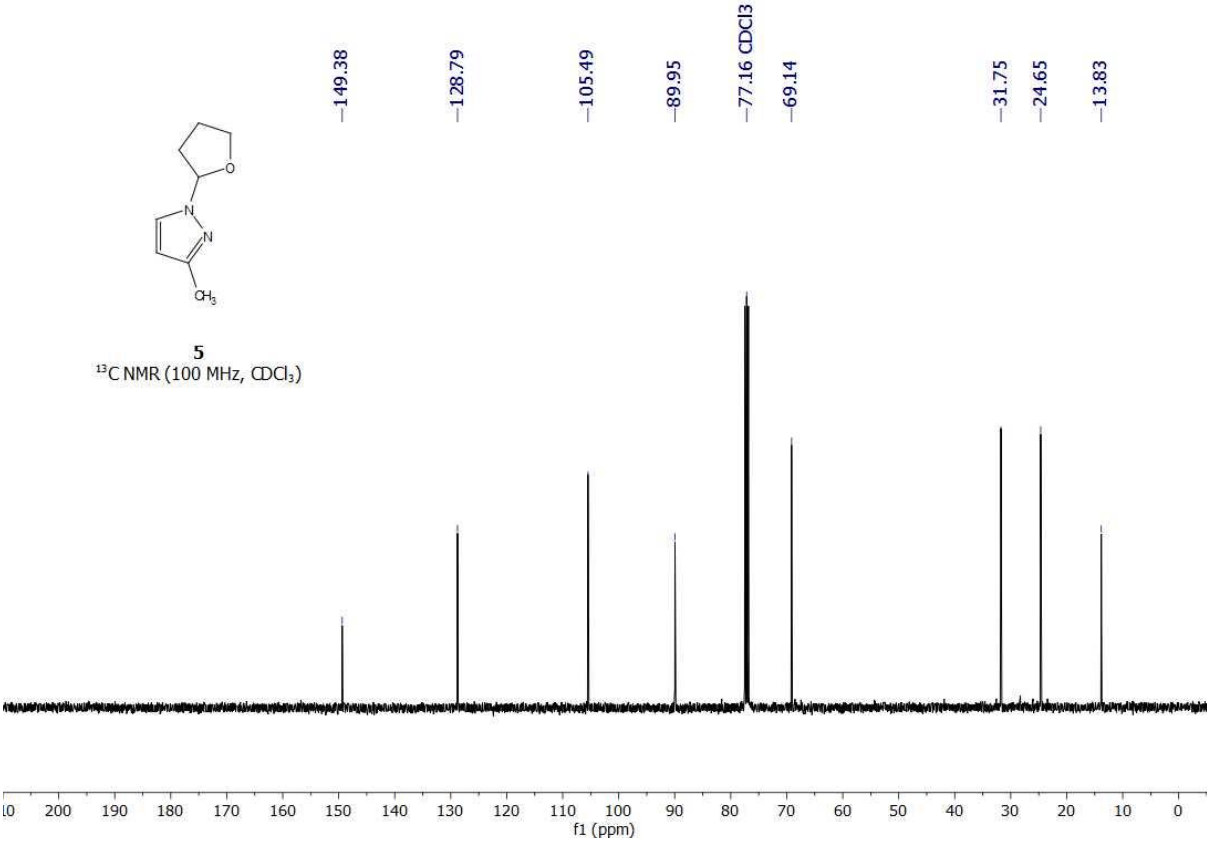

5
^{13}C NMR (100 MHz, $CDCl_3$)

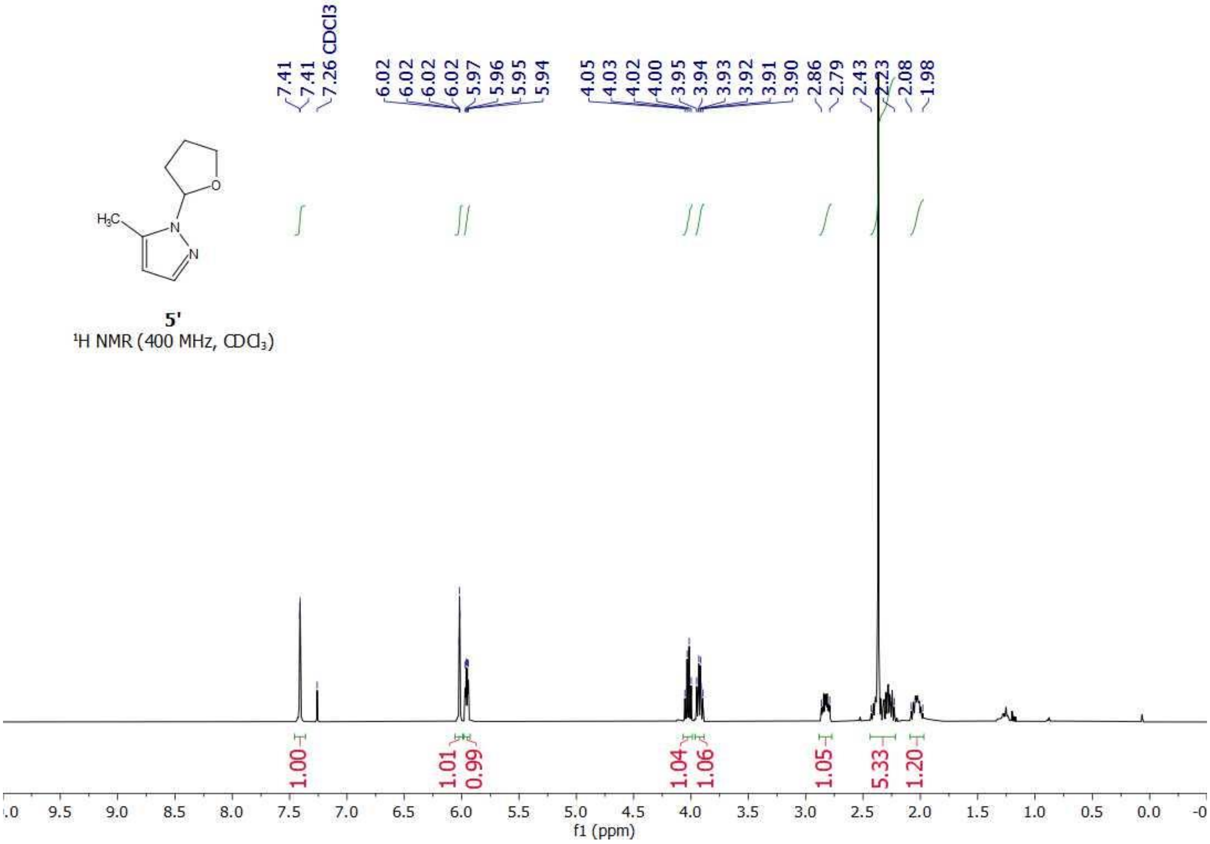

5'

^{1}H NMR (400 MHz, $CDCl_3$)

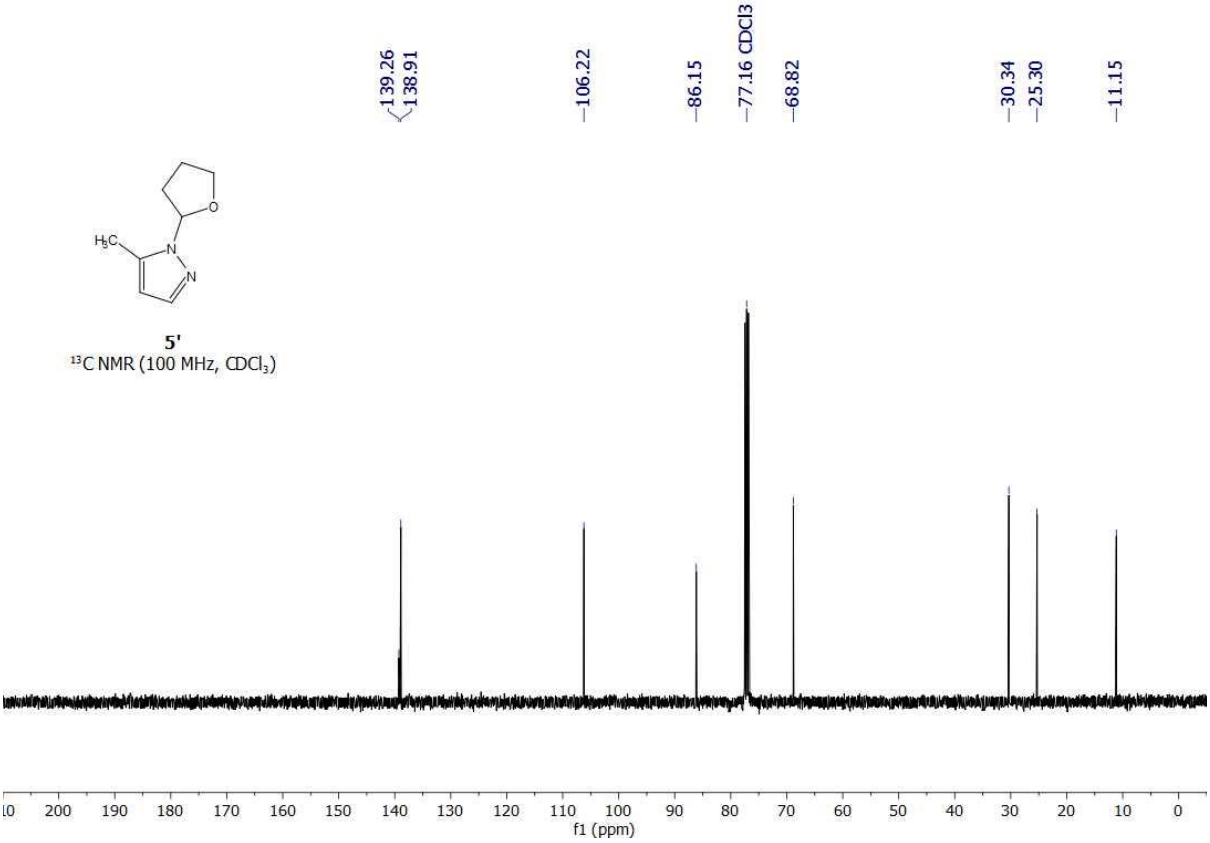

5'

^{13}C NMR (100 MHz, $CDCl_3$)

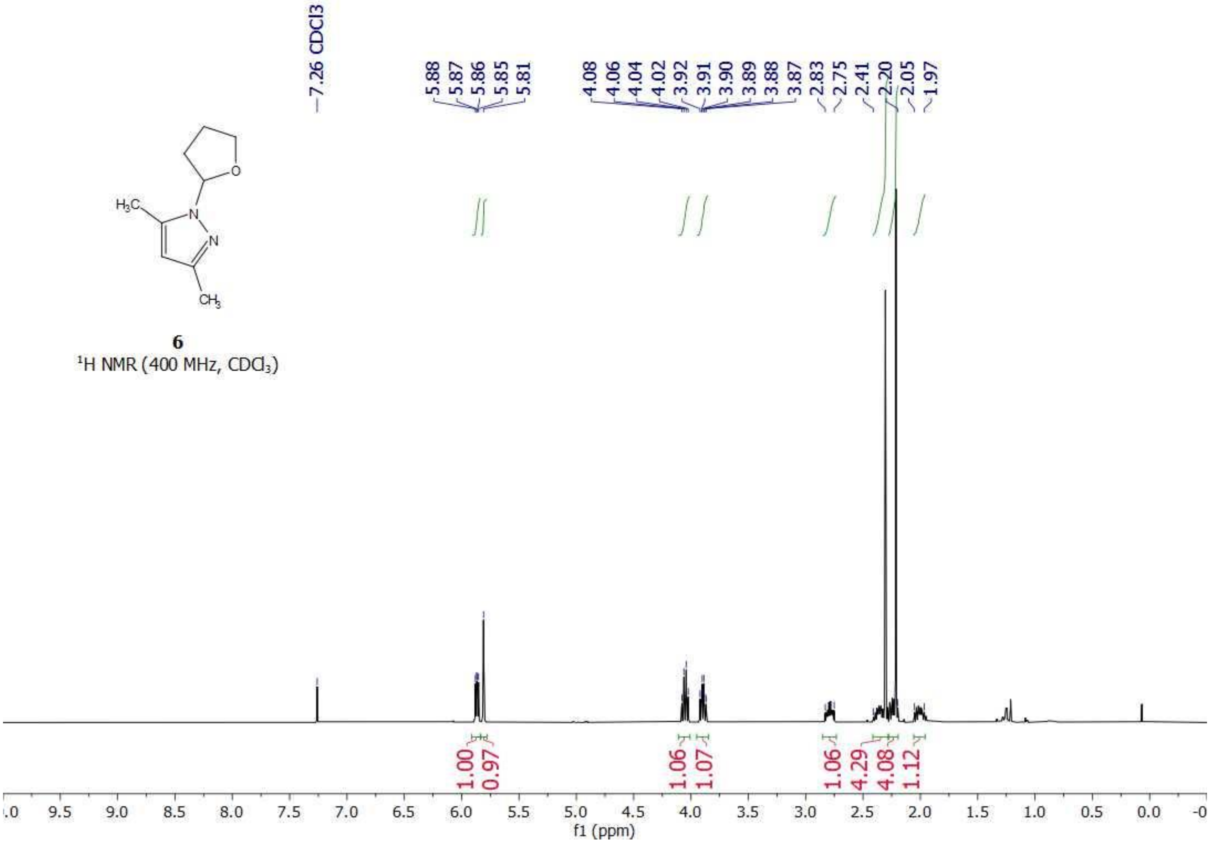

6
^{1}H NMR (400 MHz, $CDCl_3$)

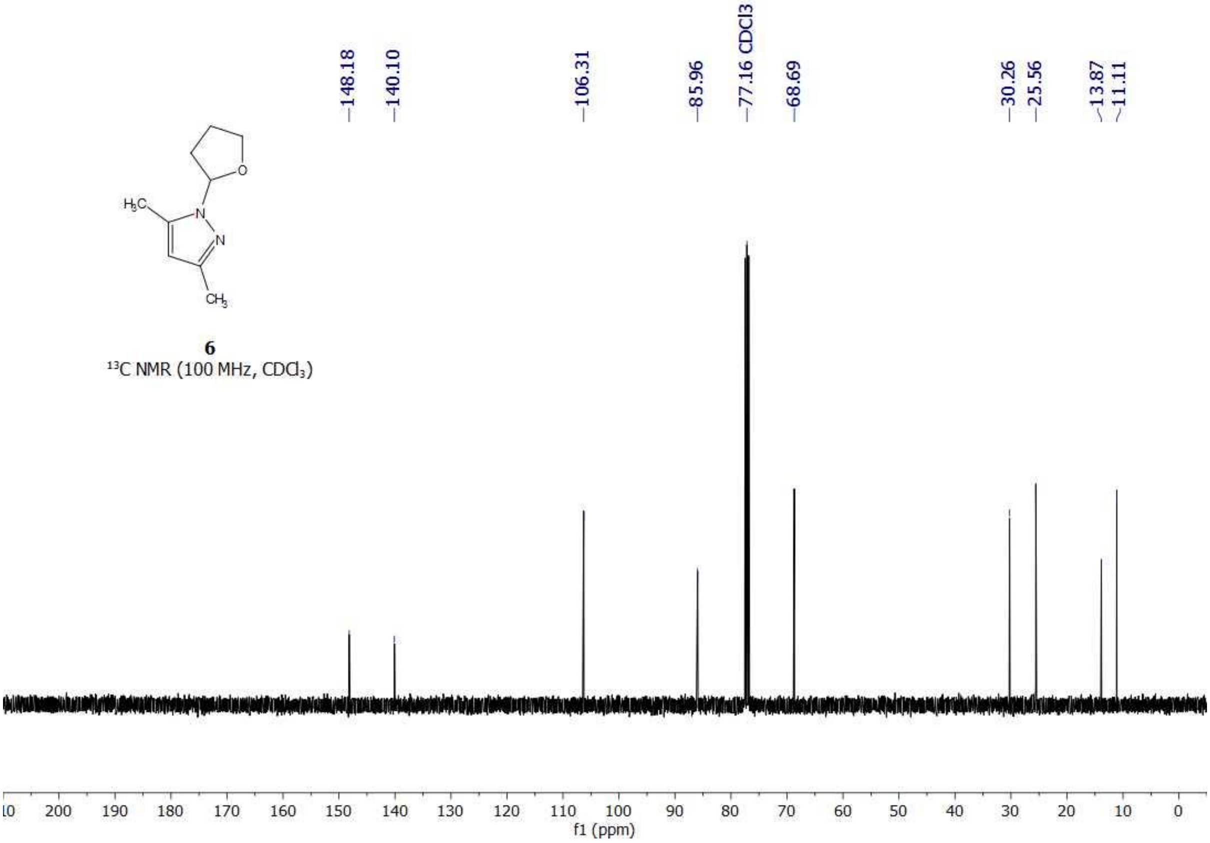

6
^{13}C NMR (100 MHz, $CDCl_3$)

7

^{1}H NMR (400 MHz, CDCl$_3$)

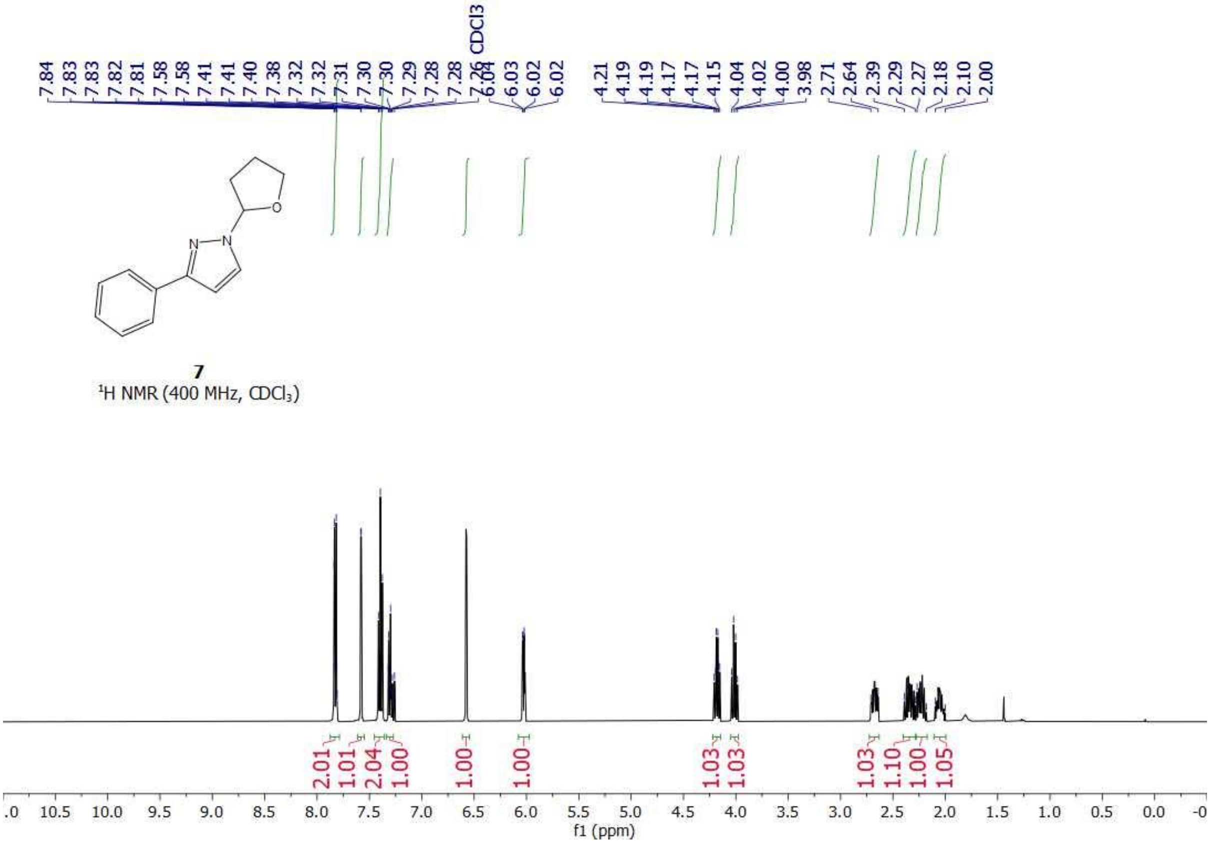

7

^{13}C NMR (100 MHz, CDCl$_3$)

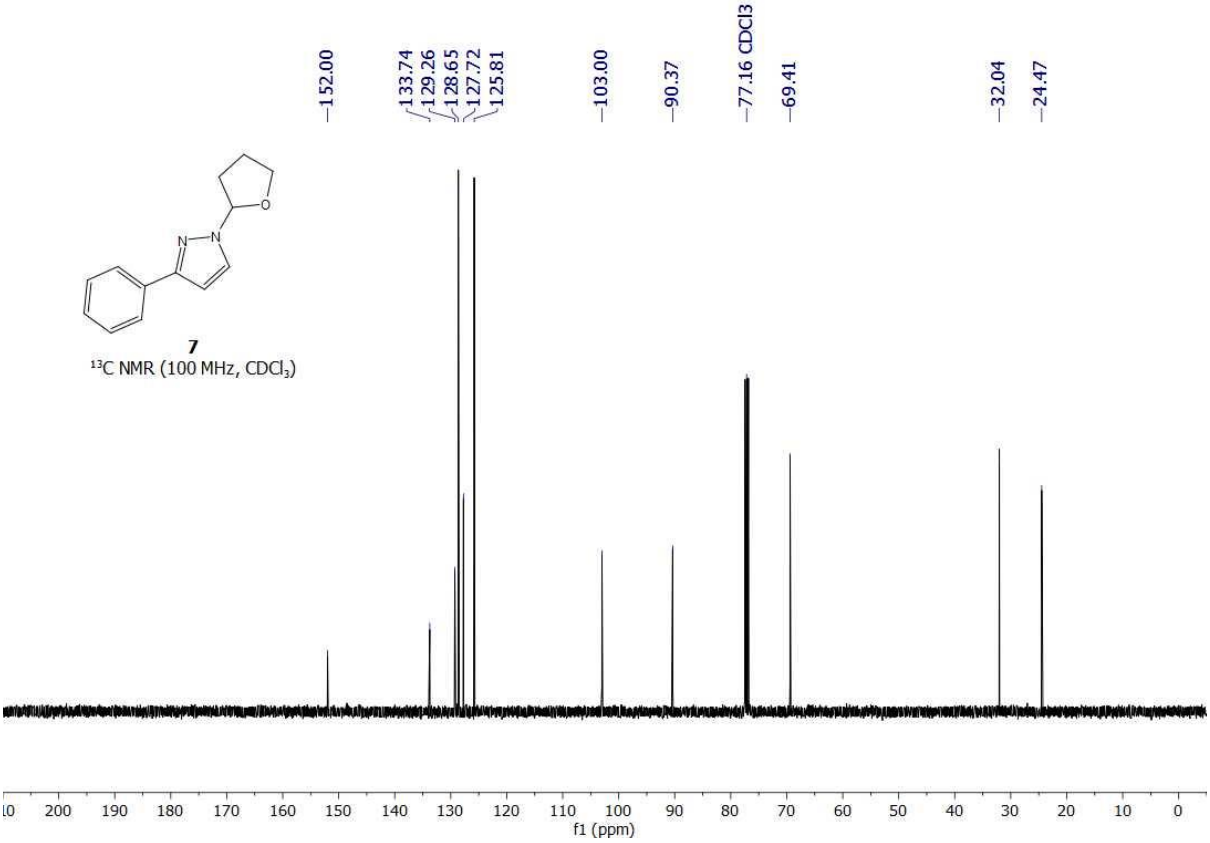

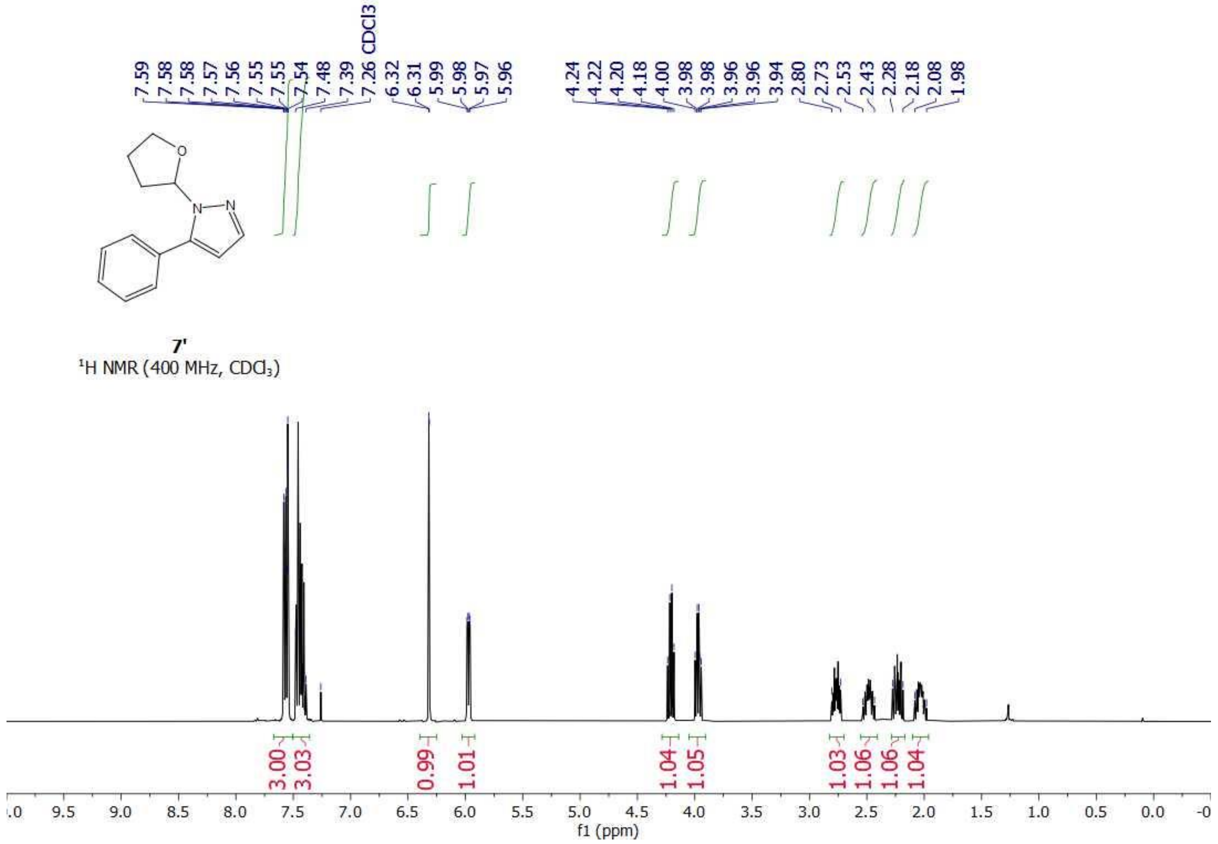

7'
^{1}H NMR (400 MHz, $CDCl_3$)

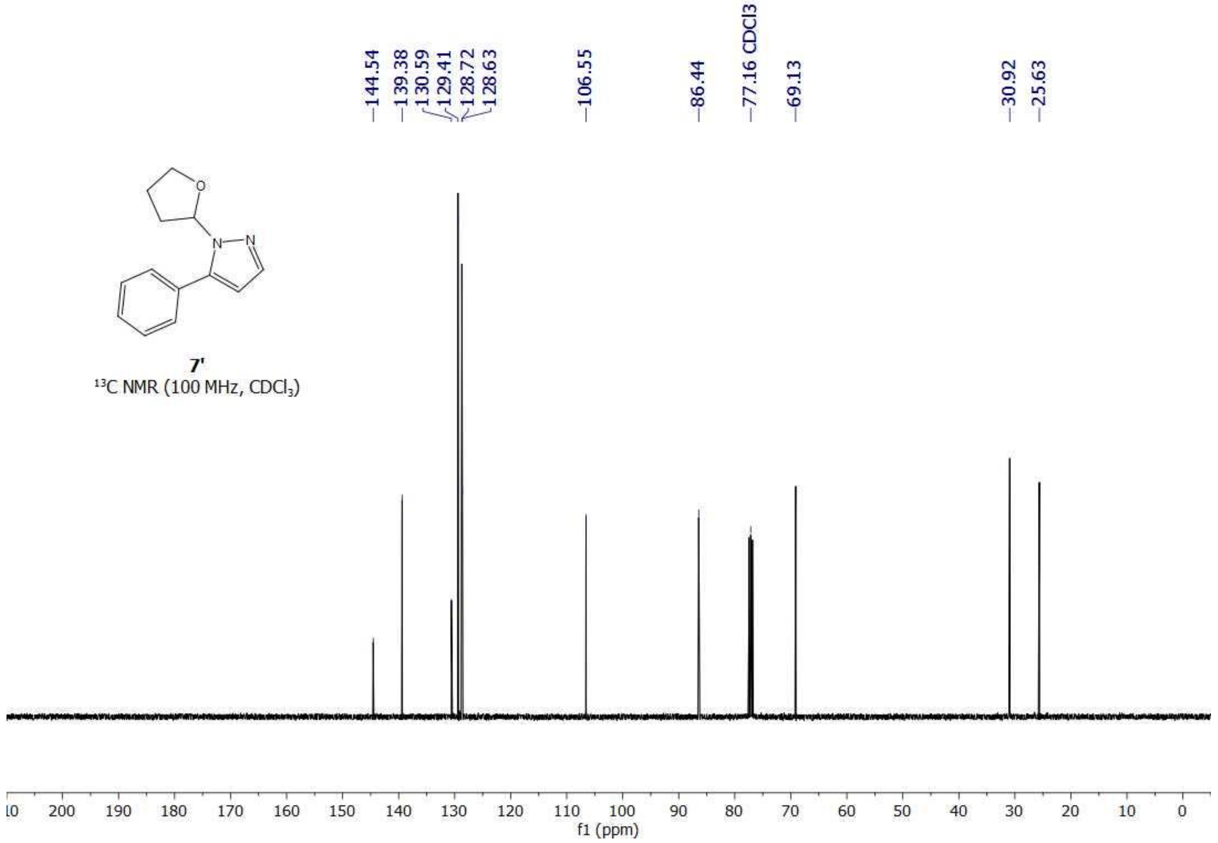

7'
^{13}C NMR (100 MHz, $CDCl_3$)

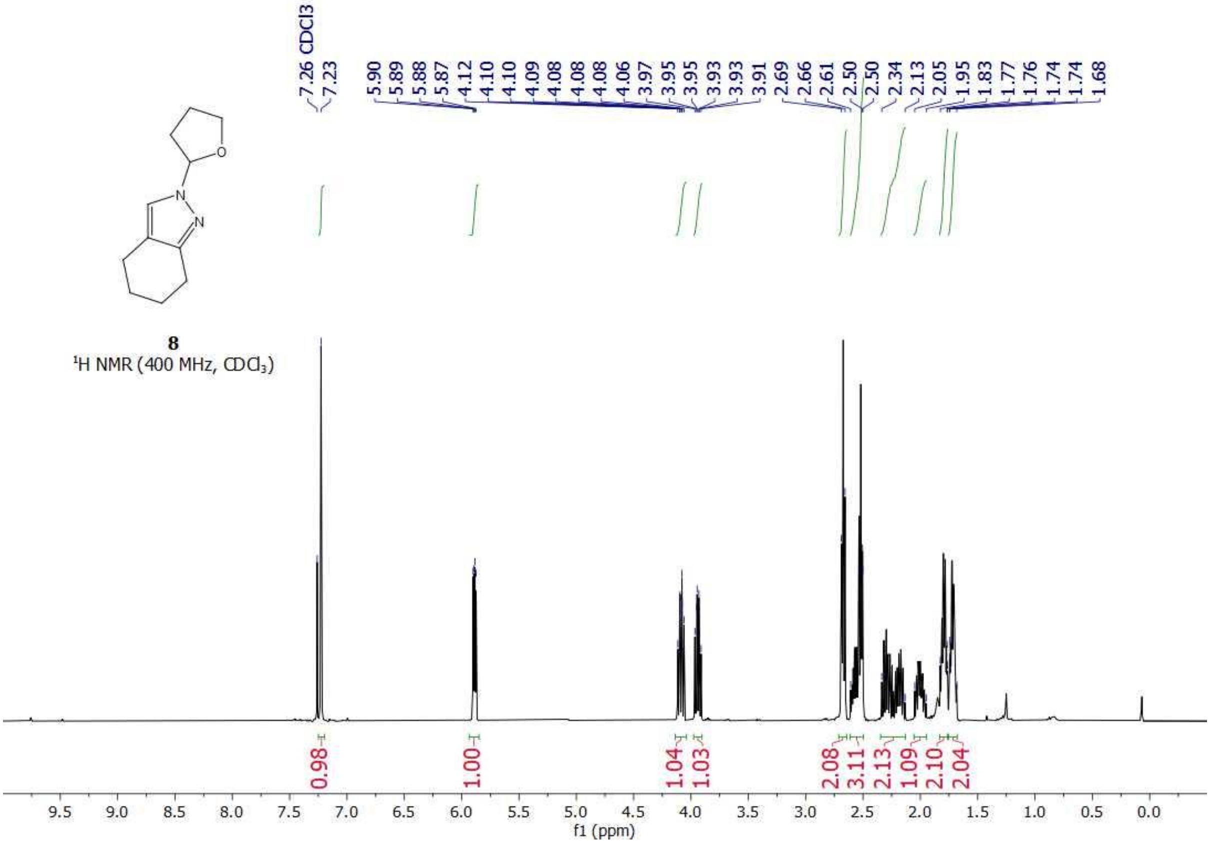

8

^{1}H NMR (400 MHz, $CDCl_3$)

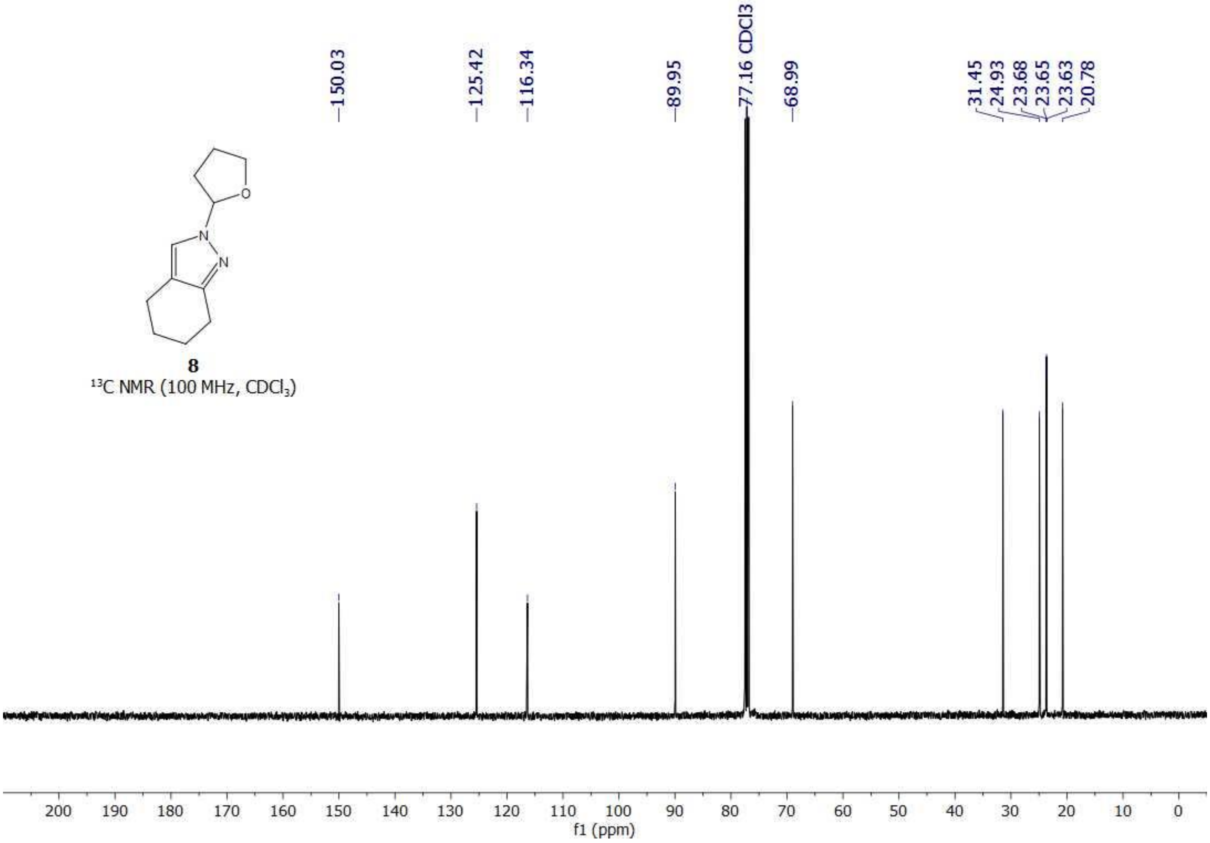

8

^{13}C NMR (100 MHz, $CDCl_3$)

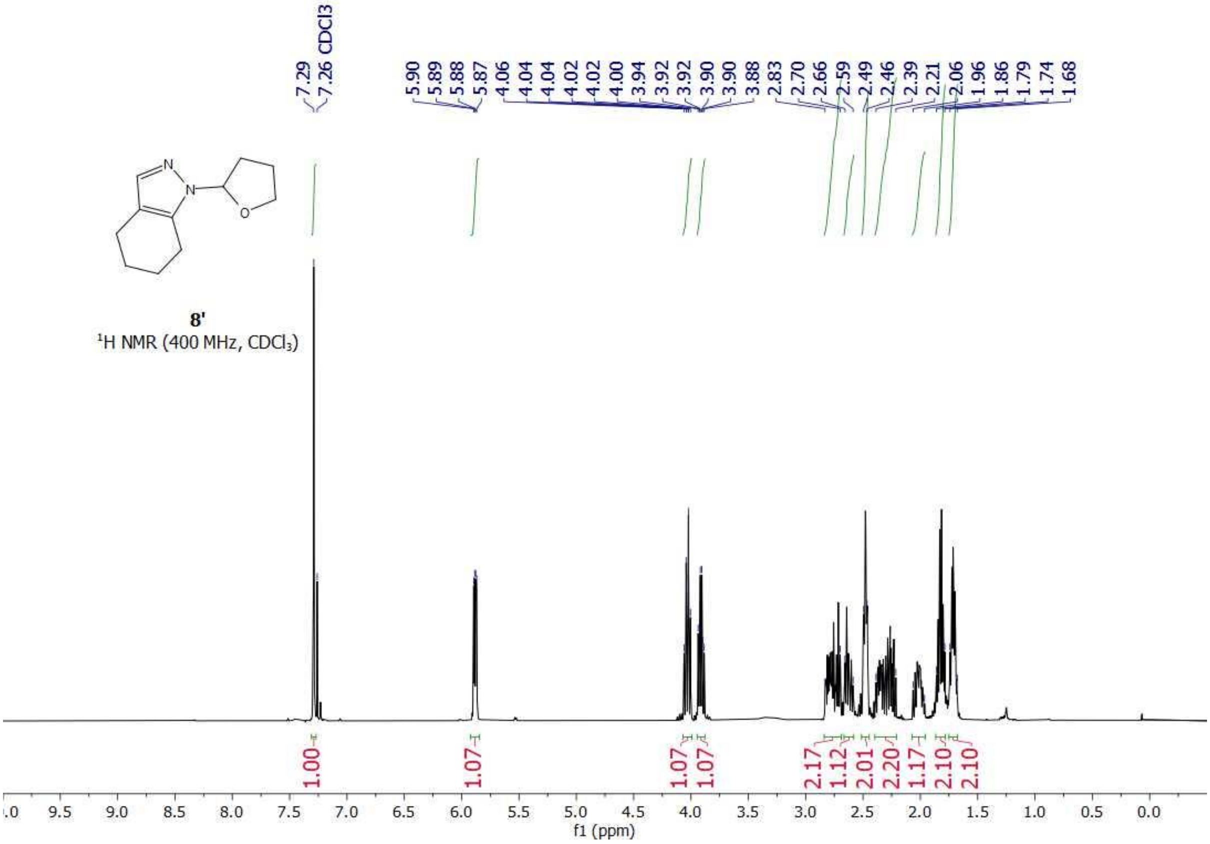
7.29
7.26 CDCl3
5.90
5.89
5.88
5.87
4.06
4.04
4.04
4.02
4.02
4.00
3.94
3.92
3.92
3.90
3.90
3.88
2.83
2.70
2.66
2.59
2.49
2.46
2.39
2.21
2.06
1.96
1.86
1.79
1.74
1.68
N
N
O
8'
^{1}H NMR (400 MHz, CDCl$_3$)
1.00
1.07
1.07
1.07
2.17
1.12
2.01
2.20
1.17
2.10
2.10
f1 (ppm)

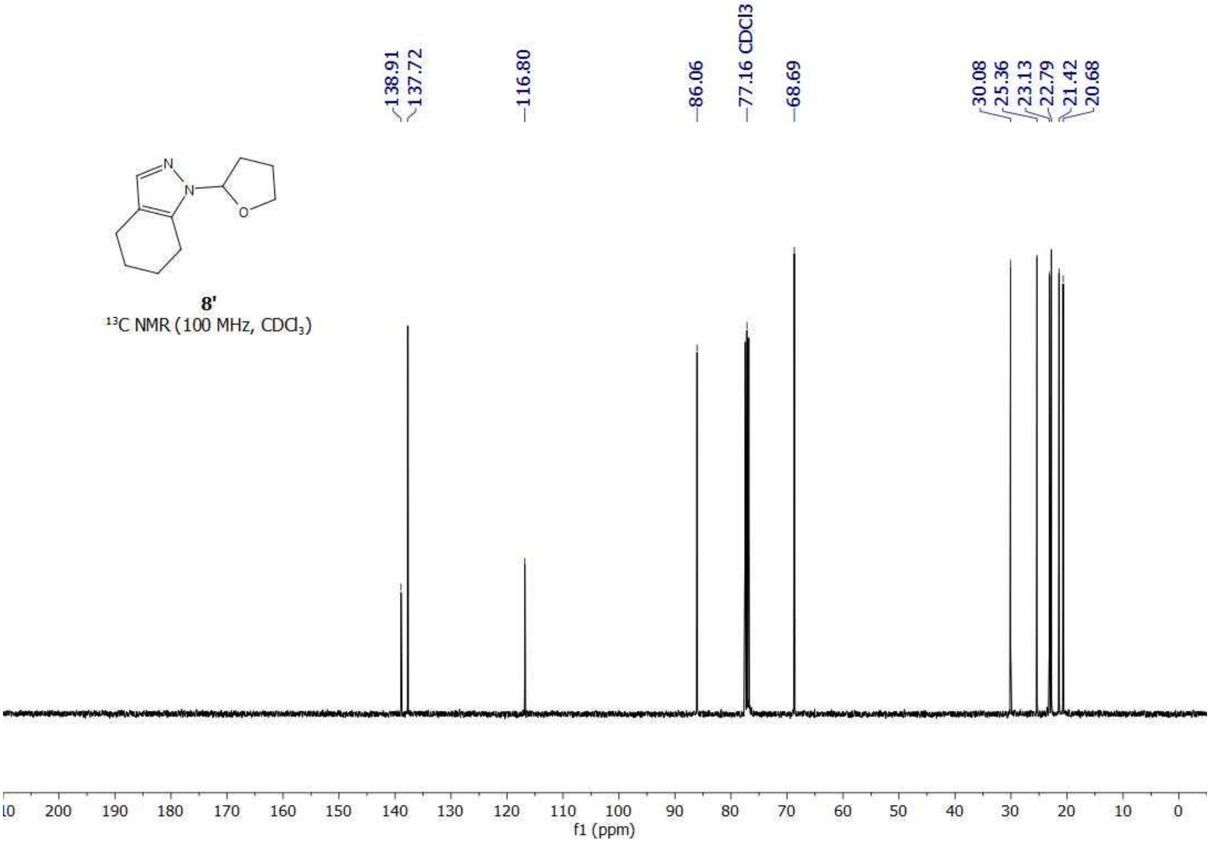
138.91
137.72
116.80
86.06
77.16 CDCl3
68.69
30.08
25.36
23.13
22.79
21.42
20.68
N
N
O
8'
^{13}C NMR (100 MHz, CDCl$_3$)
f1 (ppm)

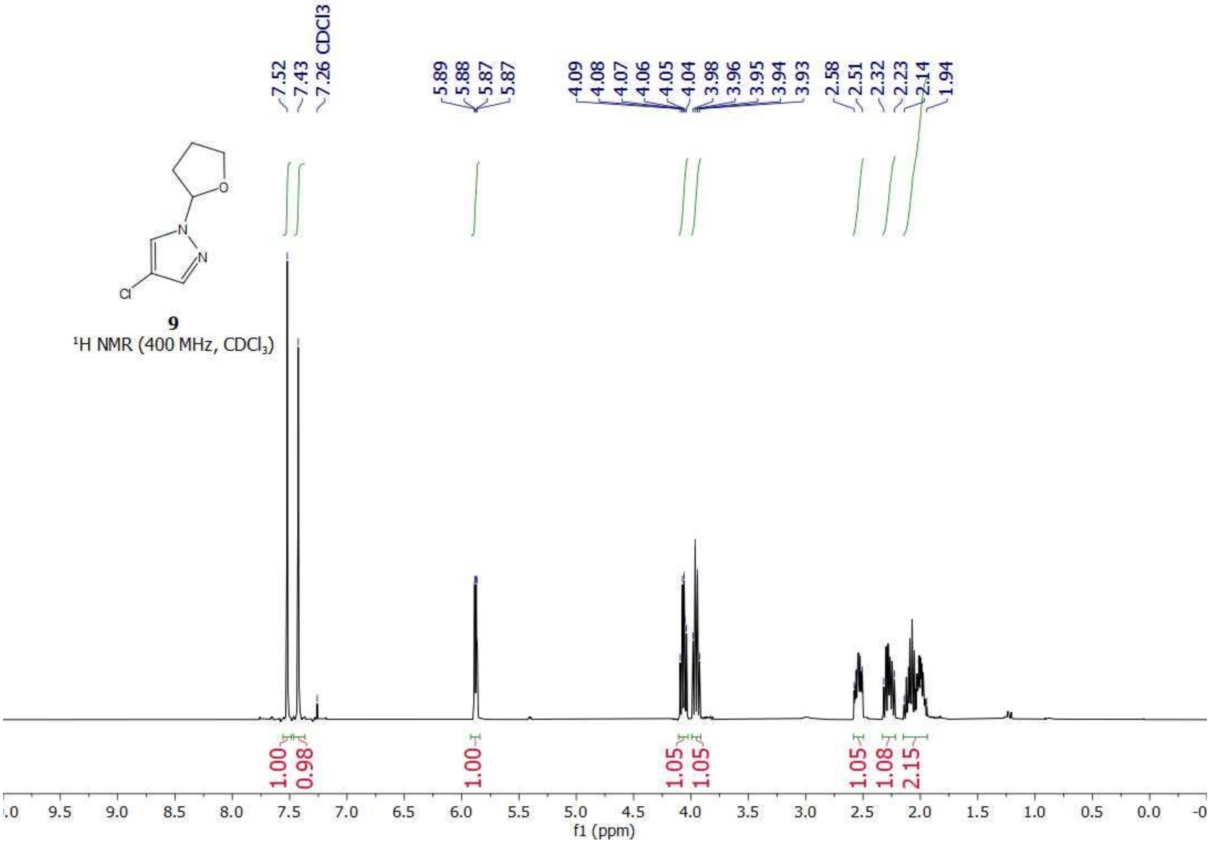

9
^{1}H NMR (400 MHz, $CDCl_3$)

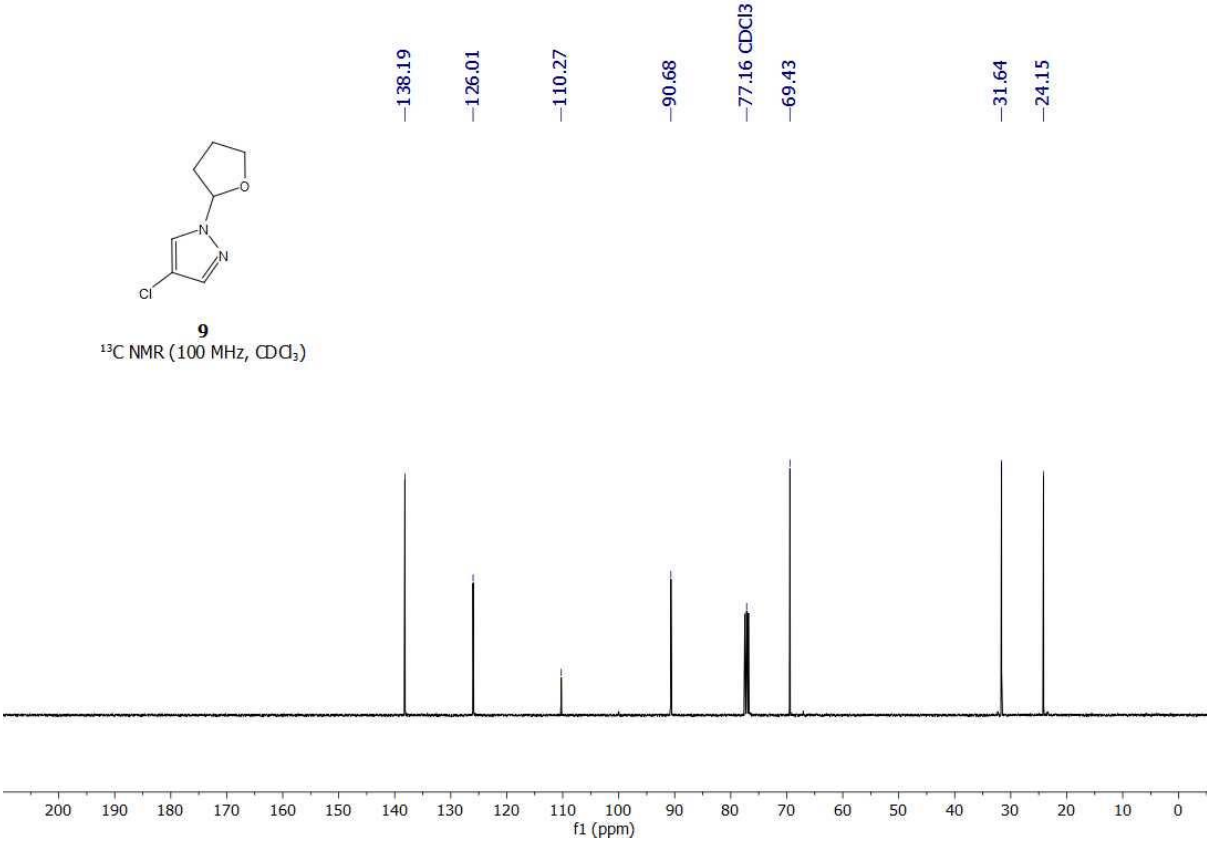

9
^{13}C NMR (100 MHz, $CDCl_3$)

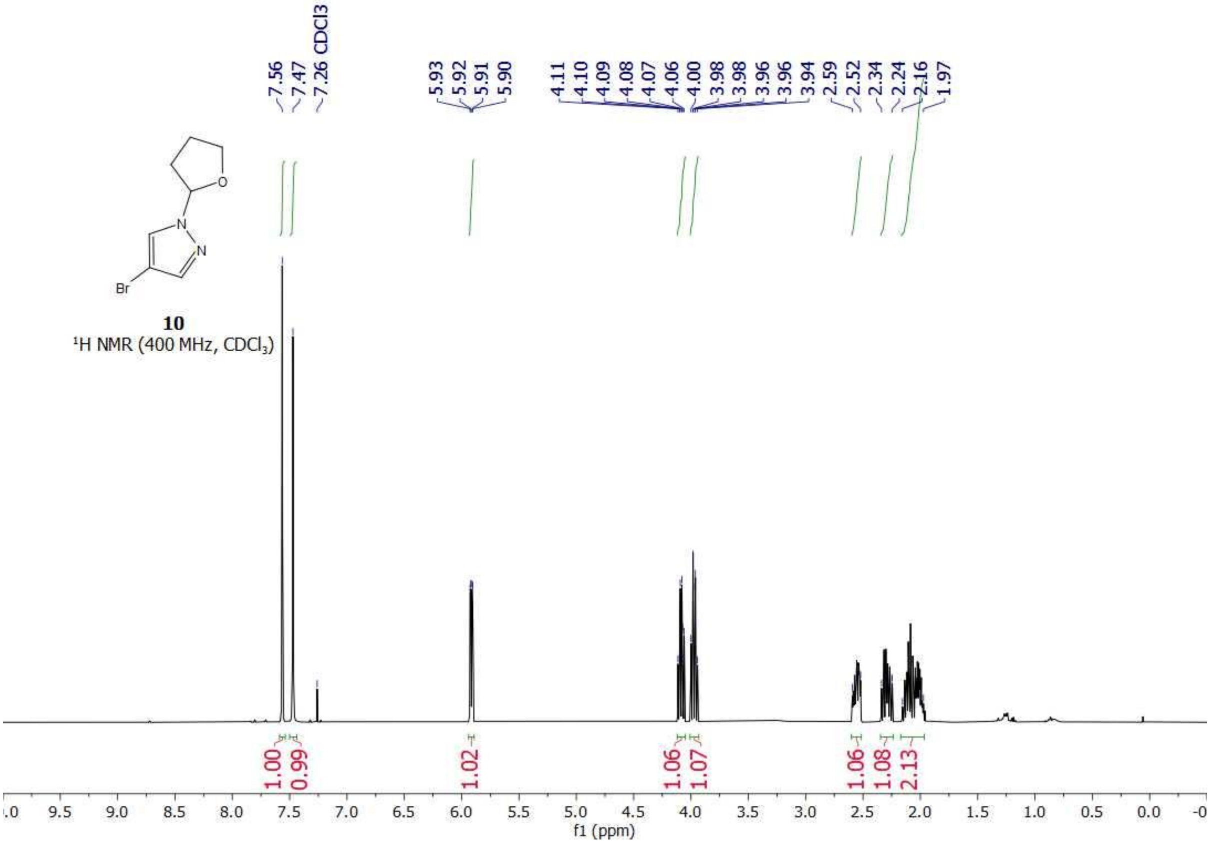
7.56
7.47
7.26 CDCl3
5.93
5.92
5.91
5.90
4.11
4.10
4.09
4.08
4.07
4.06
4.00
3.98
3.98
3.96
3.96
3.94
2.59
2.52
2.34
2.24
2.16
1.97
Br
N
O
10
1H NMR (400 MHz, CDCl3)
1.00
0.99
1.02
1.06
1.07
1.06
1.08
2.13
f1 (ppm)

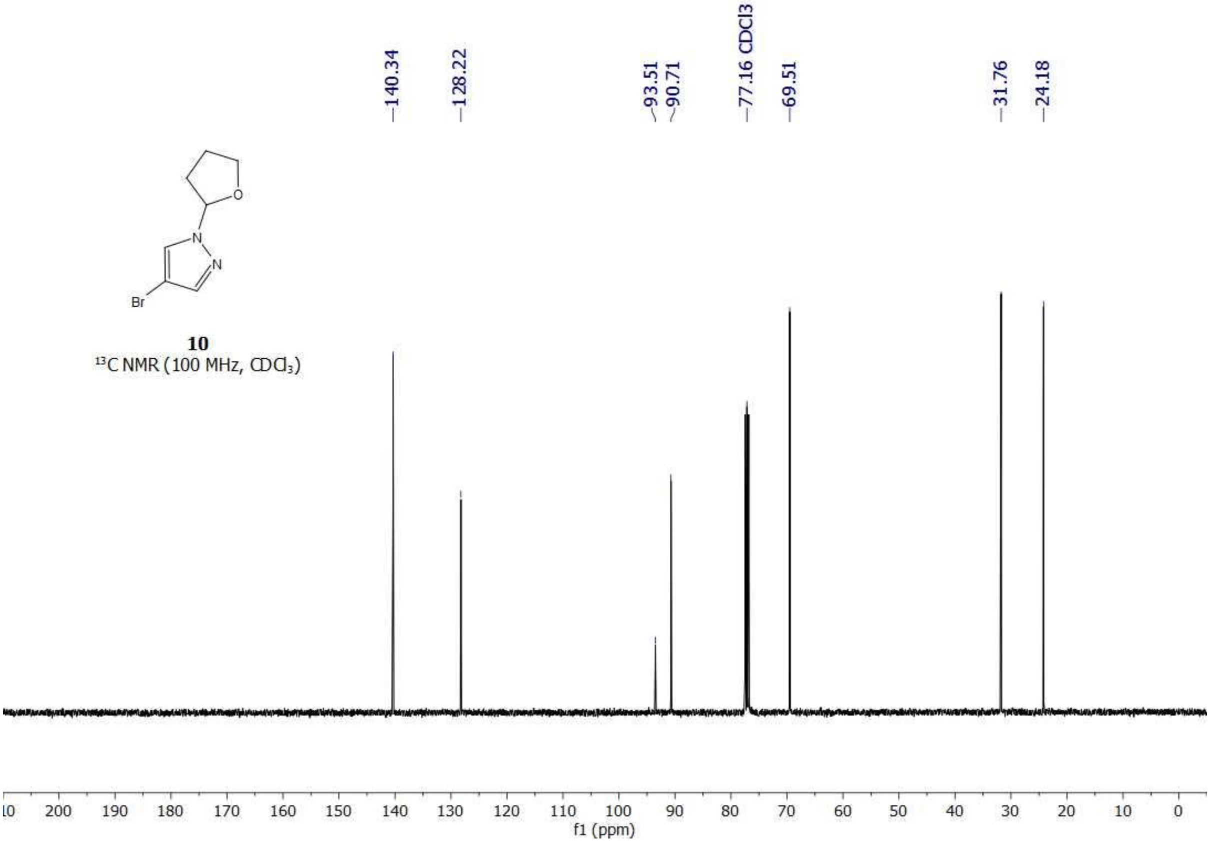
140.34
128.22
93.51
90.71
77.16 CDCl3
69.51
31.76
24.18
Br
N
O
10
13C NMR (100 MHz, CDCl3)
f1 (ppm)

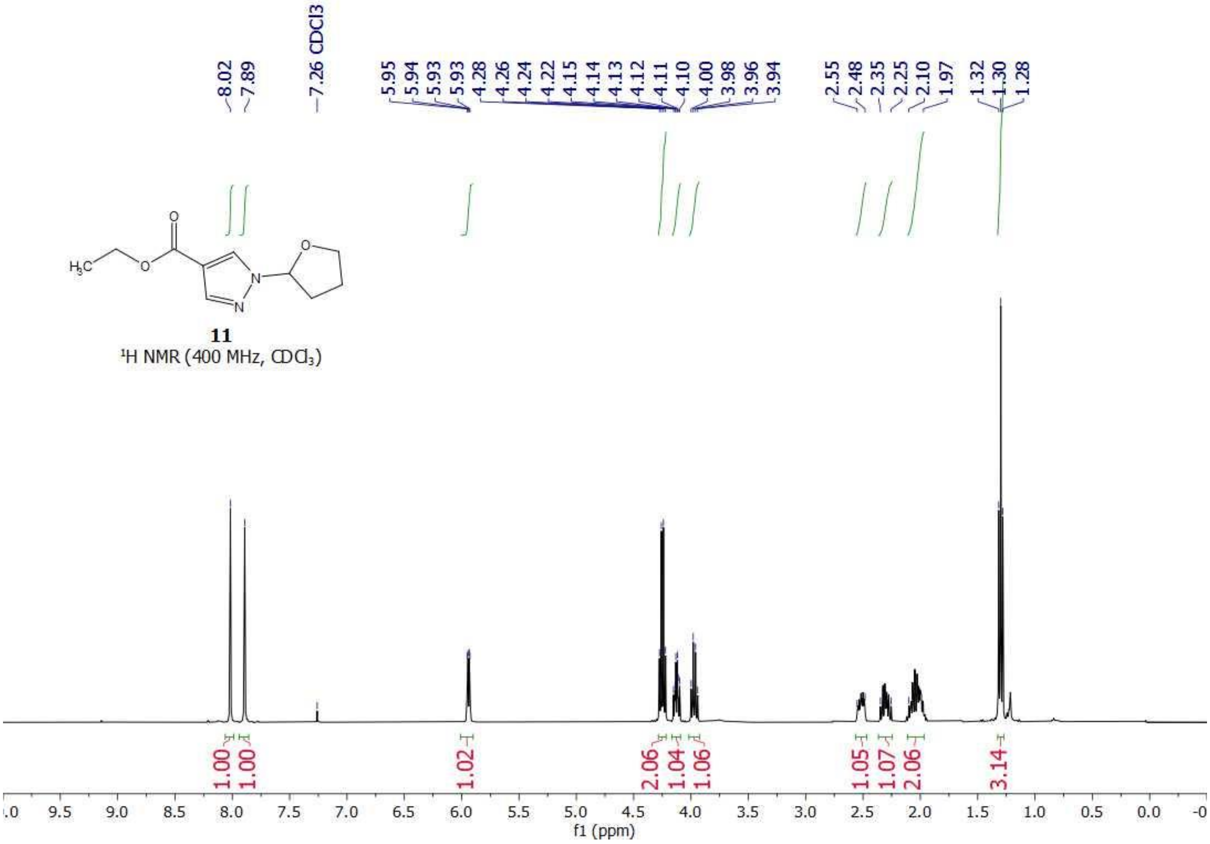

11
^{1}H NMR (400 MHz, $CDCl_3$)

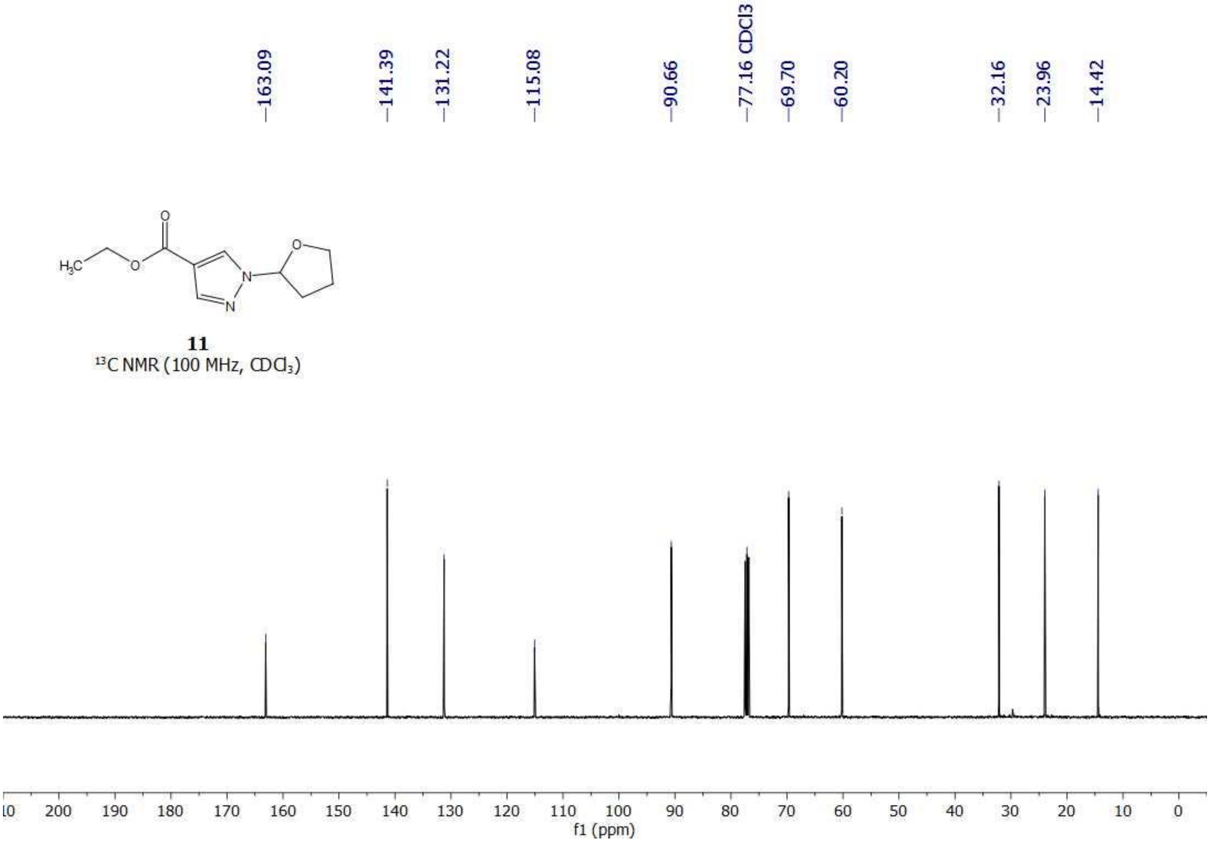

11
^{13}C NMR (100 MHz, $CDCl_3$)

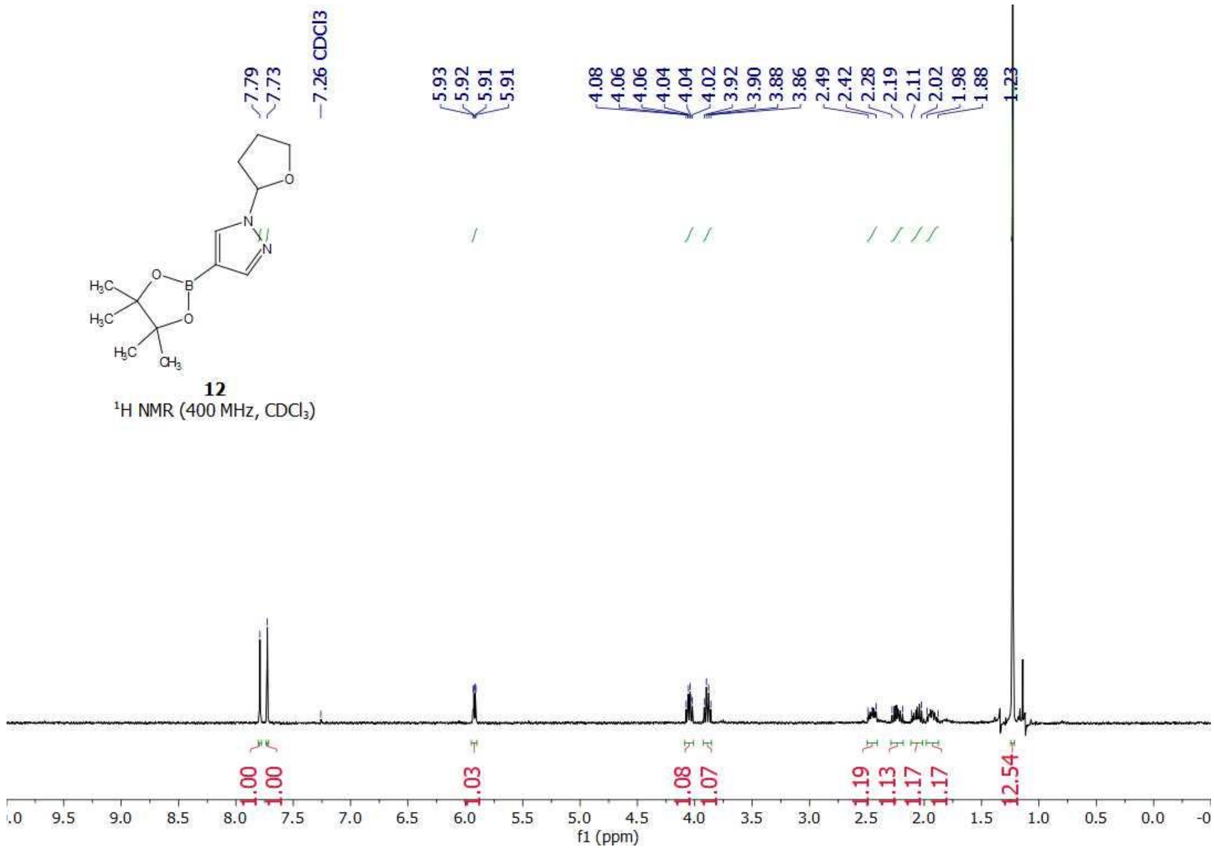

12
^{1}H NMR (400 MHz, $CDCl_3$)

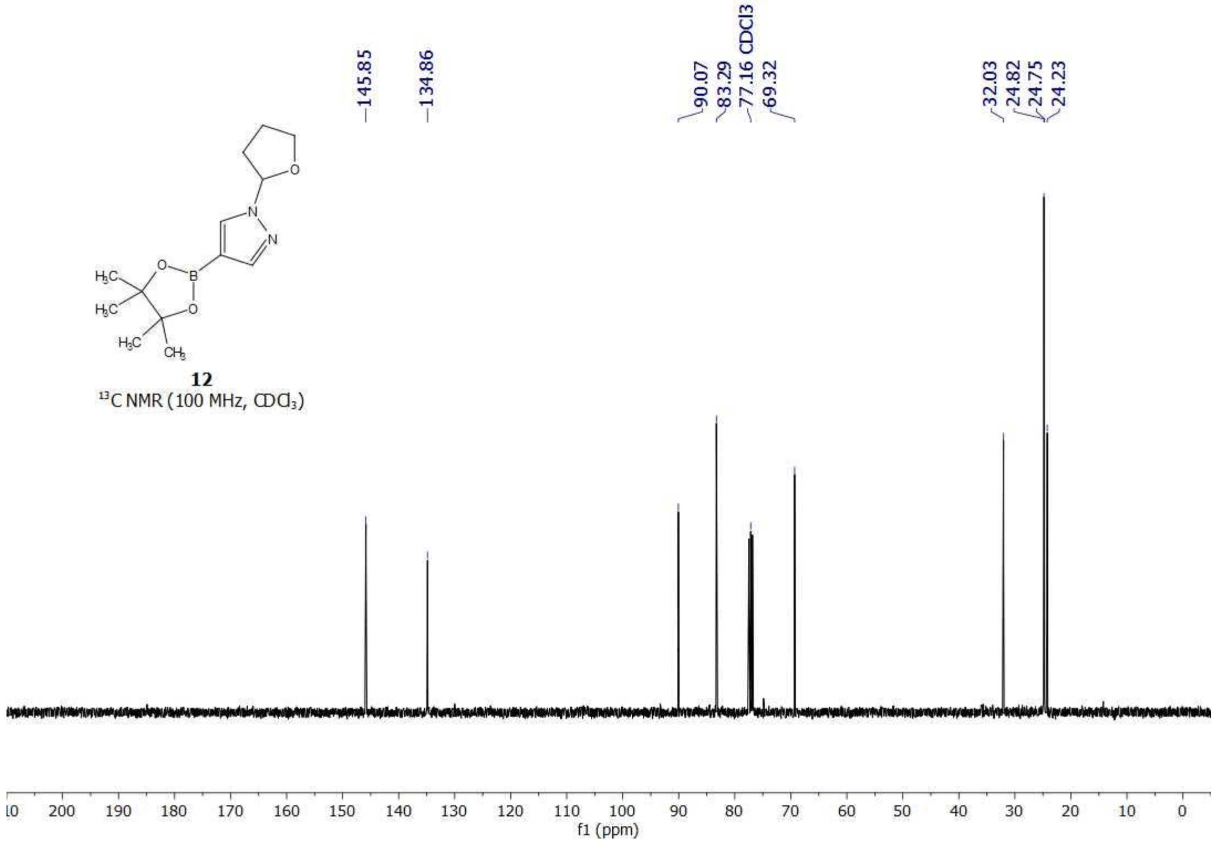

12
^{13}C NMR (100 MHz, $CDCl_3$)

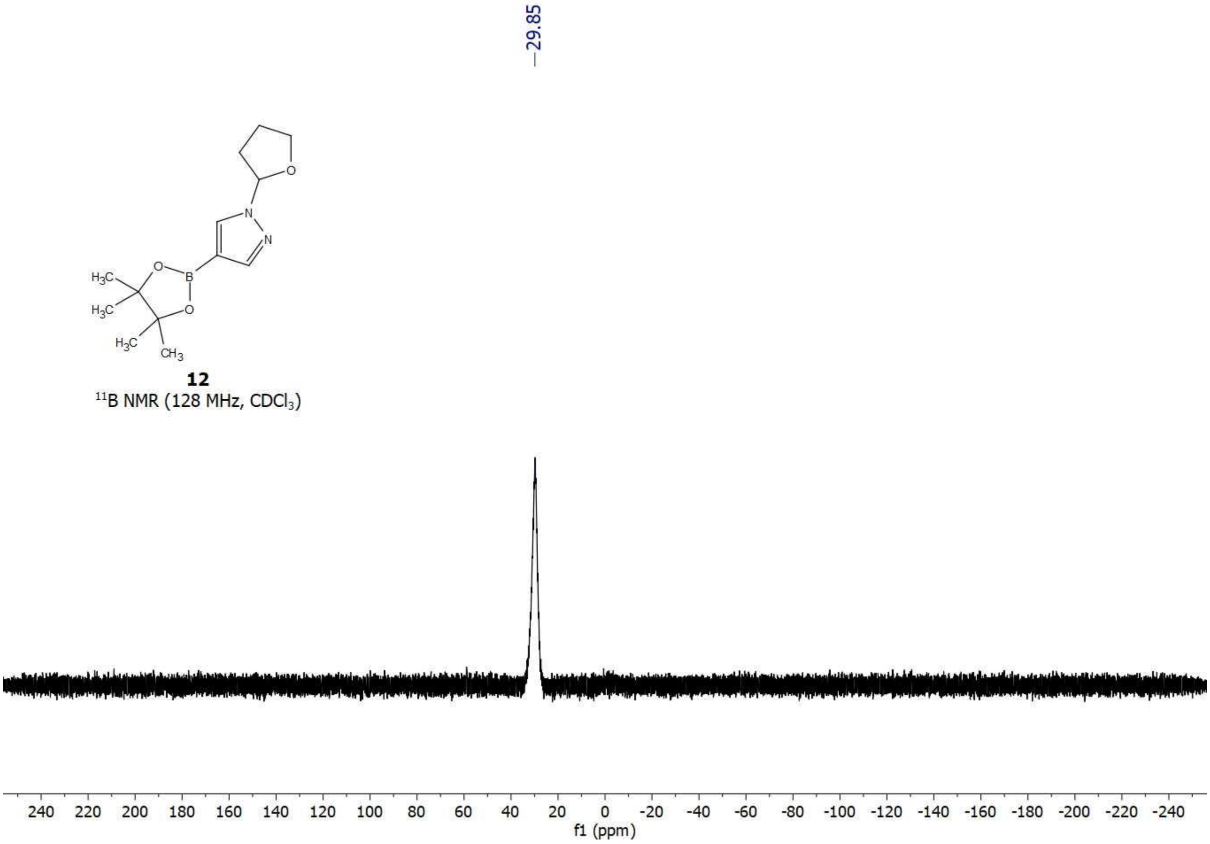

12

^{11}B NMR (128 MHz, $CDCl_3$)

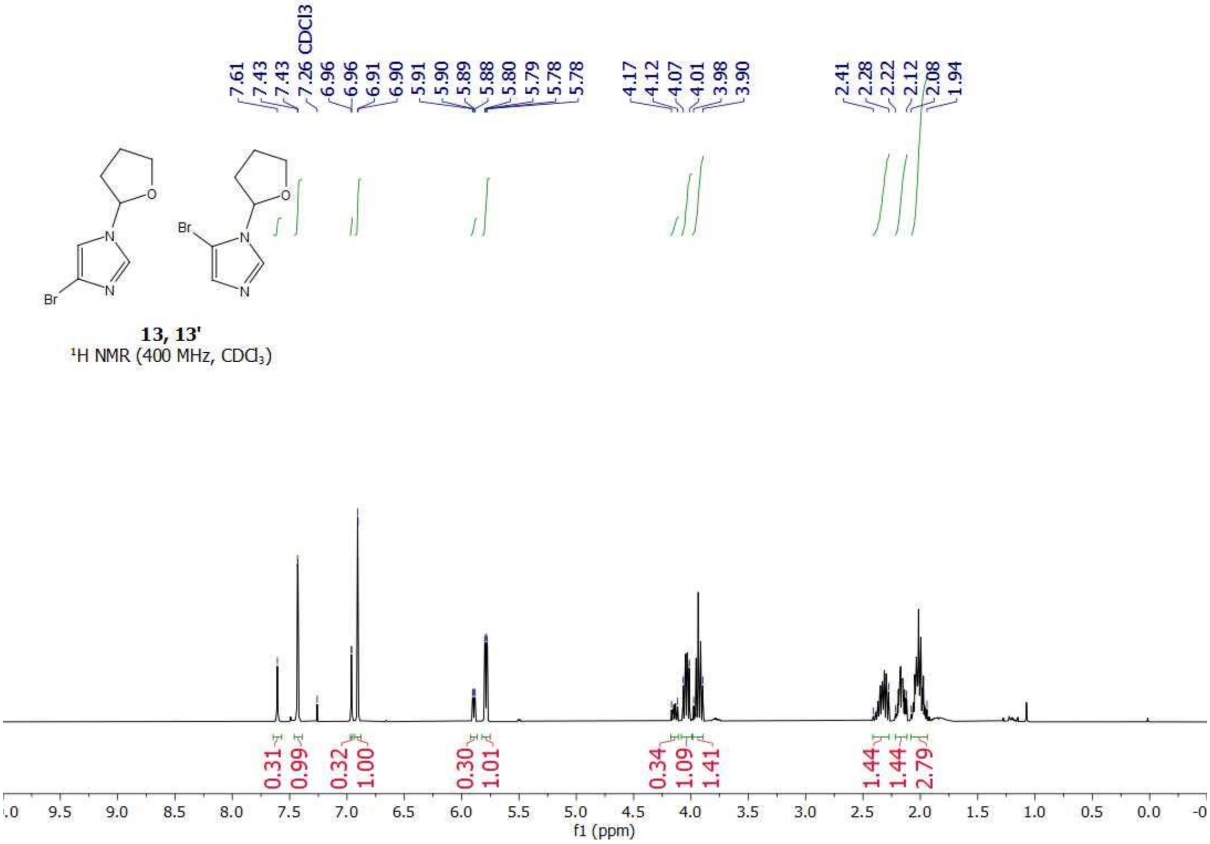

13, 13'
^{1}H NMR (400 MHz, $CDCl_3$)

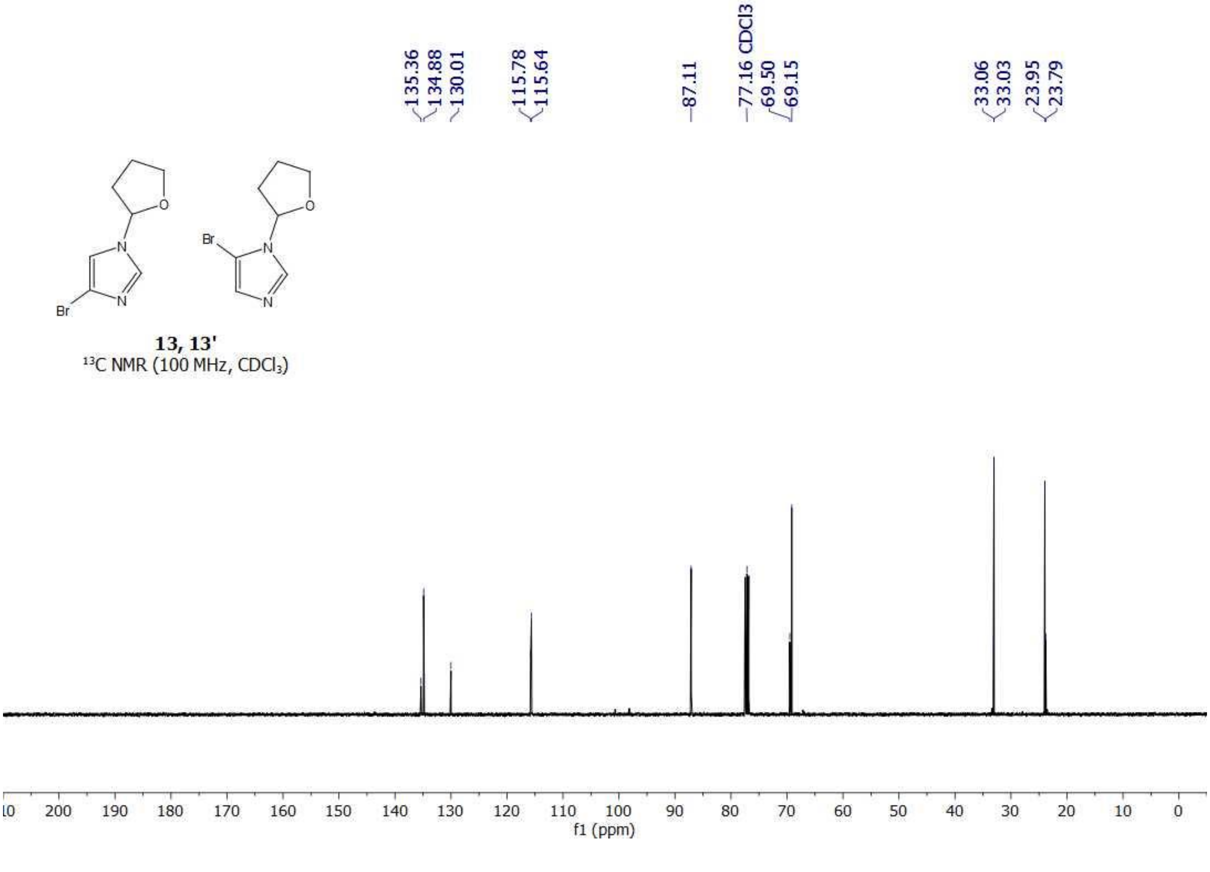

13, 13'
^{13}C NMR (100 MHz, $CDCl_3$)

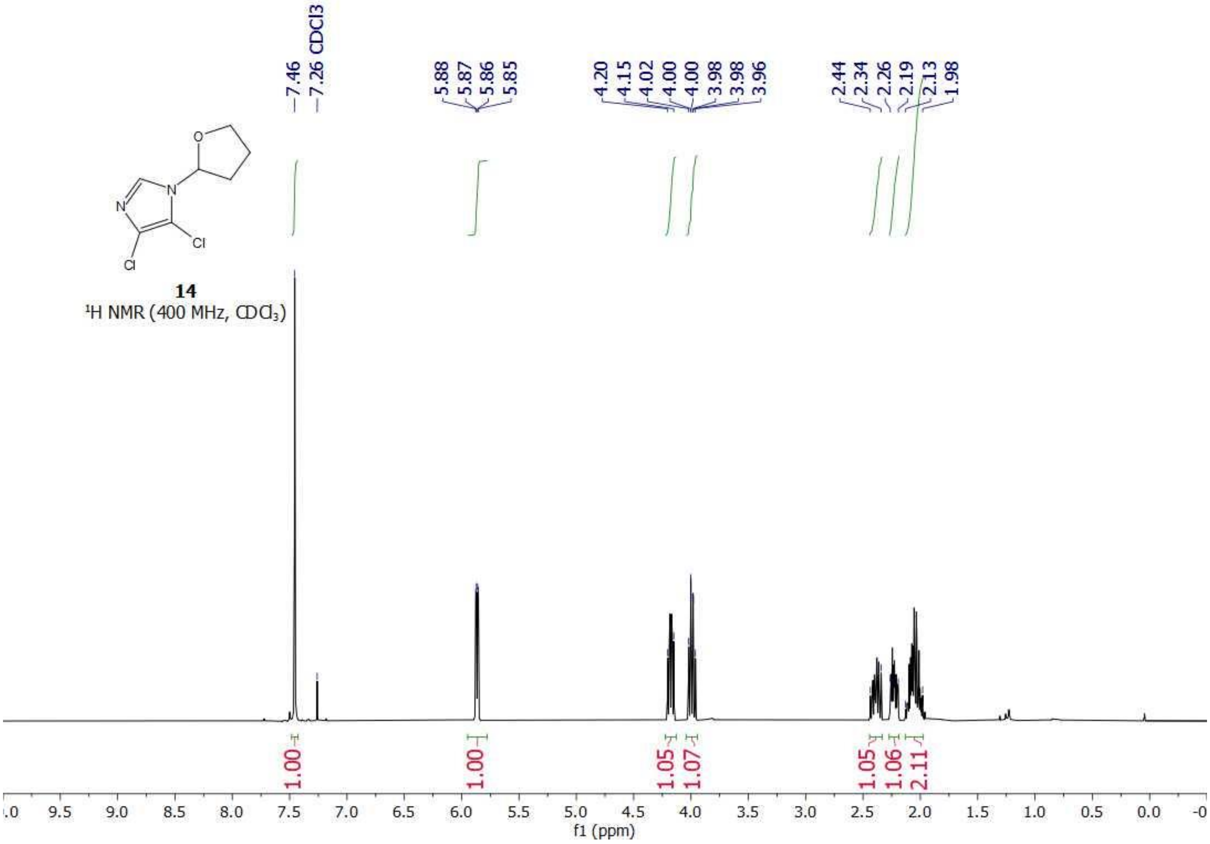

14
^{1}H NMR (400 MHz, $CDCl_3$)

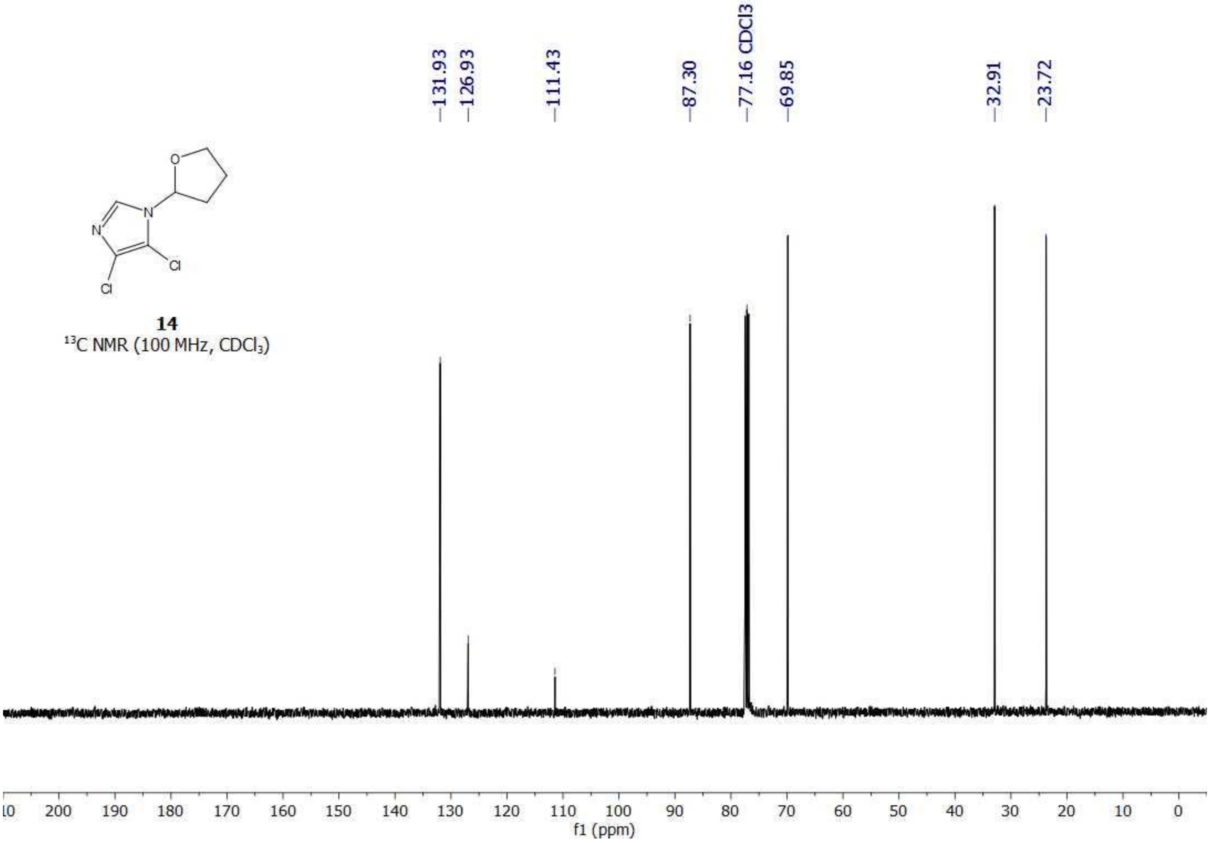

14
^{13}C NMR (100 MHz, $CDCl_3$)

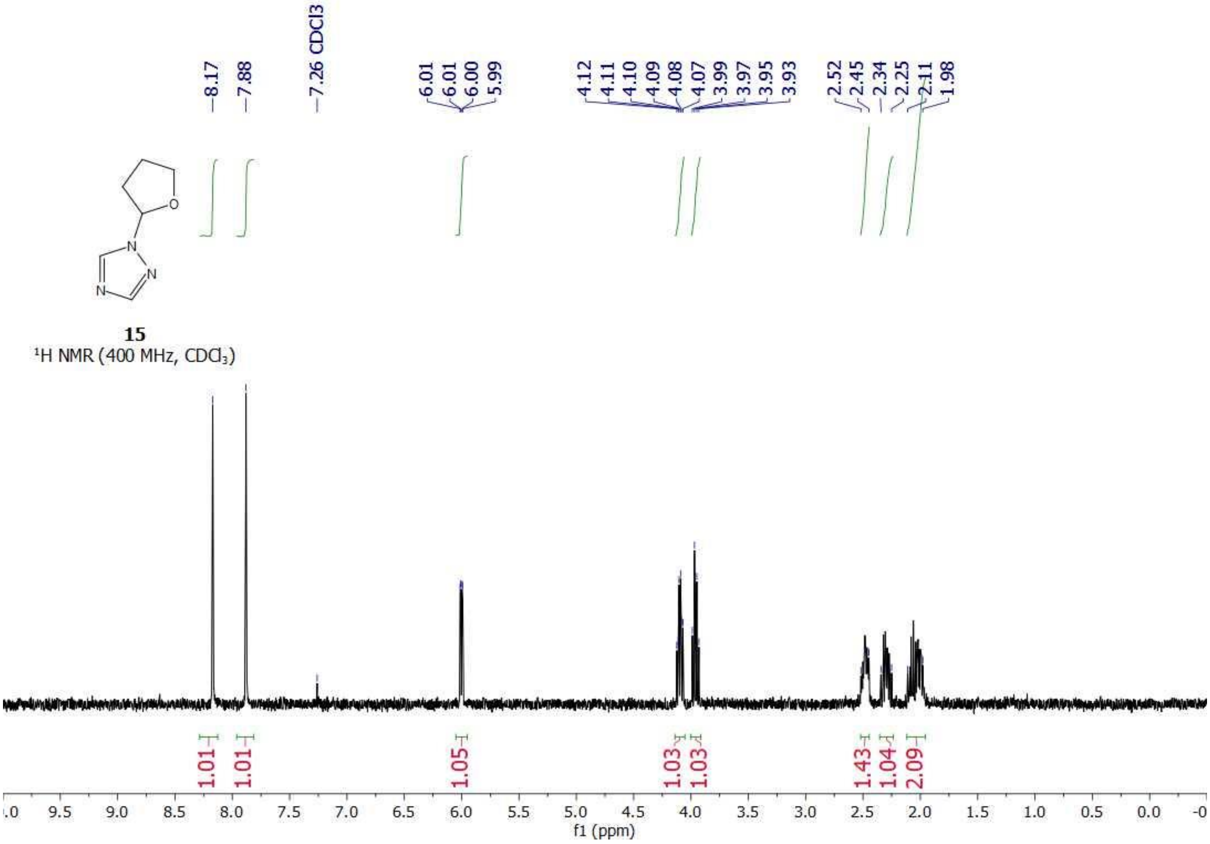

15
^{1}H NMR (400 MHz, $CDCl_3$)

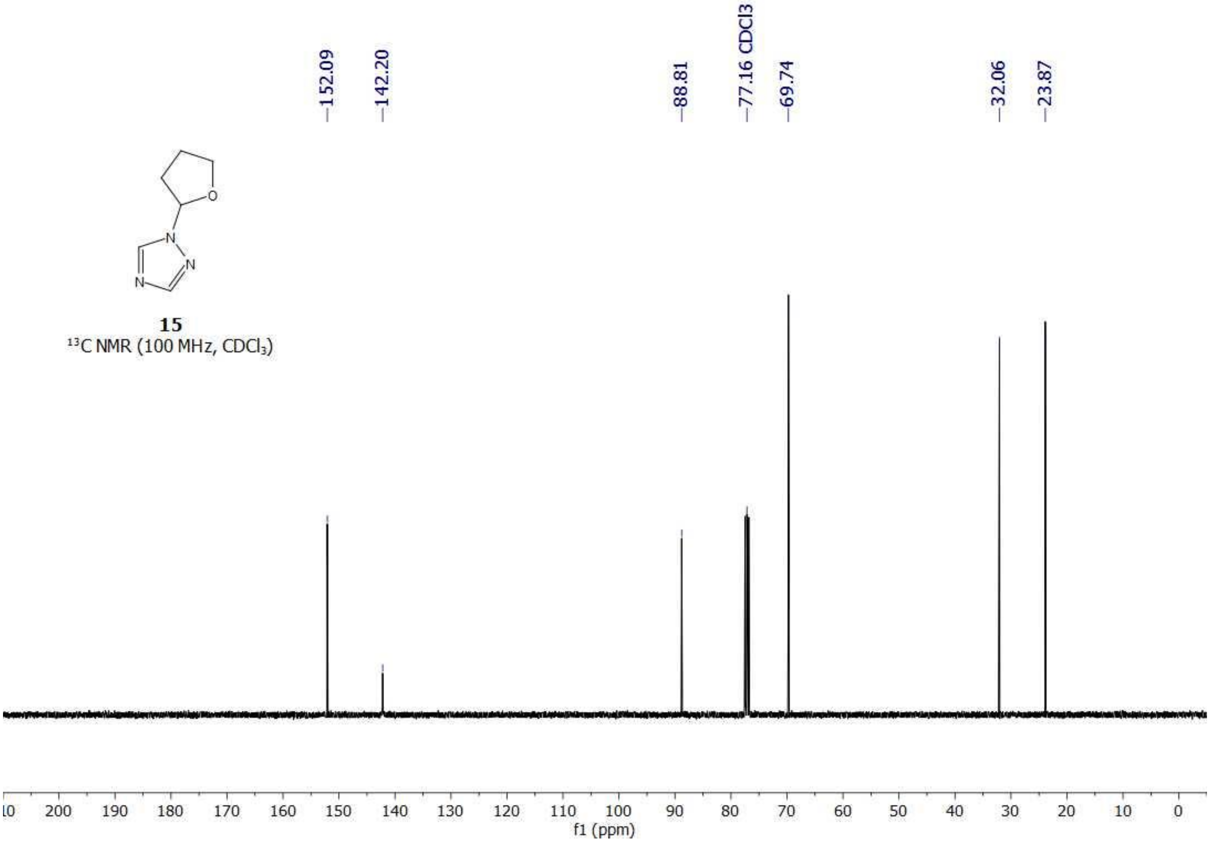

15
^{13}C NMR (100 MHz, $CDCl_3$)

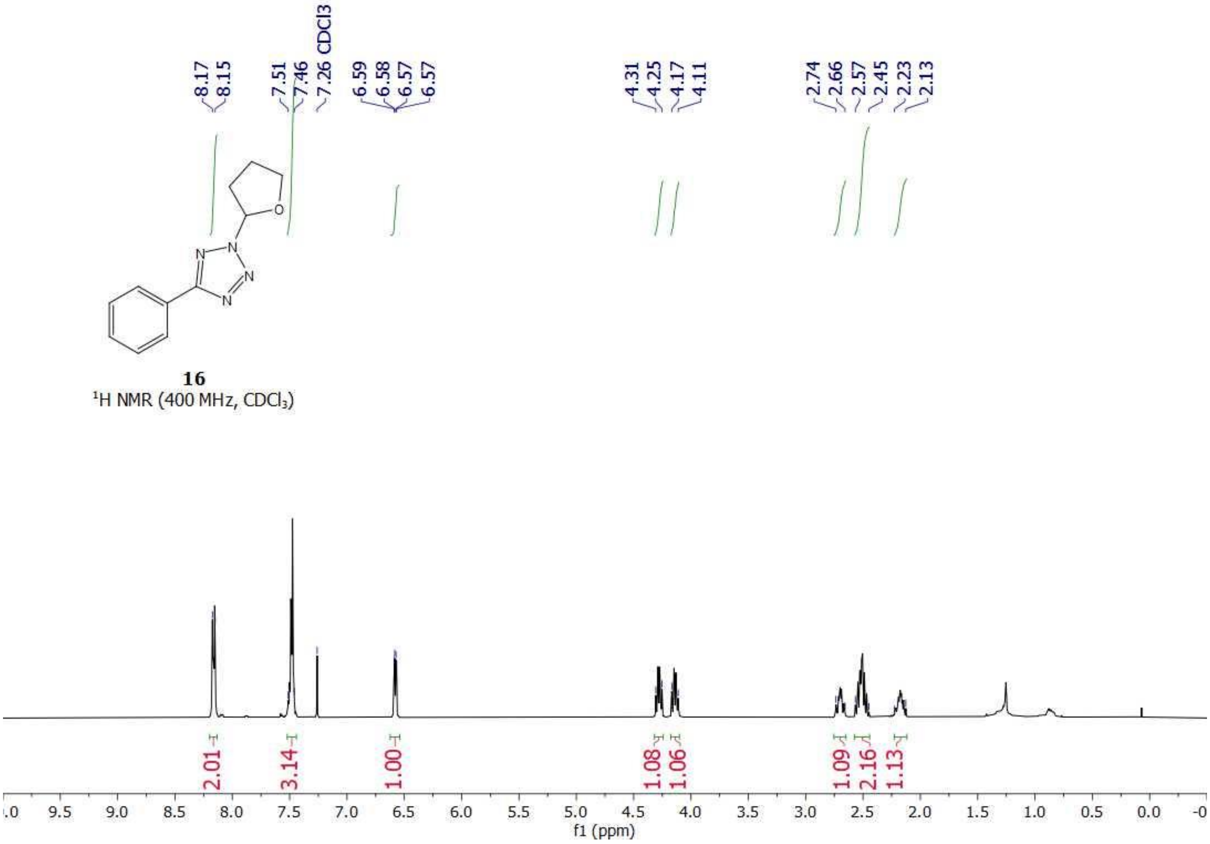

16
^{1}H NMR (400 MHz, $CDCl_3$)

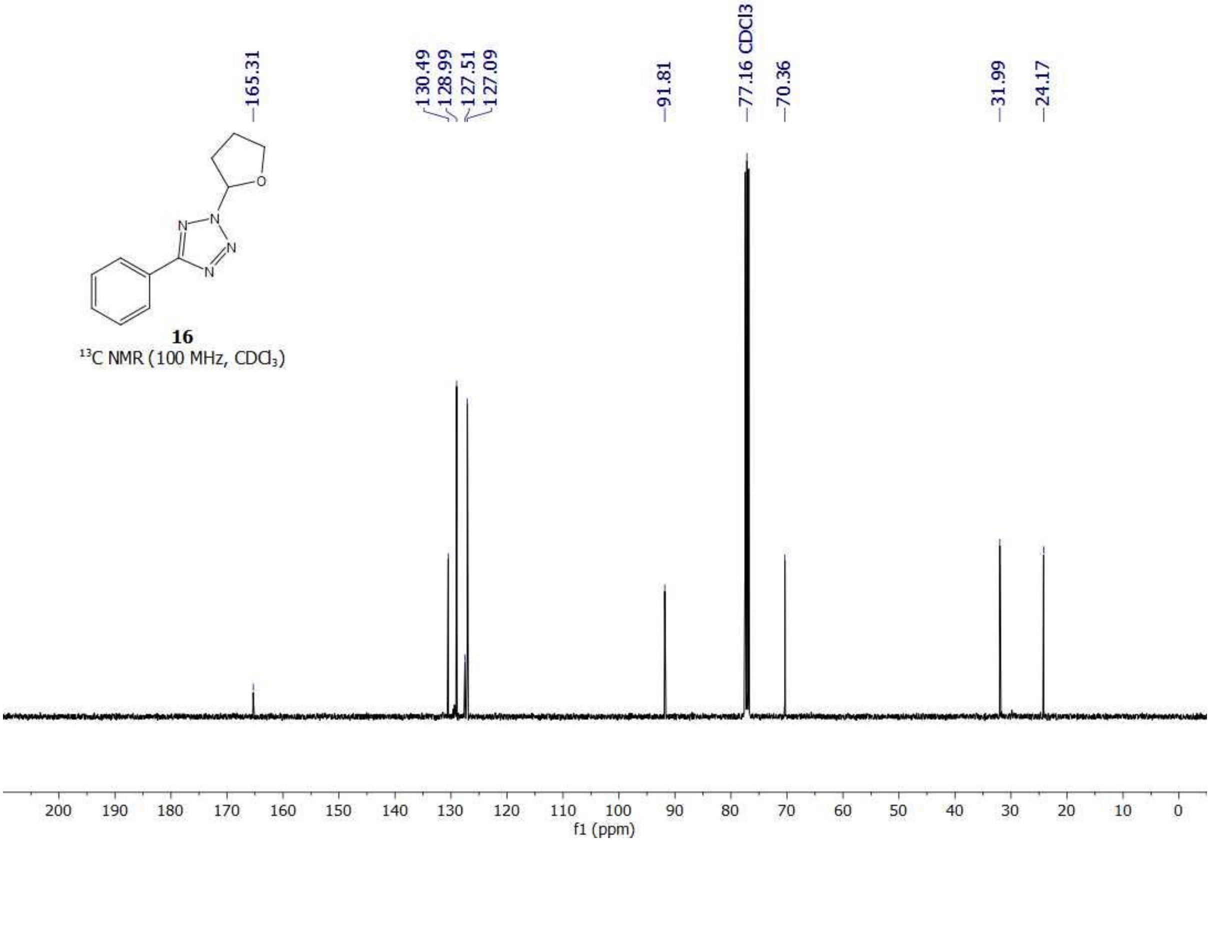

16
^{13}C NMR (100 MHz, $CDCl_3$)

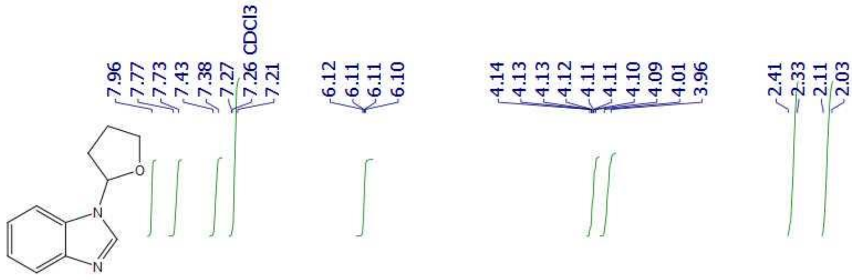

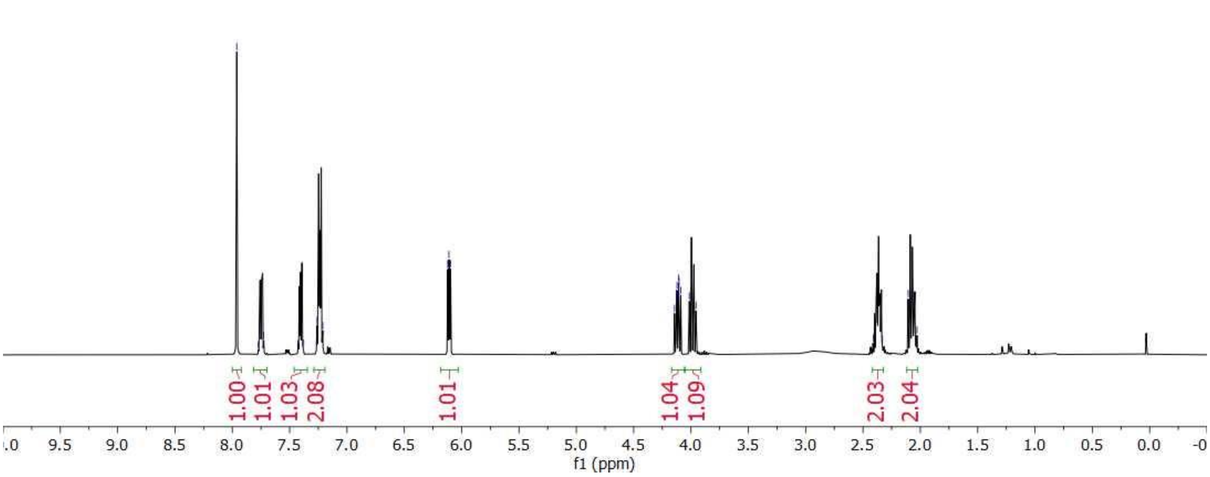

17
^1H NMR (400 MHz, $CDCl_3$)

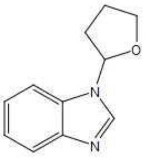

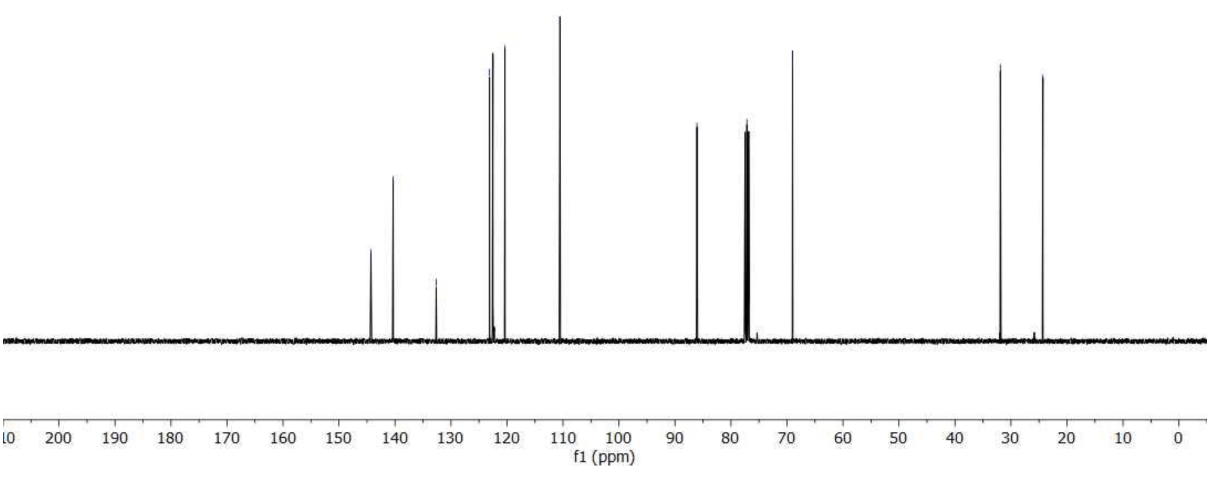

17
^{13}C NMR (100 MHz, $CDCl_3$)

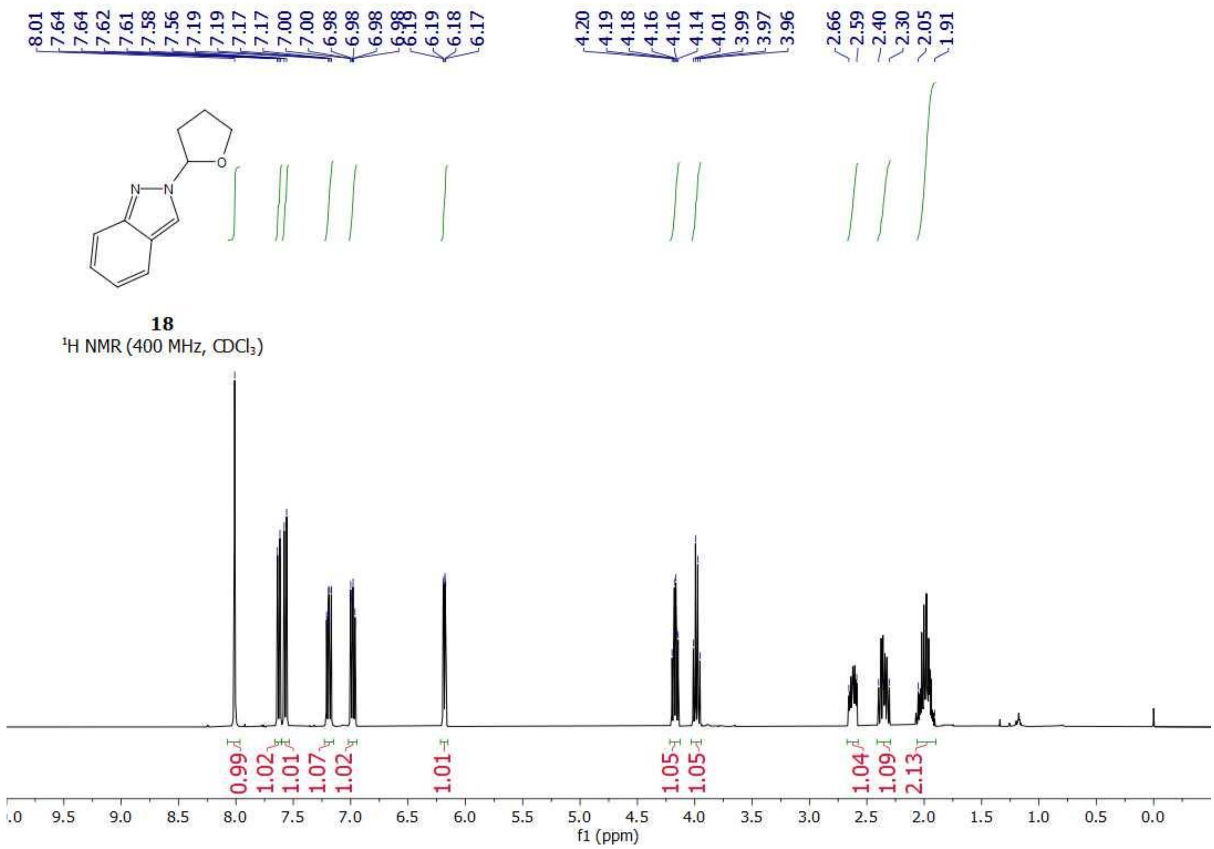

18
^{1}H NMR (400 MHz, $CDCl_3$)

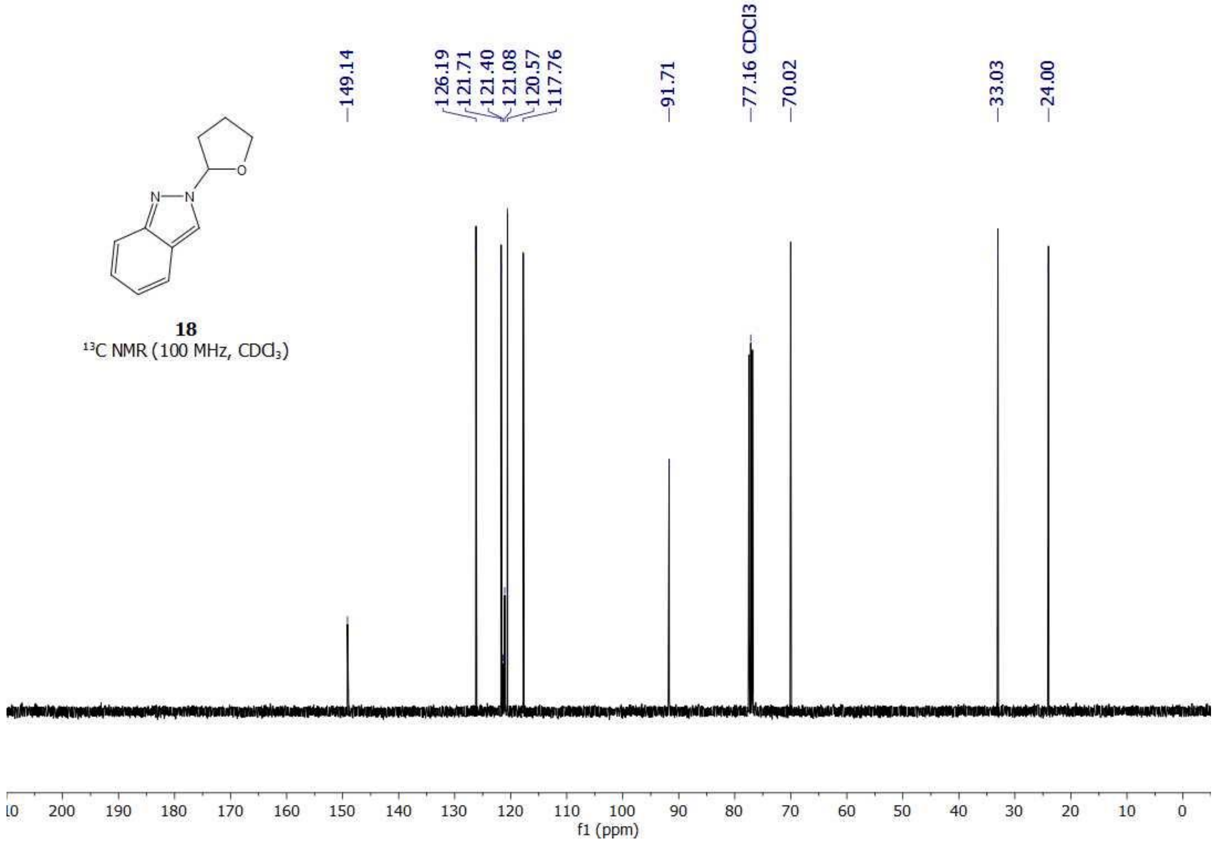

18
^{13}C NMR (100 MHz, $CDCl_3$)

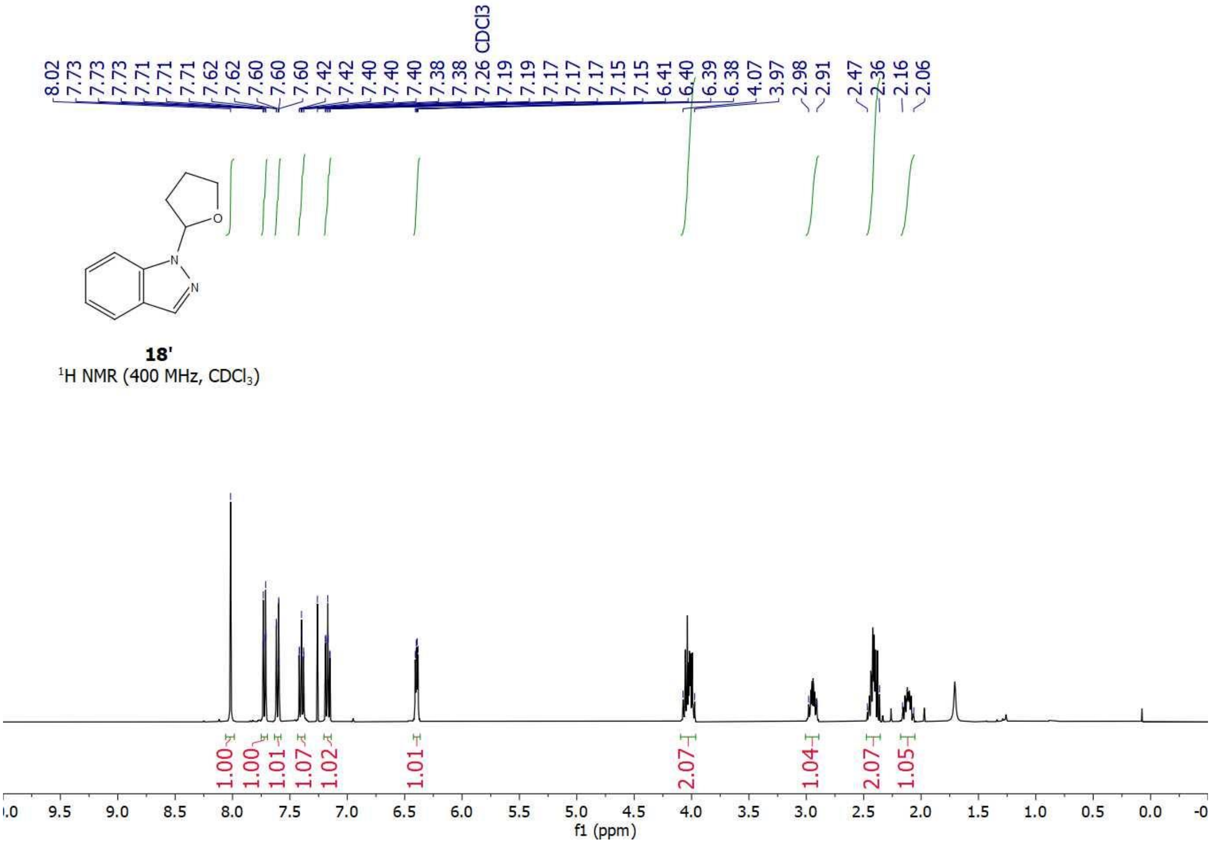

18'
^{1}H NMR (400 MHz, $CDCl_3$)

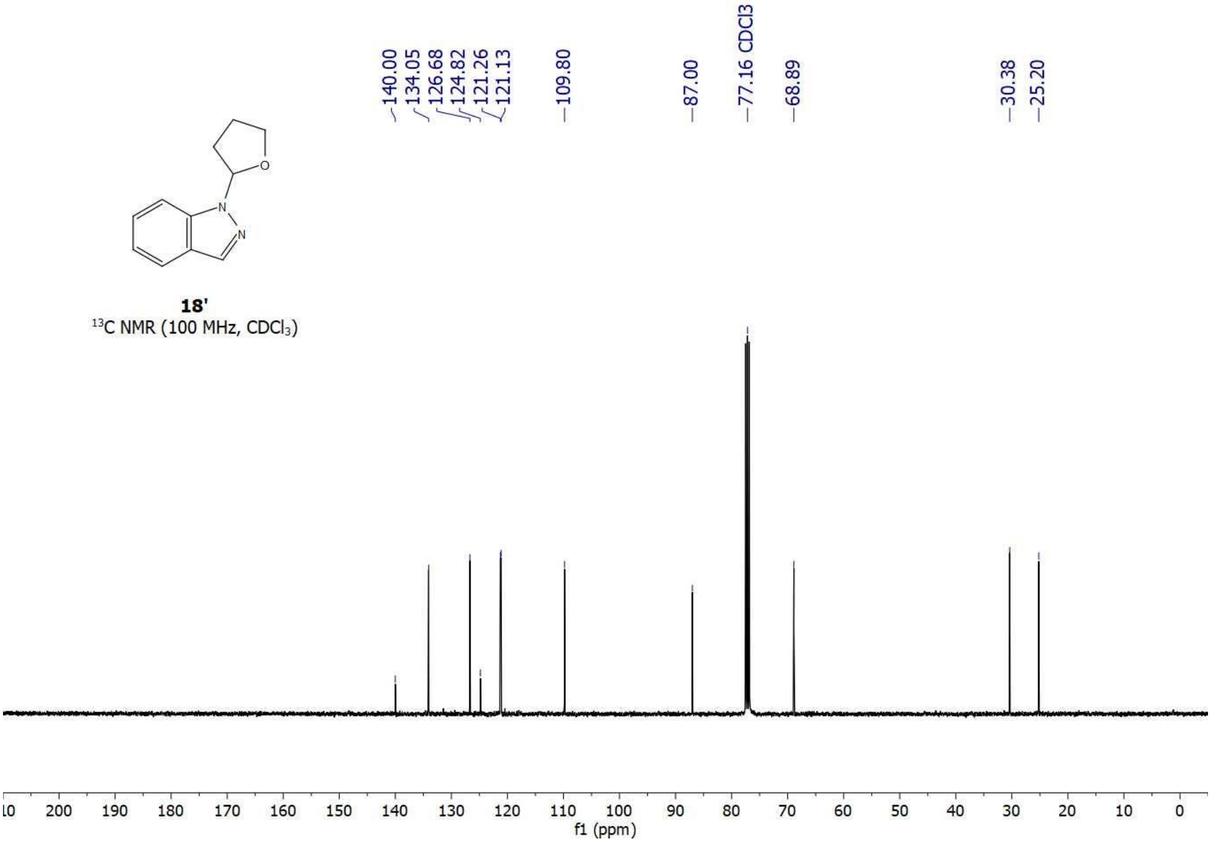

18'
^{13}C NMR (100 MHz, $CDCl_3$)

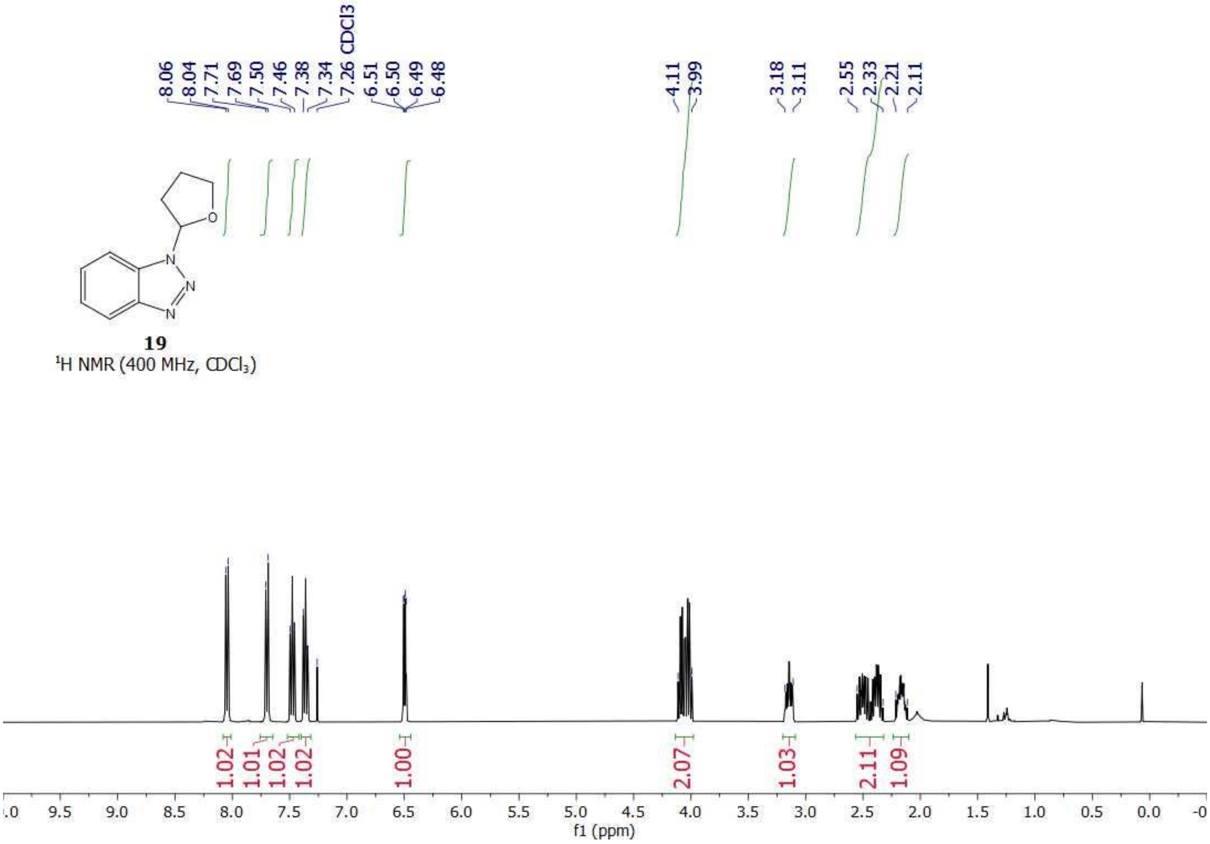

19
^{1}H NMR (400 MHz, $CDCl_3$)

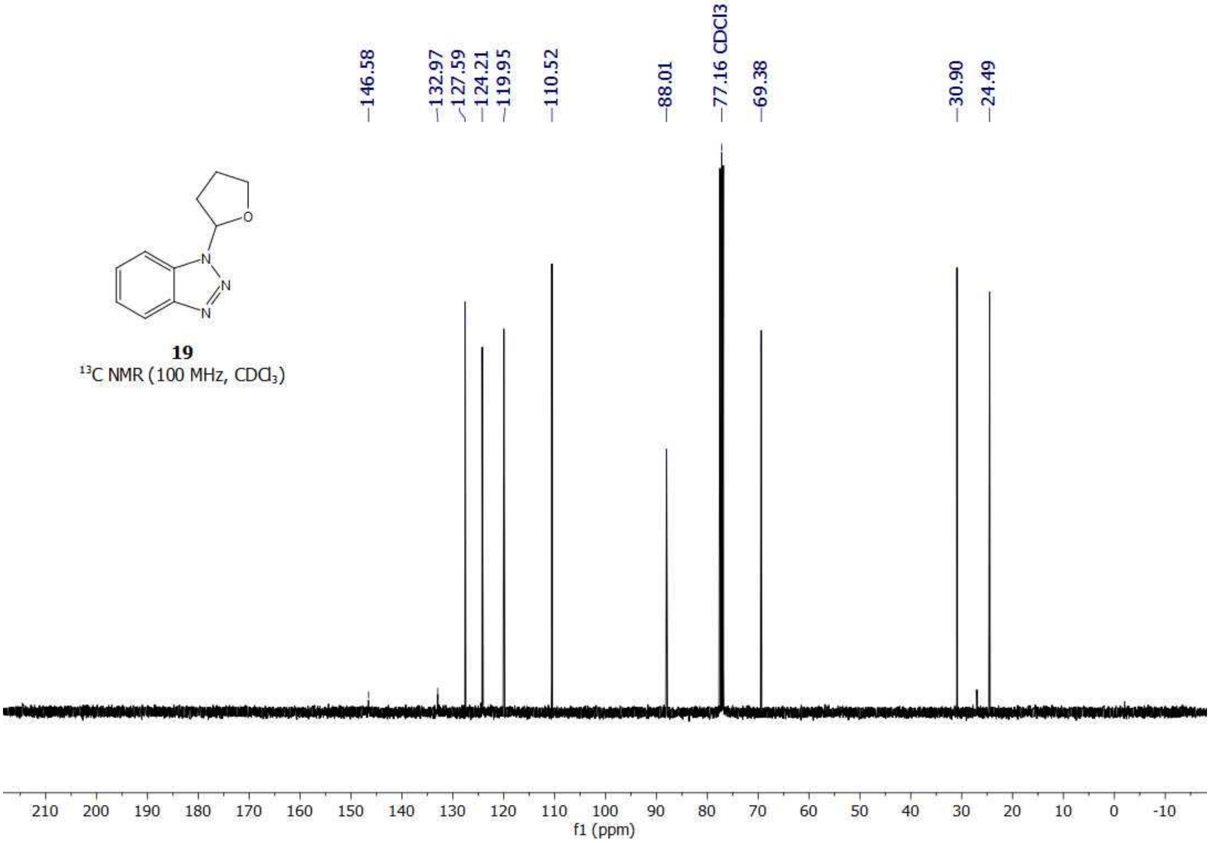

19
^{13}C NMR (100 MHz, $CDCl_3$)

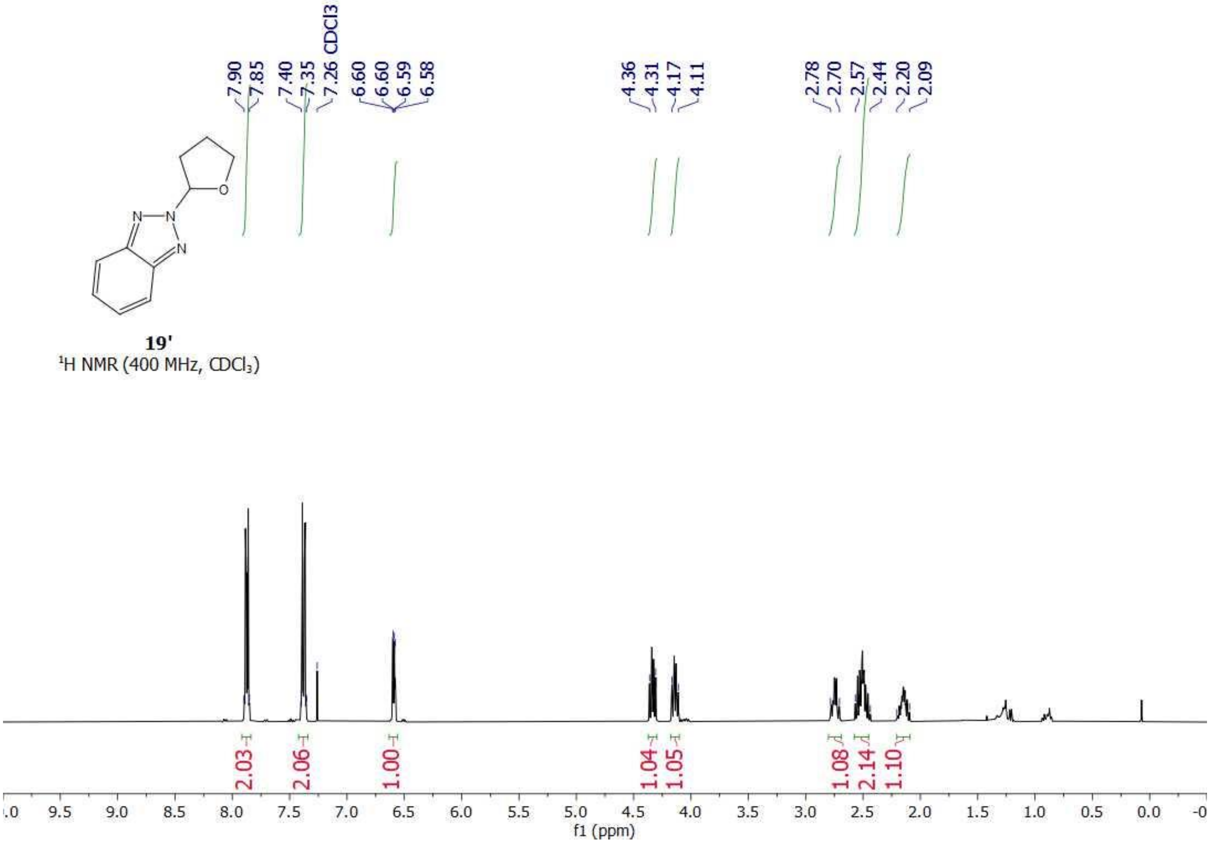
7.90
7.85
7.40
7.35
7.26 CDCl3
6.60
6.60
6.59
6.58
4.36
4.31
4.17
4.11
2.78
2.70
2.57
2.44
2.20
2.09
O
N
N
N
19'
1H NMR (400 MHz, CDCl3)
2.03
2.06
1.00
1.04
1.05
1.08
2.14
1.10
f1 (ppm)

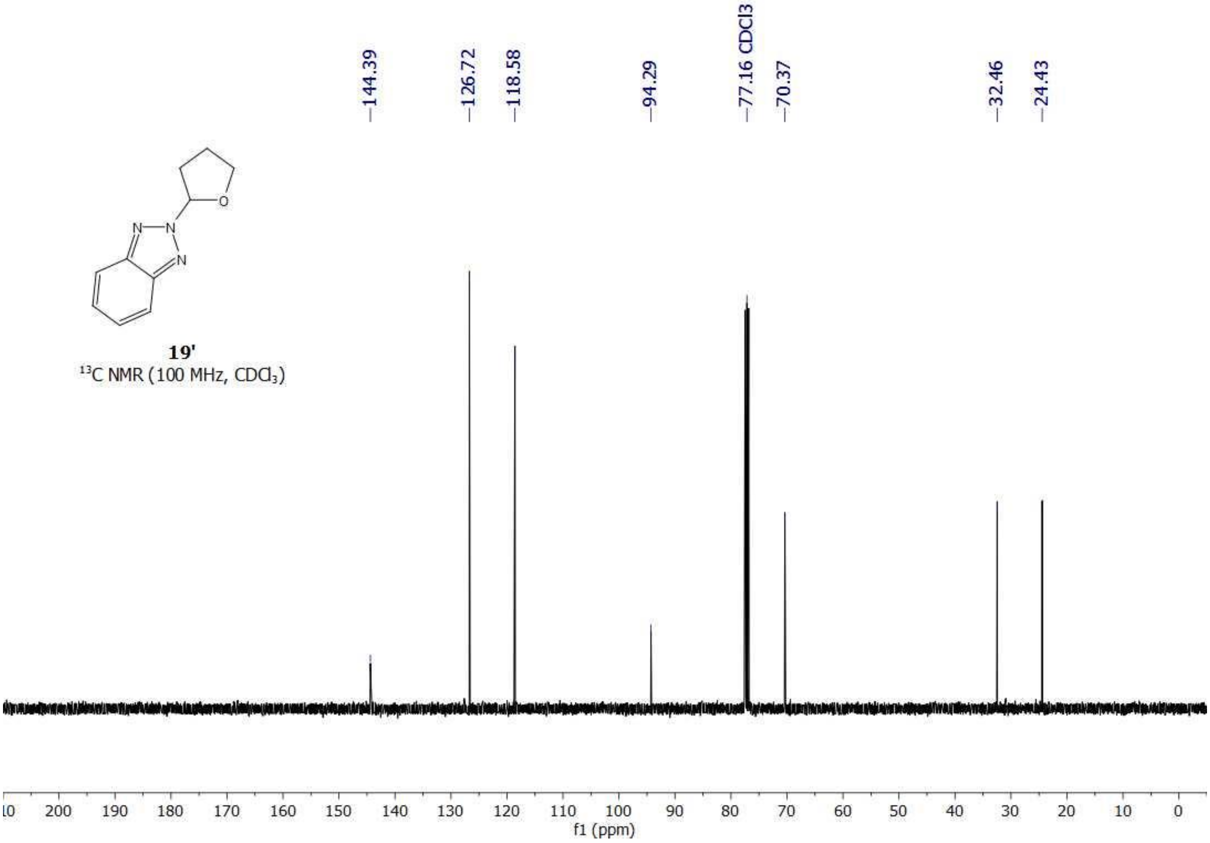
144.39
126.72
118.58
94.29
77.16 CDCl3
70.37
32.46
24.43
O
N
N
N
19'
13C NMR (100 MHz, CDCl3)
f1 (ppm)

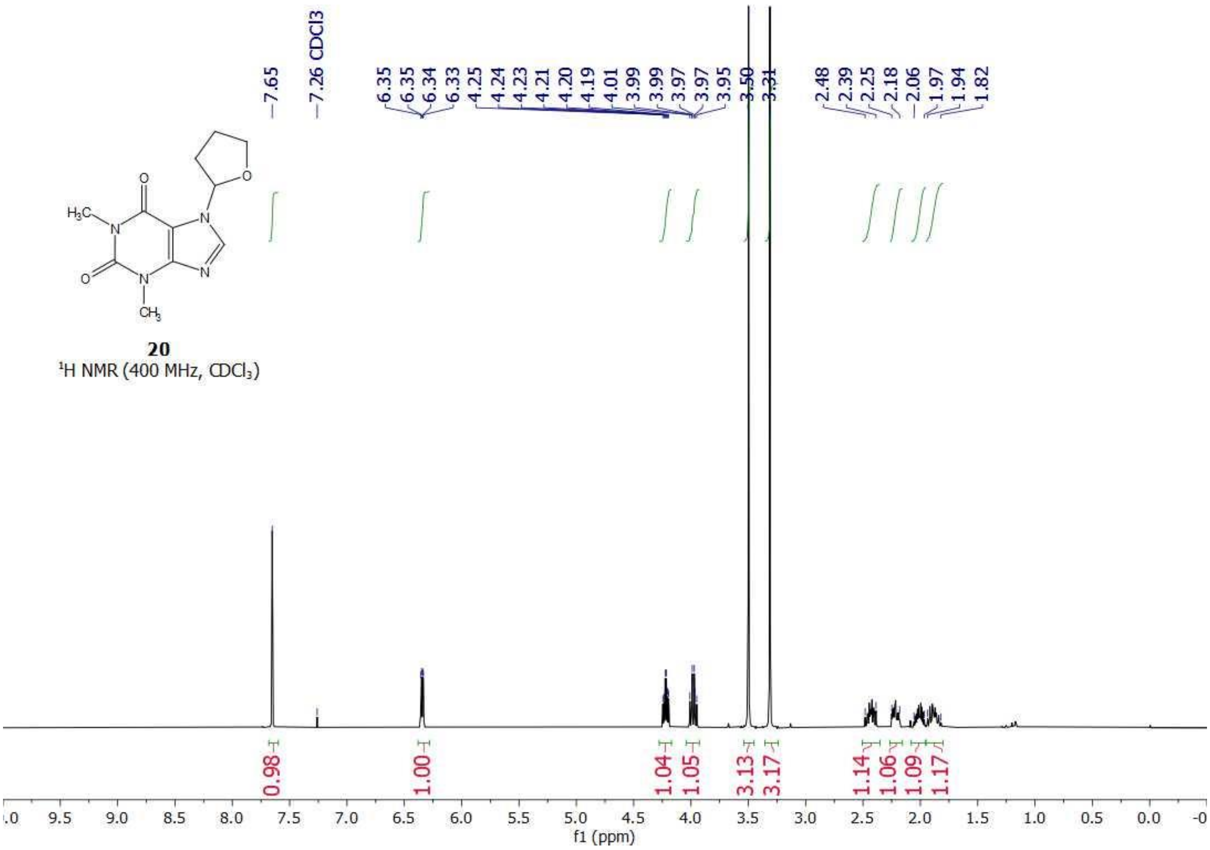

20

^{1}H NMR (400 MHz, $CDCl_3$)

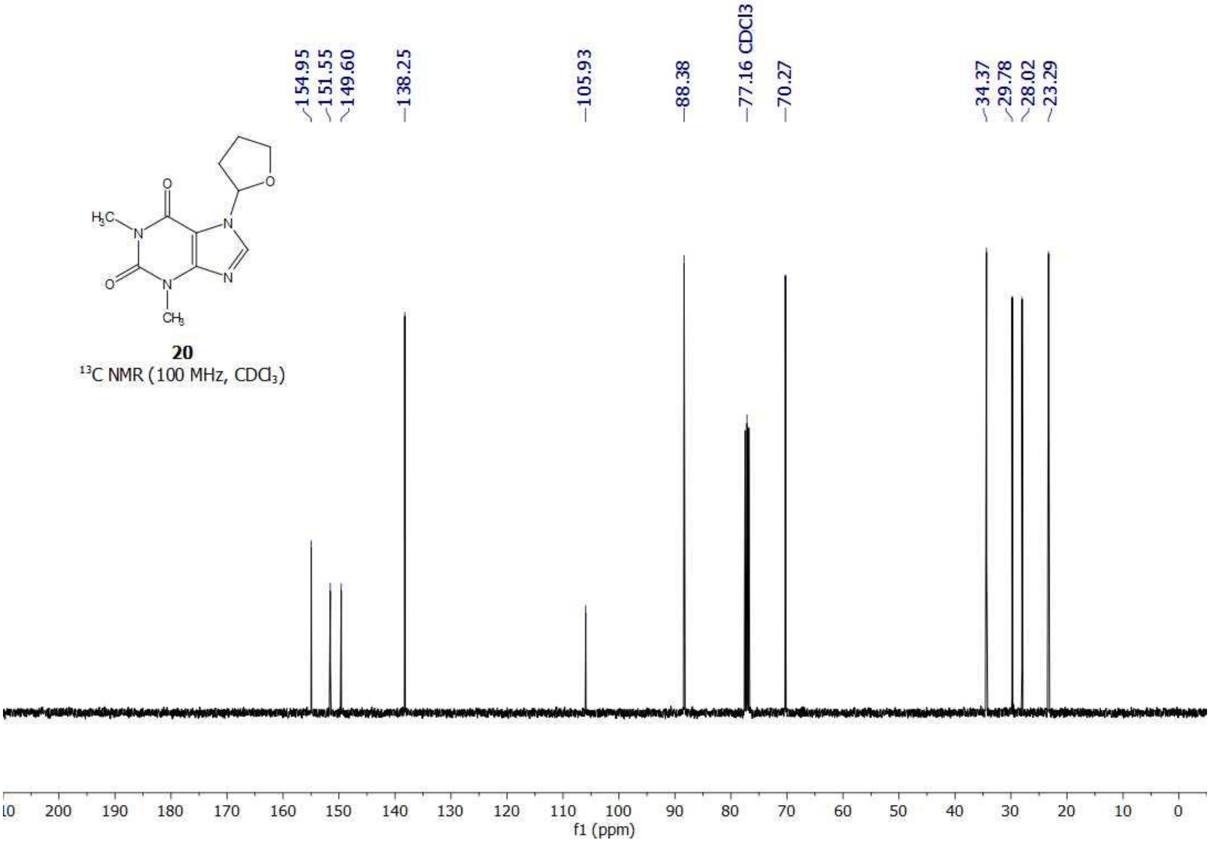

20

^{13}C NMR (100 MHz, $CDCl_3$)

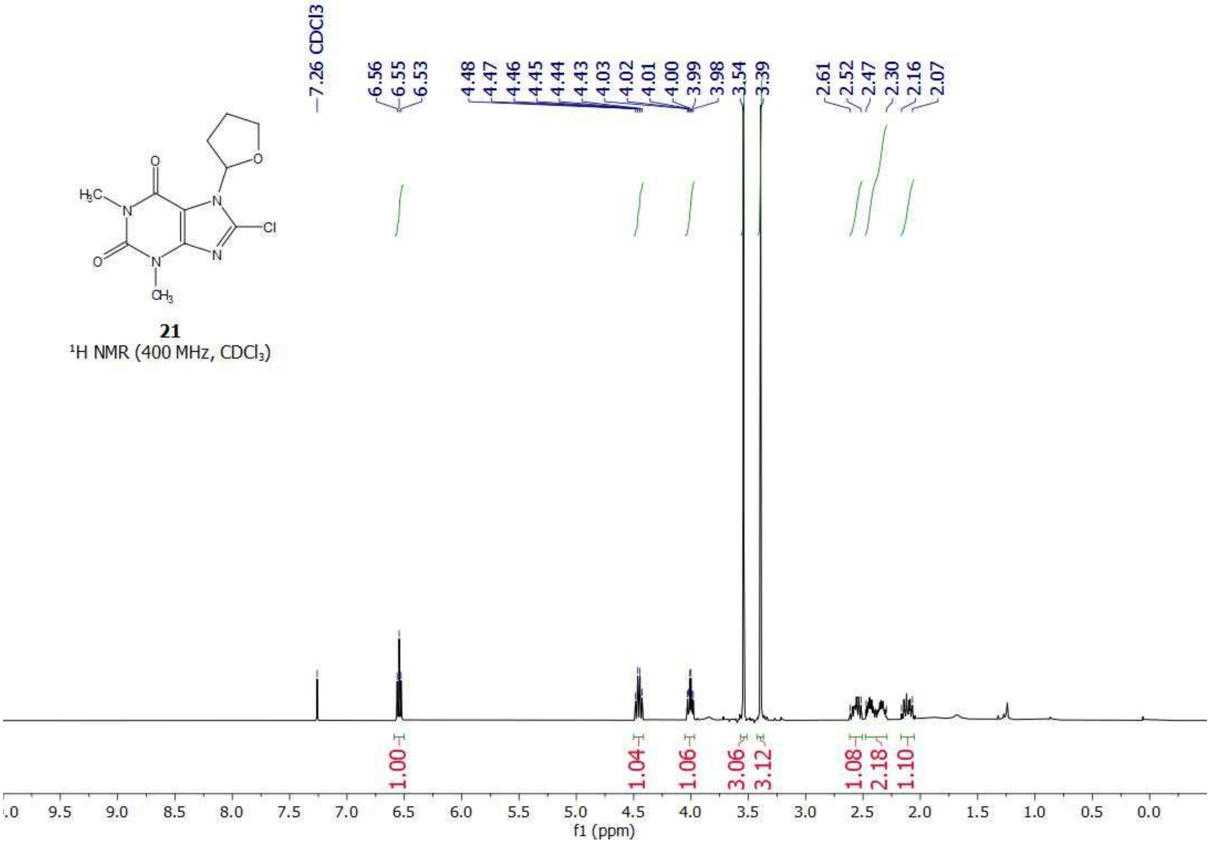
21
^{1}H NMR (400 MHz, CDCl$_3$)
7.26 CDCl3
6.56
6.55
6.53
4.48
4.47
4.46
4.45
4.44
4.43
4.03
4.02
4.01
4.00
3.99
3.98
3.54
3.39
2.61
2.52
2.47
2.30
2.16
2.07
1.00
1.04
1.06
3.06
3.12
1.08
2.18
1.10
f1 (ppm)

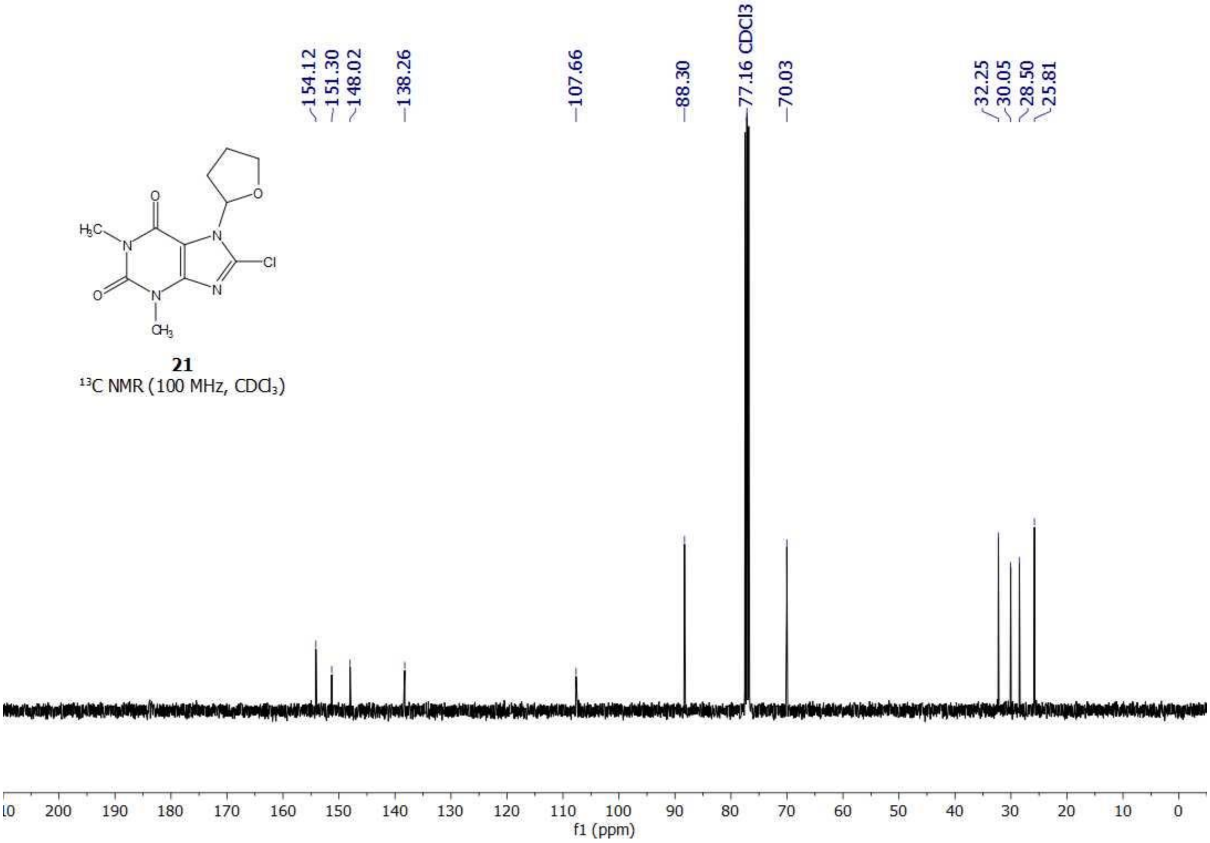
21
^{13}C NMR (100 MHz, CDCl$_3$)
154.12
151.30
148.02
138.26
107.66
88.30
77.16 CDCl3
70.03
32.25
30.05
28.50
25.81
f1 (ppm)

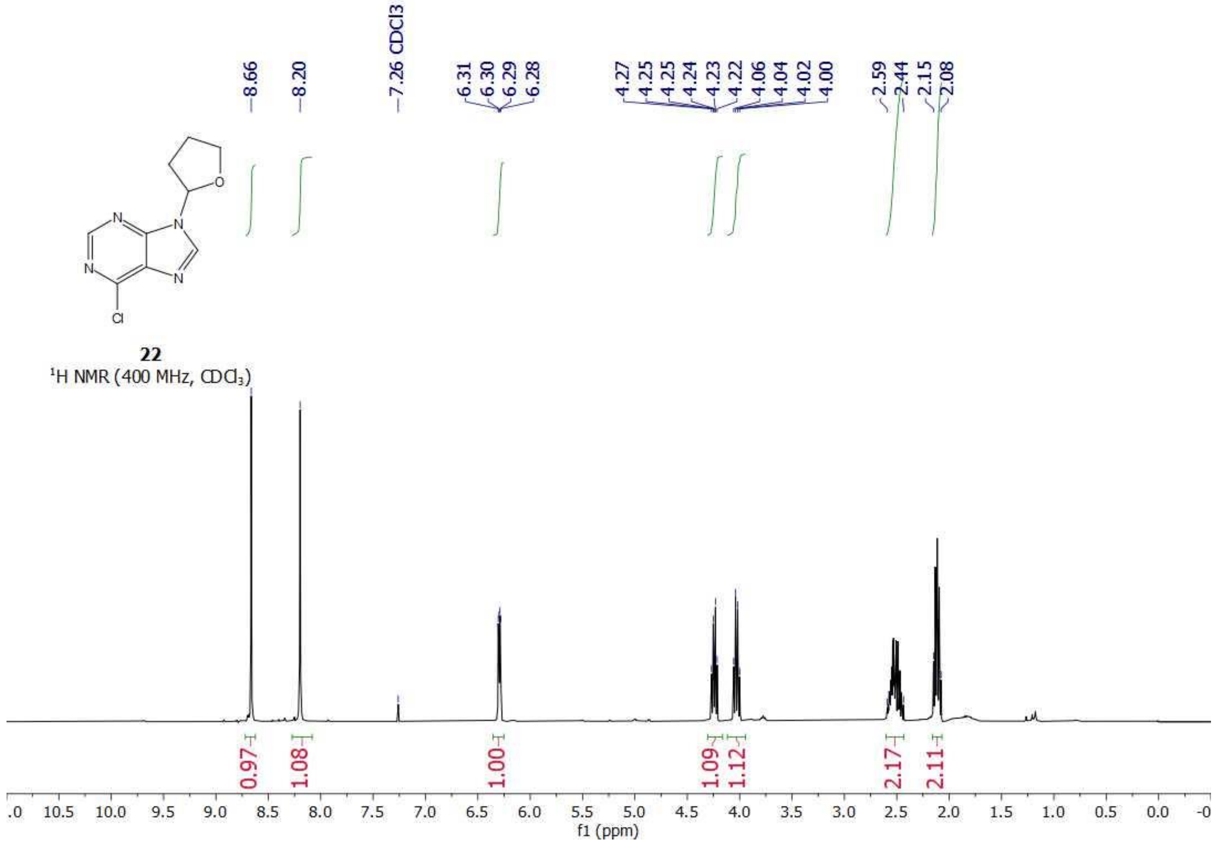
8.66
8.20
7.26 CDCl3
6.31
6.30
6.29
6.28
4.27
4.25
4.25
4.24
4.23
4.22
4.06
4.04
4.02
4.00
2.59
2.44
2.15
2.08
N
N
N
N
O
Cl
22
^{1}H NMR (400 MHz, $CDCl_3$)
0.97
1.08
1.00
1.09
1.12
2.17
2.11
f1 (ppm)

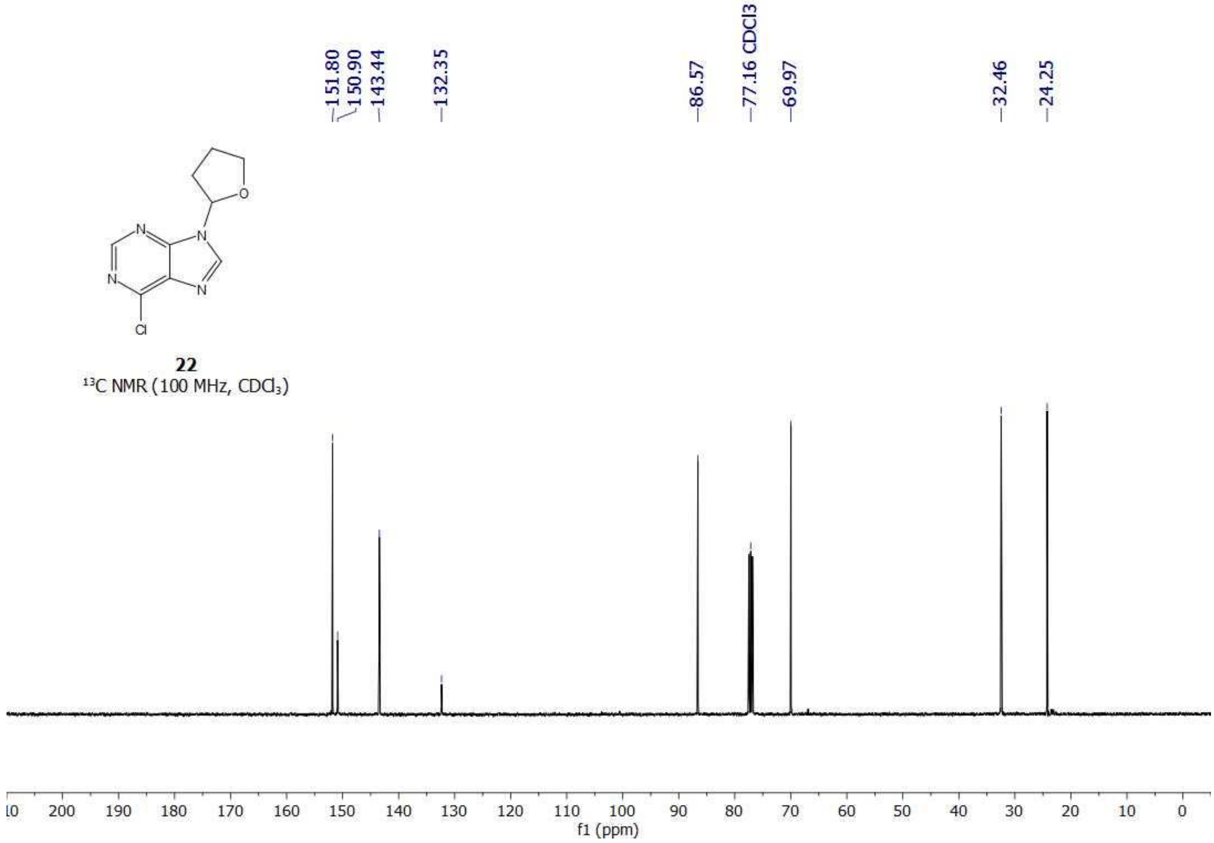
151.80
150.90
143.44
132.35
86.57
77.16 CDCl3
69.97
32.46
24.25
N
N
N
N
O
Cl
22
^{13}C NMR (100 MHz, $CDCl_3$)
f1 (ppm)

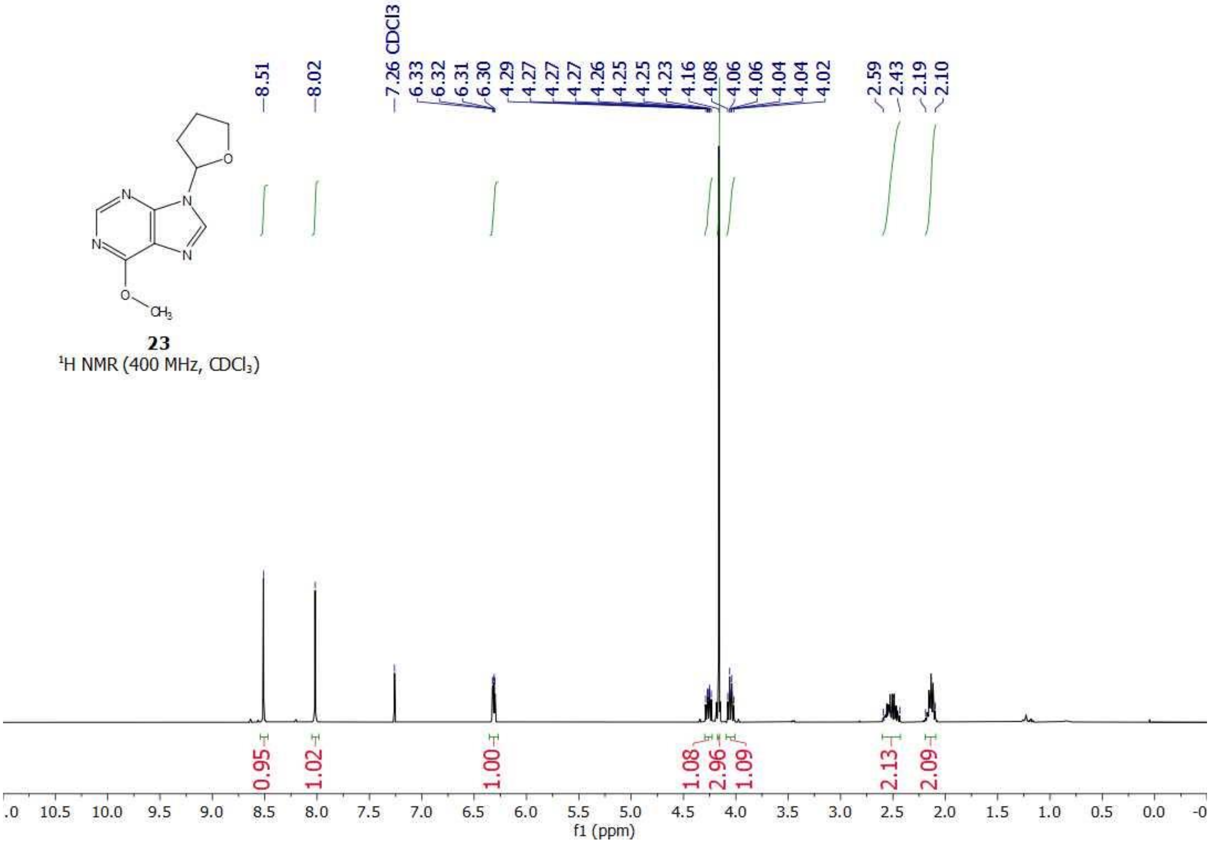

23
^{1}H NMR (400 MHz, $CDCl_3$)

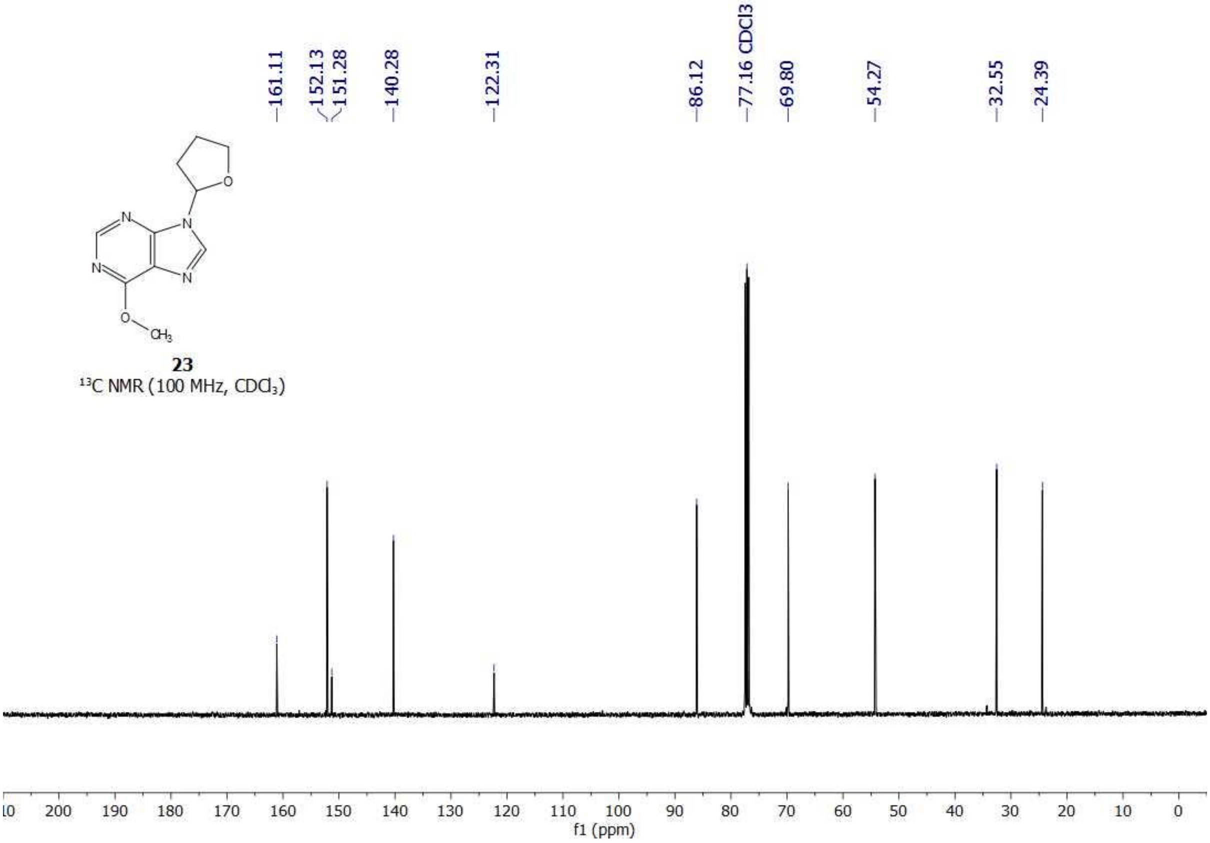

23
^{13}C NMR (100 MHz, $CDCl_3$)

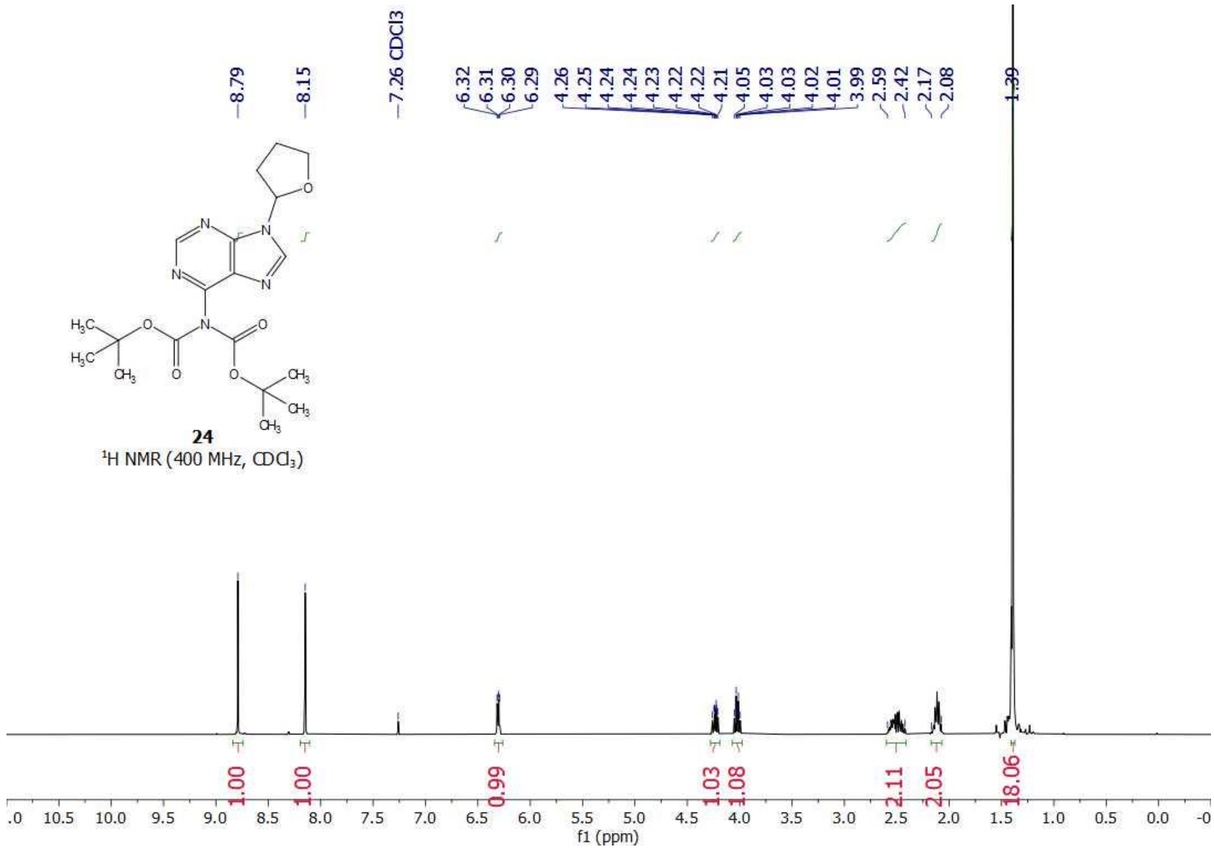

24
^{1}H NMR (400 MHz, $CDCl_3$)

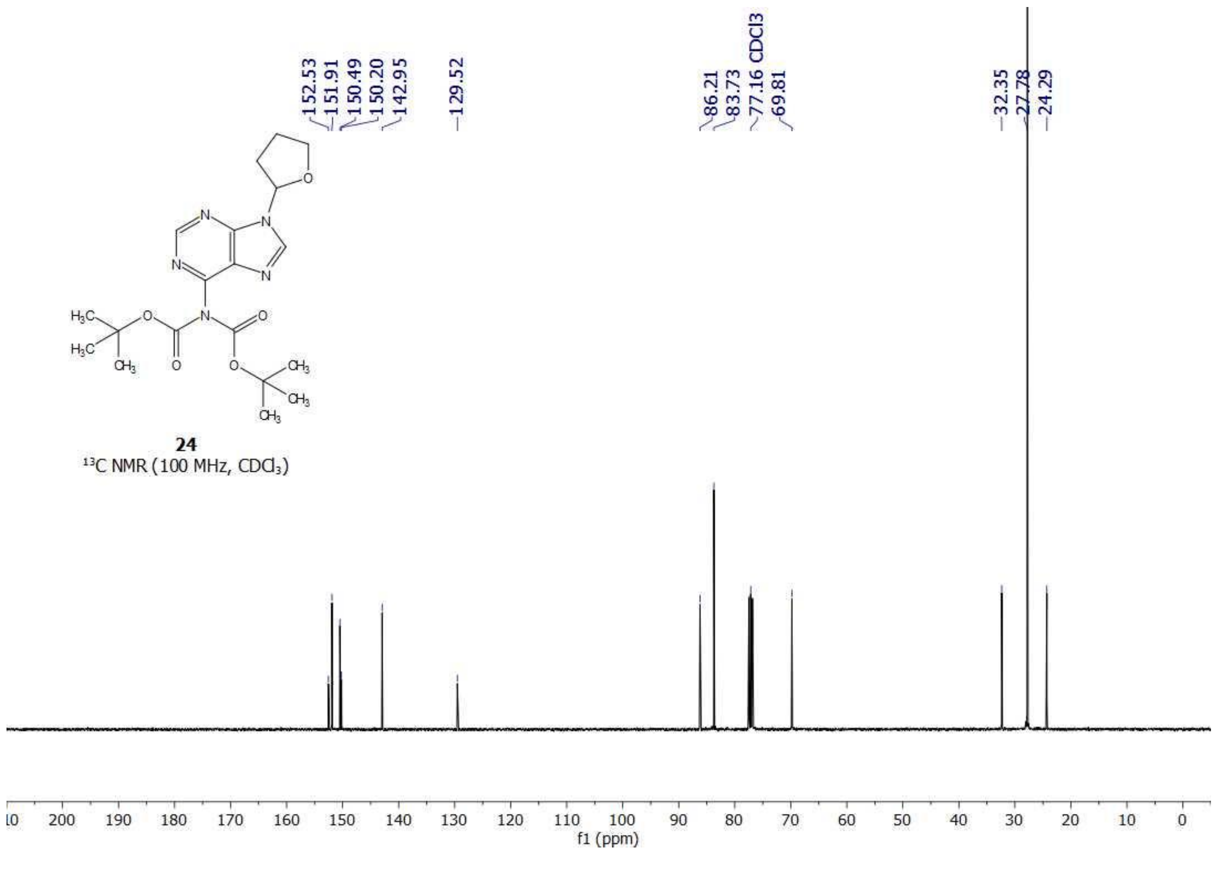

24
^{13}C NMR (100 MHz, $CDCl_3$)

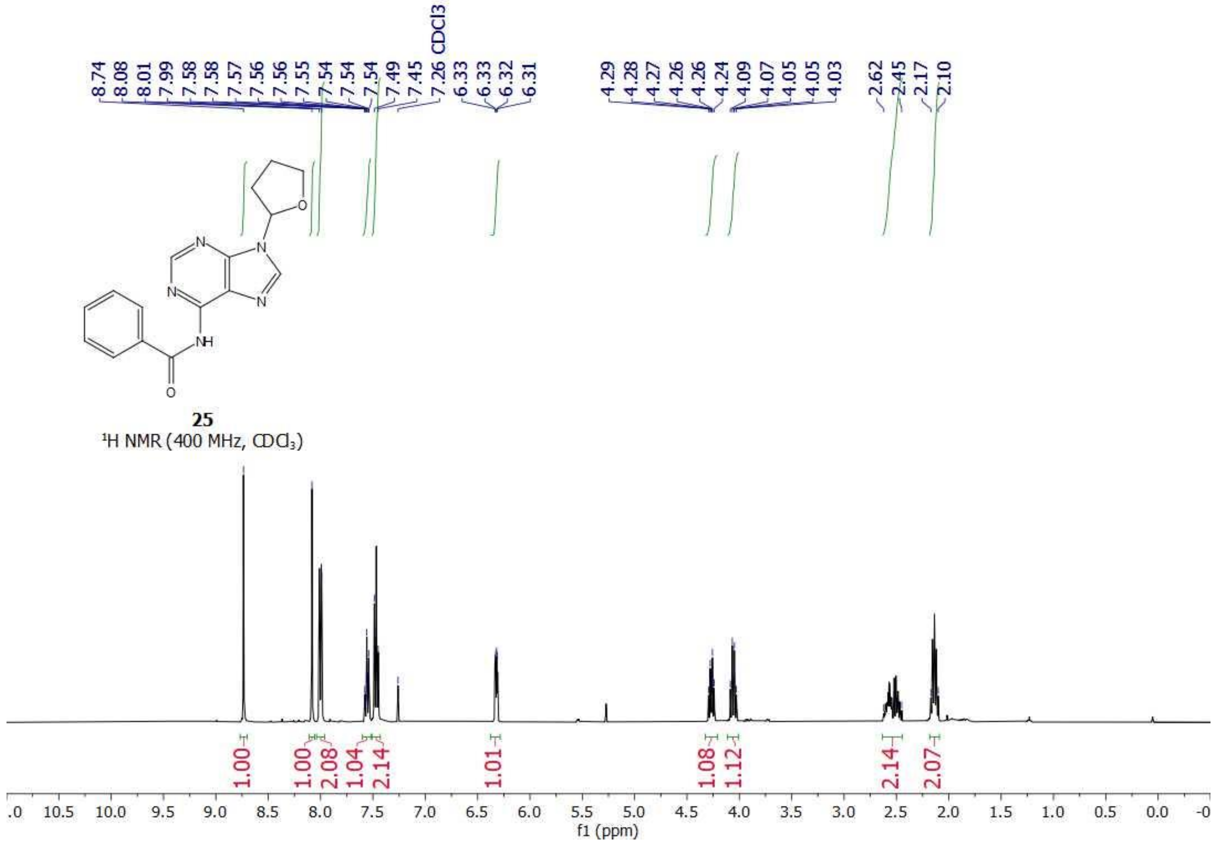
8.74
8.08
8.01
7.99
7.58
7.58
7.57
7.56
7.56
7.55
7.54
7.54
7.54
7.54
7.49
7.45
7.26 CDCl3
6.33
6.33
6.32
6.31
4.29
4.28
4.27
4.26
4.26
4.24
4.09
4.07
4.05
4.05
4.03
2.62
2.45
2.17
2.10
N
N
N
N
NH
O
O
25
1H NMR (400 MHz, CDCl3)
1.00
1.00
2.08
1.04
2.14
1.01
1.08
1.12
2.14
2.07
f1 (ppm)

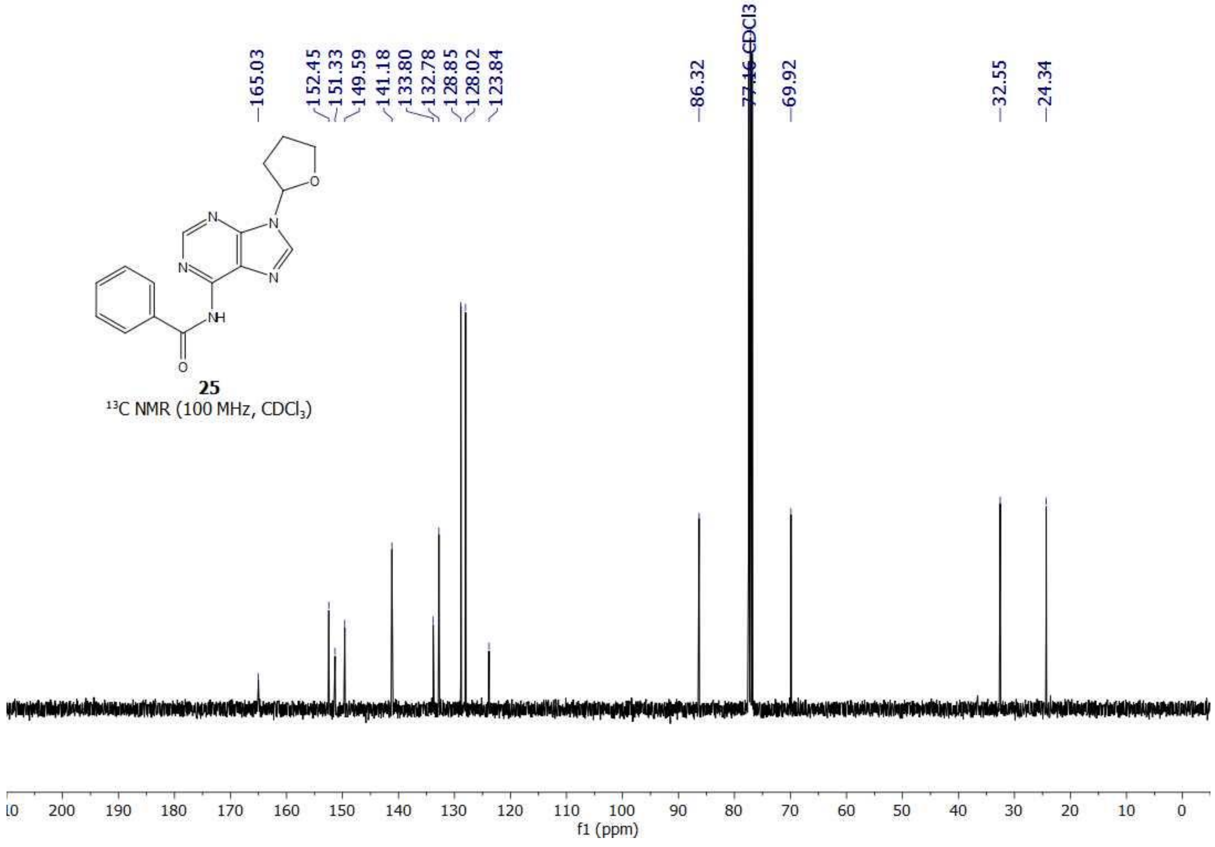
165.03
152.45
151.33
149.59
141.18
133.80
132.78
128.85
128.02
123.84
86.32
77.16 CDCl3
69.92
32.55
24.34
N
N
N
N
NH
O
O
25
13C NMR (100 MHz, CDCl3)
f1 (ppm)

26
^{1}H NMR (400 MHz, $CDCl_3$)

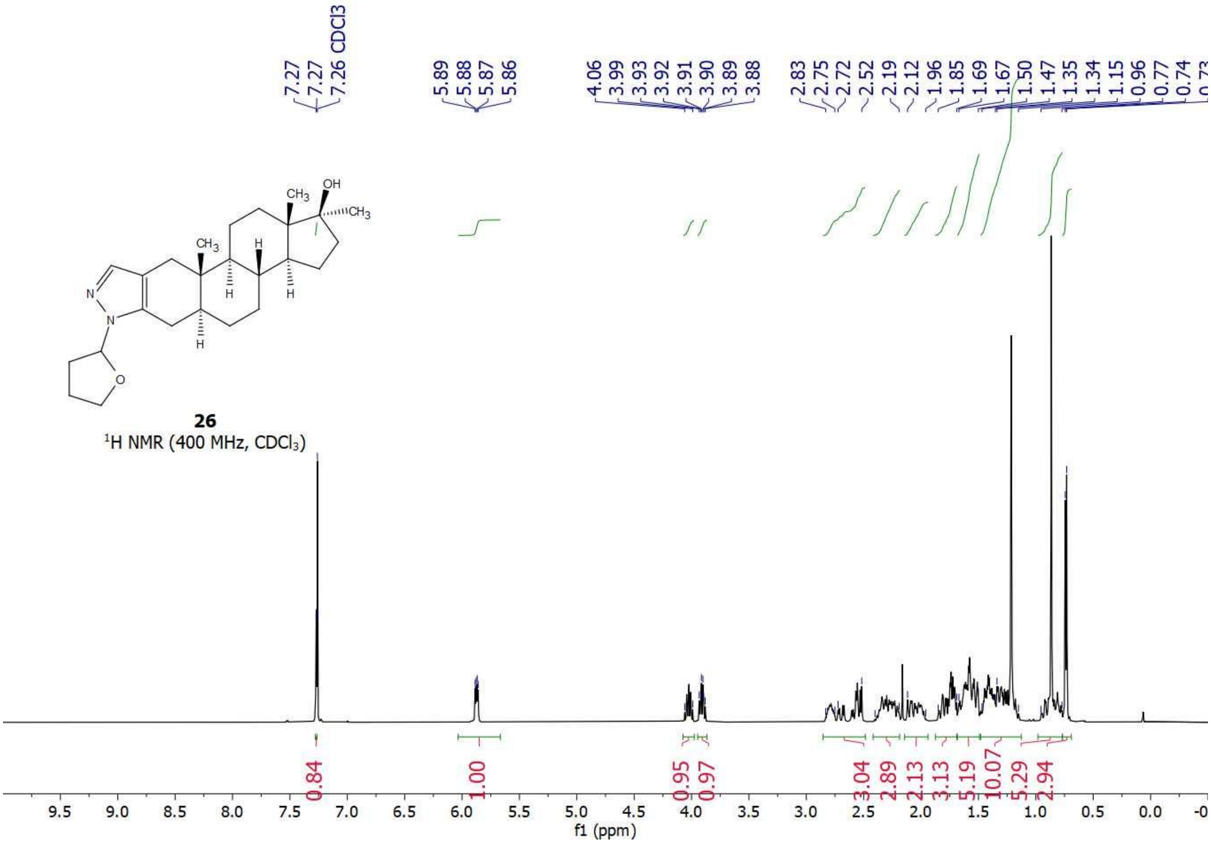

26
^{13}C NMR (100 MHz, $CDCl_3$)

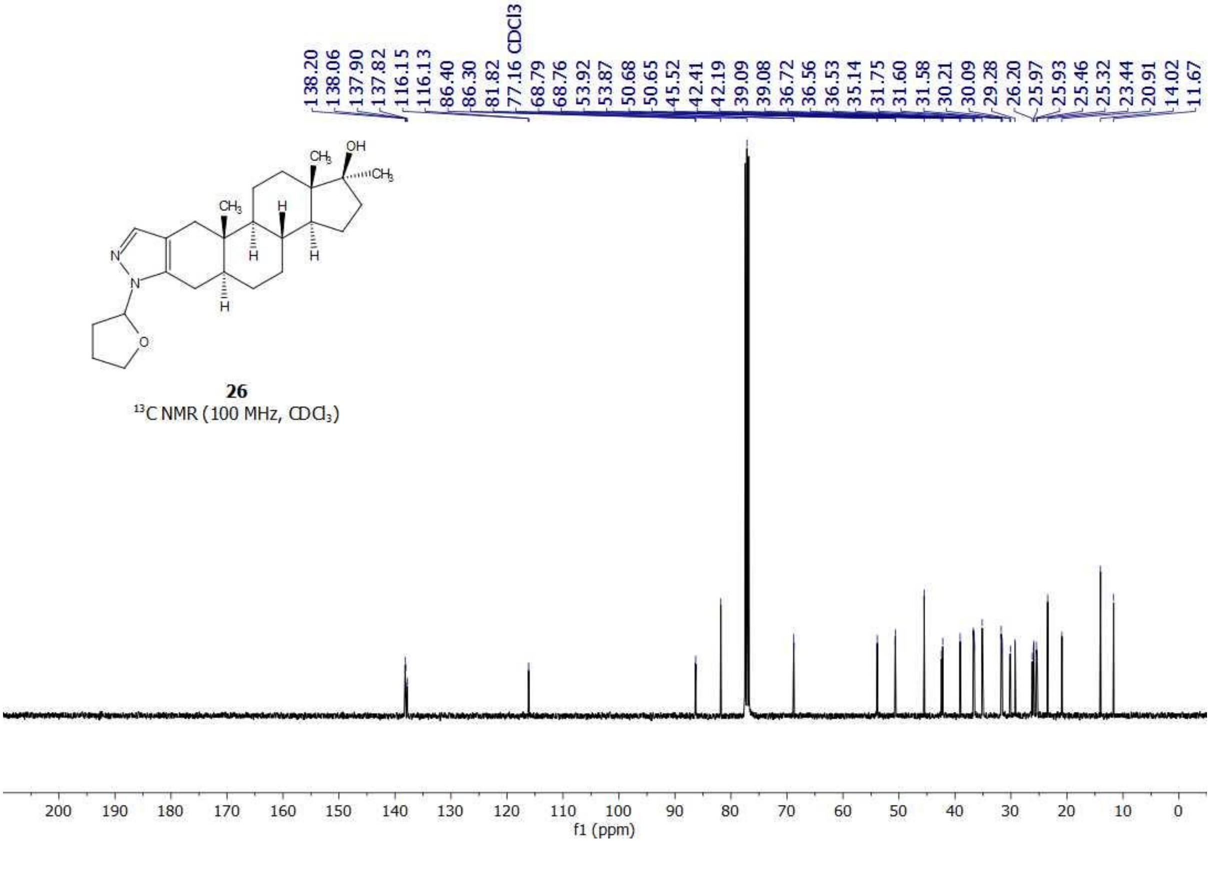

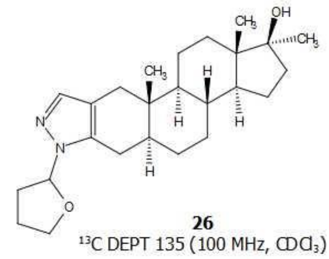

26
^{13}C DEPT 135 (100 MHz, $CDCl_3$)

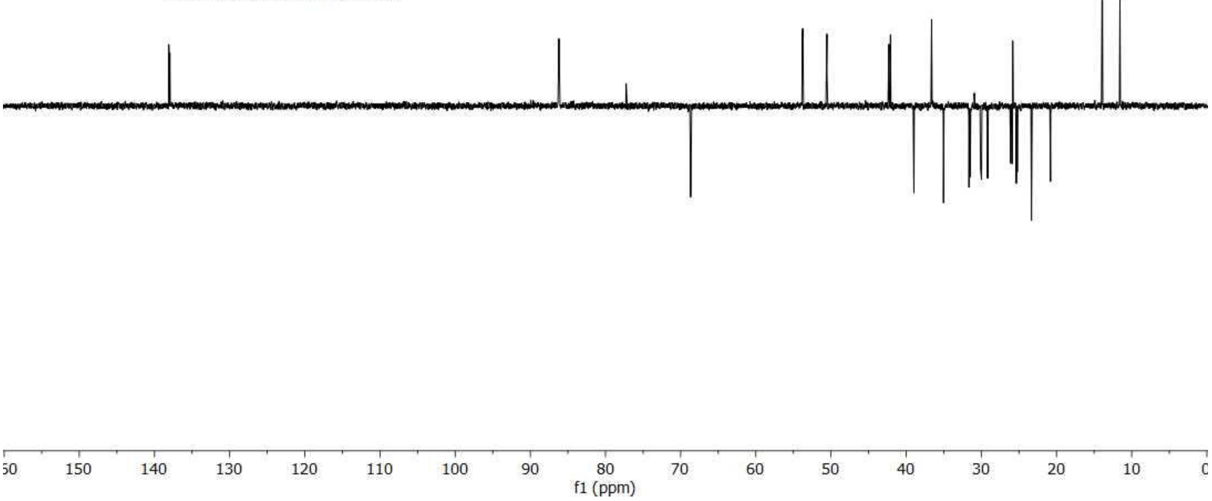

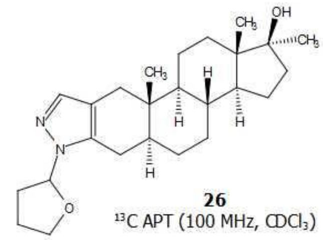

26
^{13}C APT (100 MHz, $CDCl_3$)

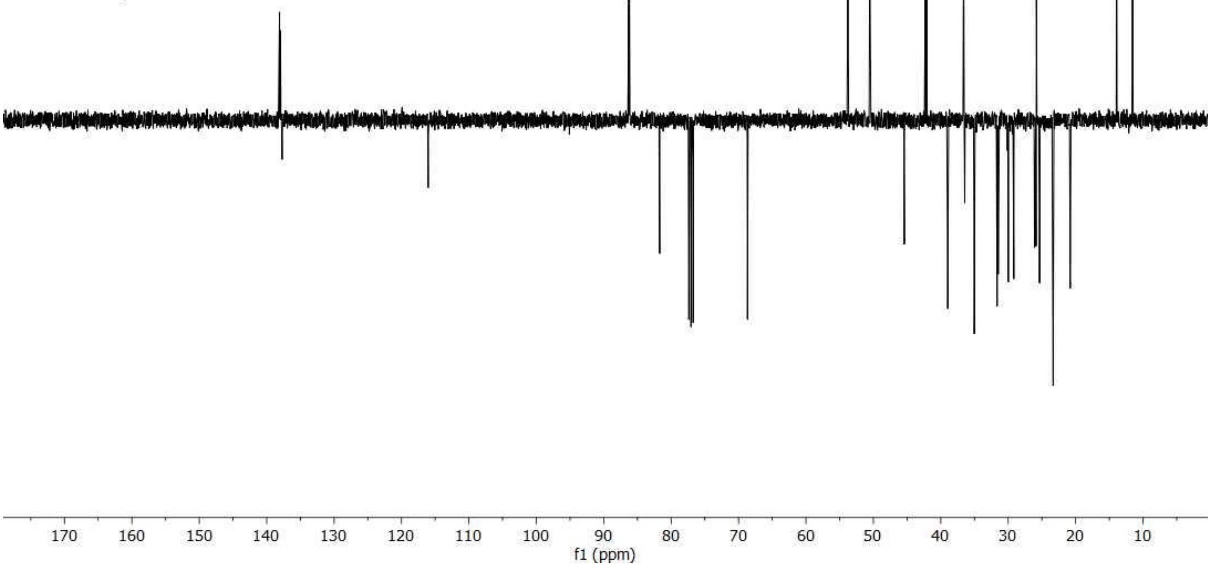

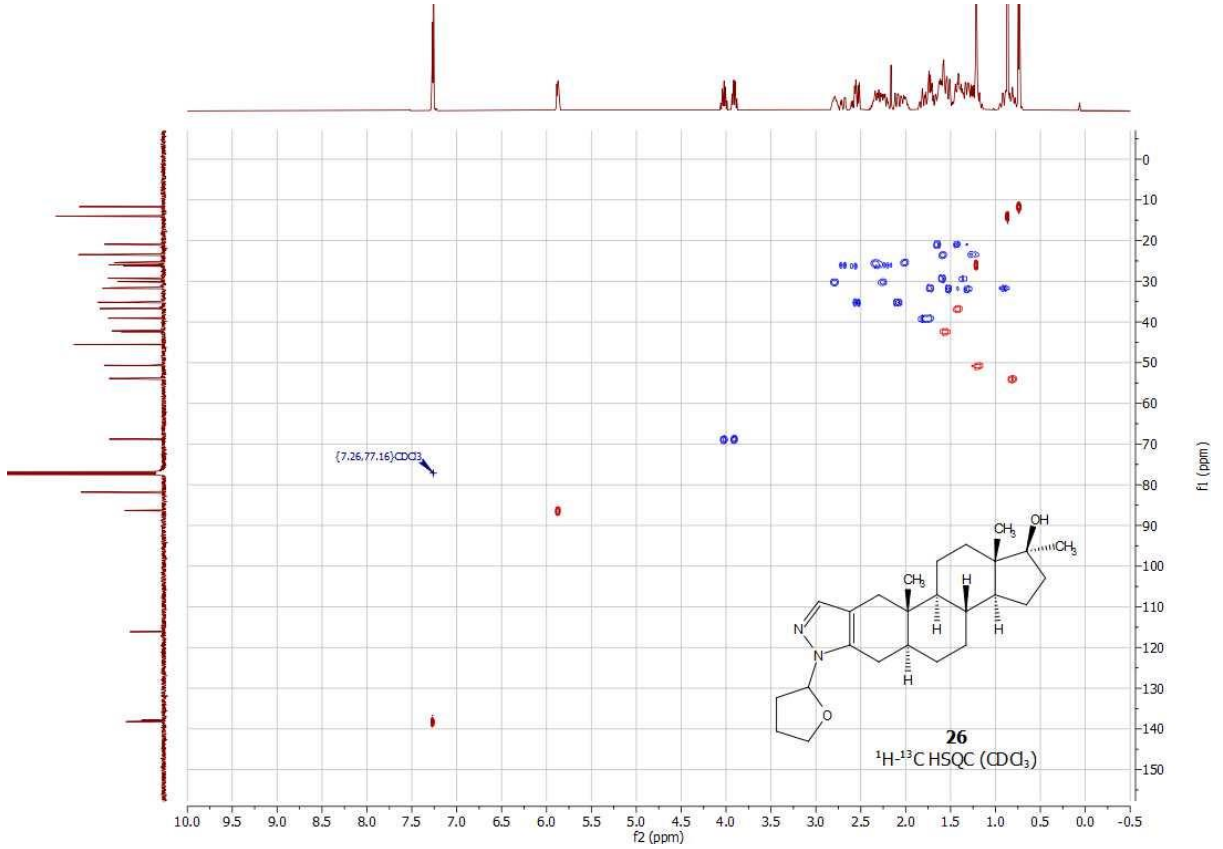
{7.26,77.16}CDCl3
26
^{1}H-^{13}C HSQC (CDCl$_3$)
f2 (ppm)
f1 (ppm)

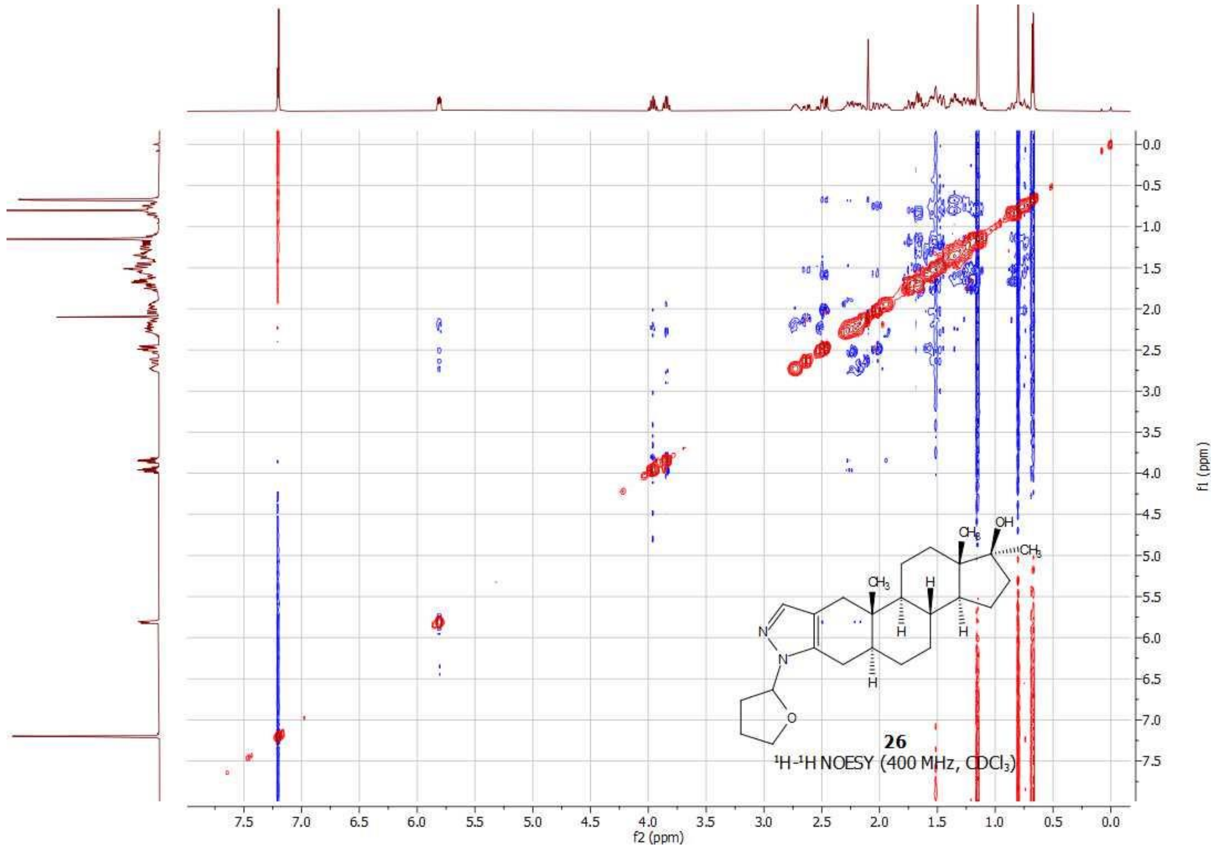
26
^{1}H-^{1}H NOESY (400 MHz, CDCl$_3$)
f2 (ppm)
f1 (ppm)

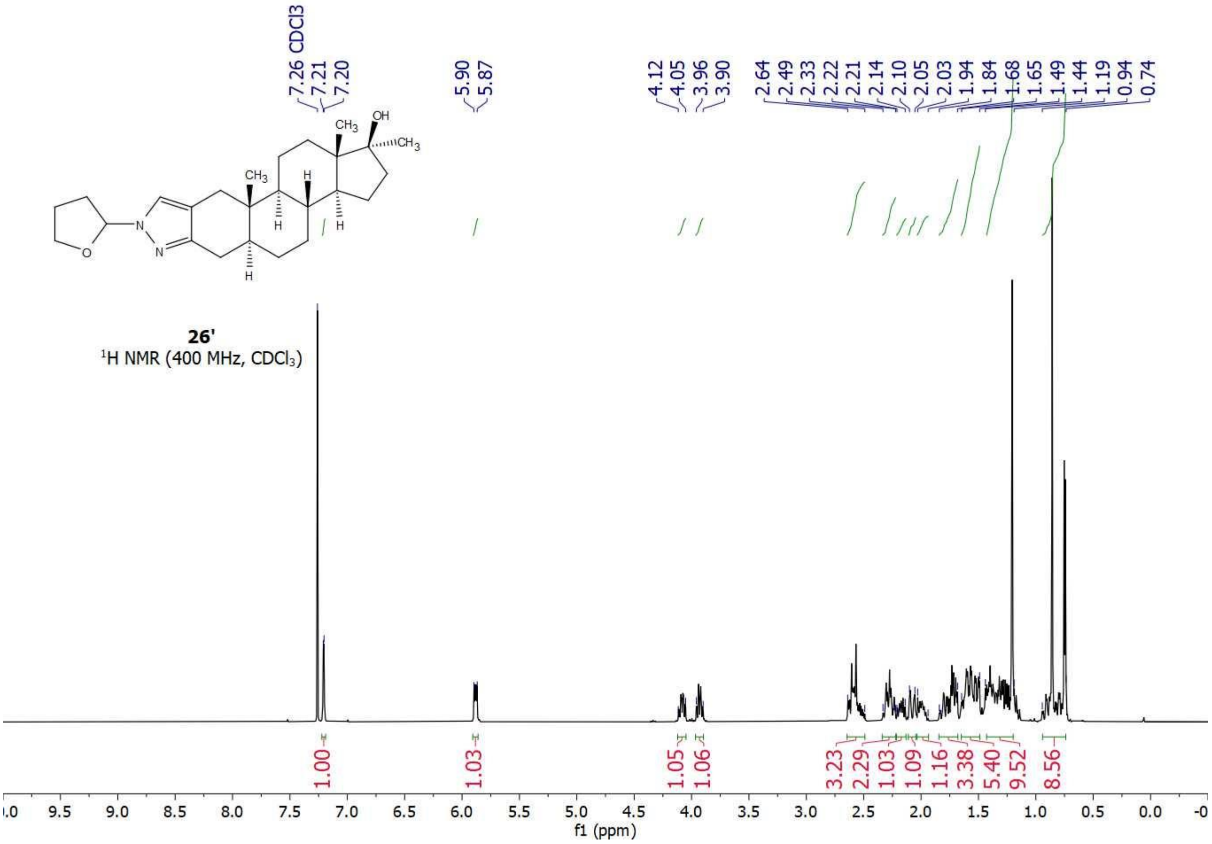

26'

^{1}H NMR (400 MHz, $CDCl_3$)

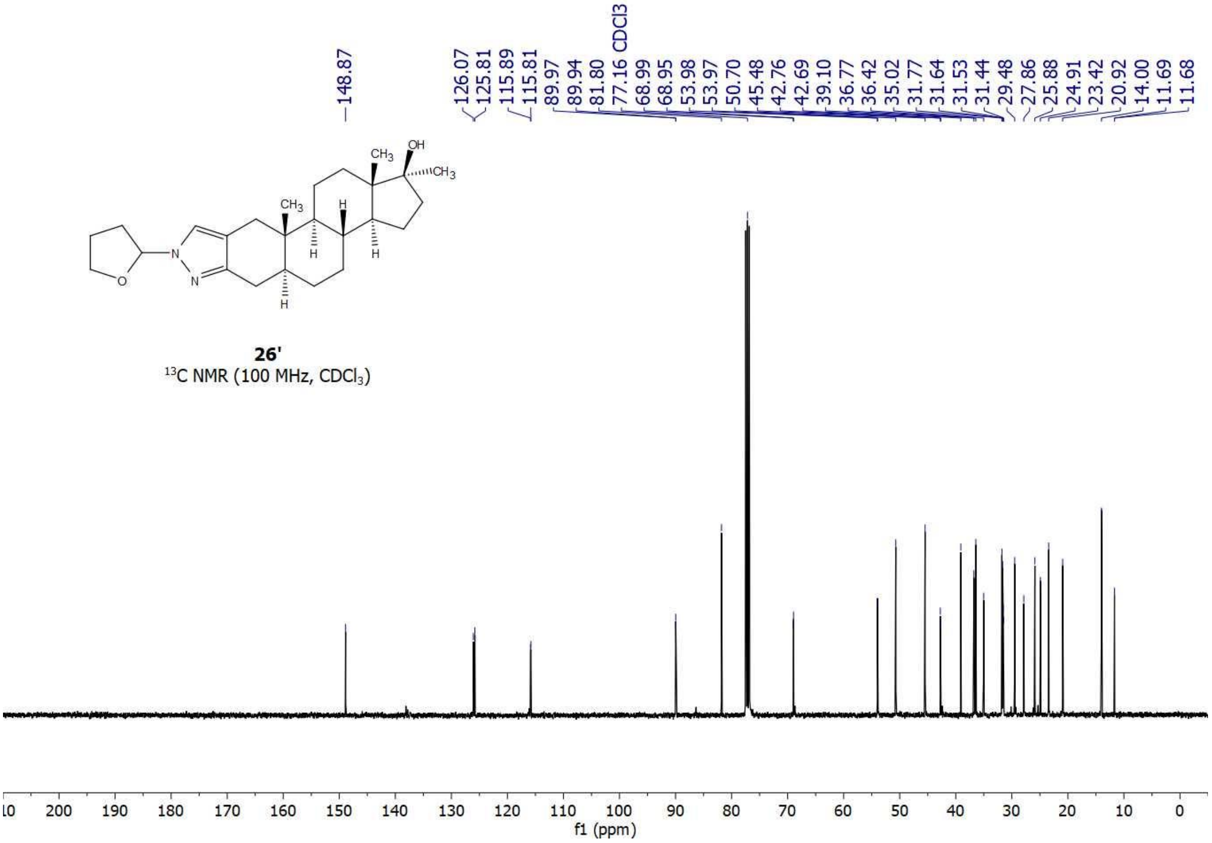

26'

^{13}C NMR (100 MHz, $CDCl_3$)

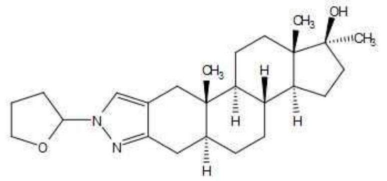

26'
^{13}C DEPT 135 (100 MHz, $CDCl_3$)

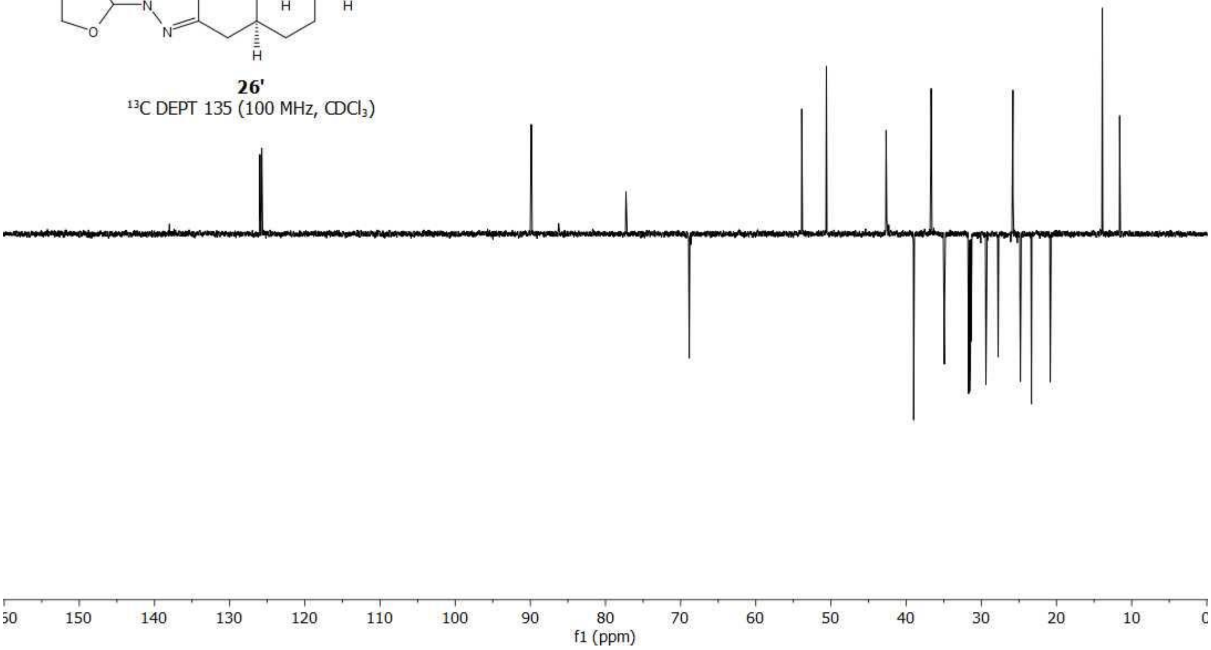

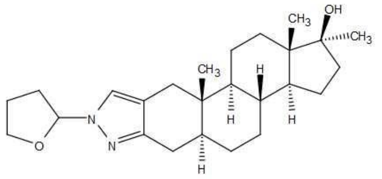

26'
^{13}C APT (100 MHz, $CDCl_3$)

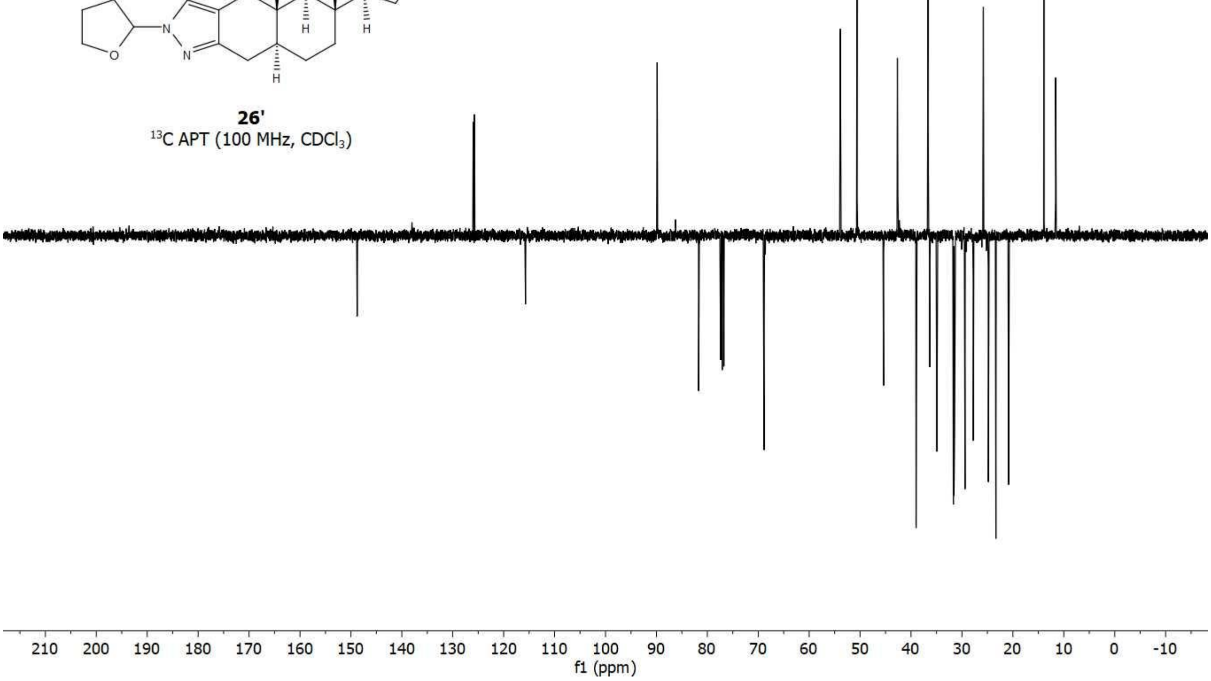

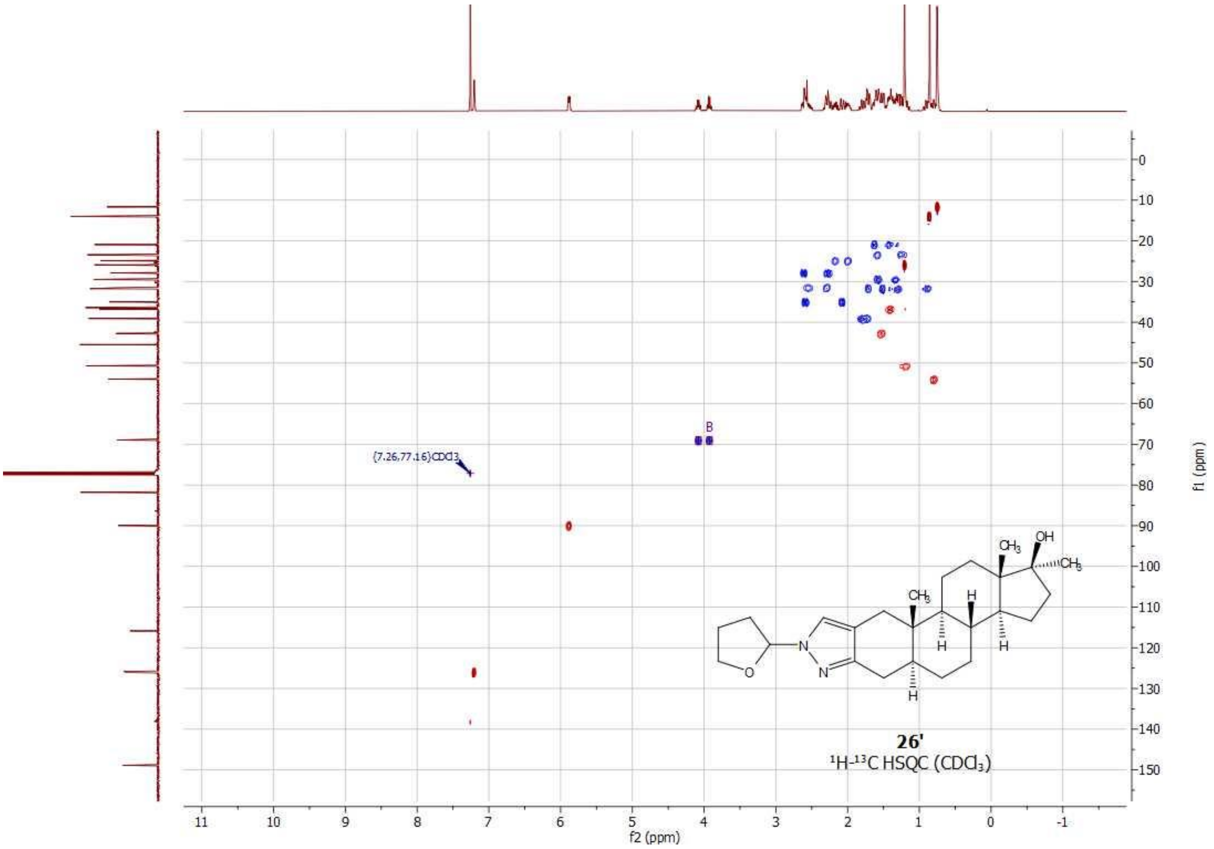
(7.26,77.16)CDCl3
B
f2 (ppm)
f1 (ppm)
26'
^1H-^{13}C HSQC ($CDCl_3$)

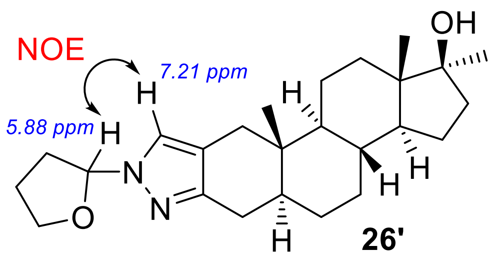
NOE
7.21 ppm
5.88 ppm
OH
26'

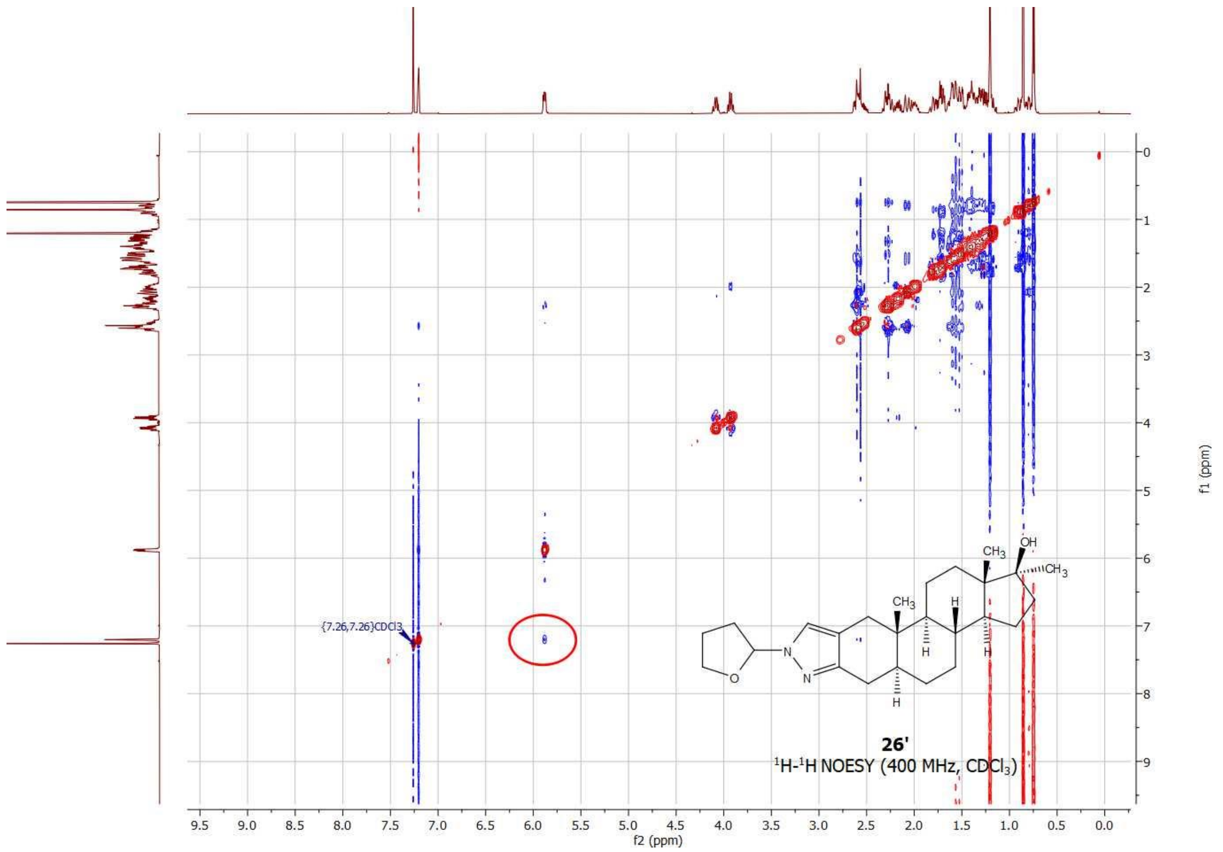
{7.26,7.26}CDCl3
26'
¹H-¹H NOESY (400 MHz, CDCl₃)
f2 (ppm)
f1 (ppm)

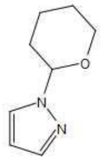

27

^{1}H NMR (400 MHz, $CDCl_3$)

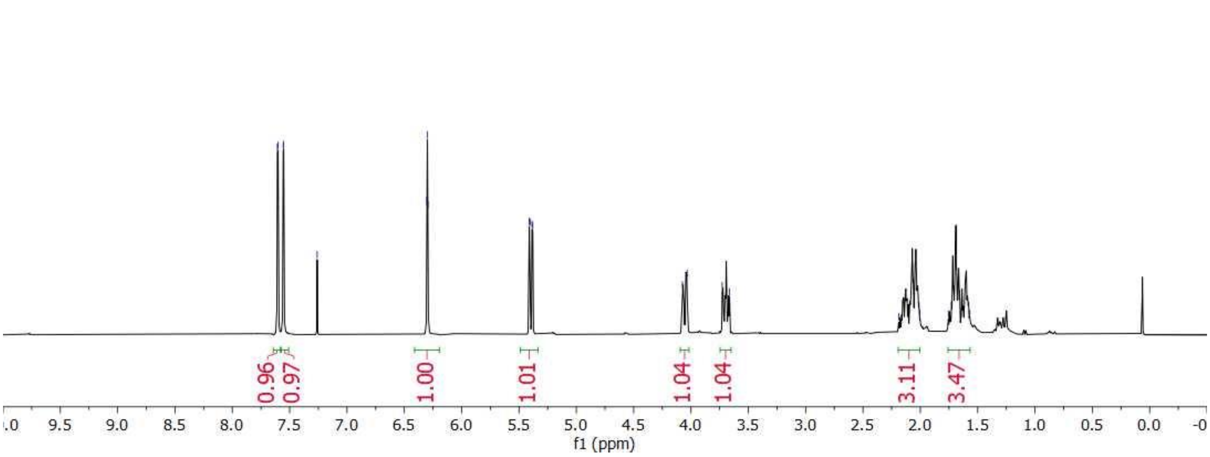

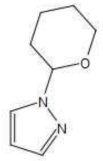

27

^{13}C NMR (100 MHz, $CDCl_3$)

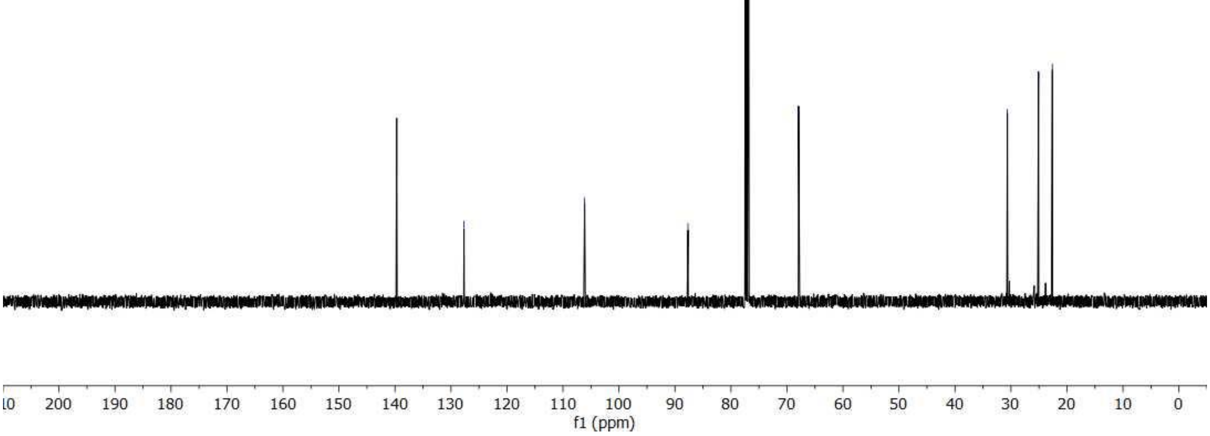

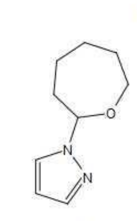

28
^1H NMR (400 MHz, $CDCl_3$)

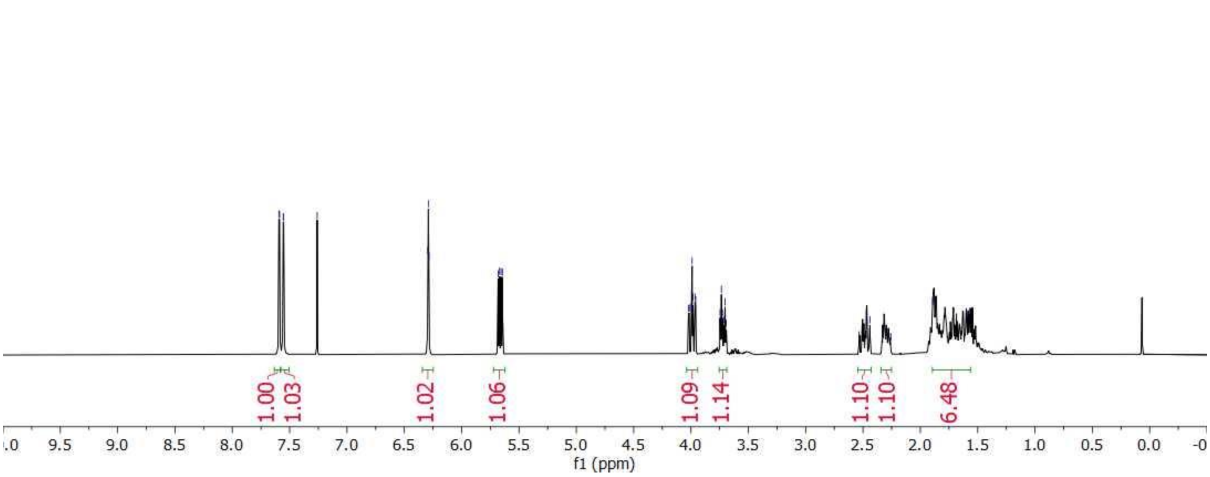

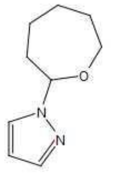

28
^{13}C NMR (100 MHz, $CDCl_3$)

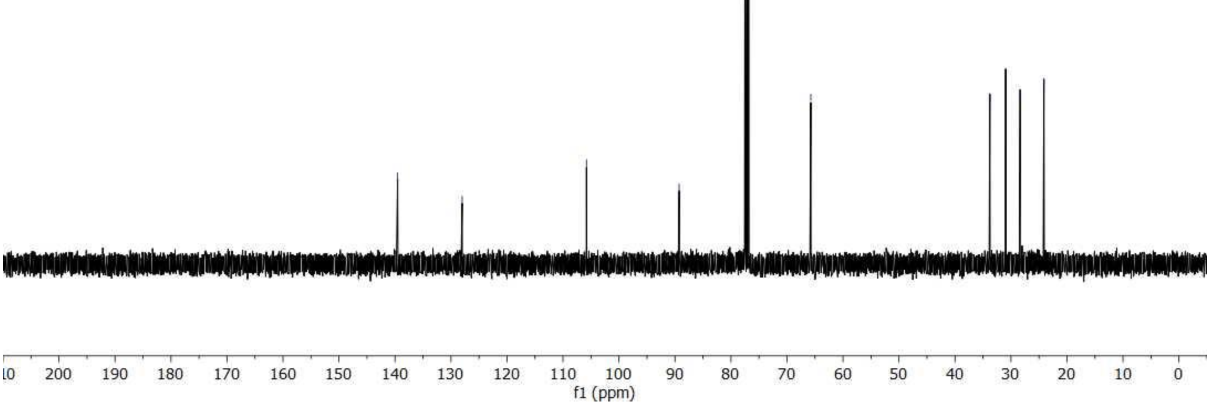

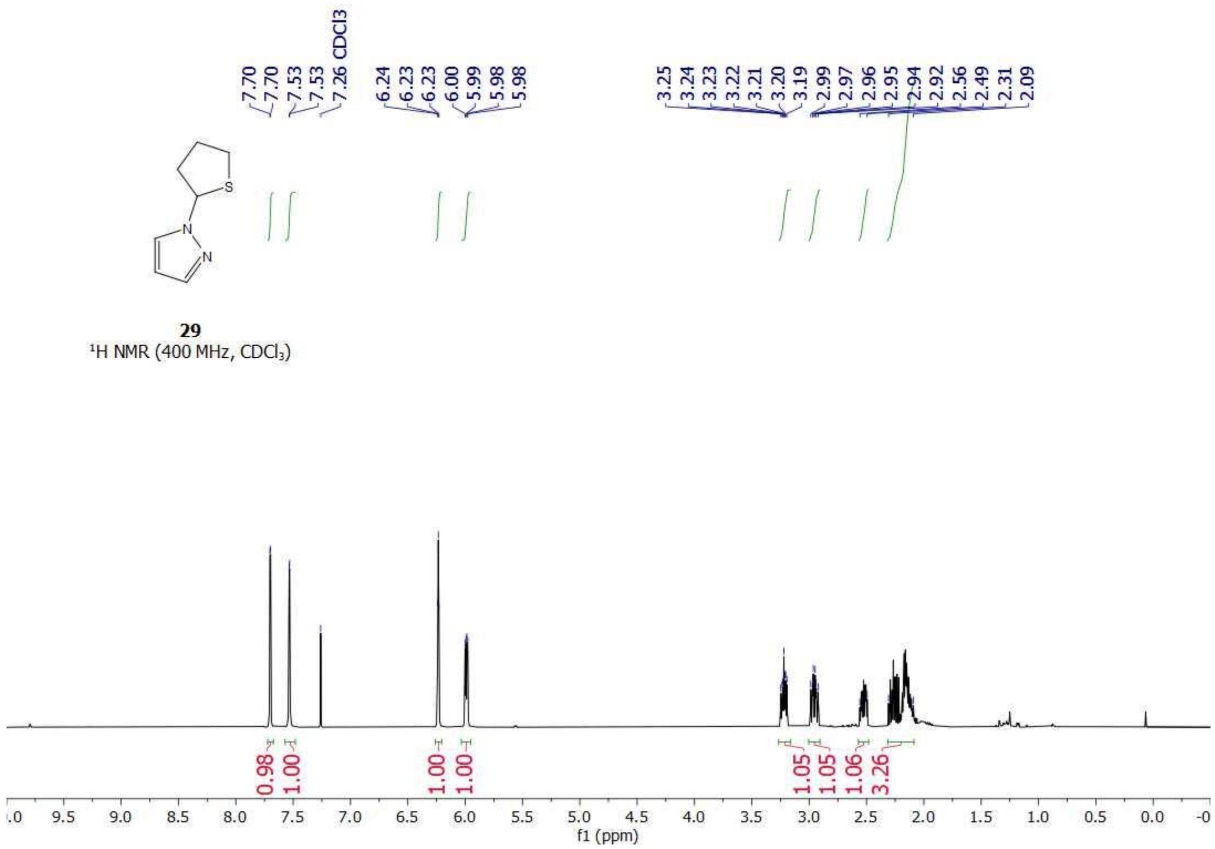

29
^{1}H NMR (400 MHz, $CDCl_3$)

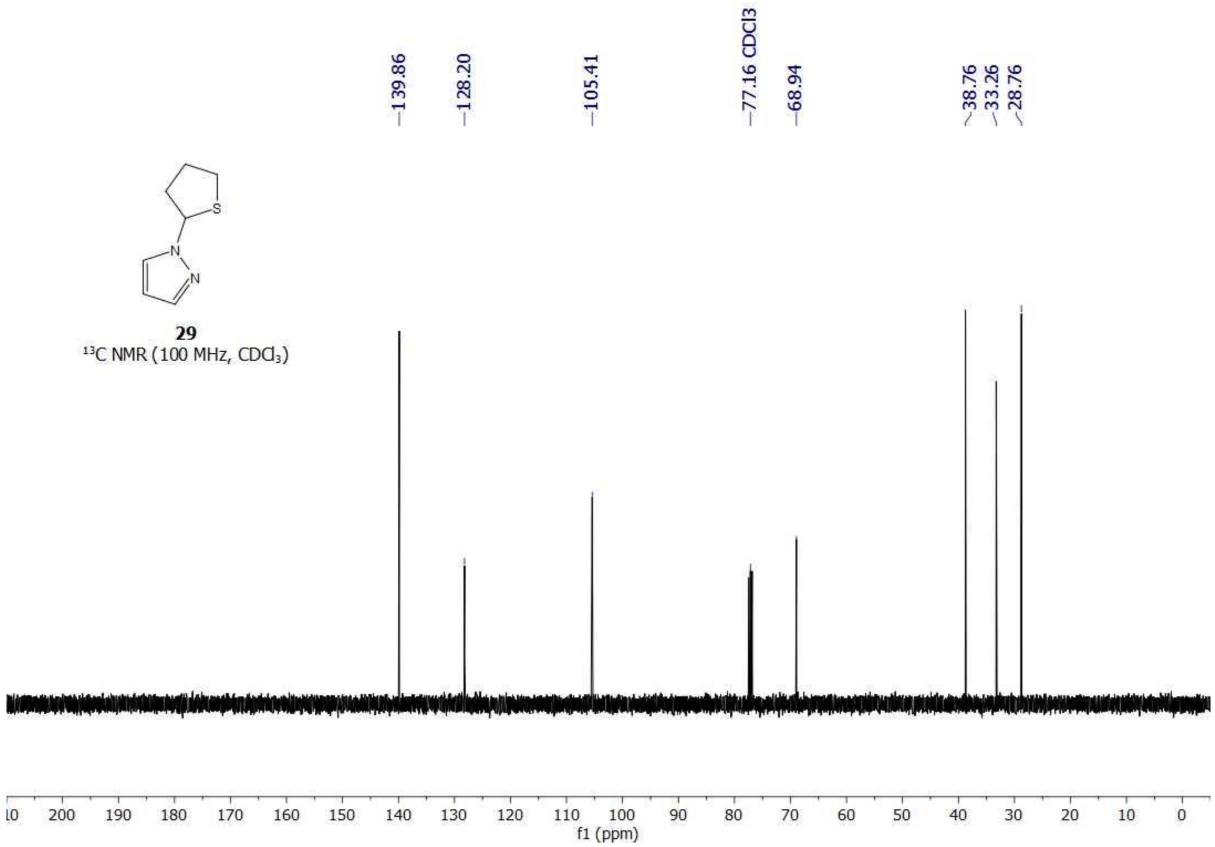

29
^{13}C NMR (100 MHz, $CDCl_3$)

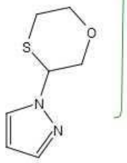

30
^{1}H NMR (400 MHz, $CDCl_3$)

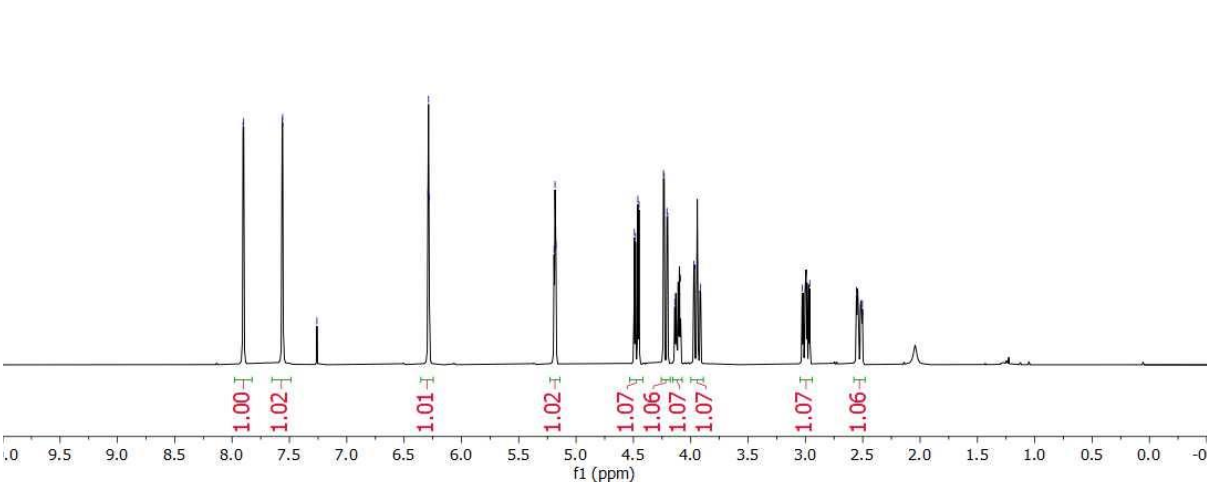

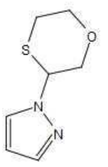

30
^{13}C NMR (100 MHz, $CDCl_3$)

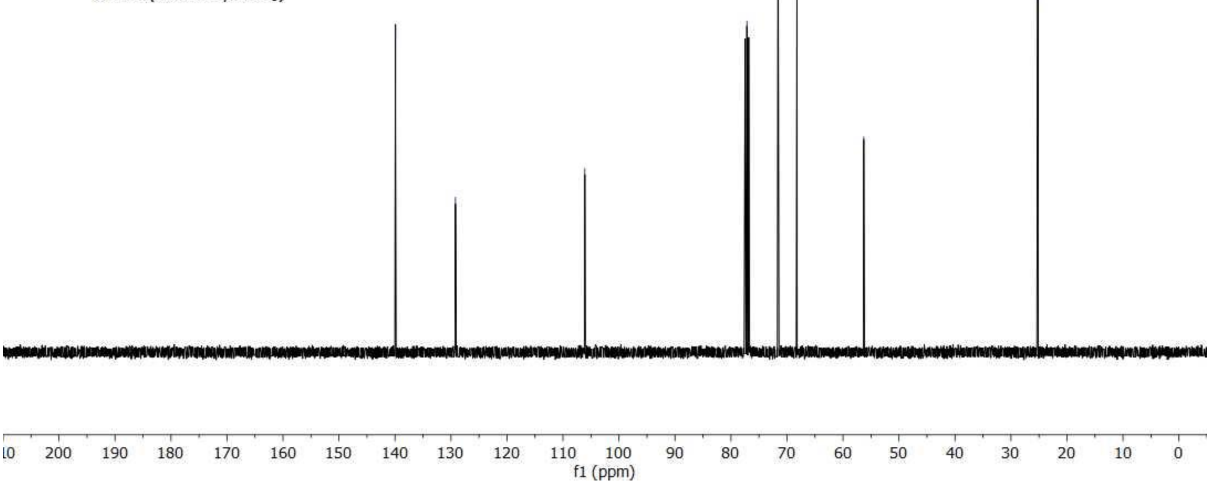

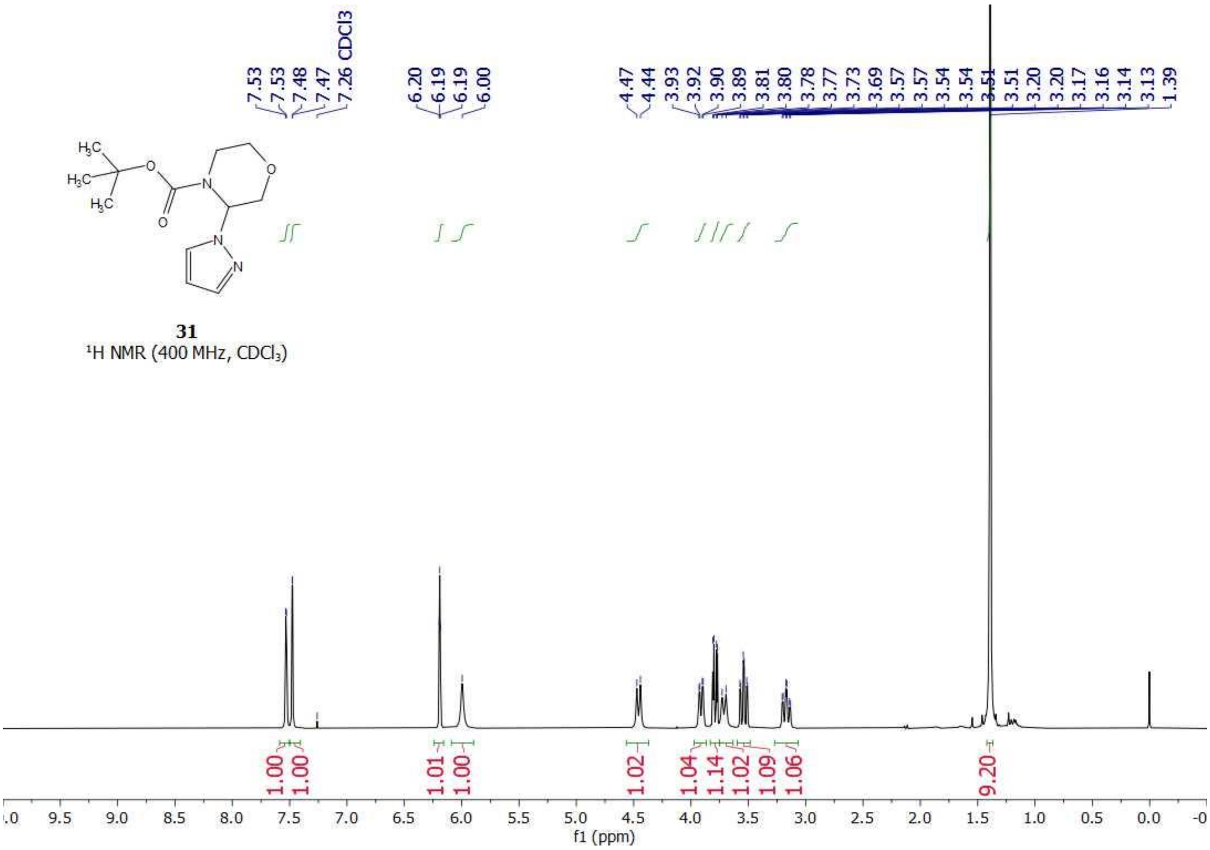

31
^{1}H NMR (400 MHz, $CDCl_3$)

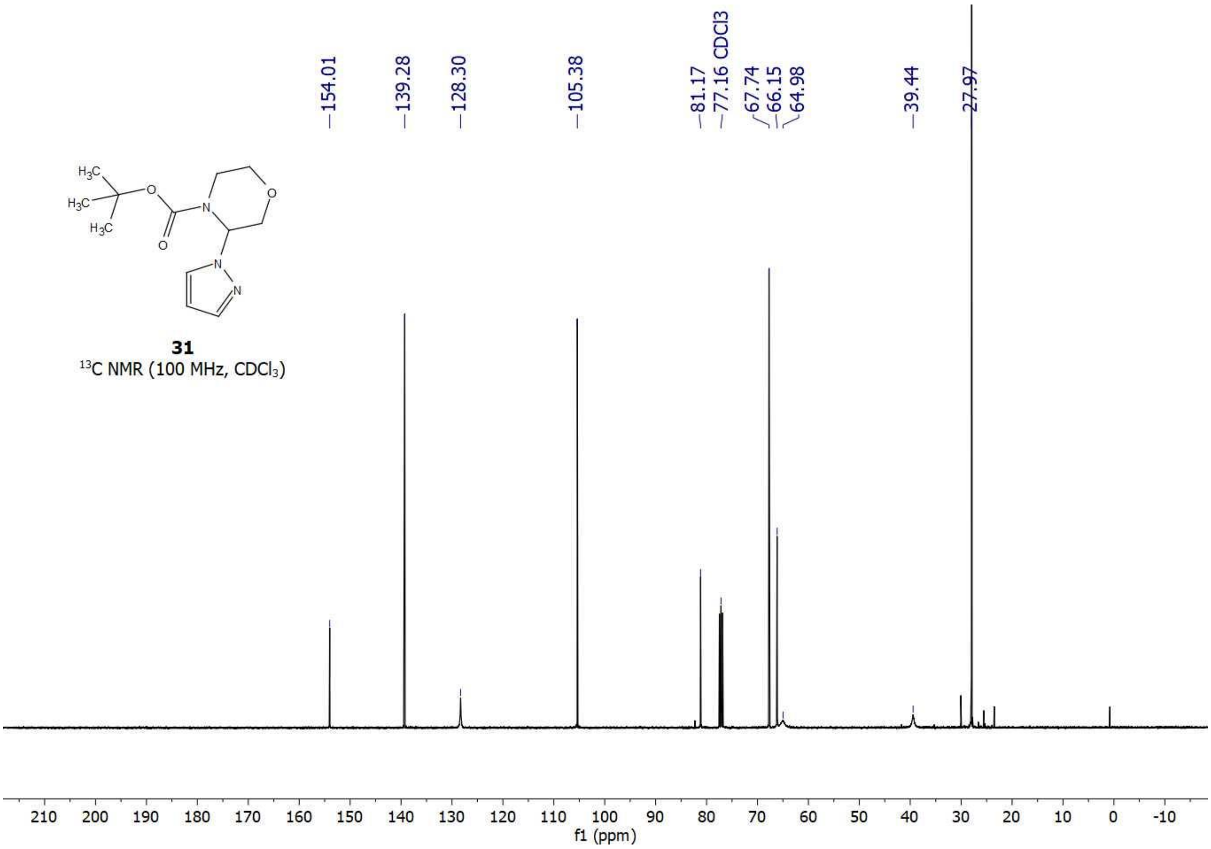

31
^{13}C NMR (100 MHz, $CDCl_3$)

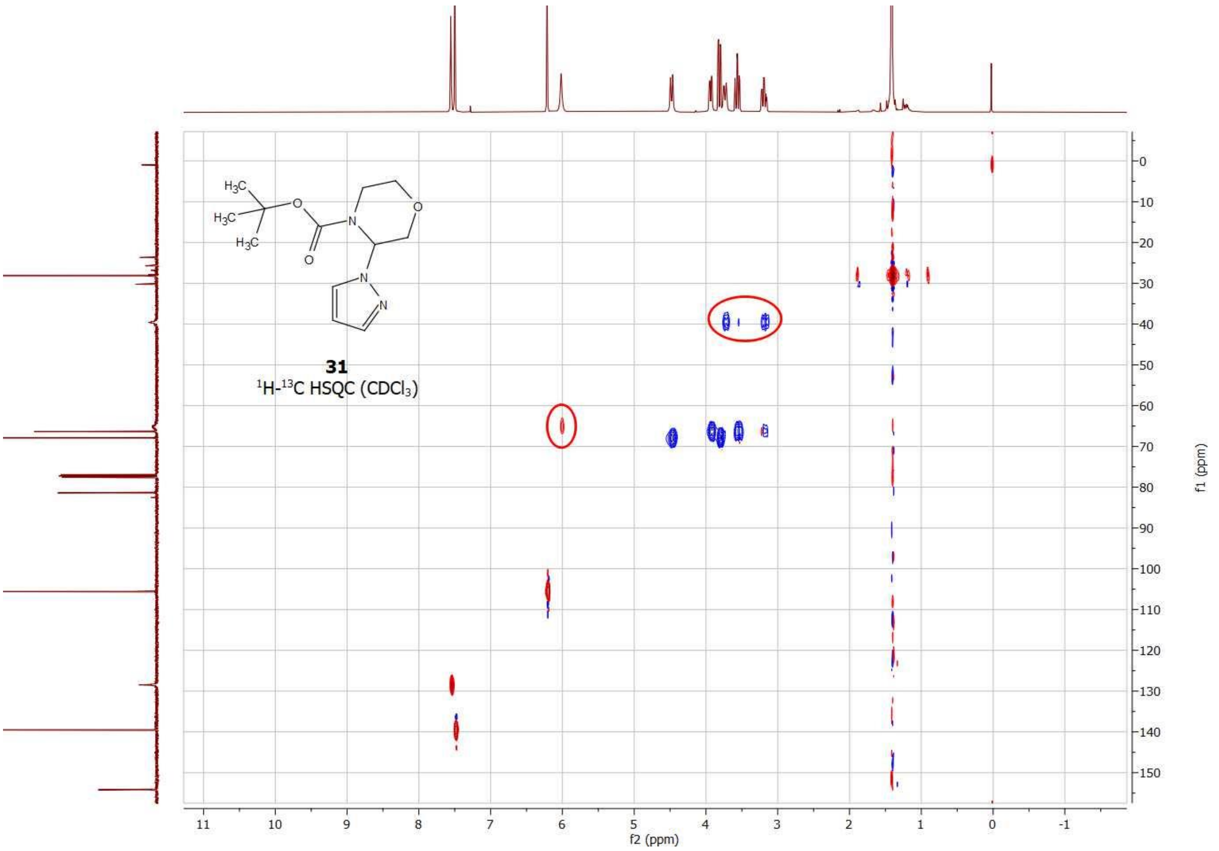
31
1H-13C HSQC (CDCl3)
f2 (ppm)
f1 (ppm)

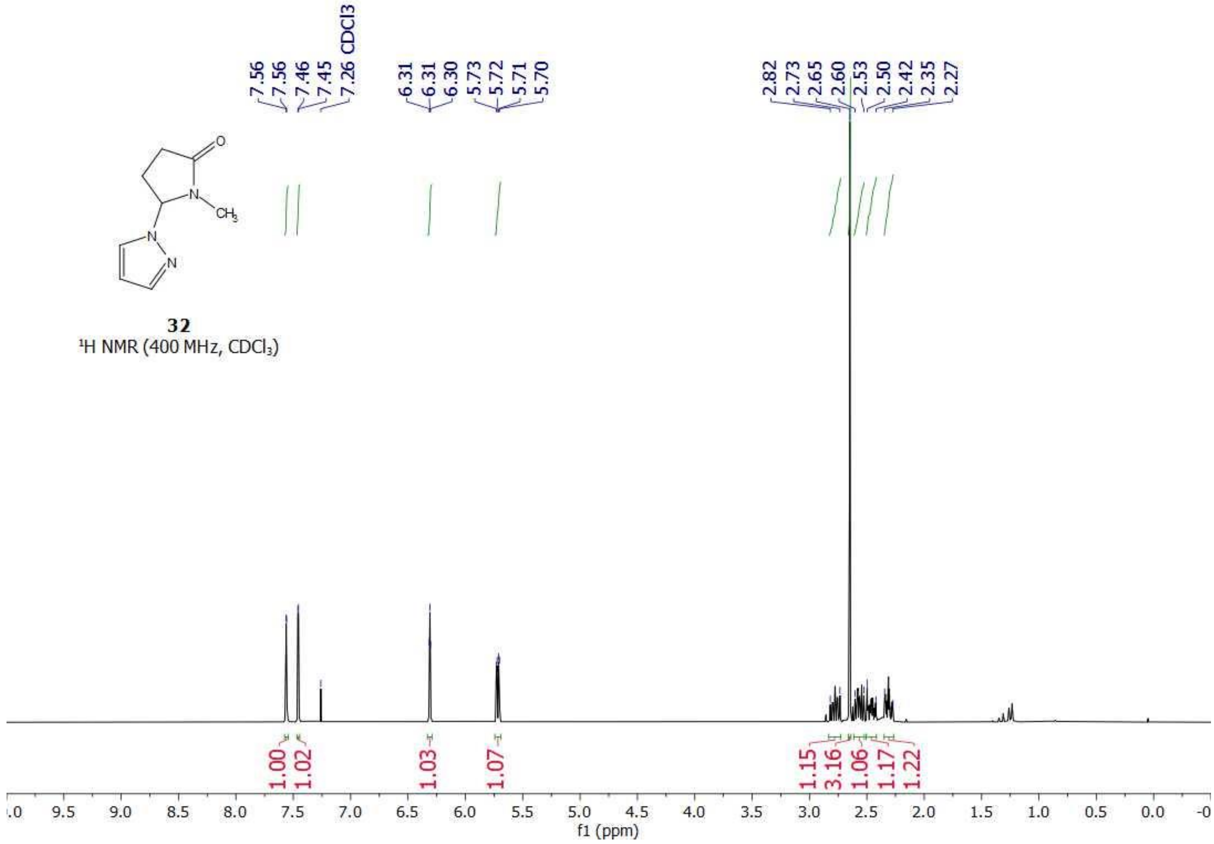
7.56
7.56
7.46
7.45
7.26 CDCl3
6.31
6.31
6.30
5.73
5.72
5.71
5.70
2.82
2.73
2.65
2.60
2.53
2.50
2.42
2.35
2.27
O
N
CH3
N
N
32
1H NMR (400 MHz, CDCl3)
1.00
1.02
1.03
1.07
1.15
3.16
1.06
1.17
1.22
f1 (ppm)

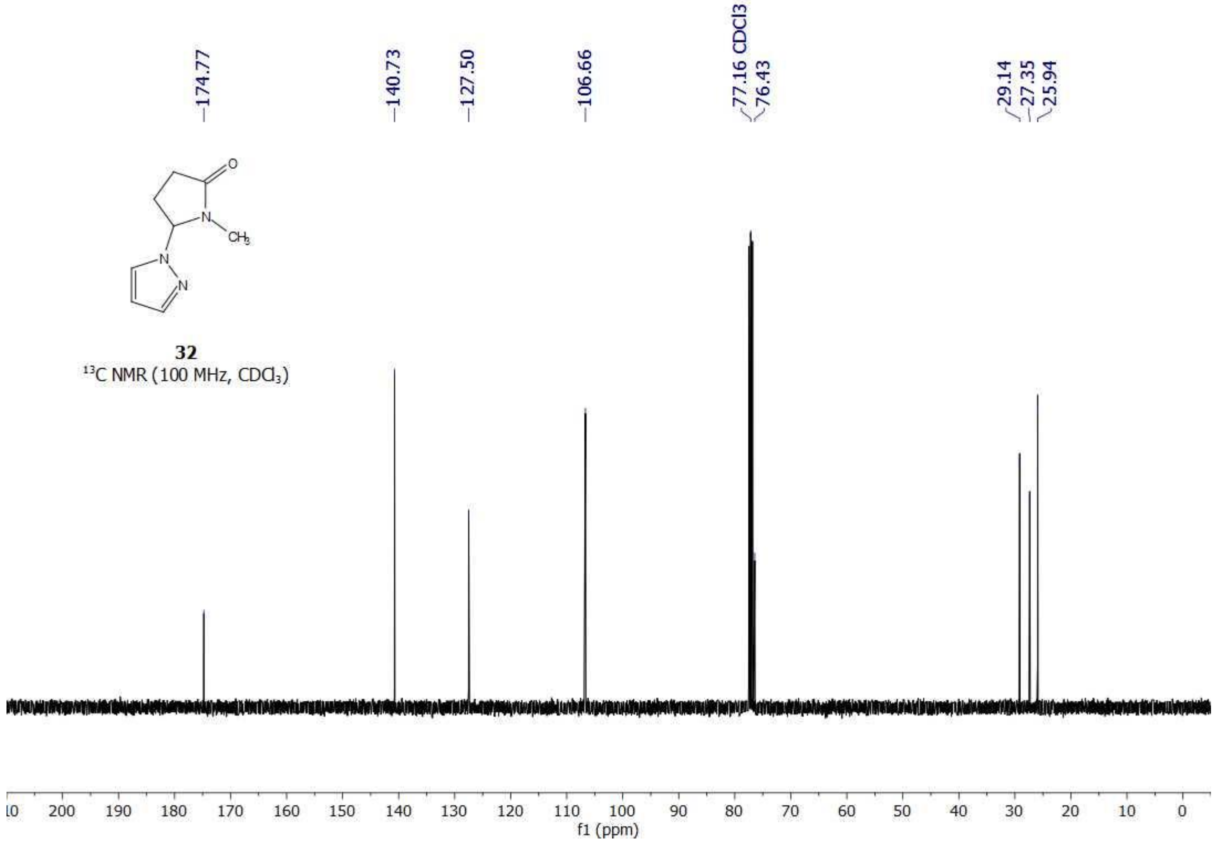
174.77
140.73
127.50
106.66
77.16 CDCl3
76.43
29.14
27.35
25.94
O
N
CH3
N
N
32
13C NMR (100 MHz, CDCl3)
f1 (ppm)

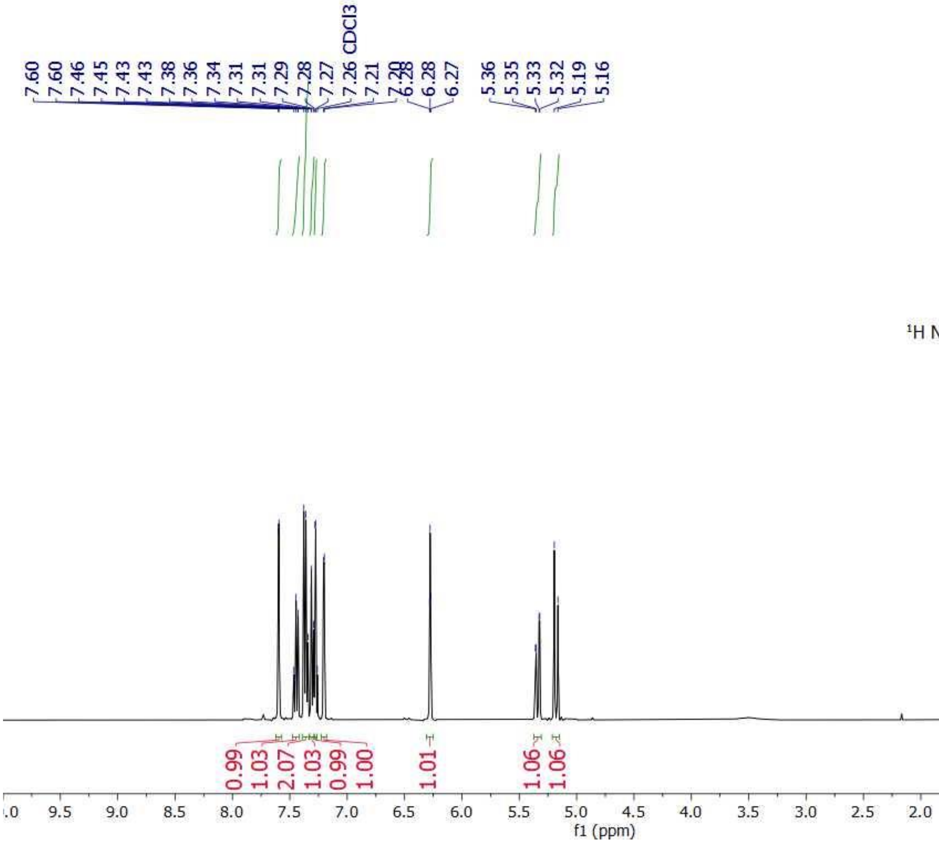

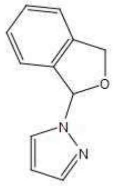

33
^{1}H NMR (400 MHz, $CDCl_3$)

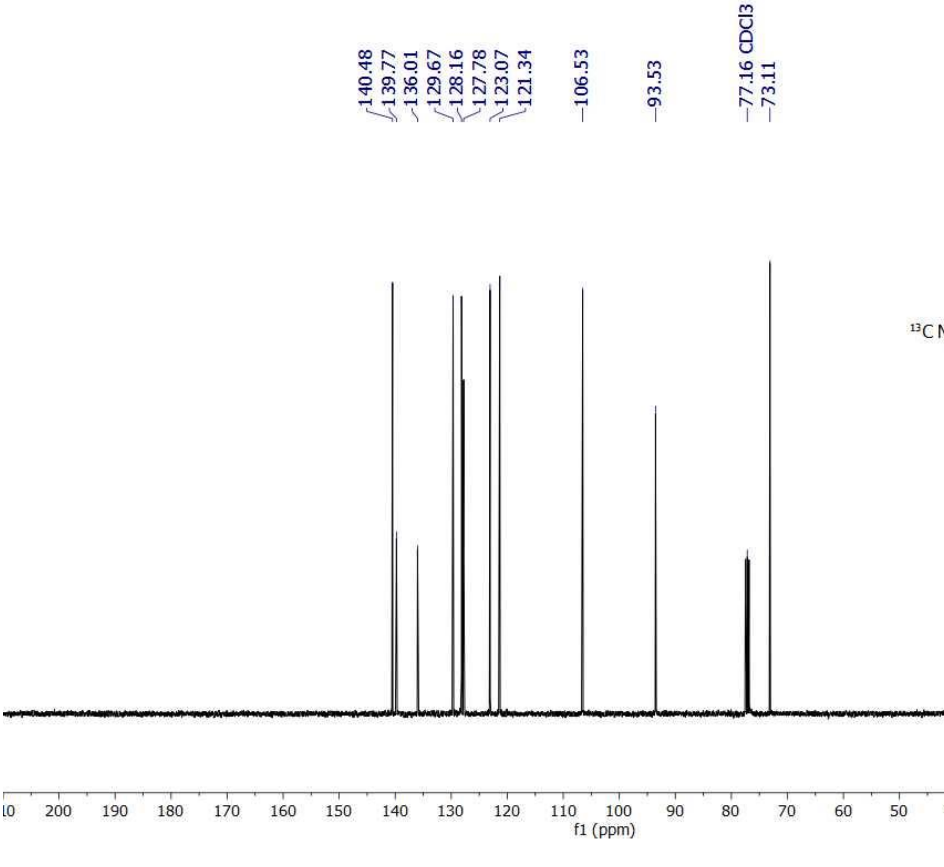

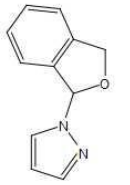

33
^{13}C NMR (100 MHz, $CDCl_3$)

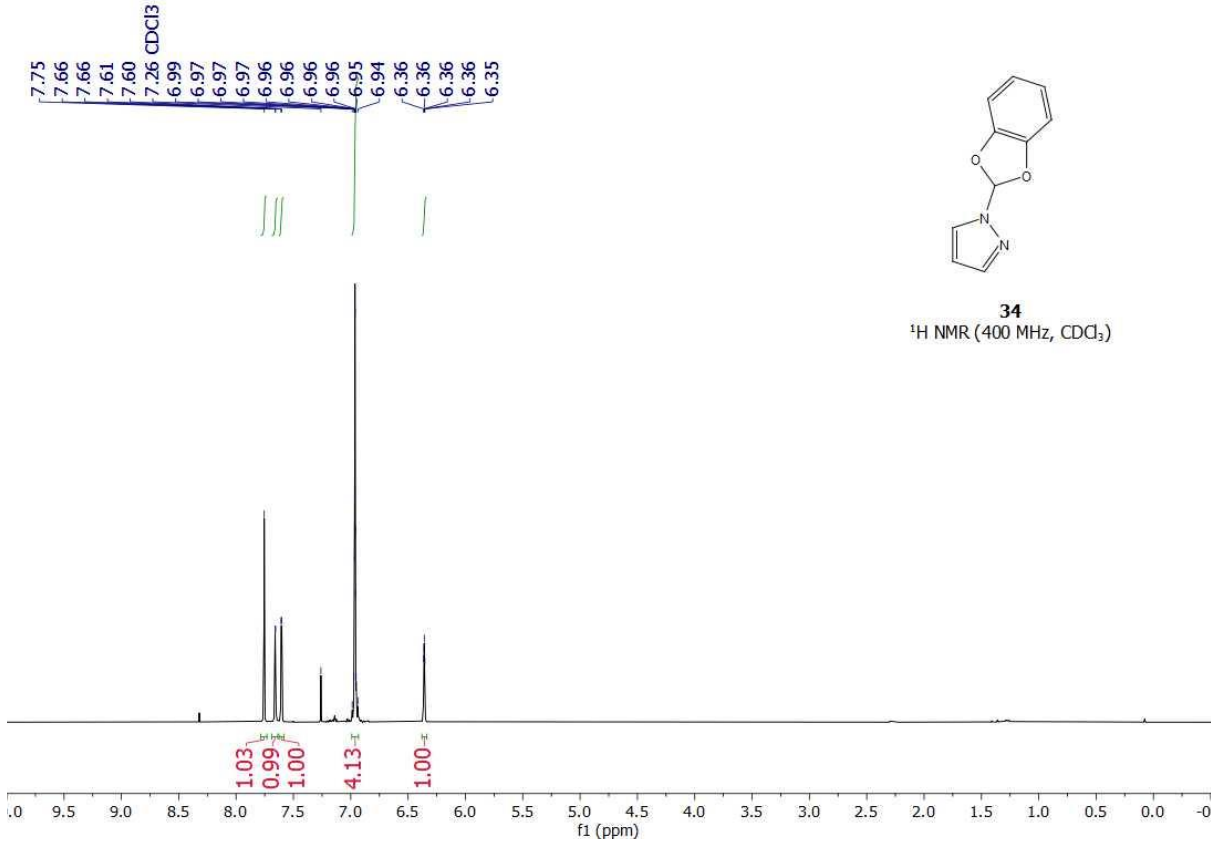

34
^{1}H NMR (400 MHz, $CDCl_3$)

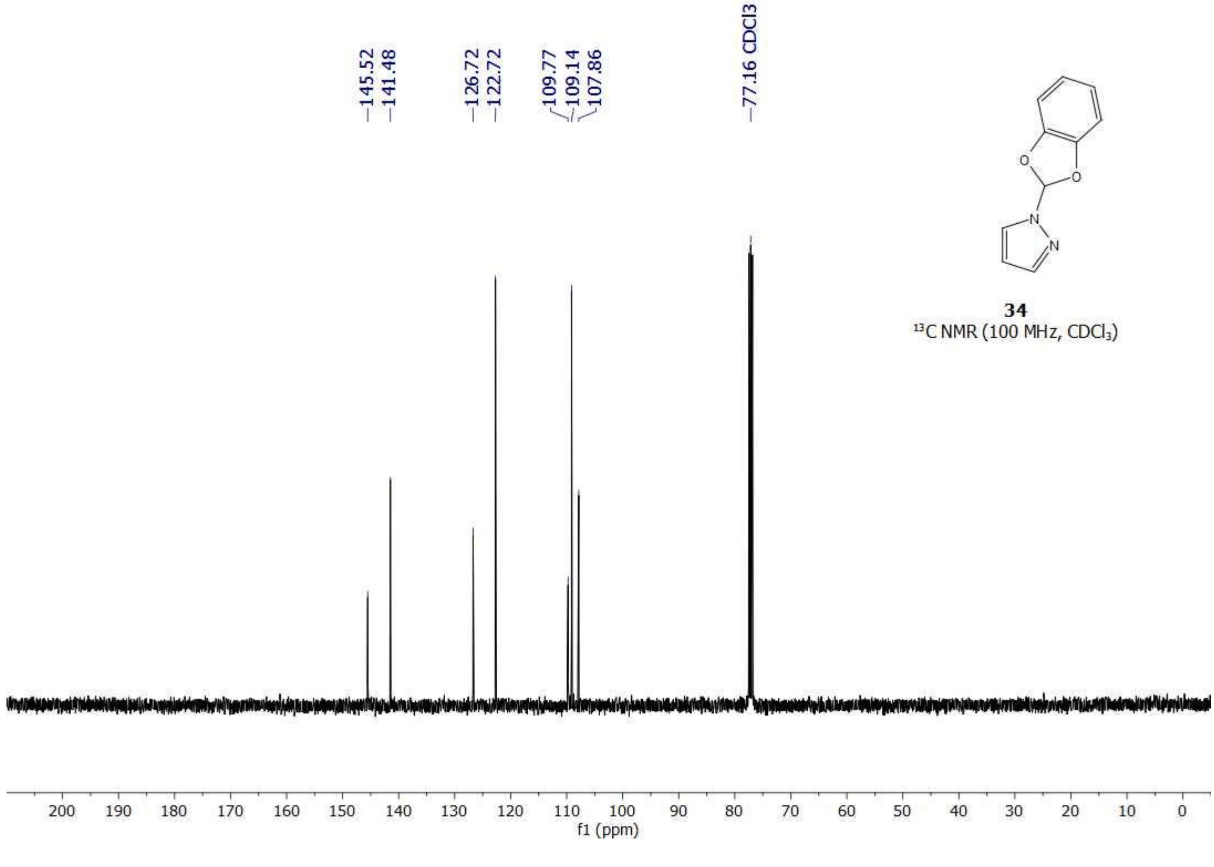

34
^{13}C NMR (100 MHz, $CDCl_3$)

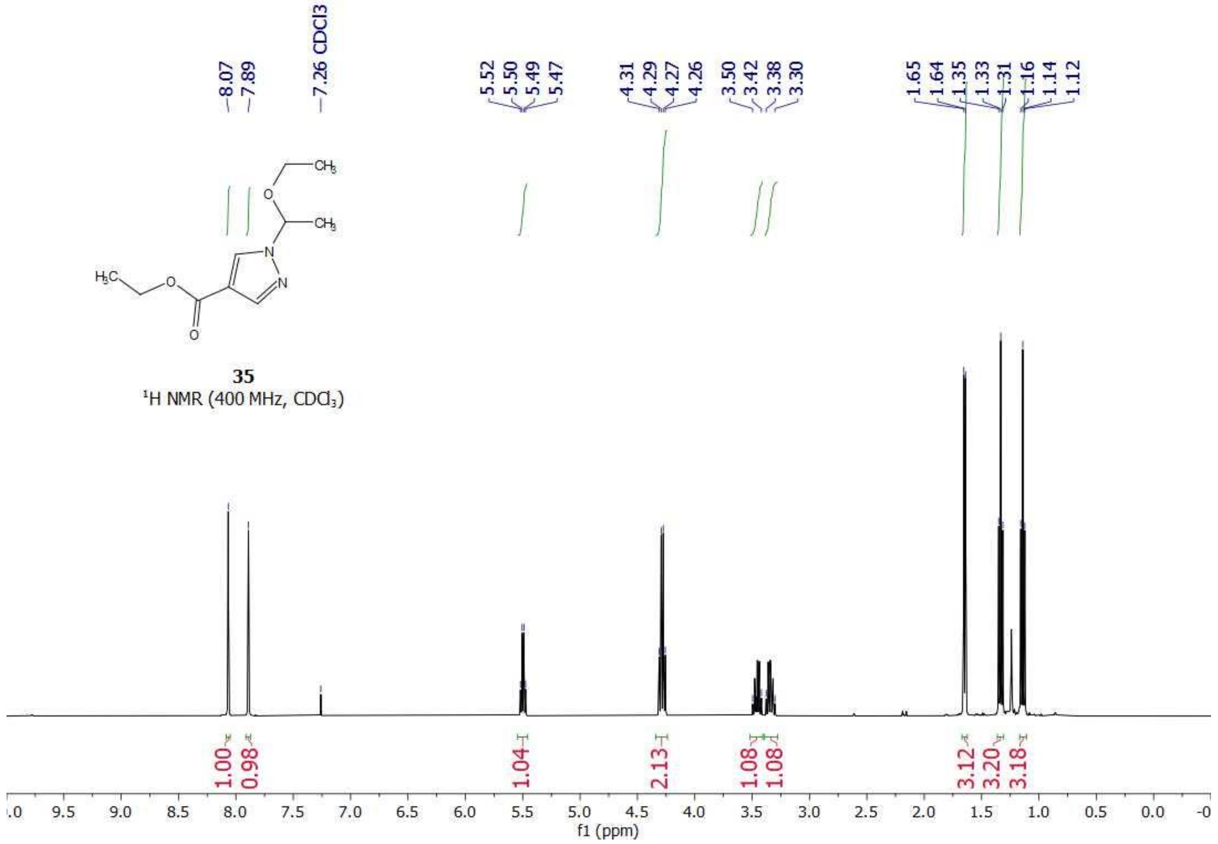

35
^{1}H NMR (400 MHz, $CDCl_3$)

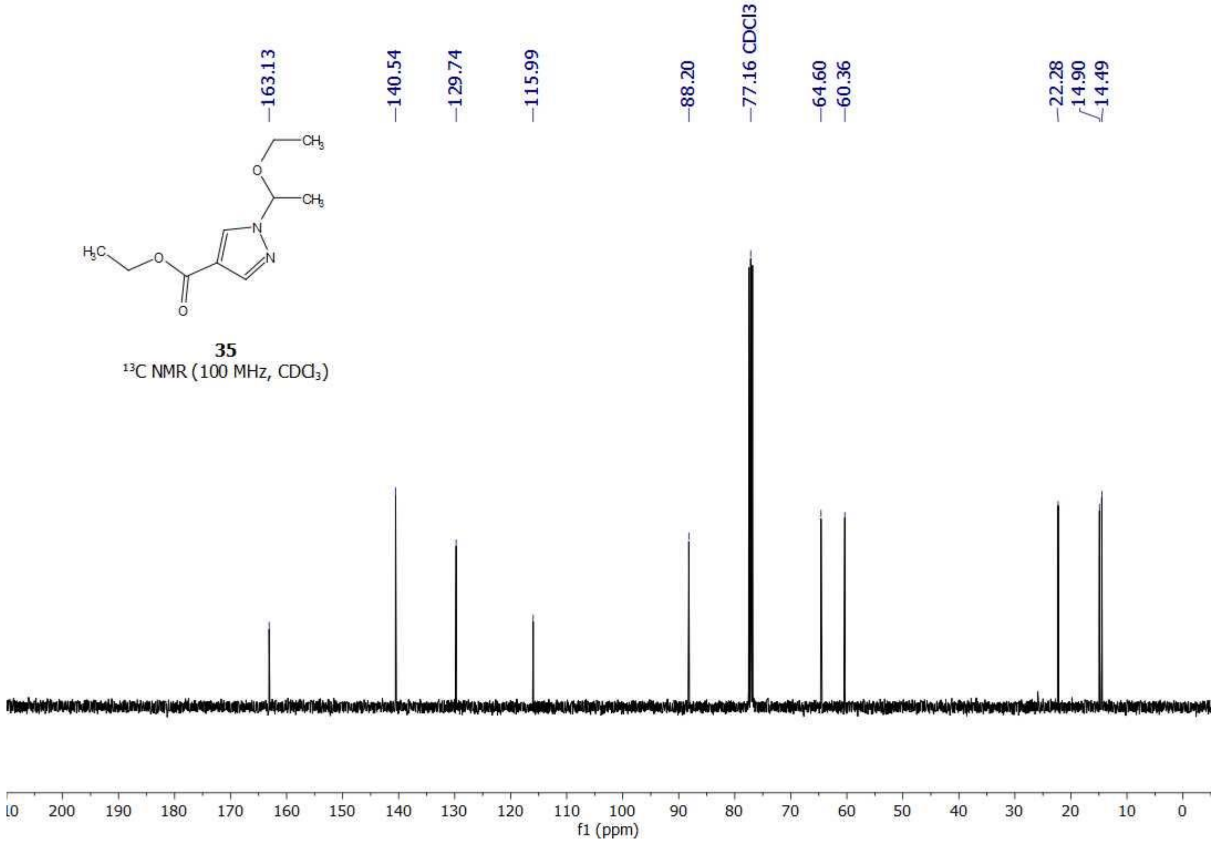

35
^{13}C NMR (100 MHz, $CDCl_3$)

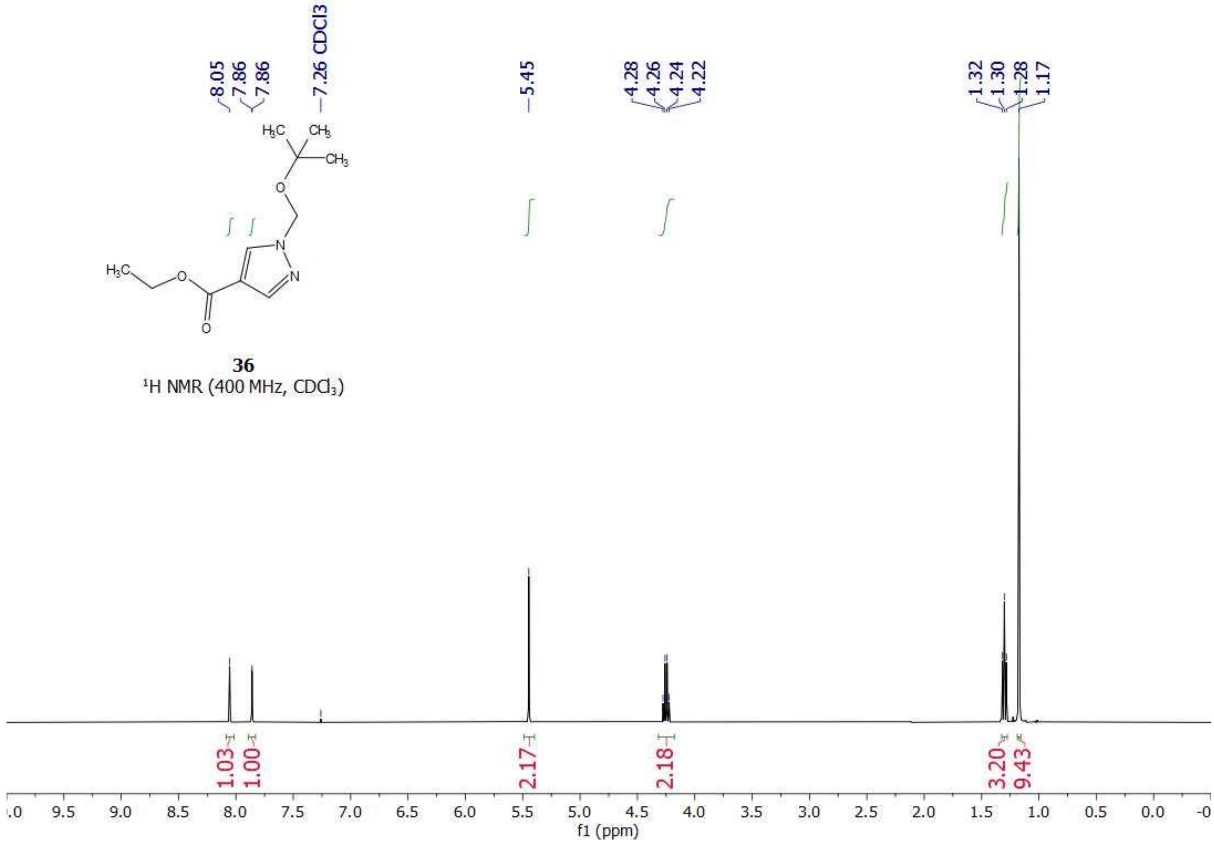

36
^{1}H NMR (400 MHz, $CDCl_3$)

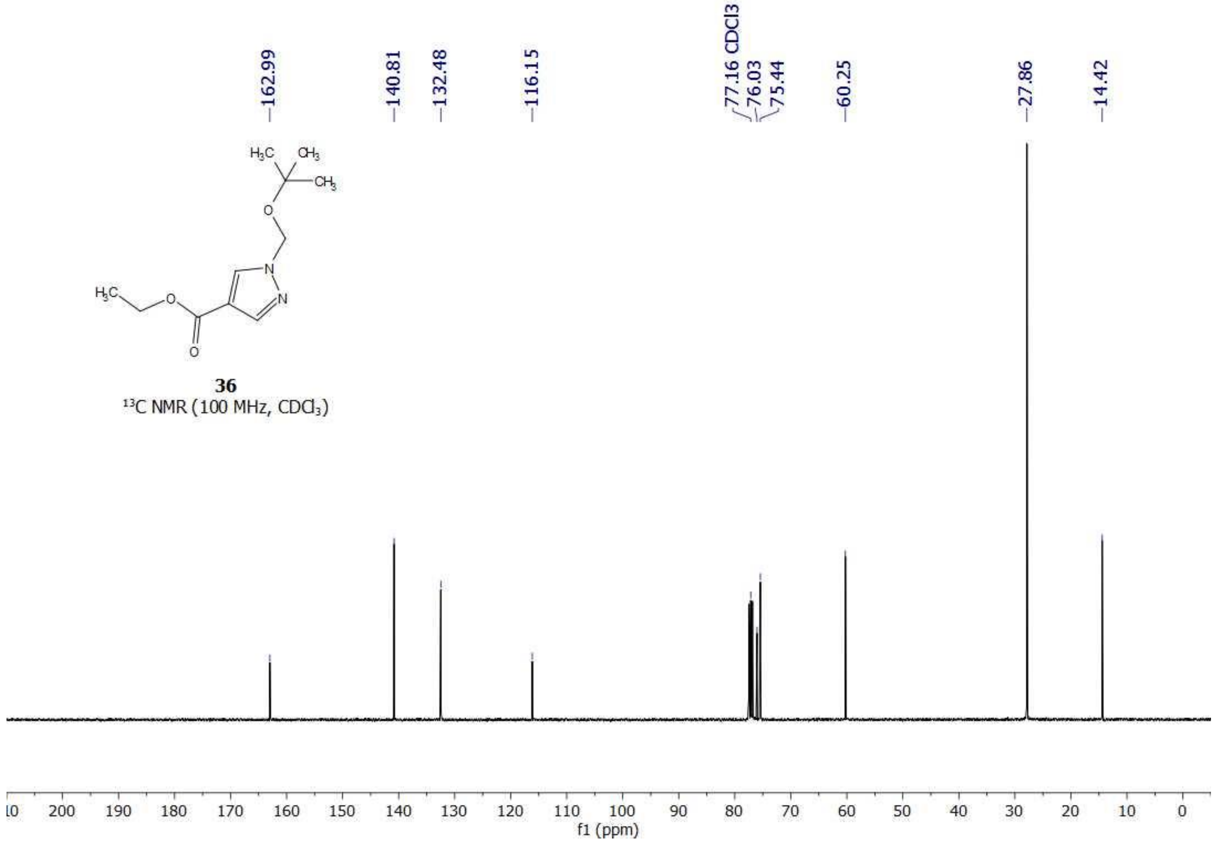

36
^{13}C NMR (100 MHz, $CDCl_3$)

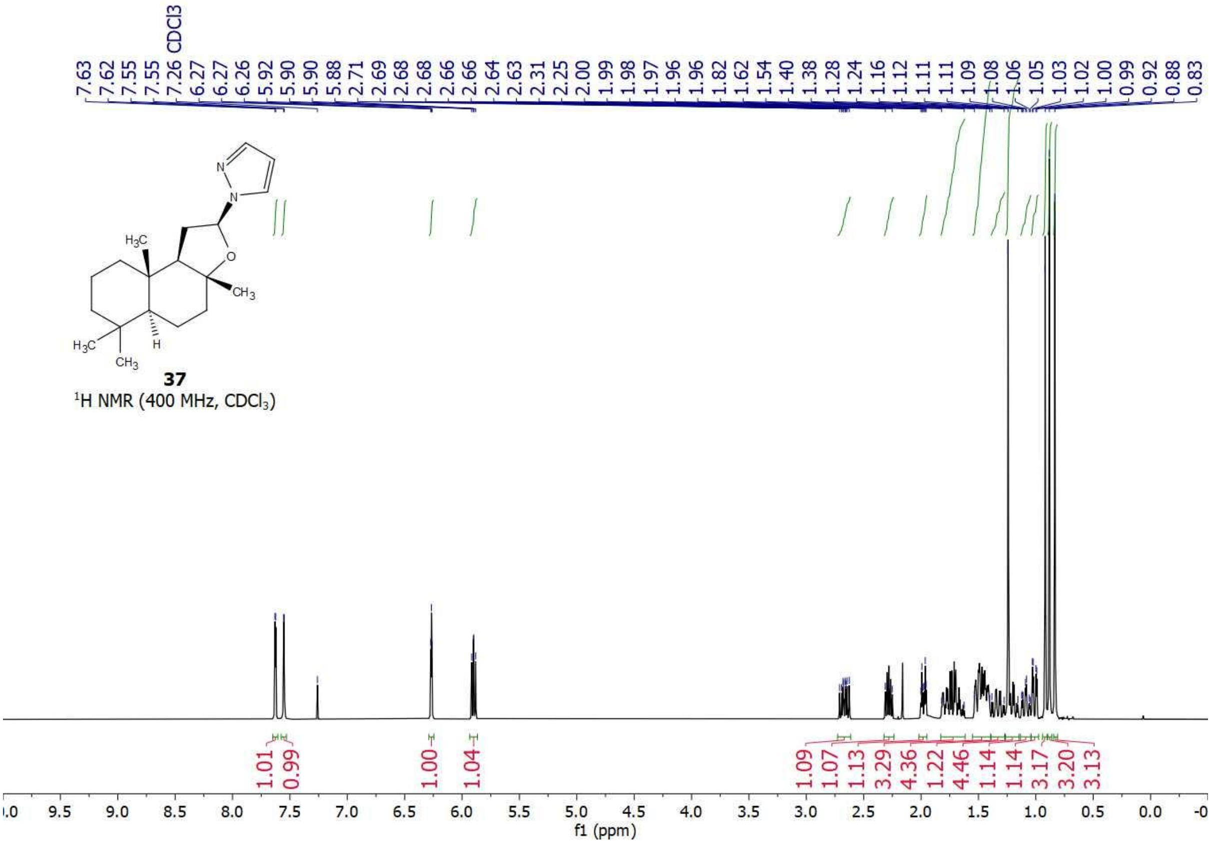

37

^{1}H NMR (400 MHz, $CDCl_3$)

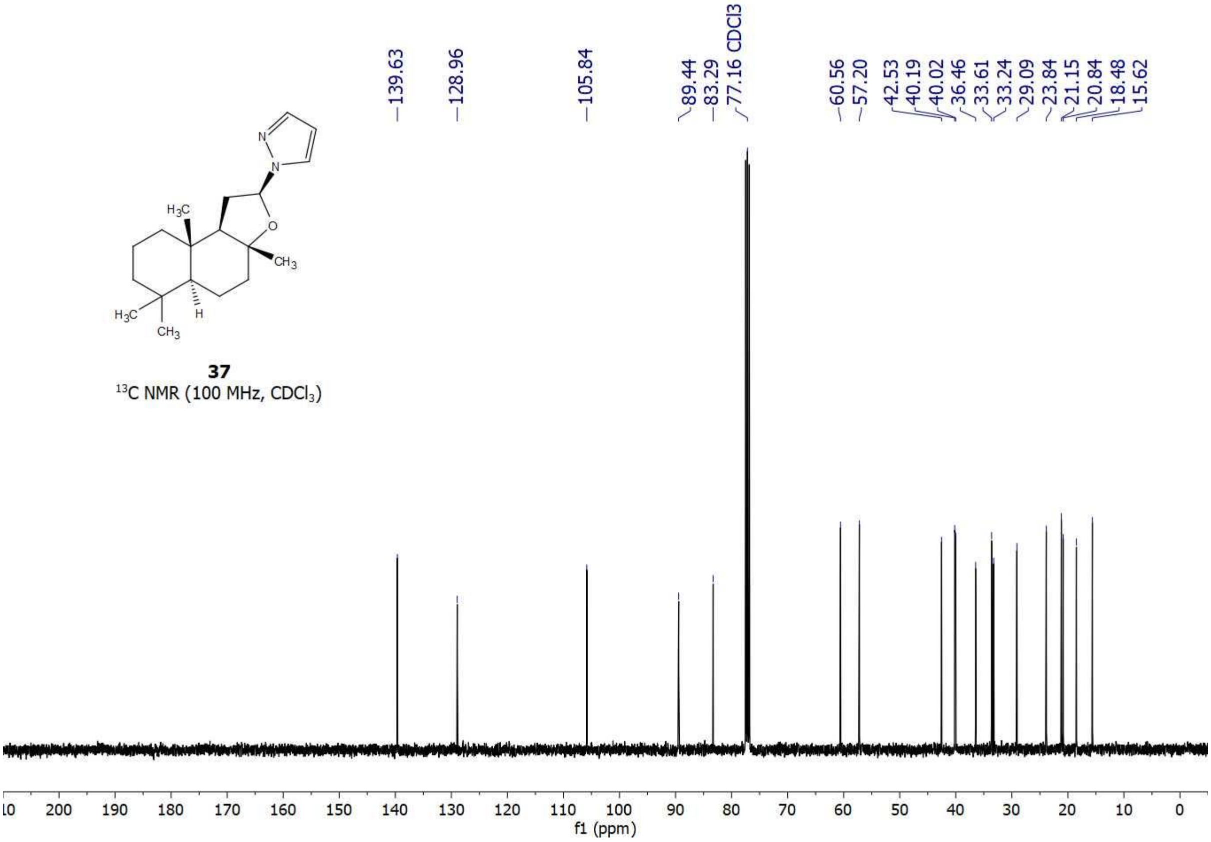

37

^{13}C NMR (100 MHz, $CDCl_3$)

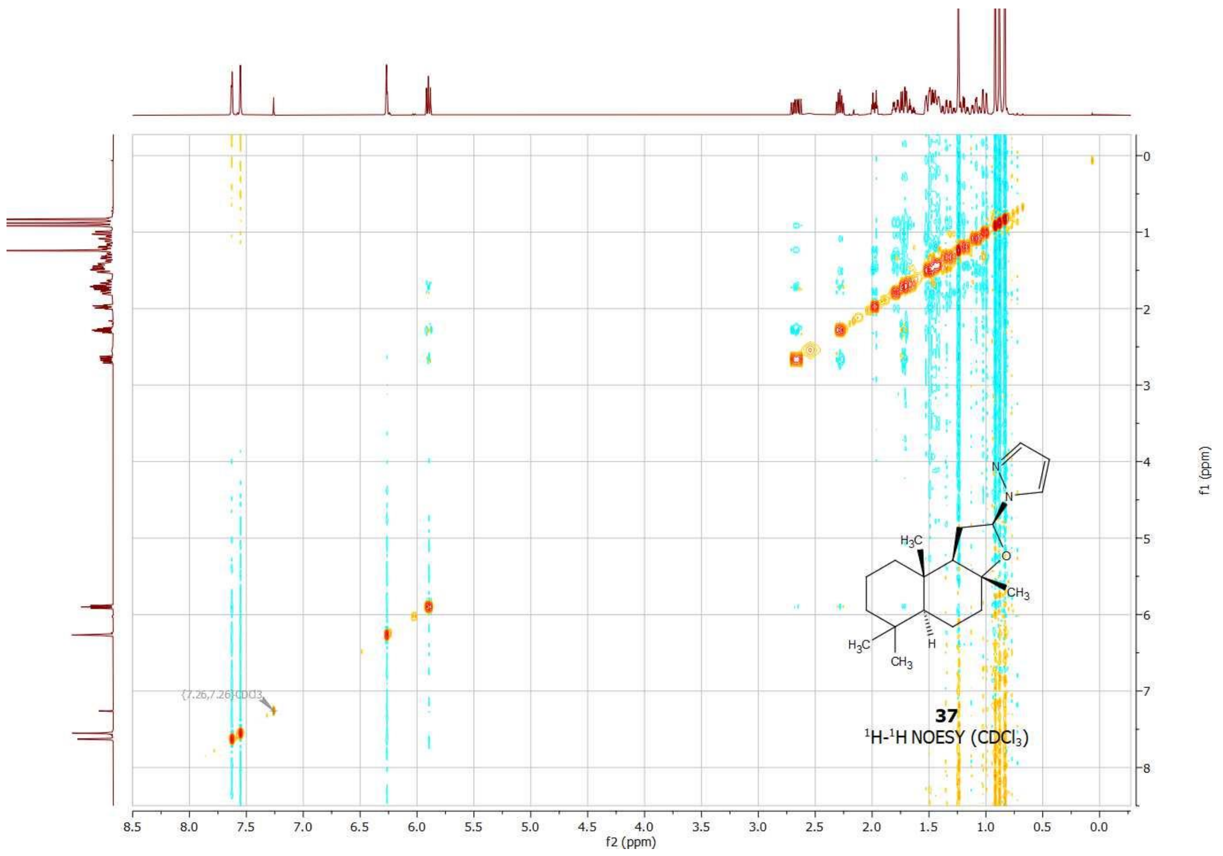
(7.26,7.26) CDCl3
f2 (ppm)
f1 (ppm)
H3C
CH3
H3C
CH3
H
N
N
O
37
1H-1H NOESY (CDCl3)

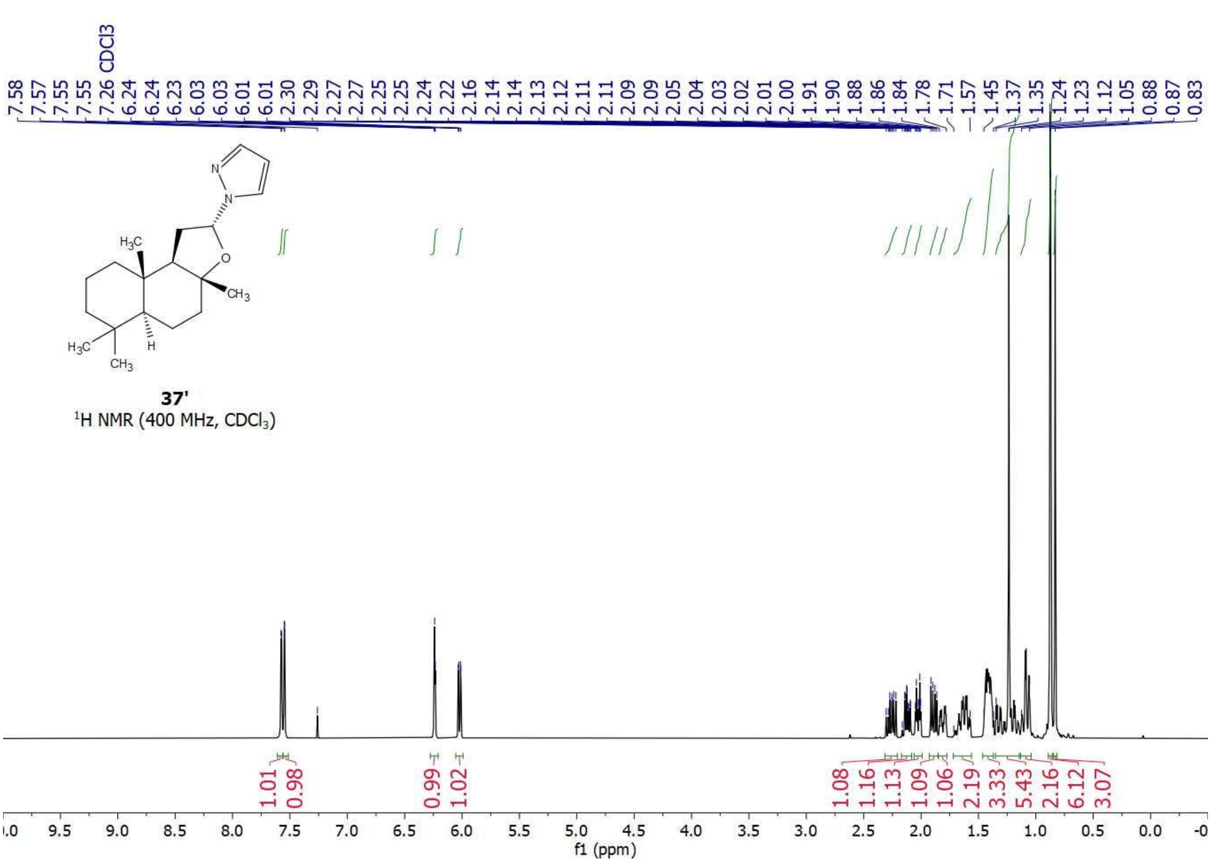
7.58 7.57 7.55 7.55 7.26 CDCl3 6.24 6.24 6.23 6.03 6.03 6.01 6.01 2.30 2.29 2.27 2.27 2.25 2.25 2.24 2.22 2.16 2.14 2.14 2.13 2.12 2.11 2.11 2.09 2.09 2.05 2.04 2.03 2.02 2.01 2.00 1.91 1.90 1.88 1.86 1.84 1.78 1.71 1.57 1.45 1.37 1.35 1.24 1.23 1.12 1.05 0.88 0.87 0.83
37'
1H NMR (400 MHz, CDCl3)
1.01 0.98 0.99 1.02 1.08 1.16 1.13 1.09 1.06 2.19 3.33 5.43 2.16 6.12 3.07
f1 (ppm)

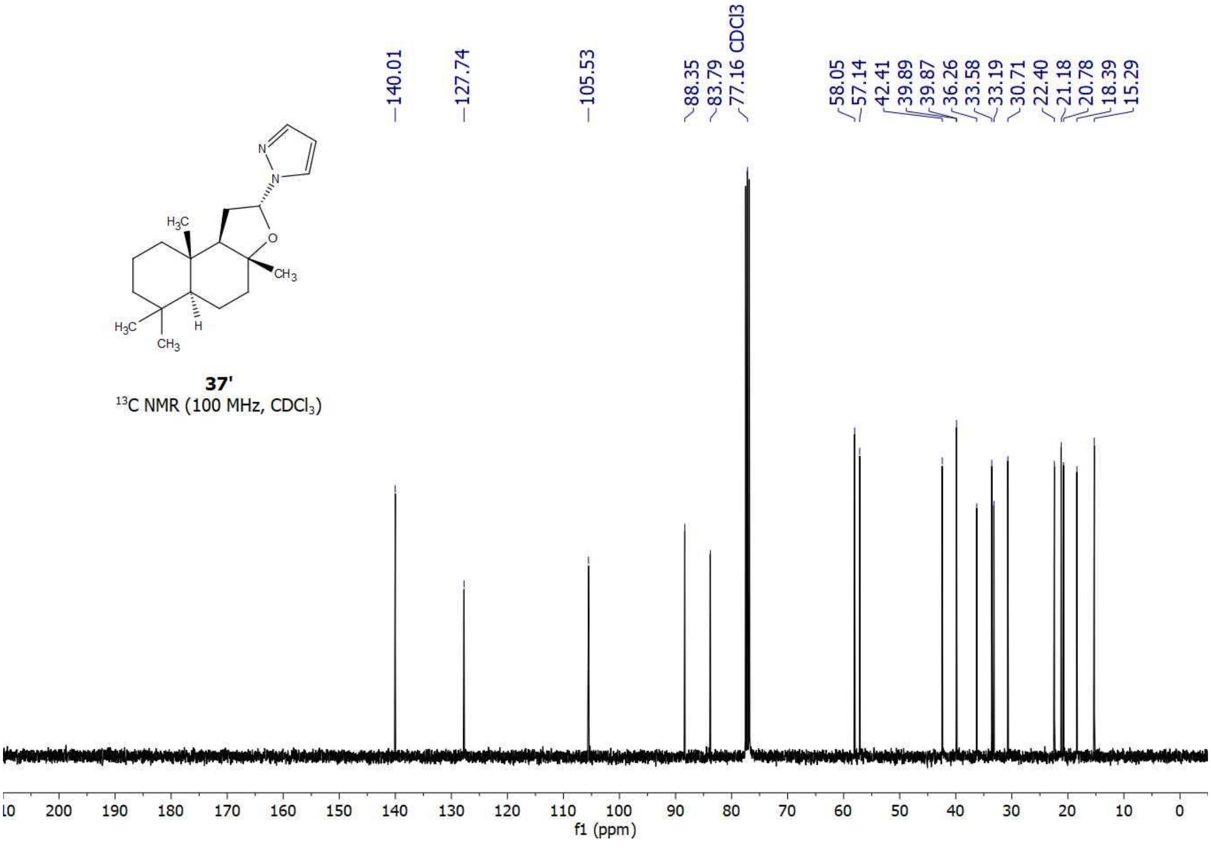
140.01 127.74 105.53 88.35 83.79 77.16 CDCl3 58.05 57.14 42.41 39.89 39.87 36.26 33.58 33.19 30.71 22.40 21.18 20.78 18.39 15.29
37'
13C NMR (100 MHz, CDCl3)
f1 (ppm)

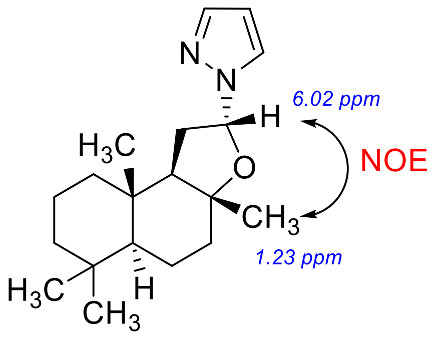
6.02 ppm
NOE
1.23 ppm

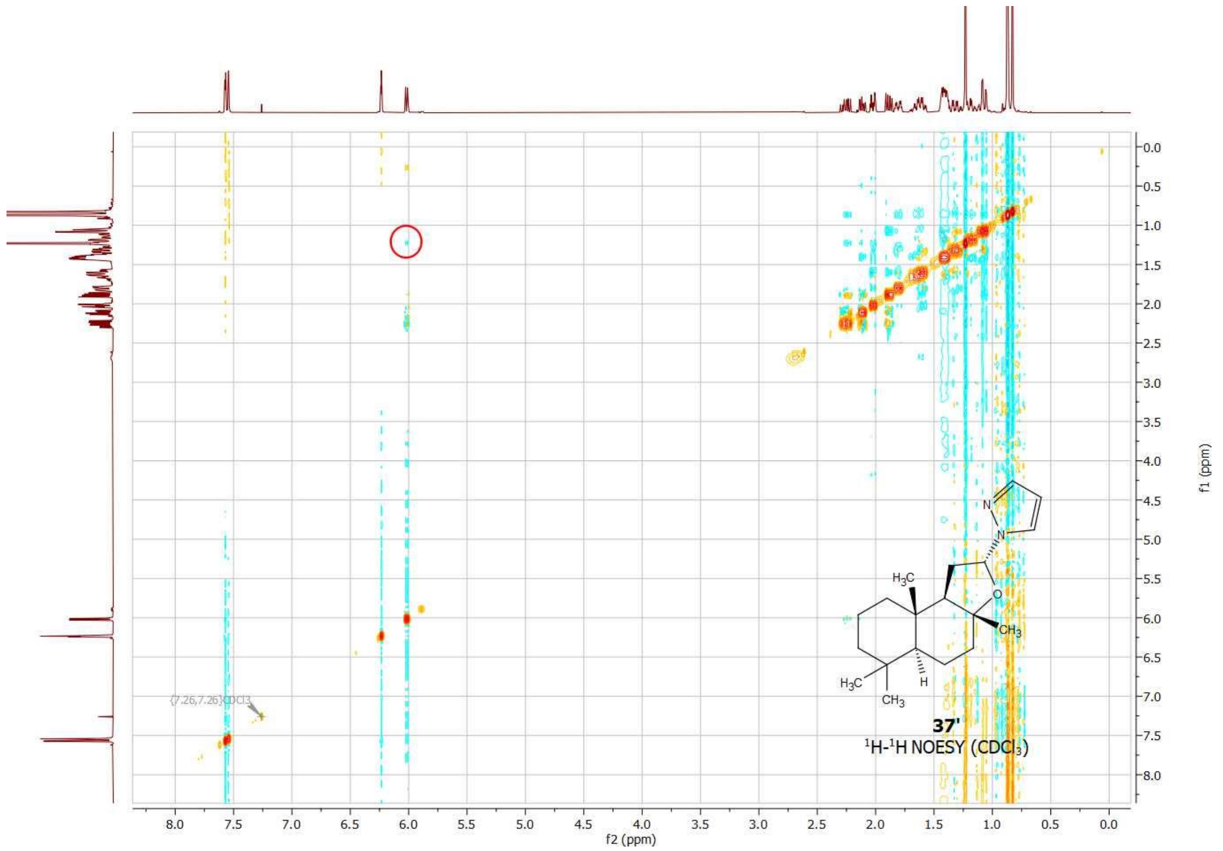
37'
^1H-^1H NOESY ($CDCl_3$)
f2 (ppm)
f1 (ppm)

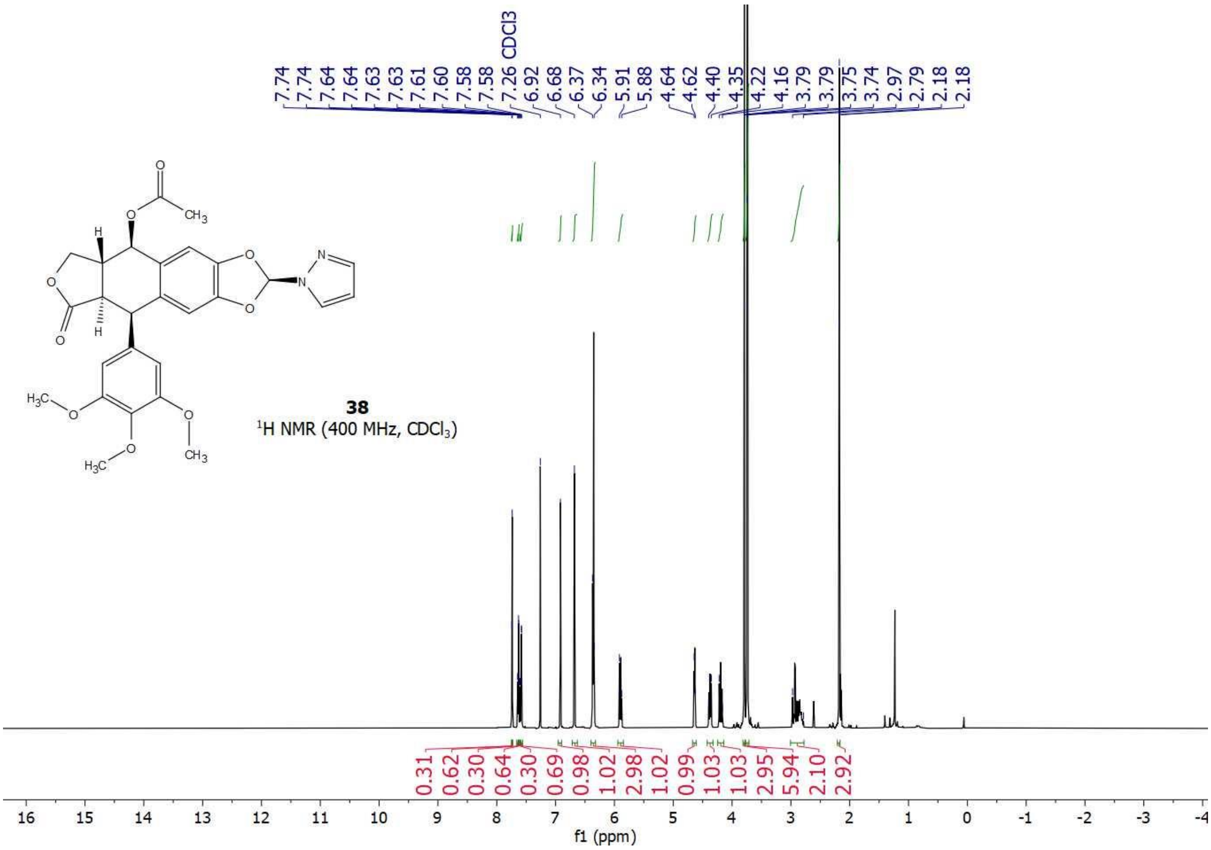

38
^{1}H NMR (400 MHz, $CDCl_3$)

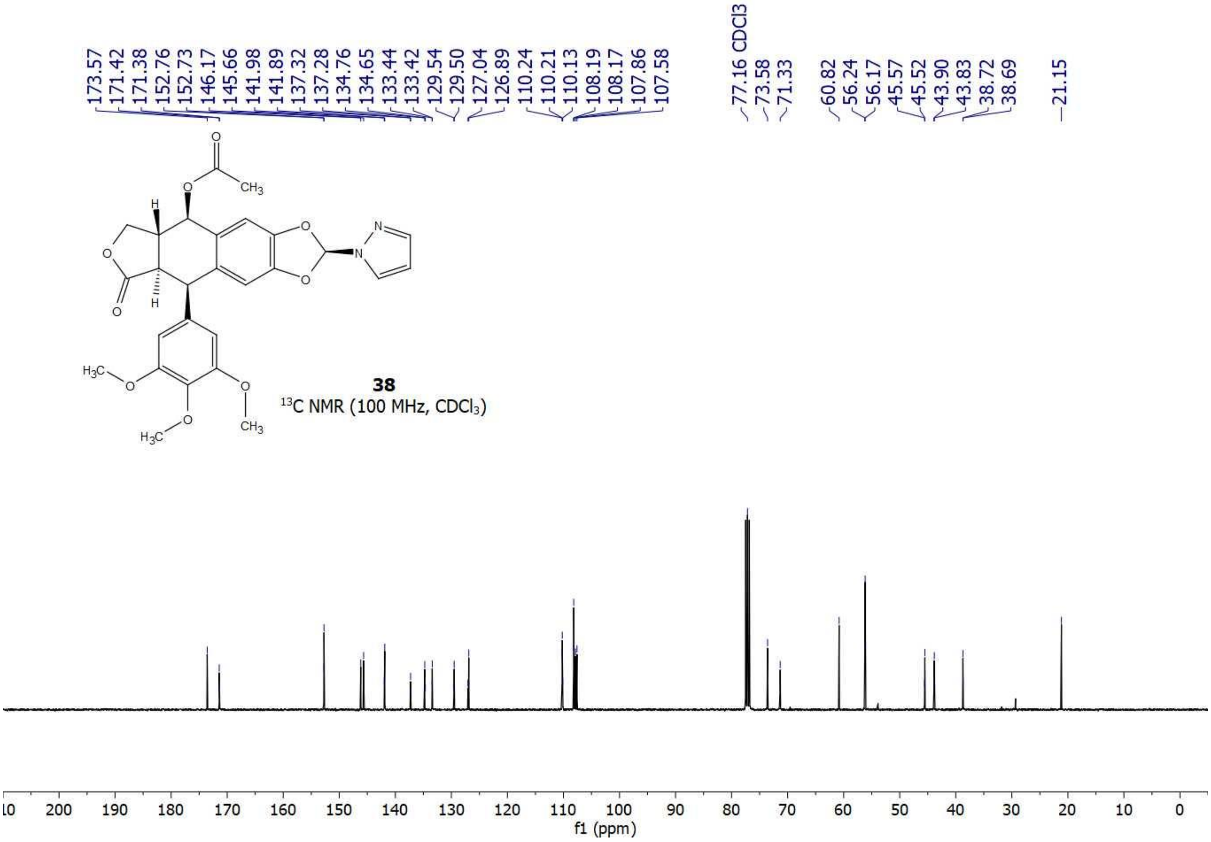

38
^{13}C NMR (100 MHz, $CDCl_3$)

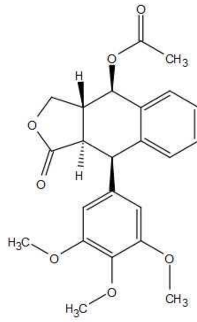

38
^{13}C DEPT 135 (100 MHz, $CDCl_3$)

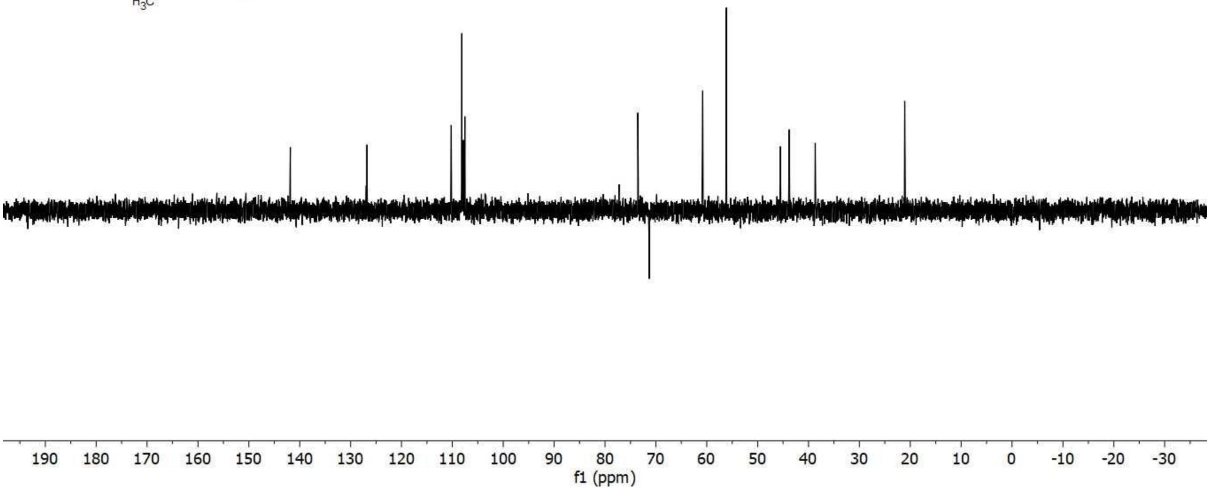

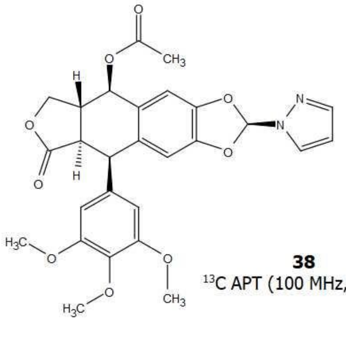

38
^{13}C APT (100 MHz, $CDCl_3$)

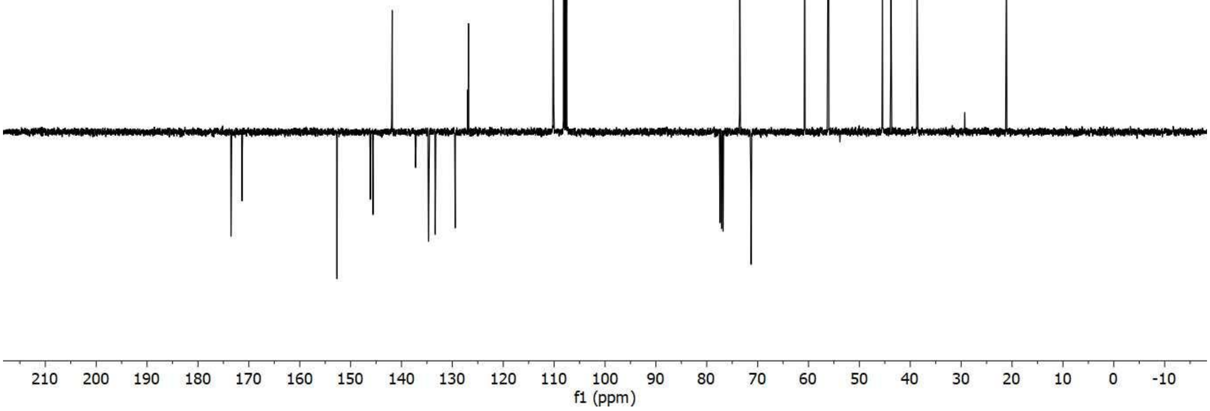

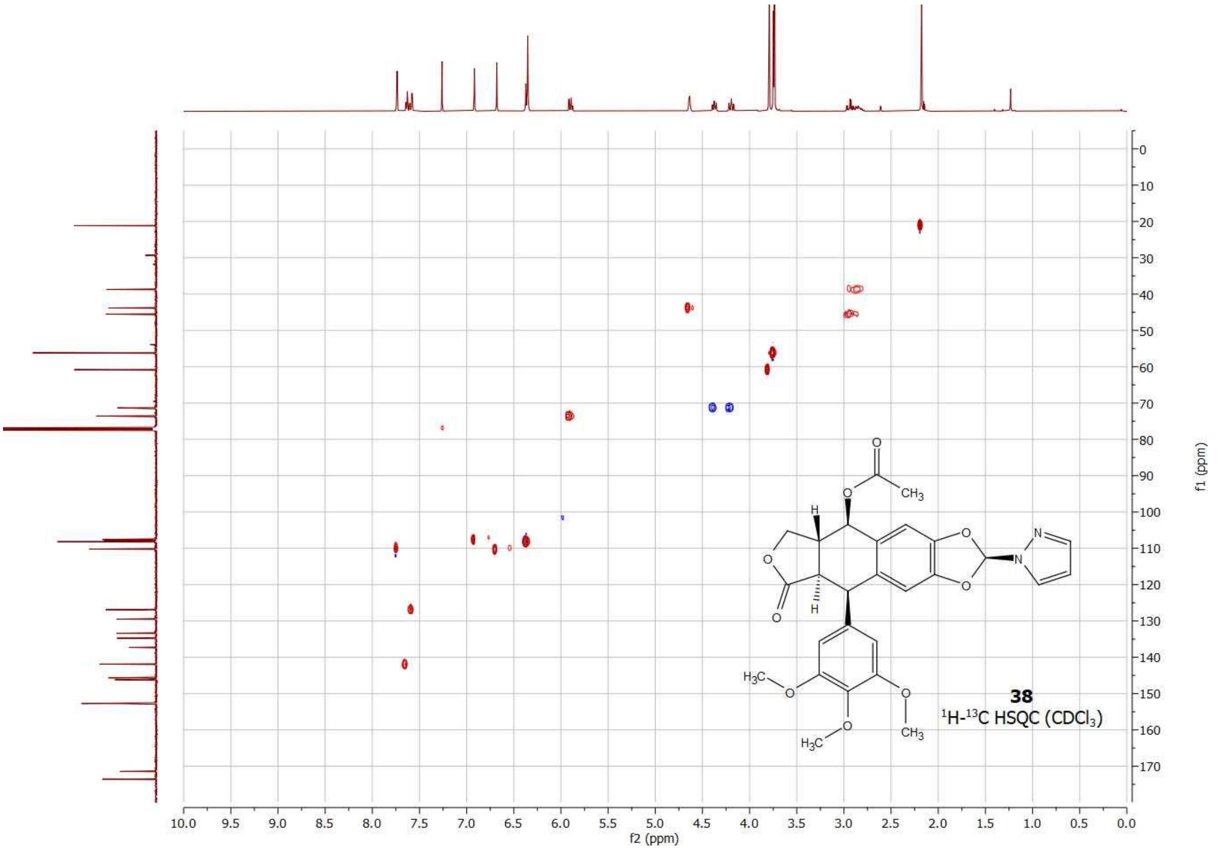
f2 (ppm)
f1 (ppm)
38
^{1}H-^{13}C HSQC ($CDCl_3$)

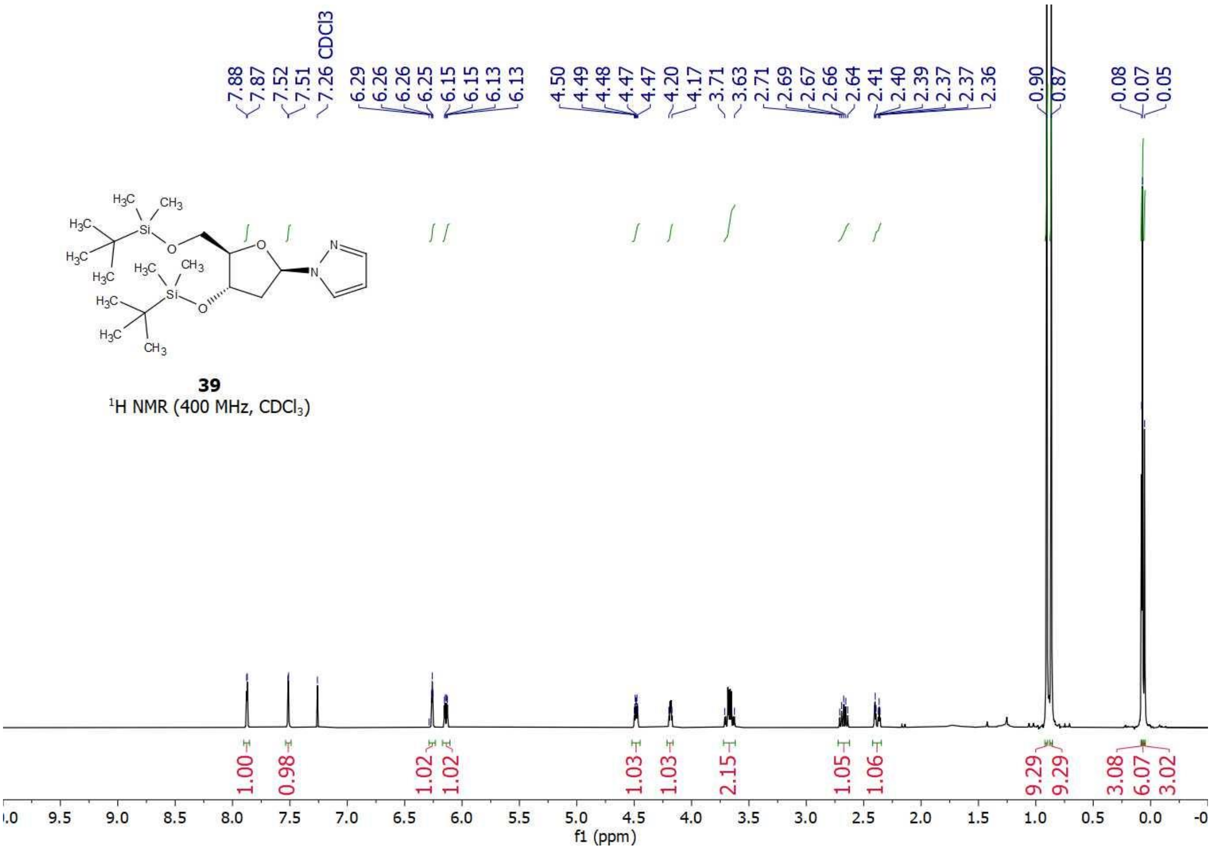

39
^{1}H NMR (400 MHz, $CDCl_3$)

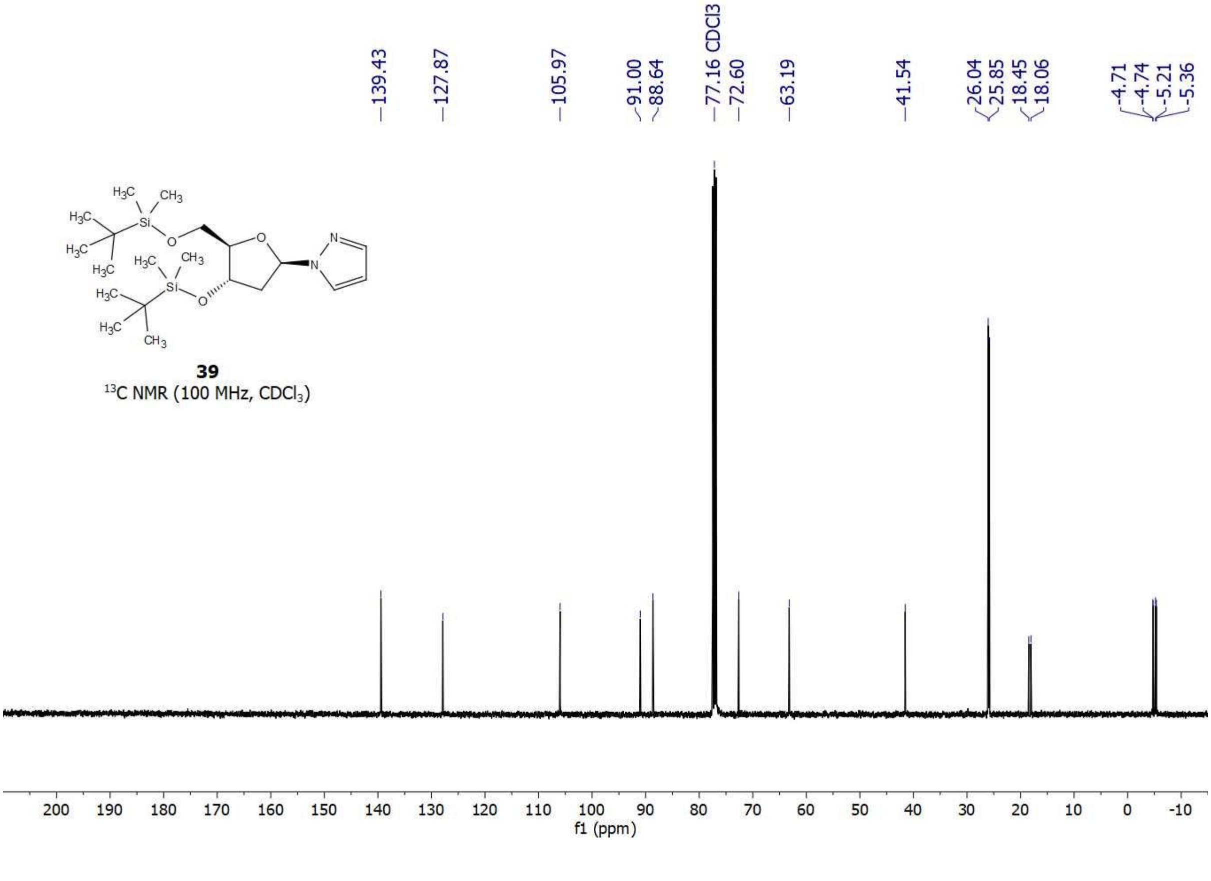

39
^{13}C NMR (100 MHz, $CDCl_3$)

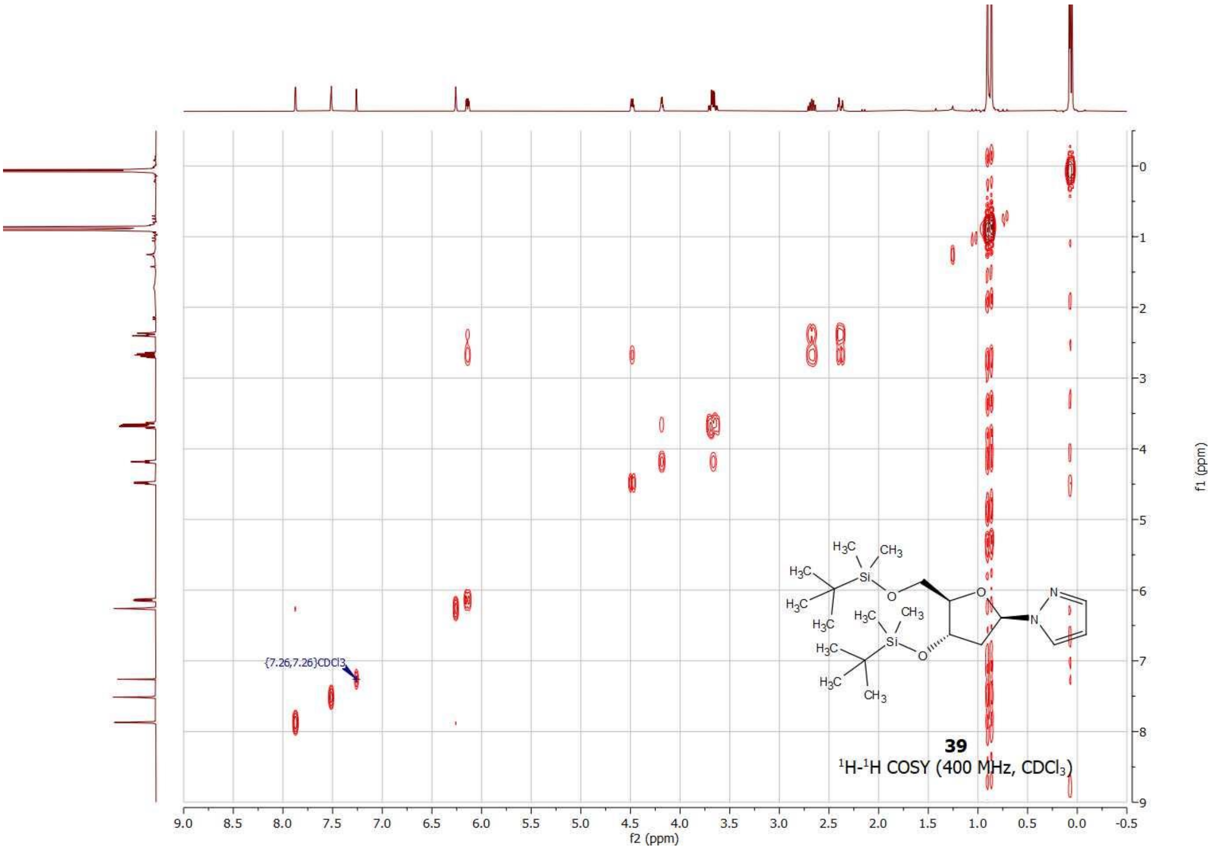

39
^1H-^1H COSY (400 MHz, $CDCl_3$)

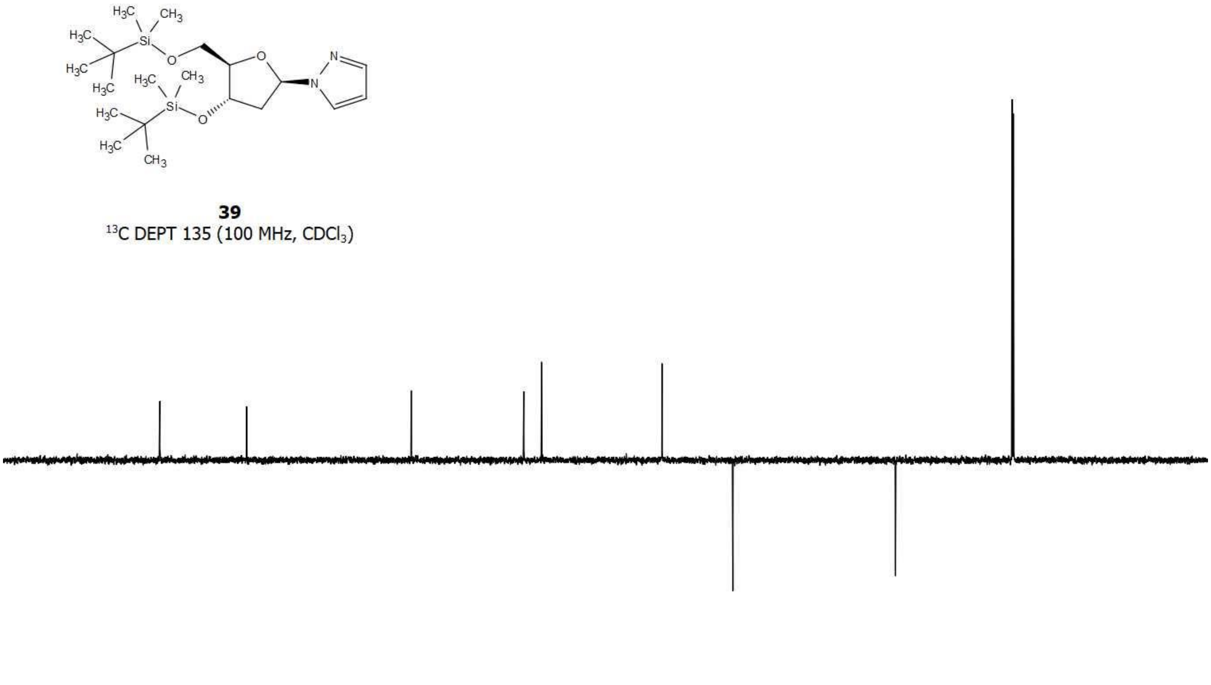

39
^{13}C DEPT 135 (100 MHz, $CDCl_3$)

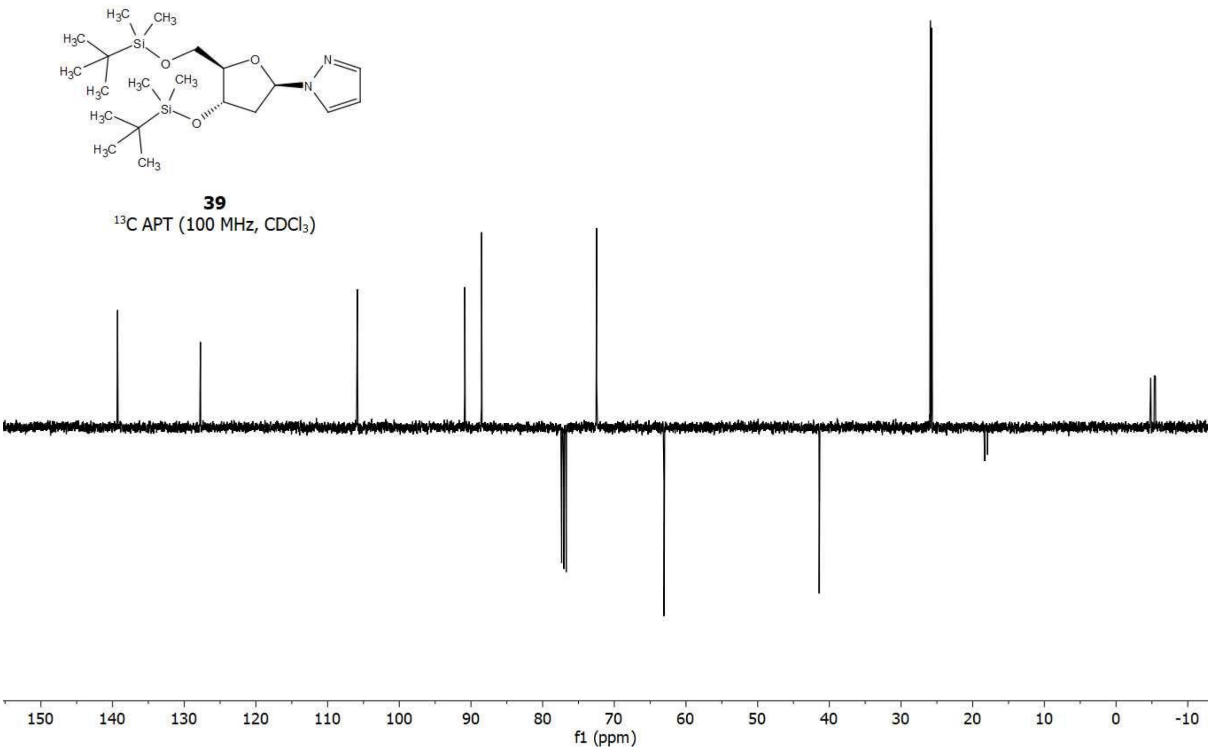

39

^{13}C APT (100 MHz, $CDCl_3$)

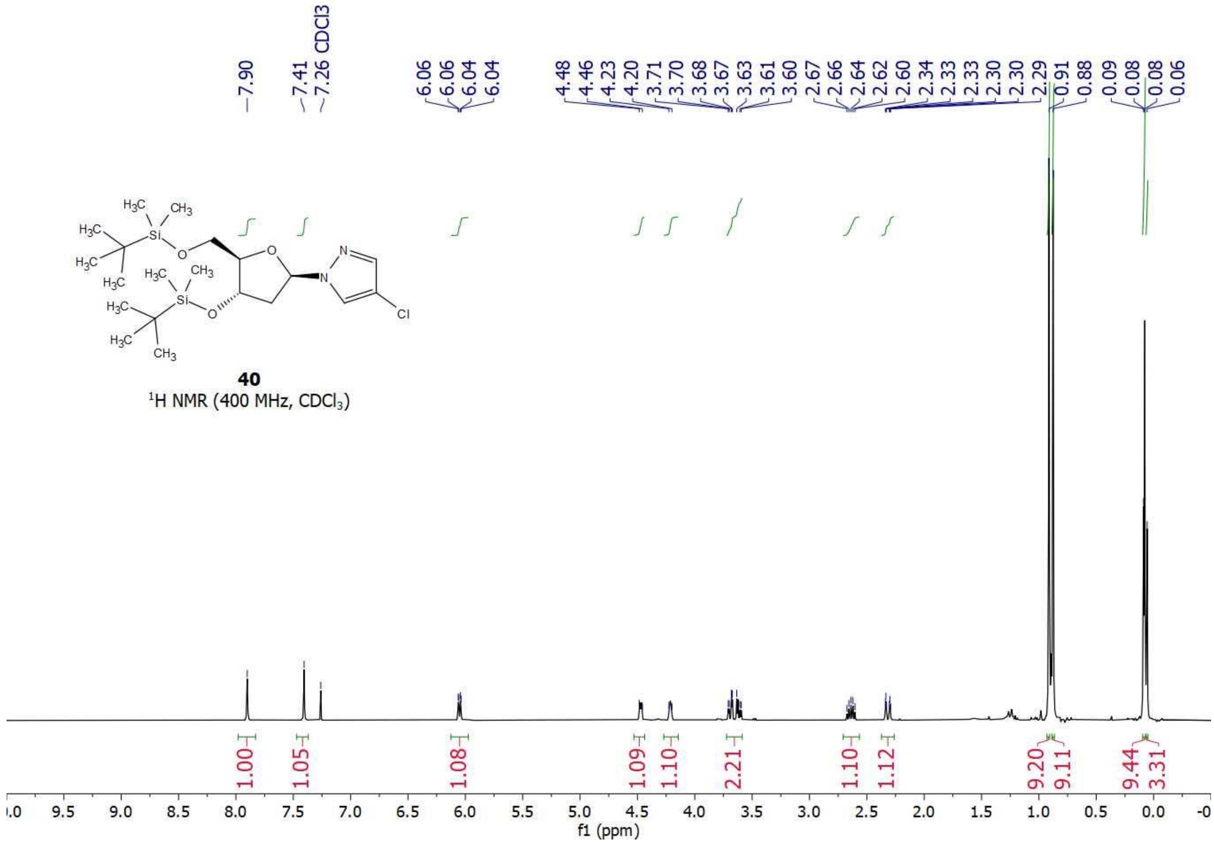

40

^{1}H NMR (400 MHz, $CDCl_3$)

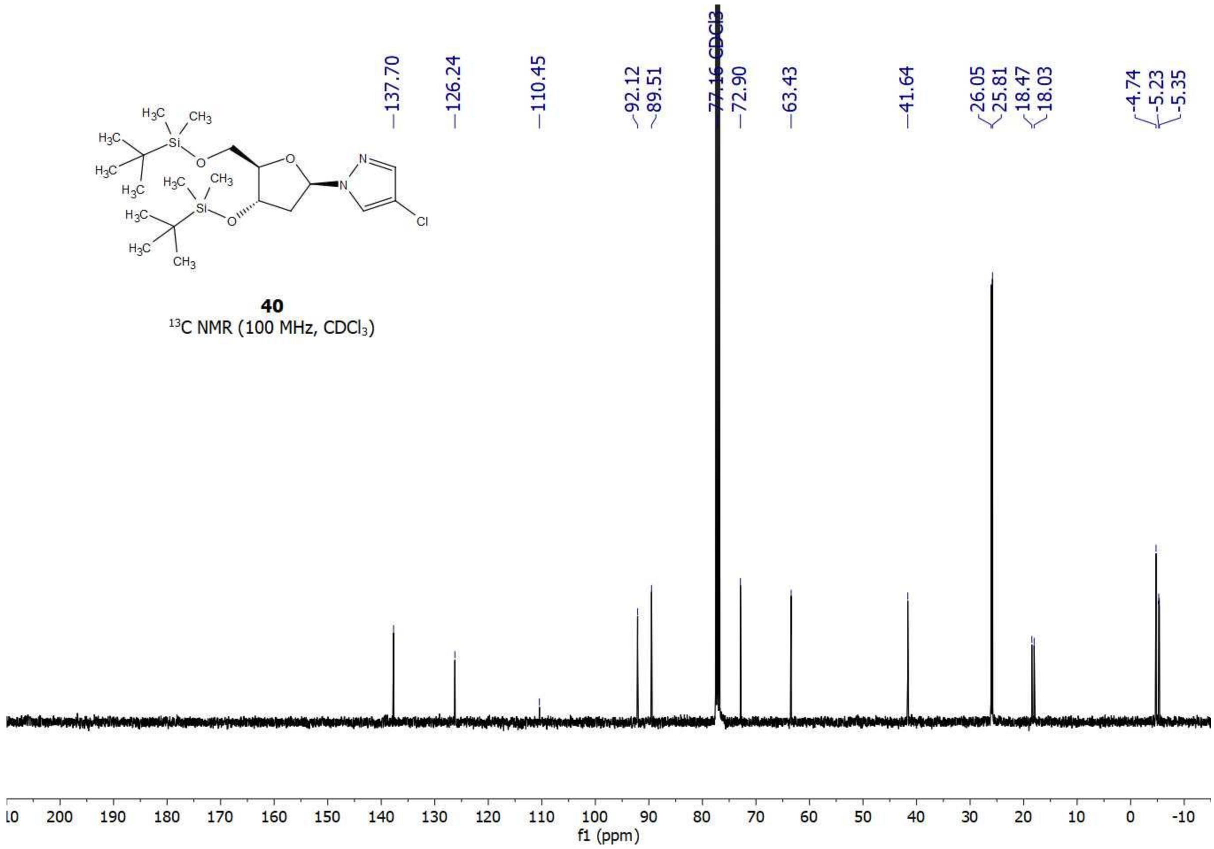

40

^{13}C NMR (100 MHz, $CDCl_3$)
